# Prenatal PM_2.5_ exposure drives epigenetic reprogramming of fetal macrophages linked to atopic dermatitis

**DOI:** 10.1038/s41467-026-76298-6

**Published:** 2026-08-08

**Authors:** Dae Yeol Yang, Song-I Yang, Yong Joo Park, Seung-Hwa Lee, Hwan-Cheol Kim, Somi Lim, Maike Herkenrath, Ah-Yoon Song, Jeong-Hyun Kim, Hyo-Bin Kim, Eom Ji Choi, Youn Ho Shin, Kyung Won Kim, Ji Soo Park, Dong In Suh, Jihyun Kim, Kangmo Ahn, Suk-Joo Choi, Soo-Young Oh, Ja-Young Kwon, Soo Hyun Kim, Jong Kwan Jun, Mi-Young Lee, Hye-Sung Won, Kwoneel Kim, Soo-Jong Hong

**Affiliations:** 1 Department of Biology, Kyung Hee University, Seoul, Republic of Korea; 2 Department of Pediatrics, Hallym University Sacred Heart Hospital, Hallym University College of Medicine, Anyang, Republic of Korea; 3 College of Pharmacy, Kyungsung University, Busan, Republic of Korea; 4 Asan Institute for Life Sciences, Asan Medical Center, University of Ulsan College of Medicine, Seoul, Republic of Korea; 5 Department of Occupational and Environmental Medicine, Inha University School of Medicine, Incheon, Republic of Korea; 6 Department of Mathematics and Computer Science, Freie Universität Berlin, Berlin, Germany; 7 Department of Pediatrics, Inje University Sanggye Paik Hospital, Seoul, Republic of Korea; 8 Department of Pediatrics, CHA Gangnam Medical Center, CHA University School of Medicine, Seoul, Republic of Korea; 9 Department of Pediatrics, Kyung Hee University Hospital, Seoul, Republic of Korea; 10 Department of Pediatrics, Yonsei University College of Medicine, Seoul, Republic of Korea; 11 Department of Pediatrics, Seoul National University College of Medicine, Seoul, Republic of Korea; 12 Department of Pediatrics, Samsung Medical Center, Sungkyunkwan University School of Medicine, Seoul, Republic of Korea; 13 Department of Obstetrics and Gynecology, Samsung Medical Center, Sungkyunkwan University School of Medicine, Seoul, Republic of Korea; 14 Department of Obstetrics and Gynecology, Yonsei University College of Medicine, Seoul, Republic of Korea; 15 Department of Obstetrics and Gynecology, CHA Gangnam Medical Center, CHA University School of Medicine, Seoul, Republic of Korea; 16 Department of Obstetrics and Gynecology, Ewha Womans University Mokdong Hospital, Seoul, Korea; 17 Department of Obstetrics and Gynecology, Asan Medical Center, University of Ulsan College of Medicine, Seoul, Republic of Korea; 18 Department of Pediatrics, Humidifier Disinfectant Health Center, National Medical Center, Seoul, Republic of Korea

**Keywords:** Mechanisms of disease, DNA methylation, Atopic dermatitis

## Abstract

Prenatal environmental exposures are increasingly recognized as contributors to atopic dermatitis (AD), yet the underlying mechanisms remain unclear. Fine particulate matter (PM_2.5_), a complex mixture of airborne pollutants, has been associated with elevated risk of allergic diseases, particularly during early development. Here we show that first-trimester PM_2.5_ exposure is associated with an increased risk of AD in early childhood and induces epigenetic alteration in the placenta. Integrative multi-omics analyses, including single-cell approaches, reveal hypomethylation of *FCER1G* in fetal macrophages, leading to its sustained overexpression. This transcriptional program persists across developmental stages and re-emerges in M2 macrophages in AD skin and peripheral blood. Functional analyses demonstrate that *FCER1G*-associated networks promote NADPH oxidase–mediated reactive oxygen species signaling and Th2-related inflammatory pathways. These findings suggest that prenatal PM_2.5_ exposure induces durable epigenetic changes in immune cells, predisposing individuals to inflammatory responses that contribute to AD pathogenesis, and highlight early-life environmental exposure as a potential target for prevention and intervention.

## Abstract (web summary)

Prenatal exposure to air pollution may shape disease risk later in life. Here, the authors show that epigenetic changes in fetal immune cells persist into later life, promoting inflammatory responses and increasing the risk of atopic dermatitis.

## Introduction

Atopic dermatitis (AD), a widespread chronic inflammatory skin disorder, affects approximately 20% of children worldwide^1^. Its pathogenesis is complex, involving both genetic factors and environmental exposures^2^. Accumulating evidence has implicated air pollution, specifically fine particulate matter (PM_2.5_), in the onset and progression of AD^3^. PM_2.5_ consists of both primary particles, which are directly emitted into the atmosphere, and secondary particles, formed through atmospheric chemical reactions involving precursor gases. Primary PM_2.5_ originates from natural sources, such as dust storms and wildfires, as well as anthropogenic sources, including fossil fuel combustion, cigarette smoke, and biomass burning. Secondary PM_2.5_ is generated via reactions between precursor gases emitted from both anthropogenic and biogenic sources. The composition of PM_2.5_ is complex and heterogeneous, comprising black carbon, polycyclic aromatic hydrocarbons, aryl hydrocarbons, volatile organic compounds, heavy metals, inorganic ions, mineral dust, organic compounds, and biological materials^4^.

PM_2.5_ is known to adversely affect respiratory, cardiovascular, and immune health^5^, and exposure during critical developmental windows such as the prenatal period may increase susceptibility to AD^6^. Emerging studies, including our own, suggest that prenatal exposure to elevated PM_2.5_, especially during the first trimester, is associated with increased risk of AD by the age of one year^7^. However, existing meta-analyses yield conflicting results on the relationship between prenatal exposure to PM_2.5_ and childhood incidence of AD^8,9^.

Despite these findings, significant gaps remain in our understanding of how prenatal PM_2.5_ exposure contributes to the development of AD. The placenta, a critical mediator of fetal growth and immune development, selectively restricts certain harmful compounds, yet PM_2.5_ has been shown to cross this barrier^10,11^. Existing studies indicate that prenatal environmental exposures, including PM_2.5_, can induce lasting epigenetic alterations in the placenta. However, much of this research is limited by small sample sizes, a narrow focus on specific pollutants, and a lack of comprehensive integration of epigenomic and transcriptomic data^12–15^. These limitations underscore the need for more extensive research to elucidate how such epigenetic changes influence immune development and disease susceptibility in offspring.

Our study aims to investigate the mechanisms linking prenatal PM_2.5_ exposure to AD by examining whether prenatal epigenetic alterations are associated with transcriptional programs that reappear across developmental stages. We hypothesize that prenatal PM_2.5_ exposure induces specific epigenetic modifications, primarily hypomethylation of immune-related genes in certain placental immune cell types. This epigenetic alteration could later re-emerge in offspring and adult immune cells, potentially priming them for heightened inflammatory responses and increased AD risk. By integrating placental epigenomic and transcriptomic data, we aim to identify molecular pathways underlying the epigenetic basis of AD, providing insights for preventive and therapeutic strategies targeting early-life environmental exposures.

## Results

### Epigenomic and transcriptomic profiling of placenta samples by prenatal PM_2.5_ exposure and AD in offspring

**Fig. 1:**
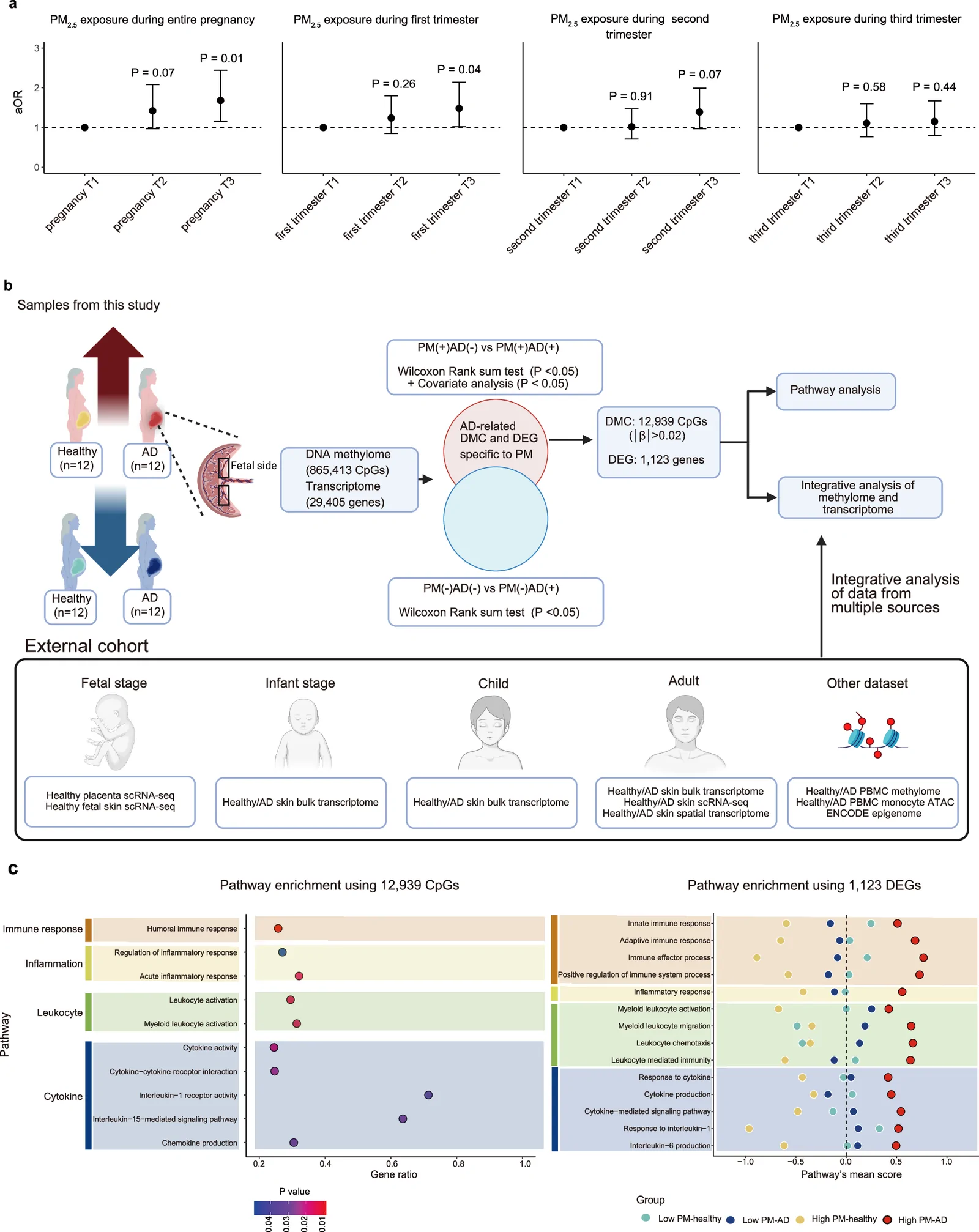
Prenatal PM_2.5_ exposure and associated mechanisms in the risk of development of atopic dermatitis. **a** Adjusted odds ratio (aOR) for incidence of atopic dermatitis (AD) at age 3 in association with PM_2.5_ levels during each gestational period. Pregnancy was divided into three trimesters, as follows: 1–13 weeks (first trimester), 14–27 weeks (second trimester), and 28–40 weeks (third trimester). PM_2.5_ levels were categorized into tertiles (T1–T3) (*n* = 986 children), with T1 being the lowest and T3 being the highest. Models were adjusted for sex, parental allergic history, maternal age, maternal education, maternal body mass index, secondhand smoke exposure during pregnancy and infancy. Error bars show adjusted odds ratios with 95% confidence intervals. *P* values were calculated using two-sided logistic regression. **b** Schematic of the experimental design, analytic pipeline, and external resources. Samples were stratified by PM₂.₅ exposure levels and AD status (*n* = 12 placental samples per group) to identify PM₂.₅-associated epigenetic and transcriptional patterns related to AD. The stage-specific external datasets are listed in the bottom panel; these were used for cross-cohort validation and multi-source integration. Created in BioRender. Yang, D.Y. (https://BioRender.com/1dpbutq). **c** Pathway analysis of differentially methylated CpG sites (DMCs; left) and differentially expressed genes (DEGs; right). The *x* axis shows the gene ratio, defined as the proportion of pathway-related genes relative to the total number of genes in a given pathway (left), and mean pathway scores across groups (right). Pathways with *P* < 0.05 are shown. Statistical significance was determined using over-representation analysis (left) and the Wilcoxon rank-sum test comparing the High PM_2.5_-AD group with other groups (right). All statistical tests were two-sided, and *P* values shown are nominal (unadjusted). Exact P values and source data are provided in the Source Data file.

The primary characteristics of the study population, which included 986 children from the Cohort for Childhood Origin of Asthma and Allergic Diseases (COCOA), are summarized in Supplementary Data 1. The average PM_2.5_ exposure level during pregnancy was 27.47 μg/m³, with an AD incidence of 37.5% among offspring. Logistic regression analysis revealed a significant association between prenatal PM_2.5_ exposure and increased risk of AD at the age of 3 years (entire pregnancy: adjusted odds ratio [aOR] 1.68, 95%CI 1.16–2.44; first trimester: aOR 1.48, 95%CI 1.02–2.14, Fig. 1a). To investigate the effects of prenatal PM_2.5_ exposure on the epigenome and transcriptome and its association with AD, we analyzed CpG methylation and gene expression profiles in placenta and cord blood samples. Placental sampling was specifically conducted on the fetal side rather than the maternal side. These samples were grouped based on PM_2.5_ exposure levels and AD status of offspring (Fig. 1b, see “Methods”). To capture exposure-specific mechanisms, we excluded differentially methylated CpGs (DMCs) and differentially expressed genes (DEGs) overlapping with the low-exposure group, thereby isolating signatures unique to high-exposure AD cases. This filtering strategy was guided by the hypothesis that widespread epigenetic perturbations induced by high PM_2.5_ exposure drive AD pathogenesis, whereas AD under low exposure may arise from genetic predisposition. Using this approach, we identified 12,939 differentially methylated CpGs (DMCs) and 1123 DEGs in the placentae of AD-affected offspring with high PM_2.5_ exposure (Supplementary Data 2). Covariate analysis confirmed that these epigenomic and transcriptomic alterations were independent of confounding factors such as sex, age, and maternal AD status. Pathway enrichment analysis of DMCs and DEGs revealed substantial disruptions in allergy-related pathways (Fig. 1c and Supplementary Fig. 1a). Specifically, placentae from AD offspring exposed to high PM_2.5_ showed pronounced activation of these pathways compared to other groups, consistent with previous reports describing their broad activation following PM exposure in AD development^16^. We designate this subgroup as the placentae of AD offspring with an epigenetic alteration, highlighting a potential mechanism by which PM_2.5_-driven epigenetic modifications may elevate the risk of AD.

### Integrative analysis identifying disrupted epigenetic regulation of *FCER1G* in the placenta and its transcriptional reactivation in AD

**Fig. 2:**
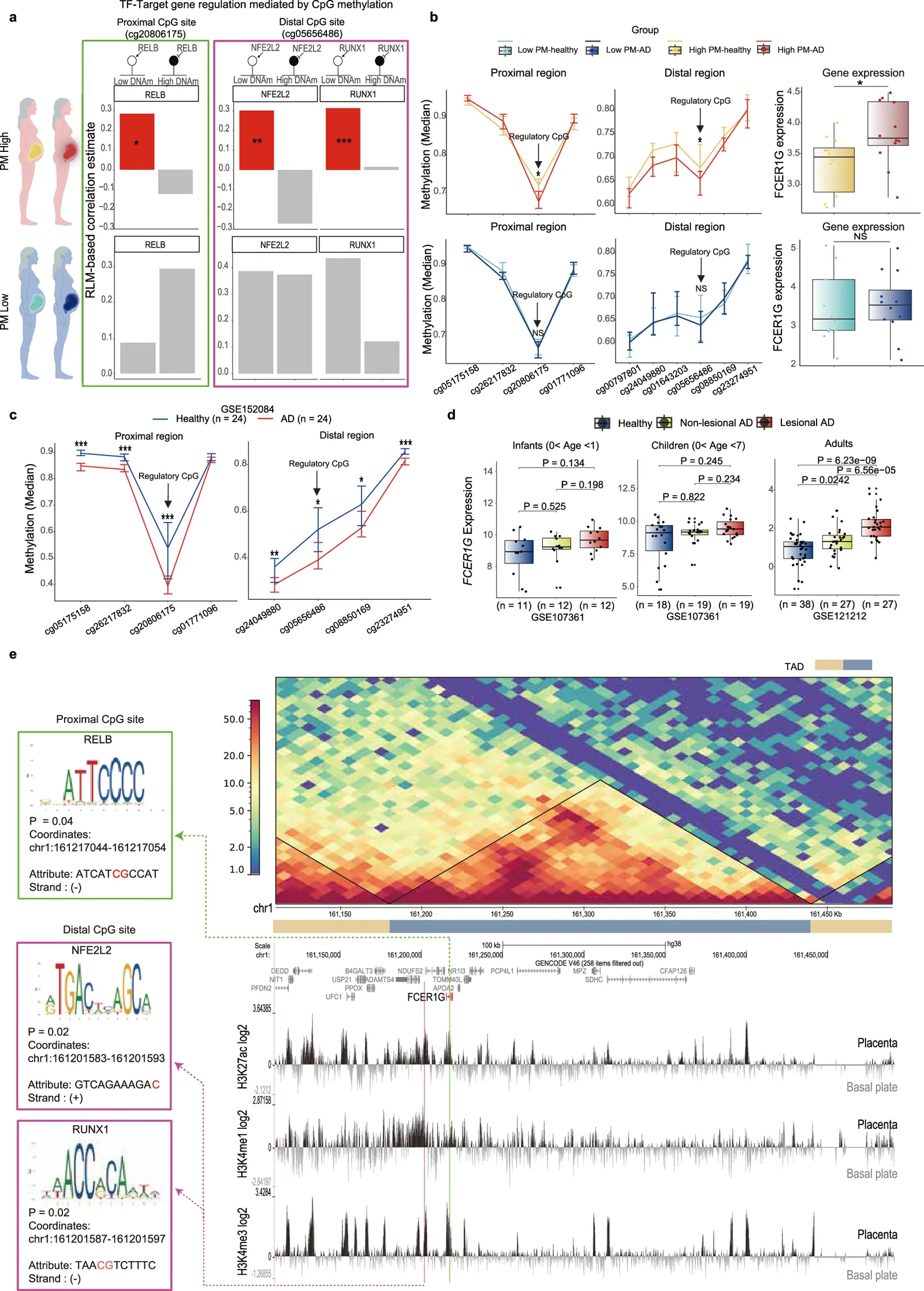
Epigenetic regulation of *FCER1G* in response to PM_2.5_ exposure and its developmental trajectory. **a** Triplet analysis (CpG-transcription factor-target gene) for *FCER1G*, highlighting proximal (cg20806175, +1814 bp from TSS) and distal (cg05656486, −13,639 bp from TSS) CpG sites. Samples were divided into low and high DNA methylation (DNAm) groups based on median DNAm levels. The y-axis shows MethReg robust linear model (RLM) estimates. Created in BioRender. Yang, D.Y. (https://BioRender.com/8h2zitl). **b** Line plots with error bars display median DNAm levels of CpGs located within ±200 bp of the regulatory CpG sites in proximal (left) and distal (middle) regions from placenta samples (*n* = 12 placenta samples per group). The CpG ID (x-axis) is ordered based on chromosomal position. Box plots (right) show *FCER1G* expression levels across four groups. **c** Line plots with error bars represent the median DNAm levels of CpGs located within ±200 bp of the regulatory CpG in proximal (left) and distal (right) regions. **d**, Box plots represent *FCER1G* expression levels across developmental stages, derived from public datasets. Sample sizes and data sources are annotated in (**c**, **d**). **e** Genome browser views of Hi-C and ChIP-seq data. Topologically associating domain (TAD) boundaries from placental Hi-C data are annotated in the bar below the Hi-C contact map. Tracks display ChIP-seq signals for H3K27ac, H3K4me1, and H3K4me3, shown as log_2_-transformed ratios comparing the placenta to the placental basal plate (maternal side). The left panel shows predicted TF binding at each regulatory CpG site. All data were obtained from ENCODE and Roadmap Epigenomics. Error bars indicate median with interquartile range (**b**, **c**). Box plots depict the median (central line), interquartile range (box edges), and whiskers extending to 1.5× IQR (**b**, **d**). Statistical analyses include MethReg RLM for DNAm groups (**a**), Wilcoxon rank-sum test (**b**–**d**), and TFBSTools for transcription factor binding sites (**e**). All statistical tests were two-sided, and *P* values shown are nominal (unadjusted). *P ≤ 0.05, **P ≤ 0.001, ***P ≤ 0.0001, NS not significant. Exact *P* values and source data are provided in the Source Data file.

To identify gene-level perturbations in the placentae of offspring with AD with an epigenetic alteration, we conducted an integrative analysis combining methylome and transcriptome data. Using a triplet analysis framework^17^, we investigated the causal regulatory relationship between transcription factors (TFs) and their target genes, mediated by CpG methylation, which modulates TF binding. Our findings highlight *FCER1G*, a subunit of the IgE receptor, as a gene positively regulated by TFs RELB, NFE2L2, and RUNX1 through hypomethylated CpG sites under high PM_2.5_ exposure (Fig. 2a and Supplementary Fig. 1b, c). These sites, both proximal and distal to *FCER1G*, were identified as potential regulatory elements. In placentae from offspring with AD exposed to high PM_2.5_, proximal and distal CpG sites exhibited significant hypomethylation compared to controls, correlating with elevated *FCER1G* expression (Fig. 2b, top). Notably, this hypomethylation extended to adjacent CpG sites within the distal regulatory region, indicating a broader epigenetic disruption. In contrast, no methylation changes were observed in the low PM_2.5_ exposure group (Fig. 2b, bottom). Across all placental samples, linear regression did not reveal a uniform inverse association between PM2.5 exposure and CpG methylation, consistent with substantial inter-individual variability^14,18^. However, inclusion of an interaction term between PM2.5 exposure and AD status uncovered a significant exposure-dependent hypomethylation effect restricted to individuals who developed AD, concomitant with elevated *FCER1G* expression (Supplementary Fig. 1d). These data support a context-specific epigenetic response to PM2.5 rather than a generalized exposure effect. A parallel analysis of cord blood methylome and transcriptome mirrored these trends but lacked statistical significance in the high PM_2.5_ exposure groups (Supplementary Fig. 1e, f). Intriguingly, the *FCER1G* hypomethylation identified in placentae of offspring with AD and high prenatal PM_2.5_ exposure was recapitulated in peripheral blood samples from children who later developed AD, collected between 0.1 and 5.4 years of age (GEO accession GSE152084) (Fig. 2c). This corresponded with a progressive overexpression of *FCER1G* in AD lesion samples across developmental stages, from infancy to adulthood^19,20^ (Fig. 2d). The regulatory CpG sites, located 13,693 bp upstream and within an intronic region of *FCER1G*, were located within the same topologically associated domain and overlapped with active histone marks (H3K27ac, H3K4me1, and H3K4me3) in placental tissue, but not in the maternal basal plate of the maternal side (Fig. 2e). To assess the potential causal role of these epigenetic modifications, we applied a one-sample Mendelian Randomization (MR) approach using 24 placental samples. This analysis revealed that hypomethylation at cg05656486 and cg20806175 was significantly associated with increased risk of infantile AD, suggesting a direct contribution of these loci to disease susceptibility (Supplementary Fig. 2a, b and Supplementary Data 3). Notably, these associations remained statistically robust after adjusting for PM_2.5_ exposure levels and other key covariates. These findings suggest that high-PM_2.5_-induced hypomethylation enhances *FCER1G* expression by increasing TF binding susceptibility, thereby contributing to AD development via sustained epigenetic alterations.

### *FCER1G*-induced signaling activates allergy-related pathways predominantly in placental macrophages

**Fig. 3:**
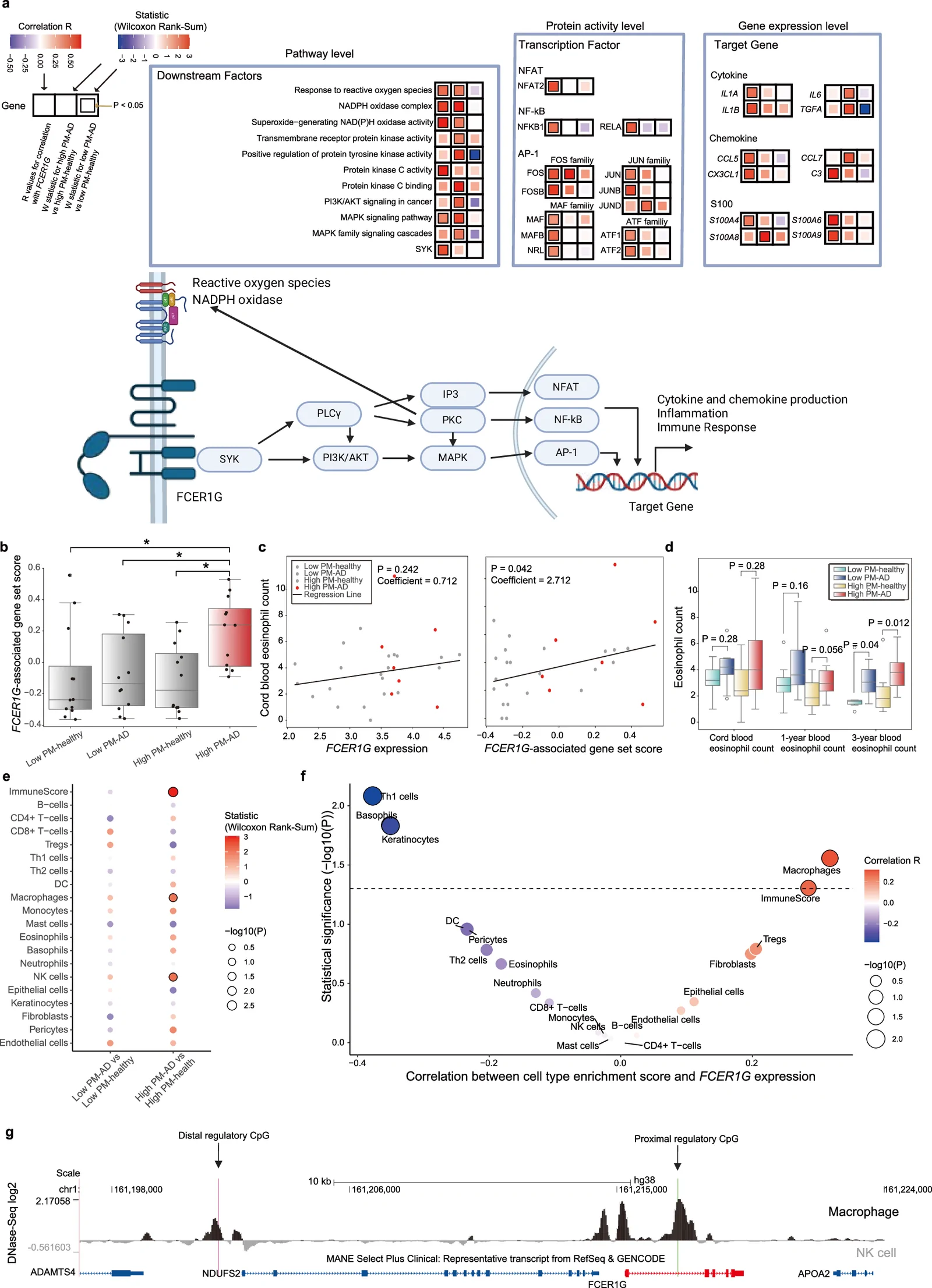
Epigenetic and cellular dynamics of *FCER1G* in PM_2.5_ exposure and atopic dermatitis. **a** Schematic of *FCER1G*-related signaling. Each panel represents an analysis dimension: pathway activation inferred by GSVA, transcription factor activity inferred by VIPER, and target gene expression levels. Within each panel, individual cells display the correlation with *FCER1G* expression and differential analysis results across comparisons: High PM_2.5_-AD vs. Low PM_2.5_-AD, and High PM_2.5_-healthy vs. Low PM_2.5_-healthy (*n* = 12 placental samples per group). Created in BioRender. Yang, D.Y. (https://BioRender.com/pco4gwh). **b** Box plots depict *FCER1G*-associated gene set scores across the four groups (*n* = 12 placental samples per group). **c** Correlation plots illustrate the associations between eosinophil count in cord blood and *FCER1G* expression (left) and *FCER1G*-associated gene set score (right) in the placenta samples. Red dots represent the High PM_2.5_-AD group (*n* = 7), while the other groups are depicted in grey. **d** Eosinophil counts are shown as box plots at different ages: Cord blood (Low PM_2.5_-healthy, *n* = 5; Low PM_2.5_-AD, *n* = 11; High PM_2.5_-healthy, *n* = 10; High PM_2.5_-AD, *n* = 7), 1 year (Low PM_2.5_-healthy, *n* = 5; Low PM_2.5_-AD, *n* = 8; High PM_2.5_-healthy, *n* = 8; High PM_2.5_-AD, *n* = 7), and 3 years (Low PM_2.5_-healthy, *n* = 6; Low PM_2.5_-AD, *n* = 9; High PM_2.5_-healthy, *n* = 10; High PM_2.5_-AD, *n* = 11). **e** Dot plots display immune cell type proportions inferred from the placental transcriptome using the xCell program. Black-outlined dots indicate results with *P* < 0.05. **f** A correlation plot shows the association between *FCER1G* expression and cell type enrichment scores. Dots with black outlines represent correlations with *P* < 0.05. The dashed line along the y-axis marks -log10(0.05). **g** Log_2_-transformed ratios of chromatin accessibility comparing macrophage to NK cell, derived from DNase-seq profiles in ENCODE. Box plots display the median (central line), interquartile range (box edges), and whiskers extending to 1.5× IQR (**b**, **d**). Statistical analyses include Spearman correlation (**a**, **f**), Huber regression (**c**), and the Wilcoxon rank-sum test (**a**–**e**). All statistical tests were two-sided, and *P* values shown are nominal (unadjusted). *P ≤ 0.05. Exact *P* values and source data are provided in the Source Data file.

We examined the downstream signaling alterations triggered by *FCER1G* overexpression. Pathway analysis revealed that elevated *FCER1G* expression correlated positively with the activation of PI3K/PIP/AKT, protein kinase C, NADPH oxidase, and MAPK signaling, as well as with SYK expression (Fig. 3a). Activation of these pathways led to enhanced activity of the transcription factors AP-1 and NFAT, driving the expression of genes linked to cytokine and chemokine production, inflammation, and immune responses associated with allergic outcomes. Moreover, these positive correlations were also activated specifically in placental samples with high PM_2.5_ exposure having the epigenetic disruption but not in the low PM_2.5_ group. We further identified a distinct *FCER1G* downstream signaling gene signature, which was notably upregulated in placentae from AD offspring with an epigenetic alteration (Fig. 3b). This signature positively correlated with eosinophil counts in cord blood (Fig. 3c), and the eosinophil count was significantly elevated at 3 years of age (Fig. 3d), implicating them as markers of allergic inflammation^21^. Additionally, our data revealed activation of reactive oxygen species (ROS) and NADPH oxidase pathways (Fig. 3a), suggesting potential signaling perturbations in immune cells such as macrophages and monocytes. To determine cell-type specificity, digital cytometry inference^22,23^ of our transcriptome data indicated increased macrophage and NK cell enrichment score in the placentae of offspring with AD exposed to high PM_2.5_, with macrophages showing a significant association with *FCER1G* expression and signaling activation (Fig. 3e, f). This positive correlation between *FCER1G* expression and macrophage enrichment was consistently observed across developmental stages—including infants, children, and adults—using independent skin transcriptome datasets from healthy controls and AD patients^19,20^ (Supplementary Fig. 3a–f). Additionally, epigenomic analysis confirmed high regulatory potential specific to macrophages, as loci of epigenetic disruption, both proximal and distal to *FCER1G*, exhibited the strongest DNase-seq signals in macrophages, compared to other immune cells (Fig. 3g and Supplementary Fig. 3g). Furthermore, our analysis of public ATAC-seq data (GEO accession GSE255396) demonstrated increased chromatin accessibility at these *FCER1G* regulatory regions in PBMC monocytes from AD patients (Supplementary Fig. 3h). Although these external datasets lack prenatal PM exposure information, the recurrence of *FCER1G* hypomethylation and chromatin accessibility at key regulatory elements underscores the likelihood of reactivation of early-life epigenetic programming in AD. Supporting our findings, Liang et al. ^24^ reported hypomethylation of the *FCER1G* promoter in peripheral monocytes from adult AD patients, which was inversely correlated with gene expression. As monocytes are precursors of tissue-resident macrophages, these results suggest that epigenetic alterations in circulating myeloid cells may be transmitted to skin macrophages. This supports the relevance of *FCER1G* dysregulation in dermal macrophage-mediated inflammation and reinforces its role in triggering allergy-related pathways, particularly in placental macrophages of offspring with AD.

### *FCER1G* overexpression in placental Hofbauer cells parallels its upregulation in AD-associated M2 macrophages

**Fig. 4:**
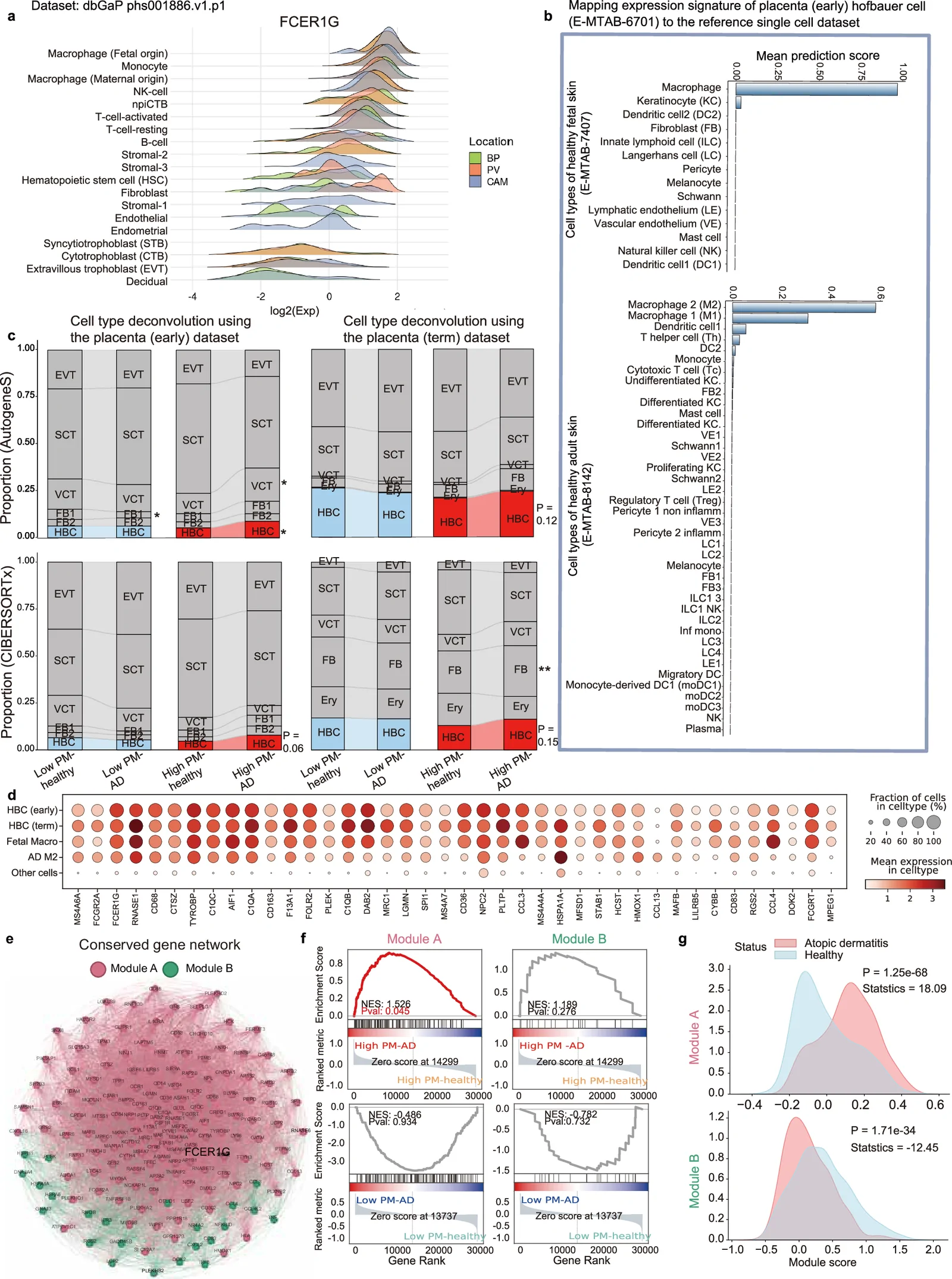
*FCER1G* expression and fetal macrophage dynamics in the placenta and atopic dermatitis. **a** Density plots show log_2_-transformed *FCER1G* expression across fetal macrophage populations (*n* = 79,906 cells) in the placenta, categorized by anatomical location: basal plate BP, placental villous tissue PV, and chorioamniotic membrane CAM. Data sourced from http://placenta.grid.wayne.edu/. **b** Bar plots display the mean prediction score of early placental Hofbauer cell states, inferred using healthy fetal skin (top) and healthy adult skin (bottom) as reference datasets. The prediction scores were obtained using the TransferAnchors function in Seurat. **c** Proportions of fetal cell types (EVT extravillous trophoblast, SCT syncytiotrophoblast, VCT villous cytotrophoblast, FB fibroblast, Ery erythrocyte/blast, HB Hofbauer cell) in the placenta inferred from the bulk transcriptome using AutogeneS (top) and CIBERSORTx (bottom). Left and right panels show estimates based on early- and term-stage placenta data, respectively, used to generate signature matrices. Asterisks indicate significant differences (*P ≤ 0.05, **P ≤ 0.001). **d** Dot plots illustrate the mean expression of the top 40 conserved genes (ranked by adjusted *P* values) in analogous cell types (Hofbauer cells, fetal skin macrophages, and adult M2 macrophages) compared to non-analogous cells. The list of conserved genes is provided in Supplementary Data 6. **e** Network visualization of conserved genes grouped into two modules, generated using Gephi. *FCER1G* is included in Module A. **f** Gene set enrichment analysis of bulk placenta transcriptomes stratified by PM levels (*n* = 12 placental samples per group), using gene sets corresponding to Modules A and B. **g** Density plots represent module scores for Module A (top) and Module B (bottom), calculated using scanpy.tl.score_genes, in adult healthy M2 macrophages (five samples, 1424 cells) and AD M2 macrophages (four samples, 940 cells). Statistical analyses included generalized linear modeling with a quasibinomial distribution for proportion comparisons, likelihood ratio tests for deconvolution estimates (**c**), negative binomial tests in FindConservedMarkers (**d**), gene set enrichment analysis (**f**), and Wilcoxon rank-sum tests (**g**). All statistical tests were two-sided, and *P* values shown are nominal (unadjusted). Exact *P* values and source data are provided in the Source Data file.

Prenatal exposure to PM_2.5_ was conceptualized as an early-life environmental stressor capable of inducing epigenetic programming in developing immune cells. To delineate the downstream cellular consequences of this programming, we traced the reactivation of *FCER1G*-driven macrophage networks across developmental stages, testing whether these programs re-emerge in AD. This developmental recapitulation suggests a mechanistic continuum linking prenatal environmental exposure to postnatal disease phenotypes, even in the absence of direct postnatal exposure data. To define the cellular specificity of *FCER1G* within the placental immune niche, we analyzed single-cell transcriptomic data from consortium-based placental atlas datasets^25^. This analysis confirmed that *FCER1G* was most prominently expressed in Hofbauer cells, the fetal macrophages located in the villous stroma at all gestational stages (Supplementary Fig. 4a). Independent single-cell data from 25 placental samples^26^ also showed consistent *FCER1G* overexpression in fetal-origin macrophages across different placental regions, including basal plate (BP), placental villous tissue (PV), and chorioamniotic membrane (CAM) (Fig. 4a). Additionally, we integrated scRNA-seq data from multiple developmental stages across placenta, fetal, and adult skin samples^25,27–29^ (Supplementary Fig. 4b). Hofbauer cells were closer to fetal skin macrophages compared to decidual macrophages in the UMAP space (Supplementary Fig. 4c). Comparative gene signature analysis of early and term placental cells against single-cell transcriptional profiles from fetal and adult skin^25,27–29^ identified phenotypic similarities between Hofbauer cells, fetal skin macrophages, and M2 macrophages (Fig. 4b and Supplementary Fig. 4d). Conversely, these parallels were absent in other placental or skin-derived cell types (Supplementary Fig. 5). Adult M2 macrophages have been reported to be more enriched in the skin of patients with AD than in healthy controls^29^. To investigate the potential role of Hofbauer cells as M2 progenitors in AD, we conducted digital cytometry analysis using AutogeneS^22^ and CIBERSORTx^23^ on bulk transcriptomic profiles from our placental samples. Cell-type-specific gene sets were derived from early and term placental single-cell transcriptomes^25,27^. This analysis revealed a significantly higher abundance of Hofbauer cells in placental samples from offspring with AD exposed to elevated PM_2.5_ levels (Fig. 4c), which coincided with increased *FCER1G* expression (Fig. 2a). At the gene regulatory level, co-expression network analysis revealed a conserved gene module, including *FCER1G*, that was overexpressed across developmental stages in placental macrophages, fetal skin macrophages, and adult M2 macrophages (Fig. 4d). Notably, several genes within this conserved module, including CD163, F13A1, and MRC1, have been experimentally validated to be overexpressed in AD skin lesions through immunohistochemistry^29,30^. This module, designated “module A,“ was selectively activated in the placentae of offspring with AD displaying an epigenetic alteration, but remained inactive in those without such epigenetic alterations (Fig. 4e, f). Furthermore, in an independent single-cell transcriptome cohort of adult AD and healthy samples^29^, module A genes were significantly upregulated in M2 macrophages from skin samples with AD compared to healthy controls, a trend not observed in module B genes (Fig. 4g). Integrative single-cell analyses across developmental stages confirmed sustained activation of module A in Hofbauer cells, fetal macrophages, and M2 macrophages of adult skin with AD but not in healthy controls (Supplementary Fig. 6a). This consistent activation pattern suggests that *FCER1G*-overexpressing Hofbauer cells in the placenta may re-emerge as M2 macrophages in adult skin with AD.

### Trajectory of *FCER1G* expression in M2 macrophages is distinct in adults with AD

**Fig. 5:**
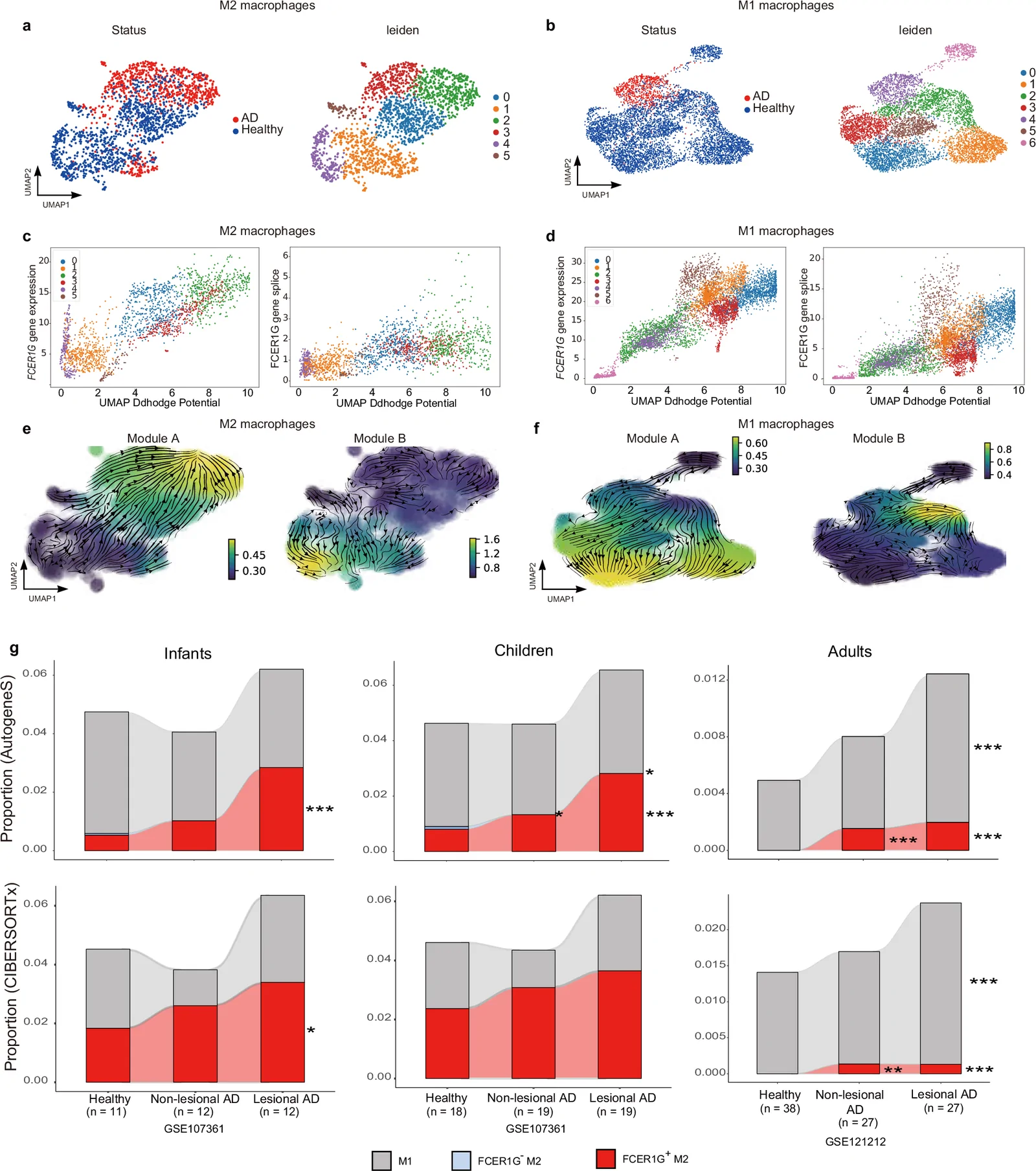
Single-cell trajectory and velocity analyses reveal *FCER1G* dynamics in macrophage subsets during the progression of atopic dermatitis. **a** Uniform manifold approximation and projection (UMAP) plots of adult M2 macrophages, labeled by AD status (left) and cell type clustering (right). The analysis includes adult healthy M2 (five samples, 1200 cells) and AD M2 (four samples, 844 cells) obtained from E-MTAB-8142. **b** UMAP plots of adult M1 macrophages, labeled by AD status (left) and cell type clustering (right). The dataset includes adult healthy M1 (five samples, 5781 cells) and AD M1 (four samples, 875 cells). **c** *FCER1G* expression (left) and splicing dynamics (right) along pseudotime, represented as ddHodge potential in M2 macrophages with Leiden cluster annotation. Leiden clusters 2 and 3 are associated with AD status. **d** *FCER1G* expression (left) and splicing dynamics (right) along pseudotime in M1 macrophages with Leiden cluster annotation. Leiden cluster 4 is associated with AD status. **e** RNA velocity analysis for module A (left) and module B (right) projected onto UMAP in M2 macrophages. **f** RNA velocity analysis for module A (left) and module B (right) projected onto UMAP in M1 macrophages. Color represents module scores, calculated using scanpy.tl.score_genes. **g** Proportion of M1, *FCER1G*^-^ M2, and *FCER1G*^+^ M2 inferred from bulk transcriptome data of infants (GSE107361), children (GSE107361), and adults (GSE121212) using AutogeneS (top) and CIBERSORTx (bottom). scRNA-seq data (E-MTAB-8142) were used to generate the signature matrix for each algorithm. Proportions were compared between lesional AD and healthy groups, as well as between non-lesional AD and healthy groups using generalized linear modeling with a quasibinomial distribution. Asterisks indicate significant differences. Statistical analysis include likelihood ratio test (**g**). All statistical tests were two-sided, and *P* values shown are nominal (unadjusted). *P ≤ 0.05, **P ≤ 0.001, ***P ≤ 0.0001. Exact *P* values and source data are provided in the Source Data file.

To examine the dynamics of *FCER1G* expression in macrophages during the progression of AD, we analyzed macrophage populations from single-cell transcriptomes of adult skin samples with AD and healthy controls^29^ using trajectory analysis. Macrophages were classified into M2 (Fig. 5a) and M1 lineages (Fig. 5b), derived from both AD and healthy samples, and distinct cell states were inferred. *FCER1G* expression was tracked along pseudotime by assessing both expression levels and splicing patterns (Fig. 5c, d). In M2 macrophages, *FCER1G* expression gradually increased, particularly in cell states 2 and 3, which predominantly originated from patients with AD (Fig. 5a, c). This trend remained consistent when analyzed using splicing patterns for enhanced inference accuracy^31,32^. In contrast, M1 macrophages in AD samples did not show *FCER1G* overexpression, with higher expression observed in healthy controls (Fig. 5b, d). Analysis of *FCER1G*-associated gene module A, previously described in Fig. 4e, reflected this overexpression pattern in M2 macrophages, whereas module B, which was not associated with *FCER1G*, followed an opposing trajectory (Fig. 5e and Supplementary Fig. 6b). For M1 macrophages, neither module A nor module B demonstrated convergence on AD-specific cell states (Fig. 5f). Further, discriminative genes of *FCER1G*-overexpressing (*FCER1G*^+^) M2 macrophages were used for digital cytometry analysis, as outlined in Fig. 4c, to estimate cell-type proportions in bulk transcriptomes of AD and healthy skin across developmental stages (Fig. 5g, see “Methods”). The proportion of *FCER1G*^+^ M2 macrophages progressively increased from healthy to AD lesional samples, irrespective of developmental stage. In contrast, *FCER1G*^−^ M2 macrophages and M1 macrophages did not exhibit significant increases across developmental stages. To assess the association with clinical severity, we stratified lesional AD patients by SCORAD, a validated index of AD severity. *FCER1G*⁺ M2 macrophage enrichment scores were markedly elevated in patients with severe AD compared to those with mild to moderate disease (Supplementary Fig. 6c), establishing a positive correlation between *FCER1G*⁺ M2 macrophage abundance and disease severity. These findings suggest that the re-emergence of *FCER1G*^+^ M2 macrophages contributes to the development of AD at both cellular and developmental trajectory levels.

### Re-emerging *FCER1G*-centered signaling in M2 macrophages contributes to AD via activation of the ROS pathway

**Fig. 6:**
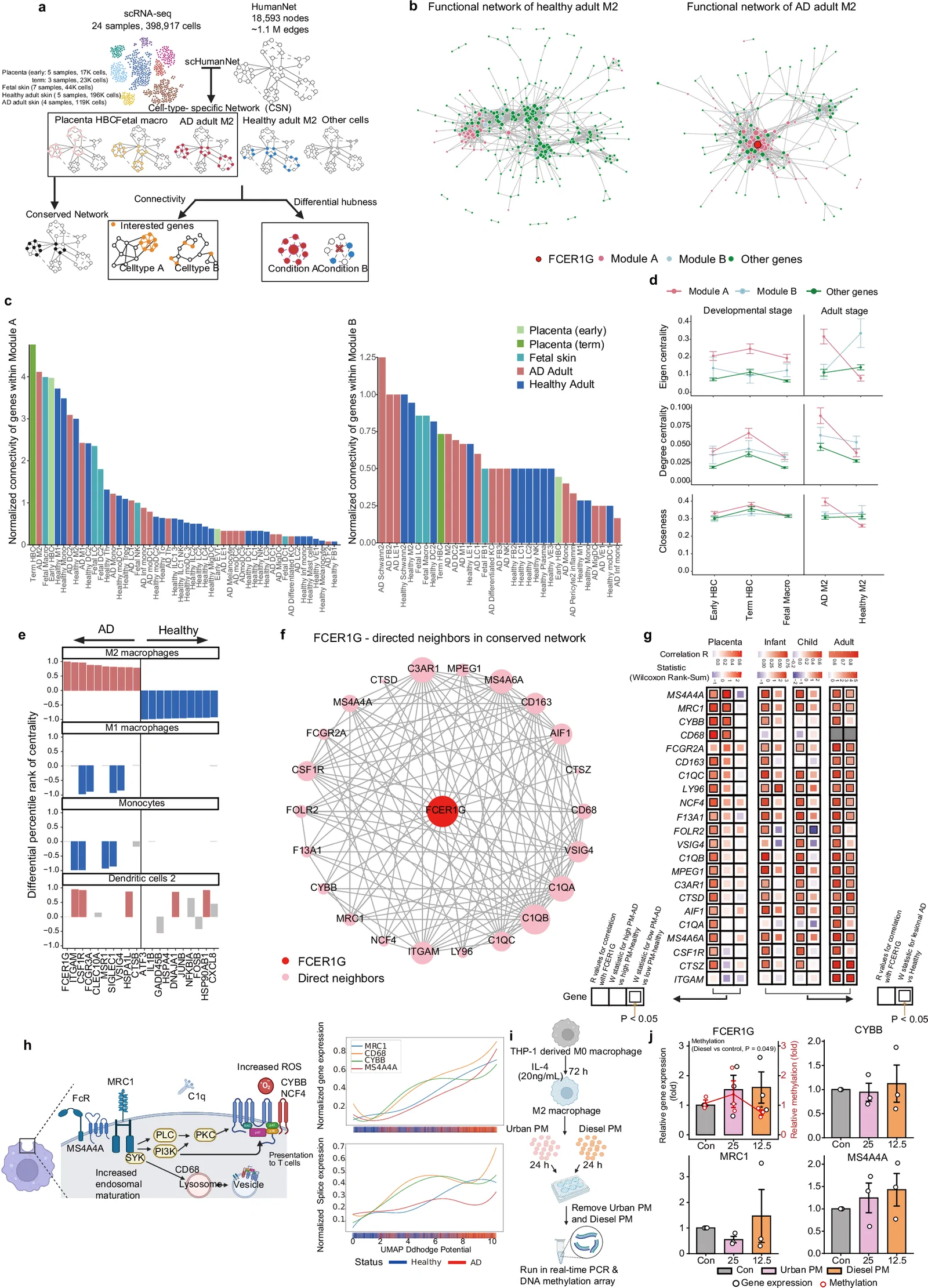
Network-based analysis identifies *FCER1G*-centered interactions across developmental stages and the progression of atopic dermatitis. **a** Schematic of the network analysis pipeline. A cell-type-specific network (CSN) was constructed using scRNA-seq data across developmental stages. See Methods for details. Created in BioRender. Yang, D.Y. (https://BioRender.com/o2pzq4w). **b** Network visualization of healthy adult M2 macrophages (left) and AD adult M2 macrophages (right). Nodes represent genes, with colors indicating gene or functional modules. Node size reflects the number of interactions. **c** Normalized within-group connectivity scores for Module A (left) and Module B (right) in the placenta, fetal skin, and adult cell-type-specific networks. **d** Network centrality analysis of Modules A and B across developmental stages, including Hofbauer cells (early: *n* = 1966 cells; term: *n* = 1699 cells), fetal skin macrophages (*n* = 3,085 cells), and adult M2 macrophages (healthy: *n* = 1214 cells; AD: *n* = 782 cells). **e** Differential percentile rank of centrality scores for cell types. The top 10 genes in Module A, ranked by *P* values, are shown for each status. **f** Network visualization of genes directly connected to *FCER1G* within the conserved network (Supplementary Fig. 8d). Node size corresponds to the number of interactions. **g** Correlation and expression analysis of *FCER1G*-associated genes across developmental stages using bulk transcriptome data. **h** Schematic of biological interactions between *FCER1G* and neighboring genes (left). Expression (top right) and splicing dynamics (bottom right) of PM_2.5_-high AD–upregulated genes (*MS4A4A*, *MRC1*, *CD68*, *CYBB*) are shown along pseudotime (ddHodge potential) in M2 macrophages. Created in BioRender. Yang, D.Y. (https://BioRender.com/t7h2tia). **i** Schematic of the experimental design to assess PM_2.5_ effects on M2 macrophages. Created in BioRender. Yang, D.Y. (https://BioRender.com/z48ggo1). **j** Relative expression of *FCER1G*, *CYBB*, *MRC1*, and *MS4A4A* under urban PM (25 µg/mL) and diesel PM (12.5 µg/mL) conditions in M2 macrophages; control (Con) cells were not exposed to PM. The relative methylation level of a distal regulatory CpG associated with *FCER1G* is indicated by a red line with error bars. Gene expression and methylation data represent *n* = 3 biological replicates per condition. Error bars represent mean ± s.e.m (**d**, **j**). Statistical analyses include FindDiffHub from scHumanNet (**e**), Pearson correlation (**g**), and the Wilcoxon rank-sum test (**g**, **j**). All statistical tests were two-sided, and *P* values shown are nominal (unadjusted). *P ≤ 0.05, **P ≤ 0.001, ***P ≤ 0.0001. Exact *P* values and source data are provided in the Source Data file.

To elucidate the role of *FCER1G*^+^ M2 macrophages during fetal development and their implications in AD, we conducted a comprehensive single-cell transcriptomics analysis spanning developmental stages, from the placenta to adult skin. Our dataset integrated 398,917 cells from 24 samples across placental, fetal skin, and adult skin tissues of healthy controls and patients with AD^25,27–29^ (Fig. 6a and Supplementary Fig. 7). Cell-type-specific functional networks were derived using scHumanNet^33^, an algorithm that constructs context-specific, highly interactive gene networks by integrating diverse biological interactions, including genetic associations and protein-level signaling. A conserved co-expression module, termed module A, prominently included *FCER1G* (Fig. 6b) and demonstrated sustained connectivity in macrophages across developmental stages, particularly from placental macrophages to adult M2 macrophages in patients with AD (Fig. 6c and Supplementary Fig. 8). Comparative network analyses revealed significantly higher connectivity of module A in M2 macrophages of adults with AD compared to healthy controls and other cell types (Fig. 6c, left). Conversely, module B, which exhibited a similar co-expression profile but excluded *FCER1G*, displayed only moderate interconnections (Fig. 6c, right). Centrality and closeness metrics confirmed the sustained and progressively amplified connectivity of module A in AD-related macrophages, with the highest degree of network integration observed in M2 macrophages from adult skin with AD, contrasting sharply with lower connectivity in their healthy counterparts (Fig. 6d). These findings demonstrate that the *FCER1G*-enriched module, which is conserved across developmental stages, undergoes reactivation and heightened functionality in M2 macrophages of adult skin with AD.

To further investigate the functional significance of *FCER1G* in M2 macrophages of adult patients with AD, we assessed its role as a central hub in cell-type-specific networks. AD-specific centrality scores for all genes were calculated by comparing gene interactions between patients with AD and healthy controls using single-cell transcriptomes (see “Methods”). *FCER1G* emerged as the top-ranked core gene specifically in M2 macrophages, outperforming its centrality in other cell types (Fig. 6e and Supplementary Fig. 9). To further explore its functional interactions, we constructed a *FCER1G*-centered directed network using its neighboring genes from conserved macrophage-specific networks across placental, fetal, and adult AD M2 macrophages (Fig. 6f). Directed neighbors of *FCER1G* displayed significantly positive expression correlations with *FCER1G* and were consistently overexpressed in AD across developmental stages, as confirmed by bulk transcriptome analysis (Fig. 6g). Gene set scoring revealed the specific activation of these directed neighbor genes in early macrophage developmental stages and adult AD-associated M2 macrophages (Supplementary Fig. 10a-d). Among the genes directly interacting with *FCER1G*, *CD68*, *CYBB*, *MRC1*, and *MS4A4A* were identified as key players involved in NADPH oxidase-mediated ROS signaling, a pathway implicated in macrophage-driven inflammatory responses and increased risk of AD^34–36^ (Fig. 6h, left). Consistently, elevated expression of *FCER1G*, *CYBB*, and *MS4A4A* has been reported in tape-stripped atopic dermatitis lesional skin^37^. Single-cell trajectory analyses further demonstrated a progressive upregulation of these genes in M2 macrophages transitioning from healthy to AD states, confirmed by both gene expression and splicing-based status inference analyses (Fig. 6h, right). At the tissue level, gene set enrichment analysis demonstrated a marked elevation of *FCER1G* module scores in lesional AD skin relative to paired non-lesional samples (Supplementary Fig. 10e–j). Concordantly, NADPH oxidase–mediated ROS signaling exhibited a parallel activation trajectory. Correlation analysis across individual samples confirmed a strong positive association between *FCER1G* module activity and ROS signaling across all developmental stages. This cellular signaling cascade could be imprinted by early-stage PM exposure. To validate this, we performed in vitro experiments assessing M2 macrophage transcriptomic and epigenomic dynamics under urban and diesel PM_2.5_ exposure conditions. In THP-1-derived M2 cells, *FCER1G* mRNA expression showed an increasing trend under both PM_2.5_ conditions (Fig. 6i, j). In parallel, DNA methylome profiling revealed that PM exposure induced hypomethylation at the same distal *FCER1G* regulatory regions identified in high PM-exposed placentae, coinciding with *FCER1G* transcriptional activation. Additionally, *FCER1G*-associated signaling molecules, such as *CYBB*, *MRC1*, and *MS4A4A*, were significantly overexpressed under diesel PM_2.5_ exposure. Experimental evidence from PM_10_ exposure in an OVA-induced atopic dermatitis mouse model further supports these in vitro findings, demonstrating significantly increased *FCER1G* expression in the skin of PM_10_-exposed mice^38^. Collectively, these results underscore *FCER1G* as a critical regulator within M2 macrophages, driving aberrant ROS signaling and promoting AD progression across developmental stages.

### Spatial enrichment of *FCER1G*^+^ M2 macrophages aligns with ROS signaling and Th2 chemokine activation in AD lesions

**Fig. 7:**
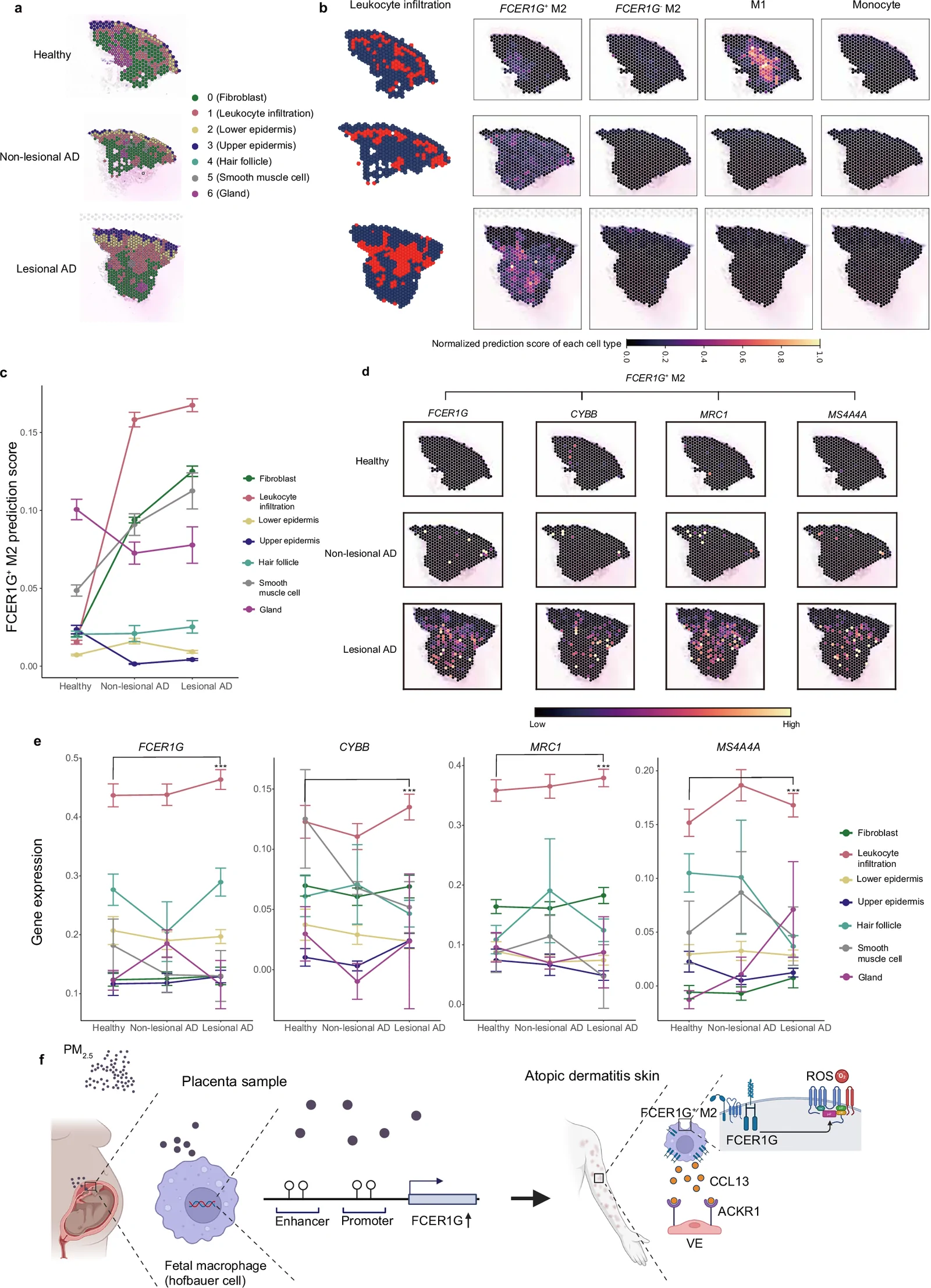
Spatial transcriptome analysis reveals the abundance of *FCER1G*^+^ M2 macrophages and the enrichment of directed neighboring genes in atopic dermatitis. **a** Clustering of spatial transcriptomics spots in healthy, non-lesional, and lesional atopic dermatitis (AD) skin sections (HE_5, AD_6_NL, AD_6_LS). **b** The leukocyte infiltrated area is indicated in red (left). Normalized prediction scores of each cell type (*FCER1G*^+^ M2, *FCER1G*^-^ M2, M1 macrophages, Monocytes) were predicted using cell2location across healthy, non-lesional, and lesional AD sections. **c** Abundance of *FCER1G*^+^ M2 prediction scores across each cluster in all samples (healthy, *n* = 6; Non-lesional AD, *n* = 5; Lesional AD, *n* = 7). **d** Gene expression of *FCER1G*, *CYBB*, *MRC1*, and *MS4A4A* in *FCER1G*^+^ M2 macrophages across healthy, non-lesional, and lesional AD sections. **e** Expression of *FCER1G*, *CYBB*, *MRC1*, and *MS4A4A* across each cluster in all samples (healthy, *n* = 6; Non-lesional AD, *n* = 5; Lesional AD, *n* = 7). The comparison was performed between healthy and lesional AD groups. **f** Schematic diagram represents the pathway from placental particulate matter exposure to M2 macrophage in AD skin. Created in BioRender. Yang, D.Y. (https://BioRender.com/ldaljle). Error bars indicate the mean ± s.e.m (**c**, **e**). Statistical analyses include two-sided Wilcoxon rank-sum test (**e**). *P* values shown are nominal (unadjusted). *P  ≤  0.05, **P  ≤  0.001, ***P  ≤  0.0001. Exact *P* values and source data are provided in the Source Data file.

We analyzed spatial transcriptomics data from healthy, non-lesional AD, and lesional AD skin samples to investigate the spatial dynamics of ROS signaling in AD^39^ (Fig. 7a). Histopathological analysis confirmed a progressive increase in leukocyte infiltration from healthy to AD lesion tissues, as assessed through hematoxylin and eosin staining and validated using specific gene markers (Supplementary Fig. 11a). These findings are consistent with previous studies^39^. Using spatially resolved cell enrichment analysis based on gene signatures derived from scRNA-seq data of adult skin with AD^29^ (Supplementary Fig. 11b), we observed that annotated cell types exhibited spatial localization patterns corresponding to histopathological features. Keratinocytes were confined to the epidermis, while fibroblasts aligned with fibroblast-associated histopathological features in all samples. Notably, *FCER1G*^+^ M2 macrophages showed a distinct enrichment pattern, increasing in density from healthy to AD lesions and aligning with areas of intense leukocyte infiltration. This trend was not observed in *FCER1G*^−^ M2 macrophages or M1 macrophages (Fig. 7b). Further regional enrichment analysis across individual samples demonstrated that *FCER1G*^+^ M2 macrophages were significantly concentrated in AD lesions, rather than in healthy skin, reinforcing their specific association with localized inflammatory microenvironments (Fig. 7c). Furthermore, key ROS signaling mediators—*CYBB*, *MRC1*, and *MS4A4A*—previously identified as direct targets of *FCER1G*^+^ M2 macrophages (Fig. 6h), were significantly enriched in lesional AD skin, colocalizing spatially with *FCER1G*^+^ M2 macrophages (Fig. 7d). Notably, *MS4A4A* was most enriched in non-lesional AD skin. This enrichment was absent in *FCER1G*^−^ M2 macrophages, M1 macrophages, and monocytes (Supplementary Fig. 11c). Further histopathological analysis confirmed a spatially coordinated increase in *FCER1G*^+^ M2 macrophages and ROS signaling factors across different skin samples, demonstrating a consistent enrichment pattern from healthy to lesional AD skin (Fig. 7e). The selective enrichment of the *FCER1G*⁺ M2 macrophage gene signature in AD skin was further validated using the largest available AD skin single-cell consortium dataset, comprising 710,704 cells from 91 AD patients and 62 healthy controls^40^. This independent dataset demonstrated a stepwise increase in *FCER1G*⁺ M2 macrophages from healthy to lesional AD skin (Supplementary Fig. 12). To explore their role in type 2 inflammation, we assessed the expression of Th2-associated chemokines. *CCL13*, previously implicated in AD severity^41,42^, was progressively upregulated in *FCER1G* + M2 macrophages along the healthy-to-lesional axis (Supplementary Fig. 13). This expression trajectory aligns with SCORAD severity correlations reported in prior studies^42,43^. Spatial co-localization analysis revealed that this chemokine was enriched in *FCER1G* + M2 macrophages and colocalized with their receptor, *ACKR1*, which was predominantly expressed in VE3 cells, a vascular endothelial subset. *ACKR1* expression in VE3 cells also increased progressively with disease severity. These results establish a spatially coordinated interaction between *FCER1G* + M2 macrophages and VE cells via the *CCL13*–*ACKR1* axis, suggesting a mechanistic role in ROS-driven Th2 inflammation during AD progression. Collectively, integrative single-cell and spatial transcriptomic analyses together with histopathological evidence support a model in which prenatal PM_2.5_ exposure induces epigenetic alteration in placental macrophages that is associated with reactivation of *FCER1G*⁺ M2 macrophage programs in AD skin, driving ROS-mediated signaling and Th2 inflammation during AD progression, as summarized in the proposed causal pathway (Fig. 7f).

## Discussion

This study represents one of the largest investigations into the association between prenatal PM_2.5_ exposure, particularly in the first trimester, and the increased risk of AD in preschool-aged children from the COCOA birth cohort^44^. We provide the first detailed molecular insight into an epigenetic alteration resulting from environmental exposure and its maintenance in the development of AD. Specifically, we identified *FCER1G*, a subunit of the IgE receptor, as a central molecular target of epigenetic alteration, characterized by hypomethylation and overexpression in the placentae of offspring with AD and driven by the transcription factor activity of NFE2L2 and RUNX1. This epigenetic alteration triggers a downstream signaling cascade that activates allergy-related genes, which we systematically confirmed, alongside a positive association with eosinophil counts in cord blood. To further elucidate the cellular context, we identified M2 macrophages enriched with *FCER1G* overexpression within the placentae of offspring with AD. Integrative analysis across multiple cohorts of AD development, including our own, revealed reactivation of *FCER1G*-associated transcriptional and network programs in *FCER1G*⁺ M2 macrophages. Notably, genes directly interacting with *FCER1G* within the M2 macrophage-specific functional network, including *CD68*, *CYBB*, *MRC1*, and *MS4A4A*, were associated with NADPH oxidase-mediated ROS signaling, a pathway known to drive macrophage-dependent inflammatory responses and increase the risk of AD^34–36^. These ROS signaling factors exhibited cellular trajectory dynamics associated with *FCER1G*^+^ M2 macrophages, and in vitro experiments confirmed that their overexpression could be induced under high PM_2.5_ exposure in M2 macrophages. Our findings underscore the potential role of epigenetically modified *FCER1G*^+^ M2 macrophages in immune priming during early fetal development, contributing to an increased risk of AD in offspring via ROS activation. Beyond ROS activation, spatial transcriptomics revealed that *FCER1G*⁺ M2 macrophages progressively upregulated the Th2-associated chemokine *CCL13*, which colocalized with *ACKR1*-expressing VE3 endothelial cells, establishing a localized immunological axis within AD lesions. Together, these findings highlight *FCER1G*⁺ M2 macrophages as a critical cellular population linking prenatal PM_2.5_ exposure to AD pathogenesis through sustained epigenetic dysregulation, ROS-mediated signaling, and *CCL13*–*ACKR1*-mediated Th2 inflammation.

Previous studies indicate inconsistent associations between prenatal air pollution exposure and childhood eczema, with unclear underlying mechanisms^8^. Evidence suggests that exposure to air pollution in the first trimester may increase the risk of AD^45–47^, aligning with our previous finding that increased exposure to PM_2.5_ in the first trimester correlates with a heightened risk of developing AD at age 3^7^. Epigenetic changes during critical prenatal periods, when fetal skin and immune cells develop, may mediate these associations^48,49^. Despite growing interest, only a limited number of studies have identified specific molecular mechanisms or targets. We propose FCER1G as a targeted molecular candidate, potentially elucidating the mechanism behind epigenetic modulation in AD. Previous research^24^ highlighted the association of *FCER1G* demethylation in the promoter region with increased expression and development of AD in adults, specifically in monocytes. However, this study was limited to promoter profiling and did not explore the mechanistic details of *FCER1G* regulation or its enhancer activity within cellular contexts. In contrast, our findings expand this knowledge by detailing *FCER1G* enhancer regulation and providing specific epigenetic insights within defined cellular settings, thus identifying a concrete mechanism of its dysregulation in AD. Further supporting our conclusions, recent findings^29^ demonstrated the re-emergence of fetal-like macrophage characteristics in adult M2 macrophages in AD, marked by interactions with vascular endothelial cells (VE3) mediated through the *CXCL8*–*ACKR1* axis. Our study corroborates and extends these findings by demonstrating that this developmental recapitulation occurs across life stages, including fetal, childhood, and adult phases, characterized by *FCER1G* overexpression. This overexpression aligns with heightened inflammatory partner protein levels in both cord blood and peripheral blood of offspring with AD, further validated in eosinophils within our cohort. This cross-sectional analysis offers a deeper, mechanistic understanding of the role of *FCER1G* in the pathogenesis of AD.

The re-emergence of *FCER1G*^+^ M2 macrophages in AD appears to be influenced by prenatal environmental exposures, particularly PM_2.5_, which can induce epigenetic changes in the placenta that affect immune function later in life^50,51^. PM_2.5_-induced hypomethylation of *FCER1G* leads to increased expression of IgE receptors on antigen-presenting cells, priming hypersensitivity responses central to AD^10,52^. This epigenetic alteration in placental macrophages, known as Hofbauer cells, may be sustained and reactivate in adult AD, promoting *FCER1G* expression in M2 macrophages linked to allergic pathways^24,53^. Furthermore, elevated eosinophil activity, correlating with *FCER1G* expression, suggests that *FCER1G* significantly contributes to the risk of AD by enhancing allergic inflammation in AD lesions^54,55^.

Our study has several limitations that should be acknowledged, along with compensatory strategies implemented to address them. First, to estimate individual-level exposure to ambient PM_2.5_, we used land-use regression (LUR) models^56^. Personal monitoring of PM_2.5_ exposure throughout pregnancy in large cohorts is logistically challenging and cost-prohibitive. The use of LUR modeling likely reduced exposure misclassification compared to simpler interpolation methods such as inverse distance weighting. Nevertheless, some degree of exposure misclassification may persist, particularly due to temporal variability or individual mobility, which we were not able to fully account for. Future studies may benefit from incorporating higher-resolution spatiotemporal models, including hybrid approaches that integrate satellite-based data and chemical transport models, to further enhance exposure assessment accuracy. Moreover, the use of a general population-based, prospective birth cohort study enhances the generalizability of our findings to the broader population^57,58^. Second, by focusing solely on PM_2.5_ during pregnancy, we cannot completely exclude the influence of other environmental factors on the development of AD, such as indoor pollutants, pollens, fungal spores, and meteorological conditions, which may confound or modify the effects of PM_2.5_. Among these potential confounders, we adjusted for season of birth, as well as NO_2_ and ozone concentrations, and the significant association between PM_2.5_ and AD remained significant. PM_2.5_ exposure during early childhood did not affect the risk of AD, and even after adjusting for this exposure, the impact of prenatal PM_2.5_ exposure on AD remained significant, highlighting the importance of prenatal exposure. Stratified analyses across exposure years further confirmed the consistency of these findings, despite potential temporal variation in PM_2.5_ levels. Nevertheless, we acknowledge the complexity of air pollution exposure and highlight the need for future studies to apply refined multi-pollutant modeling approaches to better disentangle the interactive effects of various environmental factors on AD. In our study, PM_2.5_ was selected as a representative environmental exposure because of its well-documented association with systemic inflammation and its potential to induce long-lasting epigenetic modifications, or epigenetic alterations. Rather than isolating the effects of specific pollutants, we focused on PM_2.5_-induced epigenetic changes as an indicator of environmental insult, providing insights into the broader effects of exposure on immune function. To further validate these findings, we conducted in vitro experiments exposing M2 macrophages to PM, directly assessing its effects on their transcriptional profiles. Third, the relatively small sample size for placental methylome and transcriptome analyses, coupled with the dichotomization of PM_2.5_ exposure, likely reduced statistical power and constrained the generalizability of our results. It is also important to note that the low-exposure group in our cohort (<24.70 μg/m³) exceeded WHO, US EPA, and Korean guideline thresholds, indicating that even the lower category reflects moderate urban exposure and should be considered when generalizing to populations with cleaner air. Nevertheless, clinical characteristics were comparable between the analyzed subset and the full cohort, and rigorous tissue processing ensured data quality. Collection of large quantities of placental tissue is challenging, and comprehensive profiling methylome and transcriptome profiling requires highly specialized tissue processing. Additionally, we acknowledge that we did not directly assess the epigenetic profiles of dermal macrophages in adult AD skin tissue, which limits our ability to fully validate the maintenance of placental epigenetic alterations in affected skin. To address these limitations, we implemented complementary strategies including one-sample Mendelian randomization, leveraging placental genotypes to assess the causal relationship between *FCER1G* methylation and AD. Although exploratory due to sample size, these results aligned with our primary findings. Furthermore, we supplemented our findings with independent datasets spanning multiple developmental stages and disease contexts of AD. These included DNA methylome, chromatin accessibility, and transcriptome data, encompassing both bulk tissues and single-cell resolution, to reinforce the robustness and biological relevance of our conclusions. Specifically, single-cell trajectory analysis provided insights into the expression dynamics of *FCER1G* in M2 macrophages, revealing its association with the status of AD compared to healthy controls and indicating a possible mechanistic role in disease progression. These compensatory strategies, including enhanced exposure modeling, a targeted focus on PM_2.5_ as an environmental proxy, experimental validation, and the integration of multiple analyses and datasets, strengthen our findings and support the hypothesis that PM_2.5_ exposure induces durable epigenetic changes, particularly in M2 macrophages, which could play a central role in the development of AD. Future studies with larger sample sizes are warranted and should consider utilizing continuous exposure metrics or exploring alternative classification methods to more accurately capture the complexity of exposure–response relationships.

In conclusion, this study provides the most comprehensive evidence to date linking first-trimester PM_2.5_ exposure to an increased risk of AD in young children, identifying a prenatal epigenetic alteration in *FCER1G* that is associated with persistent dysregulation of *FCER1G* expression during development. Specifically, *FCER1G* hypomethylation and overexpression activate allergy-related genes, correlating with elevated eosinophil levels. The enrichment of *FCER1G*^+^ M2 macrophages in offspring with AD suggests these cells may prime immune responses linked to the risk of AD, partly through the activation of NADPH oxidase-mediated ROS signaling. Our findings identify potential biomarkers for early development of AD and highlight *FCER1G* as a candidate for therapeutic intervention, paving the way for targeted prevention and treatment strategies in immune-related diseases influenced by prenatal PM exposure.

## Methods

### Epidemiological study

#### Study populations

This study used data from the Cohort for Childhood Origin of Asthma and Allergic Diseases (COCOA), an ongoing prospective population-based birth cohort in Seoul, Republic of Korea^44,59^. COCOA was designed to represent an urban Korean population and to investigate how early-life genetic, epigenetic, psychosocial, nutritional, microbial, and environmental factors contribute to the development and natural course of allergic diseases. Recruitment began on 19 November 2007 and was conducted through five tertiary medical centers and eight public health centers providing antenatal care in Seoul. Pregnant women were eligible for enrollment before 26 weeks of gestation if they were residents of Seoul, planned to deliver at an affiliated medical center, and had no high-risk maternal conditions that could affect allergic disease development in the child, including diabetes, preeclampsia, anemia, or severe infection. Infant eligibility was assessed after delivery by collaborating obstetricians and pediatricians. Infants were excluded if they were born before 37 weeks of gestation, had major congenital anomalies, or experienced birth asphyxia requiring oxygen supplementation.

Mother–child pairs are followed according to a standardized protocol, with routine assessments of children at 6 months of age and planned annual assessments from 1 to 20 years of age. At enrollment and scheduled visits, structured questionnaires were administered to collect information on demographic characteristics, environmental exposures, and allergic and respiratory symptoms. Clinical examinations were conducted by pediatric allergy and pulmonology specialists, and blood samples were collected from children at 1 and 3 years of age. Children who were followed up for the first three years of life and had PM_2.5_ exposure data from the prenatal period to 3 years of age were included in this analysis. The study protocol, including the collection of human samples and the use of the COCOA dataset, was approved by the institutional review boards (IRBs) of Asan Medical Center (IRB number, 2008-0616), Samsung Medical Center (IRB number, 2009-02-021), Severance Medical Center (IRB number, 4-2008-0588), CHA Medical Center (IRB number, 2010-010), and Seoul National University Hospital (IRB number, H-1401-086-550). Written informed consent was obtained from the parents of each child. No participant compensation was provided.

#### Definition of AD

AD was determined through parental reports of physician-diagnosed AD at ages 6 months, 1 year, 2 years, and 3 years. The parental report was based on a positive response to the following written question: “Was your child diagnosed with AD by a doctor in the last 6 months or last year?“ During hospital follow-up visits, the presence of AD was clinically diagnosed by pediatric allergy specialists according to the criteria established by Hanifin and Rajka^60^. Children diagnosed with AD at any point during the study were considered to have AD incidence at age 3.

#### Measurement of outdoor PM_2.5_ exposure

Ambient PM_2.5_ exposure was estimated using a land-use regression (LUR) model for the Seoul metropolitan area^61^. The model was constructed using annual average PM_2.5_ concentrations measured at 37 regulatory air quality monitoring stations operated by the Korean Ministry of Environment and a comprehensive set of geographic information system (GIS)-derived predictor variables. The LUR approach was designed to characterize small-scale spatial variation in ambient air pollution and to provide individual-level exposure estimates based on residential location.

Hourly PM_2.5_ measurements obtained from the national monitoring network were aggregated into daily averages when at least 75% of hourly observations were available. Annual average concentrations were subsequently calculated for monitoring sites with at least 75% data completeness throughout the year. Geographic predictor variables were generated using ArcGIS (ESRI, Redlands, CA, USA) and classified into five categories: (1) traffic-related variables, (2) land-use characteristics, (3) population indicators, (4) topographic variables, and (5) spatial trend variables.

Traffic-related variables included the length of all roads, length of major roads, traffic intensity on the nearest road, traffic intensity on the nearest major road, traffic load, and heavy-duty traffic load. These variables were calculated within multiple buffer distances (25, 50, 100, 200, 300, 500, and 1,000 m) surrounding each monitoring location. Traffic load was estimated as a function of road length and traffic intensity, whereas heavy-duty traffic load was derived from the estimated volume of heavy-duty vehicles operating within the buffer area.

Land-use variables included residential, commercial, industrial, agricultural, forest, water, and urban green areas. The proportions or areas of these land-use categories were calculated within buffer distances of 100, 200, 300, 500, 1,000, and 2,000 m. Population-related variables included population density and the number of residents within the corresponding buffers. Topographic predictors included elevation and terrain characteristics. Spatial trend variables were generated from geographic coordinates (X and Y coordinates and their polynomial terms) to account for large-scale spatial gradients in pollutant concentrations.

A supervised forward stepwise regression procedure was used to develop the final prediction model. Candidate variables were sequentially evaluated according to their contribution to model performance, consistency with the expected direction of association, and absence of multicollinearity. The final model primarily incorporated traffic-related indicators, urban green space, and spatial trend variables, reflecting the dominant influence of traffic emissions and urban morphology on the spatial distribution of PM_2.5_ concentrations in Seoul.

Model performance was evaluated using leave-one-out cross-validation (LOOCV). The final PM_2.5_ model explained 66% of the spatial variability in measured annual PM_2.5_ concentrations (adjusted R² = 0.66), with a cross-validated R² of 0.56, indicating acceptable predictive performance.

Residential addresses of study participants were geocoded and linked to the LUR prediction surface. Individual PM_2.5_ concentrations were estimated at each residential location and assigned throughout the study period. For pregnancy-related analyses, exposure estimates were calculated for each trimester (weeks 1–13, 14–27, and 28–40) and for the entire pregnancy. Postnatal exposure was estimated as the annual average concentration for each year from birth to 3 years of age. If residential relocation occurred during the exposure period, the updated address was used to estimate air pollution exposure. The resulting LUR-based estimates were used as the primary indicators of long-term ambient PM_2.5_ exposure in subsequent analyses. Due to the narrow range of exposure levels, PM_2.5_ was categorized into tertiles to maximize exposure contrast. Analyses compared the middle and highest tertiles with the lowest tertile as the reference group. Multivariable logistic regression models were used to estimate the association between PM_2.5_ exposure and AD. Models were adjusted for sex, parental history of allergic disease, maternal age, maternal education, maternal body mass index, and exposure to secondhand smoke during pregnancy and infancy. Sex was ascertained from birth records, whereas all other covariates were obtained from self-reported information collected at enrollment.

#### Measurement of total serum IgE levels and eosinophils

Total IgE levels and peripheral blood eosinophils counts were measured in cord blood at birth and at 1 and 3 years of age and assessed using a fluorescent enzyme immunoassay according to the manufacturer’s instructions (AutoCAP System; Phadia AB, Uppsala, Sweden)^62^. Eosinophil counts were measured using an automated hematology analyzer.

### Placental tissue and umbilical cord blood collection and processing

Placental tissue samples were collected at delivery according to the standardized COCOA cohort protocol. After macroscopic inspection of the placenta, tissue specimens were obtained from macroscopically normal regions of the placental disc. Four tissue specimens were collected from the fetal-facing side and four from the maternal-facing side, representing the four quadrants around the umbilical cord insertion site. The umbilical cord insertion site itself was avoided during tissue sampling. Each specimen measured approximately 1 × 1 × 1 cm. Sampling was performed while avoiding areas with visible infarction, calcification, hemorrhage, hematoma, membranes, large vessels, the placental edge, or gross contamination. Each specimen was immediately placed into a pre-labeled cryovial. The collected samples were immediately transported to the laboratory on ice and stored in liquid nitrogen prior to nucleic acid extraction.

Umbilical cord blood was collected immediately after delivery from the umbilical vein into CPDA-1 anticoagulant-containing blood collection bags and processed within 24 h of collection. Cord blood mononuclear cells were isolated using Ficoll-Paque density-gradient centrifugation. Briefly, cord blood was carefully layered onto Ficoll-Paque and centrifuged at room temperature at 2,500 rpm for 25 min with the brake disabled. After centrifugation, plasma was aliquoted into 1.5-mL tubes and stored at −80 °C, and the red blood cell fraction was also aliquoted and stored at −80 °C. The mononuclear cell layer was carefully collected and washed with phosphate-buffered saline. Residual red blood cells were lysed by treatment with 10 mL of 0.83% ammonium chloride solution (8.32 g/L) with gentle pipetting for 2–3 min. The cell suspension was then centrifuged at 4 °C at 1500 rpm for 5 min, and the resulting mononuclear cell pellet was stored at −80 °C until DNA extraction and genetic analyses.

#### Methylation and transcriptome analysis

To investigate the mechanisms underlying AD associated with prenatal PM_2.5_ exposure, we selected 48 placental samples and 46 cord blood samples from the COCOA cohort, based on PM_2.5_ exposure levels during the first trimester of pregnancy and the diagnosis of AD in offspring at 2 and 3 years of age. The first trimester was chosen based on biological plausibility, as this period encompasses critical stages of fetal immune maturation and epigenetic programming, while also marking the initiation of epidermal and dermal development as well as the establishment of cutaneous immune cell populations^63–65^. There were no differences in the sampling of clinical characteristics, except for PM_2.5_ exposure levels during pregnancy (Supplementary Data 4–5). For methylation analysis, DNA was extracted from placental tissue and cord blood buffy coat using the DNeasy Blood & Tissue Kit (QIAGEN, Redwood City, CA, USA) in accordance with the manufacturer’s protocol. DNA methylation (DNAm) was measured using the Infinium MethylationEPIC BeadChip (Illumina, San Diego, CA, USA). Methylation levels at each CpG site were summarized as a β-value, defined by the ratio of the methylated (M) to unmethylated (U) signal, calculated as β = (max(M, 0))/( | U | + |M | + 100). For transcriptome analysis, total RNA was isolated from placental tissue and cord blood leukocytes using Trizol reagent (Invitrogen, Carlsbad, California) from the matched samples used in the methylation analysis. cDNA was synthesized using the GeneChip Whole-Transcript Pico Reagent Kit (Affymetrix, Santa Clara, California), following the manufacturer’s protocol. The cDNA samples were analyzed using the GeneChip® Human Gene 2.0 ST Array (Affymetrix). Raw data were summarized and normalized using the robust multi-average method implemented in Affymetrix Power Tools.

#### Genotyping, quality control, and imputation of genetic data

Genomic DNA was extracted from buffy coat in blood using the WizPrep gDNA Mini Kit (Wizbiosolutions, Seongnam, Korea) according to the manufacturer’s protocol. Whole-genome genotyping was performed using an Infinium Global Screening Array-24 v3.0 BeadChip (Illumina, San Diego, CA USA) according to the manufacturer’s protocol using approximately 200 ng genomic DNA. All samples were scanned using the iScan microarray scanner (Illumina), and data processing of the normalized signal intensity and genotype was computed using the Illumina/BeadArrayFiles Python library (Illumina). Quality control (QC) procedures were executed using PLINK. The sample QC was conducted with exclusion criteria set for samples with (1) call rates below 95%. After sample QC, single nucleotide polymorphisms (SNPs) were filtered based on (1) a call rate threshold of less than 99% or (2) deviation from the Hardy-Weinberg equilibrium (HWE) test (raw *p* value < 1 × 10^−6^), or (3) they were located in the sex chromosome, or (4) Minor allele frequencies (MAFs) < 0.05. After sample and variant QC, 324,199 SNPs of 24 individuals (12 AD patients exposed to high PM_2.5_ and 12 healthy controls exposed to high PM2.5) were used for downstream analysis.

#### One-sample bidirectional Mendelian randomization analysis

To investigate the causal relationship between altered methylation induced by high PM and AD status, one-sample Mendelian randomization (MR) analysis was conducted. We assessed causality using high-PM_2.5_ exposed methylation pattern as exposure variables (X) and clinical indicators (i.e, AD or control) as outcome variables (Y). To assess the causal effect of methylation levels on clinical outcomes, SNPs associated with each CpG site were identified using linear regression at a significance threshold of raw *p* value < 1e–4. Three covariate sets were considered: set 1 included the child’s sex; set 2 included the child’s sex, parental age, and maternal AD status; and set 3 additionally included birth weight, maternal secondhand smoke exposure, or maternal cotinine level. Principal components (PC1–PC5) were also included as covariates. Independent SNPs were identified using clumping analysis in PLINK, with following set parameters: raw *p* value (--clump-p1 1e-04), genomic distance (--clump-kb 1000), and linkage disequilibrium (LD, --clump-r2 0.2). SNPs significantly associated with clinical indicators or confounders (raw-*p* value < 5 × 10^-2^) were excluded to satisfy the key assumptions of MR. Subsequently, a two-stage least square regression (TSLS) analysis was implemented using the R package ivreg (version 0.6.4).

### Experimental study

#### Chemicals

The following chemicals were used in this study: Urban particulate matter (PM) (NIST1648A, Sigma-Aldrich, St. Louis, MO, USA), Diesel PM (NIST1650B, Sigma-Aldrich), and Phorbol 12-myristate 13-acetate (PMA, Sigma-Aldrich).

#### Dispersion

Urban PM (25 and 100 µg/mL) and Diesel PM (12.5 and 50 µg/mL) were dispersed in RPMI 1640 medium and then sonicated using the SONICS Vibra Cell™ (Sonics, USA). Sonication was performed at 14 °C for 15 min, with settings of pulse on for 50 s and pulse off for 10 s.

#### Cell culture and differentiation

The monocyte-derived cell line THP-1 (TIB-202TM) was purchased from the American Type Culture Collection (ATCC, Manassas, VA, USA) and cultured at 37 °C in a humidified atmosphere containing 5% CO₂. Cells were maintained in RPMI 1640 medium (Welgene, Gyeongsan-si, South Korea) supplemented with 10% fetal bovine serum (FBS, Thermo Fisher Scientific, Seoul, South Korea/Inc., Waltham, MA, USA) and 1% penicillin–streptomycin (100X, Welgene). THP-1 cells (5 × 10⁵ cells/well) were differentiated into macrophages by treatment with 25 ng/mL of phorbol 12-myristate 13-acetate (PMA, Sigma-Aldrich) for 72 h in 6-well plates.

#### Real-time quantitative reverse transcription polymerase chain reaction (qRT-PCR)

THP-1-derived macrophages were cultured in 6-well plates at a density of 5 × 10^5^ cells/well at 37 °C. To induce M2 macrophage differentiation, cells were treated with 20 ng/mL of IL-4 (R&D systems, Minneapolis, MN, USA). Differentiation conditions were optimized by analyzing the expressions of M1 and M2 markers for 24, 48, and 72 h. The highest expression of the M2 marker was observed at 72 h, which was selected as the optimal differentiation time. M2-differentiated macrophages were exposed to various concentrations of Urban PM and Diesel PM for 24 h, after which the media containing Urban PM and Diesel PM were removed. RNA was extracted from the cultured macrophages using TRIzol (RNAiso Plus, Takara, Tokyo, Japan) reagent. cDNA was synthesized using the SmartGene Compact cDNA Synthesis kit (SmartGene, Daejeon, South Korea) with 2 μg of extracted RNA in a 20-µL reaction volume. Real-time PCR was conducted in a 96-well plate with a final volume of 20 µL, containing 6 µL of DEPC-treated water (Bioseang, Yongin-si, Korea), 10 µL of THUNDERBIRD™ Next SYBR™ qPCR Mix (Toyobo, Osaka, Japan), 1 µL of each primer, and 2 µL of cDNA. To detect the expression of M1 and M2 markers, primers for TNF-α, IL-6, IL-10, and CCL17 (M2 markers) were used, and real-time PCR was performed using *FCER1G, CYBB, MRC1, and MS4A4A* primers to detect the expressions induced by Urban PM and Diesel PM.

#### DNA methylation analysis in M2 macrophages

DNA samples were assessed for quality using a NanoDrop® ND-2000 UV–Vis spectrophotometer and further evaluated by agarose gel electrophoresis. Only samples showing intact genomic DNA without evidence of degradation were selected for downstream analysis. Genomic DNA was quantified using the Quant-iT PicoGreen assay (Invitrogen) and diluted to a final concentration of 50 ng/µL. All samples were bisulfite-converted according to the manufacturer’s protocol using the Zymo EZ DNA Methylation Kit. DNA methylation profiling was performed using the Infinium MethylationEPIC BeadChip. Beta values were obtained after background correction and normalization using the ENmix pipeline, followed by quantile normalization.

### Computational methods

#### Differential methylation and expression analysis

To examine the impact of prenatal PM exposure on the epigenome and transcriptome and its association with AD, we compared the AD status of offspring between low and high PM exposure levels. PM_2.5_ exposure during the first trimester was dichotomized based on tertile distribution, with the lowest tertile (<24.70 µg/m³) designated as the low-exposure group and the highest tertile (>29.21 µg/m³) as the high-exposure group, to accommodate the limited sample size and facilitate robust statistical comparisons. These cut-offs reflect the actual distribution of urban PM_2.5_ exposure in our study population, rather than strict health-based guidelines. DMCs were identified using the criteria absolute β-value > 0.02 and *P* value (Wilcoxon rank-sum test) <0.05. DEGs were selected based on *P* value (Wilcoxon rank-sum test) <0.05. Covariate analysis for both the methylome and transcriptome was conducted using the statsmodels.regression.linear_model.OLS function in Python to account for confounding factors such as fetal sex, weight, and maternal AD status. We excluded results from the low PM exposure groups to identify the specific pattern of AD in offspring with high PM_2.5_ exposure.

#### Gometh

We used the gometh function in the missMethyl R package^66^ to perform gene ontology (GO) and KEGG pathway enrichment analysis for differentially methylated CpGs on the Illumina EPIC array. This analysis addresses potential biases resulting from the differing number of probes per gene and the annotation of CpGs to multiple genes.

#### Gene set variation analysis (GSVA)

We used the Gene Set Variation Analysis (GSVA) function in the GSVA R package^67^ to calculate pathway enrichment scores using gene sets of GO, KEGG, and Reactome. This allowed us to investigate pathway activity in individual samples. We then selected pathways that were enriched in offspring with AD exposed to high PM_2.5_ relative to those without AD, using the Wilcoxon rank-sum test (*P* < 0.05). Pathways that showed significant enrichment in the low PM exposure group were excluded from the analysis.

#### Gene set enrichment analysis

Gene Set Enrichment Analysis of module gene signatures was performed on our placental transcriptome data using the gseapy function in Python, with default parameters^68^.

#### Cell-type score

To generate module scores for adult M2 macrophages and the *FCER1G*-directed neighbor gene score for each cell type, we used the scanpy.tl.score function (use_raw = False) in Python^69^.

#### Triplet analysis

To systematically identify CpG methylation with significant regulatory effects on gene expression by influencing TF activity, we used the MethReg R package^17^. We used JASPAR2022^70^ and ReMap2022^71^ databases for TF motif scanning at CpG sites to identify potential transcription factor binding sites. CpG sites were mapped to target genes using the create_triplet_distance_based function in MethReg. Regulatory CpGs were classified as:

- proximal CpGs: located ±2 kb around the transcription start site (TSS), linked to target genes.
- distal CpGs: located >2 kb from the TSS, linked to all genes within a fixed distance window of ±250 kb.

These distance settings are considered default parameters. Each triplet (CpG-TF-Target Gene) was assessed using the interaction_model function, a robust linear model with the following formula: ${\log }_{2}\left({{{\rm{Target}}}}\; {{{\rm{Gene}}}}\right)\sim {\log }_{2}\left({{{\rm{TF}}}}\right)+{{{\rm{DNAm}}}}\; {{{\rm{Group}}}}+{\log }_{2}\left({{{\rm{TF}}}}\right)\times {{{\rm{DNAm}}}}\; {{{\rm{Group}}}}$

TF activity scores were calculated using the DoRothEA^72^ and VIPER R packages^73^. DNAm Group was defined as a binary variable, representing high (above median) or low (below median) DNA methylation levels at the CpG site. MethReg classified the role of TFs in target gene expression and the effect of DNA methylation on TF activity using the stratified_model function. We did not consider scenarios where high DNA methylation increases TF activity in this analysis. Based on the ReMap (ChIP-seq) database, we first identified associations between regulatory CpG and transcription factors (TFs) within ±200 bp of the CpG sites. Given that TFs recognize specific DNA sequence motifs of 6–12 bp^74^, we investigated the direct association of regulatory CpG with TFs by performing TF binding site analysis on sequences within ±12 bp of the CpG site using the TFBStools R package^75^. Only results with *P* values (TFBStools) < 0.05, where the CpG site was directly included in the TF motif, were selected.

#### Epigenetic data

The ChIP-seq, DNase-seq, and Hi-C data used in this analysis were all obtained from ENCODE. We integrated each BAM file (ChIP-seq and DNase-seq) using the merge function in SAMtools^76^. The callpeak function (ChIP-seq: -p 0.01, DNase-seq: -p 0.01 --nomodel --shift −100 --extsize 200) in MACS3 was used to call the significantly enriched regions as peaks. Hi-C data from ENCODE was converted from.hic files to.cool files at 10 kb resolution using hicstraw in Python^77^. The.cool files were then converted to.h5 format using the hicConverFormat function to define topologically associating domains (TADs). TADs were identified using the hicFindTADs function (mindepth = 10 kb, maxdepth = 100 kb) in HiCExplorer^78^. The identified peaks and TADs were saved in BED format. BEDtools intersect function^79^ was used to investigate biological triplets (CpG-TF-Target gene) by assessing the overlap between regulatory CpG sites and active marker peaks (H3K27ac, H3K4me3, H3K4me1, DNase-seq). The target genes were examined to determine whether they were located within the same TAD. BigWig files for the ChIP-seq and DNase-seq signals were integrated using the bigwigAverage function in deepTools^80^. To compare two BigWig files (e.g., placenta vs. basal plate, macrophages vs. T cells), we used the bigwigCompare function in deepTools. The resulting BigWig files were visualized using the UCSC Genome Browser^81^. Hi-C heatmaps were generated using the hicPlotMatrix function in HiCExplorer.

#### *FCER1G*-associated gene signatures

The *FCER1G*-associated gene signature was derived from bulk and scRNA-seq data. For the bulk transcriptome-based gene signature, we curated genes from the following pathways: innate and immune response, adaptive immune response, immune effector process, positive regulation of immune system, inflammatory response, myeloid leukocyte activation, myeloid leukocyte migration, leukocyte chemotaxis, leukocyte-mediated immunity, response to cytokines, cytokine production, cytokine-mediated signaling pathway, response to ROS, NADPH oxidase complex, superoxide-generating NAD(P)H oxidase activity, transmembrane receptor protein kinase activity, positive regulation of protein tyrosine kinase activity, protein kinase C activity, protein kinase C binding, PI3K/AKT signaling in cancer, and MAPK signaling pathway. Next, we selected genes that were positively correlated with *FCER1G* expression using Pearson correlation analysis, resulting in 1,000 genes. We used the GSVA R package to calculate the GSVA score based on these genes. For scRNA-seq data-based gene signatures, gene signatures were derived through network analysis using scHumanNet. We used the scanpy.tl.score function (use_raw = False) in Python to calculate the gene signature score.

#### Digital cytometry analysis

Cell composition was inferred using an enrichment method (xCell^82^) and two deconvolution methods (AutogeneS^22^ and CIBERSORTx^23^). xCell with default parameters was applied to our placenta data, selecting relevant cell types for calculating the ImmuneScore. AutogeneS was applied to our placental data and bulk transcriptomes from infant, child (GSE107361), and adult (GSE121212) skin. scRNA-seq data were used to calculate centroids of annotated cell states based on the top 2000 highly variable genes. These centroids were then applied to deconvolute bulk data using the Nu-support vector machine (Nu-SVR) method. CIBERSORTx was applied to the same dataset as AutogeneS. The cibersortx/fractions docker container (--rmbatchBmode True --perm 1 --single cell TRUE) was used to quantify cell proportions from bulk transcriptome data. According to previously reported data, only fetal-derived cell types, namely extravillous trophoblast (EVT), syncytiotrophoblast (SCT), villous cytotrophoblast (VCT), fibroblast (FB), erythrocyte/blast (Ery), and Hofbauer cell (HBC), were included from the placental scRNA-seq data. Previous adult scRNA-seq studies annotated cell types at a fine-grained level. To minimize collinearity, these were merged into coarse-grained categories (e.g., VE1, VE2, and VE3 were grouped into “VE”), except for macrophages.

#### Single-cell RNA-seq analysis

scRNA-seq datasets were obtained from E-MTAB-6701, GSE171381, E-MTAB-7407, and E-MTAB-8142. Genes detected in fewer than three cells and cells expressing fewer than 500 genes were excluded. To integrate multiple datasets, we used the concatenate function in Scanpy. The data were normalized using the scanpy.pp.normalize_total function, and these normalized values were used for downstream analyses and visualization. The scVI tool^83^ was used for batch correction. Uniform Manifold Approximation and Projection (UMAP) embeddings were generated using the scanpy.tl.umap function in Scanpy to visualize cellular states. The scanpy.tl.leiden function was used to perform unsupervised clustering with the Leiden algorithm. M2 macrophage states with high *FCER1G* expression (*FCER1G*^+^ M2) were identified in Leiden clusters 2 and 3, which were associated with AD.

#### Phenotypic similarity between placental cells, fetal skin cells, and adult cells

We used the FindTransferAnchors function in Seurat to identify phenotypic similarities between the placenta (query) and fetal (reference) datasets, as well as the adult (reference) dataset. Since several placental cell types were associated with multiple fetal cell types, we performed this function in reverse—using the placenta as the reference and the fetal dataset as the query—to identify the most closely associated cell types.

#### Conserved gene signature

We used the FindConservedMarkers function in the Seurat R package (min.pct = 0.5, logfc.threshold = 0.25, *p*_val_adj <0.05) to identify conserved marker genes across Hofbauer cells, fetal skin macrophages, and adult M2 macrophages. These cell types were identified as phenotypically similar based on the FindTransferAnchors results.

#### RNA velocity and pseudotime analysis

We used velocyto^84^ to obtain unspliced and spliced count matrices. CellDancer^31^ was used to calculate RNA velocity for each module, and the results were further analyzed using Dynamo^32^. Dynamo was used to estimate the transition probability between M2 macrophage states using the VectorField function (basis =’umap’, pot_curl_div = True). Vector field-based pseudotime was computed using ddhodge potential. Markov affinity-based graph imputation of cells MAGIC^85^ and generalized linear models using the linearGAM function in pygam were used to visualize gene expression and splicing trajectory along pseudotime.

#### Network analysis

Weighted Gene Co-expression Network Analysis in Python^86^ was used to generate an adjacency matrix, integrating scRNA-seq data from placental HBCs, fetal skin, and adult skin cells. This adjacency matrix was used to identify modules and visualize them using Gephi. To construct cell type-specific networks (CSNs) and incorporate biological knowledge, we used scHumanNet^33^, which utilizes the interactome database called HumanNet. The SCINET algorithm (min.weight) in scHumanNet was optimized with our approach to construct CSNs. The optimized weight was determined when the genes derived from each CSN showed low overlap with genes from other CSNs and high overlap with marker genes. Potential marker genes were identified using the FindAllMarkers function (parameters: only.pos = TRUE, logfc.threshold = 0.25, min.diff.pct = 0.25, min.pct = 0.25). We used the Jaccard index (J(A, B) = ∣A ∩ B∣/∣A∪B∣) to calculate these overlaps (similarity). The minimum weight was set at 2 and increased by 0.1 until each CSN contained at least 100 genes. Rankings were assigned based on lower Jaccard index between CSNs and higher Jaccard index between CSN genes and marker genes. The final optimized weight was determined by the lowest sum of the two rankings.

A conserved network was constructed by connecting shared nodes (genes) across placenta HBCs, fetal skin, and adult AD M2 macrophages.

#### Spatial transcriptome analysis

Spatial transcriptome (ST) data were obtained from GSE197023 and preprocessed and annotated using Seurat, in which each sample was log-normalized and integrated across samples using reciprocal principal component analysis (RPCA). Unsupervised clustering was performed with the Louvain algorithm (resolution = 0.1), and clusters were annotated based on known marker genes. The analysis pipeline is publicly available at (https://gitlab.com/juno.kim/visium-atopic-dermatitis. ST data were analyzed using cell2location^87^, which estimates reference gene expression signatures of cell states from scRNA-seq data. These signatures were then used to estimate the abundance of cell states in the spatial data. Cell-type-specific gene expression in the spatial data was estimated using the NCEM model^88^. We used CellChat^89^ to identify the potential ligand–receptor interactions.

### Reporting summary

Further information on research design is available in the Nature Portfolio Reporting Summary linked to this article.

## Supplementary information

Supplementary Information

Description of Additional Supplementary Files

Supplementary Data 1

Supplementary Data 2

Supplementary Data 3

Supplementary Data 4

Supplementary Data 5

Supplementary Data 6

Reporting Summary

Peer Review File

## Source data

Source Data

## Data Availability

Our placenta and cord blood data in this study are available in the Zenodo under accession code 14998855^90^. DNA methylation data from peripheral blood samples are available in the Gene Expression Omnibus under accession code GSE152084, and PBMC-derived monocyte ATAC-seq data are available under accession code GSE255396. RNA-seq data are available in the Gene Expression Omnibus under accession code GSE107361 (infant and child atopic dermatitis data) and GSE121212 (adult atopic dermatitis data). scRNA-seq data generated in this study have been deposited in the Gene Expression Omnibus under accession code GSE171381 (term placenta data) and the ArrayExpress database under accession code E-MTAB-6701 (early placenta data), E-MTAB-7407 (healthy fetal skin data), and E-MTAB-8142 (healthy and AD adult data). Additional scRNA-seq data for healthy and AD adult skin are available via the Single Cell Portal under accession code SCP2738. Spatial transcriptomics data generated in this study have been deposited in the Gene Expression Omnibus under accession code GSE197023. Source data are provided with this paper.

## Code availability

The code used for data analysis in this study is available at GitHub (https://github.com/sysbioinfo-khu/PM_AD) or Zenodo^91^ (https://doi.org/10.5281/zenodo.14998809).
