# Supplementary material for: Prenatal PM2.5 exposure drives epigenetic reprogramming of fetal macrophages linked to atopic dermatitis
Source: Nat Commun. 2026 Aug 8;17:9531. doi: 10.1038/s41467-026-76298-6 (PMC13547331; doi:10.1038/s41467-026-76298-6)
Supplement: Supplementary file 1 [file 41467_2026_76298_MOESM10_ESM.pdf]

nature portfolio

Peer Review File

Prenatal PM2.5 exposure drives epigenetic reprogramming of fetal macrophages linked to atopic dermatitis

Corresponding Author: Professor Kwoneel Kim

This file contains all reviewer reports in order by version, followed by all author rebuttals in order by version.

Version 0:

Reviewer comments:

Reviewer #1

(Remarks to the Author)
In this manuscript, the authors aim to assess the effects of prenatal exposure to particulate matter (PM) on the development of pediatric atopic dermatitis (AD). They conducted DNA methylome and transcriptome analyses of placental tissue and found that placentae exposed to high levels of PM exhibited hypomethylation in several regulatory regions, potentially leading to overexpression of the FCER1G gene. Re-analysis of publicly available transcriptomic data showed that FCER1G is predominantly expressed in fetal macrophage-like Hofbauer cells. Given that the expression profile of placental Hofbauer cells is similar to that of M2 macrophages in the skin, the authors hypothesize that FCER1Ghi M2 macrophages may persist from the prenatal period and function as inflammation hubs, possibly through the induction of ROS signaling.
However, the evidence supporting the authors' main hypothesis remains very weak, particularly in relation to the central claims presented in the manuscript's title: "Prenatal particulate matter exposure imprints an epigenetic scar on FCER1G in fetal macrophages, reemerging in M2 macrophages to drive atopic dermatitis development." While the authors report hypomethylation of FCER1G in placentae exposed to PM, they provide no direct evidence that this so-called "epigenetic scar" persists into postnatal life, nor that it reappears in dermal M2 macrophages during the course of AD. The study does not demonstrate whether the epigenetic alterations observed in fetal Hofbauer cells are retained in the macrophages of pediatric or adult AD patients, especially in the skin. Thus, the proposed mechanistic link between prenatal PM exposure and epigenetic imprinting in macropahges remains largely speculative. Below are specific concerns:

Major Concerns
Major Concern #1:
Although the authors suggest that the "epigenetic scar" observed in placental samples persists in adult AD skin lesions, they did not directly assess the epigenetic profiles of adult AD skin tissue. The authors should examine whether similar epigenetic alterations are present in dermal macrophages of AD patients.

Major Concern #2:
A large portion of the study relies on publicly available data from AD patients. The authors must validate the inferences drawn from transcriptomic analysis. For example, in Figure 4c, the authors predicted an increased frequency of Hofbauer cells in high PM-exposed AD patients using deconvolution analysis of bulk RNA-seq data. Similarly, they predicted an increase in FCER1Ghi M2 macrophages in AD skin lesions under high PM exposure. These findings should be validated through experimental methods such as immunostaining.

Major Concern #3:
While FCER1Ghi dermal macrophages appear to be more frequent in AD patients than in healthy controls, their functional role in skin allergic inflammation remains unclear. Is the increased frequency of FCER1Ghi dermal macrophages associated with AD severity or type 2 inflammatory markers such as IL-4 and IL-13?

Minor Concerns
Minor Concern #1:
In most parts of the study, the authors compare AD patients and healthy subjects who were exposed to high levels of PM. However, given the study's objective of assessing the effects of PM exposure on AD development, it would be more appropriate to compare AD patients with high versus low PM exposure.

Minor Concern #2:
Recent advances in single-cell RNA sequencing have revealed that the traditional M1/M2 macrophage classification oversimplifies the complexity of macrophage heterogeneity. The authors should consider using a more refined classification to characterize dermal macrophages in AD skin lesions.

Reviewer #2

(Remarks to the Author)
The authors address a potentially very important, yet still insufficiently understood phenomenon of AD, namely prenatal imprinting of the immune system.

Major

The authors need to explain better what the nature of the particulate matter is. It remains unclear to the reader.

It remains unclear whether the increase in PM is rooted in a true causality, or a confounder. Please address better potential confounding factors. This remains unclear to the reader.

What is the chemical nature of PM?

Minor

Please explain in abstract what PM2.5 in fact is.

Reviewer #3

(Remarks to the Author)
This manuscript addresses the key research question of how prenatal exposure to PM2.5 may contribute to the development of atopic dermatitis (AD) in childhood. The authors used a multi-faceted analytical approach and multi-omics data to examine prenatal environmental exposures in the early-life risk of atopic dermatitis. Integrated omics approach and focusing both methylation and gene expression in relation to AD is novel and adds meaningful knowledge to the literature.

The authors perform a epidemiological analysis, examining a COCOA cohort of 986 children to assess whether first-trimester PM2.5 exposure is associated with a significantly increased risk of AD by age three. Furthermore, the study leverages comprehensive epigenomic and transcriptomic profiling of 48 placentas to investigate the biological mechanisms linking prenatal air pollution exposure to subsequent AD risk. They found an epigenetic scar involving FCER1G, which was hypomethylated and overexpressed in the placenta of children who later develop AD. The authors identified FCER1G^{+} M2 macrophages in the placenta as key cellular carriers of this epigenetic signature. These macrophages show enriched expression of genes involved in ROS signaling, a pathway associated with inflammatory responses and AD development. Their in vitro experiments showed that high $PM_{2.5}$ exposure may induce this molecular profile. Their findings suggest that prenatal air pollution may epigenetically program immune responses, increasing AD risk through ROS-activated M2 macrophages.

However, there are several major issues that need to be addressed before the manuscript can be considered for publication. Most importantly, the manuscript should clearly explain how PM2.5 exposure was dichotomized and provide the rationale for this classification. Without clarifying what is considered ‘low’ and ‘high’ exposure, it is difficult to interpret and draw conclusions from the findings.

Major issues:
Data:
1. It is unclear which datasets were used for each analysis. The study population is described as a cohort study using the COCOA cohort, which was then matched with air pollution data. My understanding is 986 children was only used for figure 1a, and then 48 placenta and cord blood were used for other analysis. However, it is confusing where the adult samples are from. COCOA only follow up until 10 years according to their descriptions, so I guess adults are not from this cohort. There is no description of what the adult samples are or where they come from in study population section. Additionally, there are no summary statistics or explanations for the healthy and AD adult groups. How are AD adults defined? Is the definition consistent with the criteria used for children? Also, are the placental and fetal datasets derived from the COCOA cohort?

Abstract:
2. The statement in abstract, “We hypothesize that prenatal PM2.5 exposure induces specific epigenetic modifications, primarily hypomethylation of immune-related genes in certain placental immune cell types. This epigenetic scar could later re-emerge in offspring and adult immune cells, potentially priming them for heightened inflammatory responses and increased AD risk.” may overstate the evidence provided in this study.

Specifically, it is not clear whether the COCOA data set includes follow-up of children into adulthood, or whether your adult

skin cell samples include corresponding prenatal exposure data. Without longitudinal data linking prenatal exposure, childhood, and adult outcomes within the same individuals, it is difficult to assert that the observed epigenetic modifications re-emerge in adult immune cells as a consequence of prenatal PM2.5 exposure. There are other potential confounding factors and exposures that could influence the epigenome after the prenatal period.

Method:
3. Did you account for any changes in participants' residential addresses during pregnancy? If so, how was this handled in the exposure assignment?
4. You mention using "station records routinely"—could you clarify the time interval for PM2.5 measurements (e.g., hourly, daily)? What kind a land-use regression (LUR) model or another type of exposure model used?
5. How were the 1–3 year PM2.5 exposure data collected? Were these based on participants' residential addresses during pregnancy or at another time point?
6. PM2.5 levels were categorized into tertiles (T1–T3, n = 138) but the overall cohort size is stated as 986. Please clarify this discrepancy: how were exposure groups constructed and how do they relate to the full cohort size? Also, why used tertiles in logistic regression analysis and then use two low vs high group in other analysis.
7. Figure 1a states that models were adjusted for sex, parental allergic history, maternal age, education, BMI, secondhand smoke, and breastfeeding. Elsewhere, only fetal sex, weight, and maternal AD status are mentioned. Please clarify which confounders were included in which models.
8. Did you adjust for the effects of other air pollutants or seasonality in your analysis? This is important, as both factors can influence PM2.5 levels and the incidence of AD. For example, in Korea, springtime often brings elevated PM2.5 and dust pollution due to wind patterns. Failing to account for these variables could confound your results. Please clarify whether and how these factors were considered in your exposure assessment and statistical models.
9. How did your account for potential PM2.5 exposure after birth (i.e., in infancy or early childhood)? Postnatal exposure can also increase the risk of AD. How did you adjust for this?
10. Please explain your rationale and logic for dividing the exposure groups (low and high). What exposure do you use? Entire? First trimester? It is not clear. What does high mean? It is well known that there is no safe level for AAP, and without showing how you defined the low vs high dichotomize the exposure is not biologically justified. While the dichotomization is necessary given the small sample size and limited power (which the authors may need to mention), the authors may explore other outpoints to assess the robustness of the results. Also, without showing how you set low vs high, the statement that high exposure may lead the AD risk cannot be jsutified. Did you follow the WHO guideline? The World Health Organization (WHO) also recommends that annual average PM2.5 concentrations should not exceed 5 μg/m³, and 24-hour average exposures should not exceed 15 μg/m³ more than 3-4 days per year
11. Also, this is setting from Korea, it will be important to show what is average 2.5 exposure that pregnant women's confront and what would be considered as low or high, for readers not from Korea.
12. If you have categorized the groups based on first-trimester PM2.5 exposure, did you also examine whether there were differences in your results when using PM2.5 exposure from other periods of pregnancy?
13. Air pollution exposures were assessed using modeled estimates based on an inverse distance-squared weighting, which is not considered as the gold standard model to estimate ambient air pollution. To minimize exposure misclassifications, the authors may use other well-validated modeled air pollution estimates with high spatiotemporal resolutions.
14. Was multiple testing correction applied when evaluating significance (e.g., for several endpoints or pathways)? If so, please specify the method used. If you didn't use, why?
15. Pathway enrichment analysis shows pronounced activation in AD offspring with high PM2.5, but low-PM/healthy and -AD groups have near-zero mean scores, while high-PM/healthy shows the lowest scores. It appears that PM2.5 exposure has opposing effects by AD status: activating in AD but deactivating in healthy. How do you interpret these findings? Please elaborate on possible biological explanations for this phenomenon.
16. Figure 3d -> boxplot doesn't distinct which one is low pm -healthy, low PM-ad, and High pm-healthy. Also, the boxplot shows that healthy child has lower count while AD has higher count. Also, not clear the p-value is indicating. Comparing from what? The effect of PM2.5 was same for both healthy and AD group?
17. The "epigenetic scar" hypothesis would be best supported by formal mediation analysis, quantifying the role of methylation changes in mediating the PM2.5 → AD association. Have you tried this mediation analysis?
18. It would be helpful to draw a schematic diagram on causal path on pm to methylation change to immune alteration to ad to help readers follow the reasoning.
19. Why supplementary table 2 and 3 only present PM10? PM2.5 info is missing.

Minor
20. Line 214: **P ≤ 0.05 -> * P ≤ 0.05
21. Boxplot 2-d. children (age between 1 and7??)
22. 2C is boxplot, not clear -> what is the sample?

Reviewer #4

(Remarks to the Author)
The study by Yang et al aims to link prenatal PM2.5 exposure, FCER1G dysregulation and AD developments, by integrating analyses of placental epigenomic and transcriptomic data. The finding is interesting, but the statistical power is relatively low due to small sample size and no consideration of multiple-testing. The manuscript is well written. Attached please find my other comments.

1. In the analyses between air pollution and risk of AD in children, only sex, weight (at which time point?) and maternal AD status was considered as the covariates. However, other potential factors should also be considered, such as social-economic status, maternal/paternal smoking status, feeding pattern and season of birth (which may lead to variation in PM exposure) as well as PM2.5 exposure during early childhood. Some studies showed that PM2.5 concentration generally decline over years, does AD prevalence change over years in the study cohort? If it is, then years when PM2.5 was measured should as be considered as a covariate.

2. More details are needed for PM2.5 measurement: such as, how dense are the monitor stations? How far in general the address of each studied participant from the nearest monitoring station? Are monitoring data available weekly, daily or hourly? Any records are available to reflect whether the participants changed address during pregnancy?

3. Preterm birth may play a role in immune development and AD incidence. What is the percentage of preterm birth among the total 986 samples and among the 48 samples for epigenomic and transcriptomic analyses?

4. How were the 48 samples selected from the 986 total samples? How high-PM2.5 and low-PM2.5 is defined? Are the population characteristics for the 48 samples and the rest 938 samples comparable?

5. More detailed information is needed for diagnosis of AD. For example, if a kid has a physician diagnosis of AD at age 2 but not age 3, is the kid still classified as the cases with AD? Any other tests have been performed to rule out other skin conditions ? Was IgE or skin tests applied to confirm AD cases?

6. Identification of DMCs specific to PM2.5 did not account for multiple testing; In Figure 1c, why comparison between two groups was shown for pathway enrichment using 12939 CpGs, while comparison among four groups was shown for pathway enrichment using 1.123 DECs? At the left panel, does gene ratio (the x axis) actually represents the difference of gene ratio between the two groups? More annotation is needed for broader readers.

7. When t-tests (line 214) were applied to compare difference of a continuous variable between two groups, did the authors check the distribution of the analyzed variables? Or any transformation was applied to approximate normal distribution?

8. When analyzing epigenome using DNA from cord blood, cell types should be adjusted as covariates.

9. Given that most of the findings from this study are based on association analyses or correlation analyses which is observational, the authors may not be able to strengthen that the identified hypomethylations or altered gene expressions are induced or caused by high PM2.5 exposure (such as, Line 182: ".. between altered methylation induced by high PM2.5 exposure.).

Version 1:

Reviewer comments:

Reviewer #1

(Remarks to the Author)
In the revised manuscript, the authors have modified the main claims and the title, now proposing that prenatal exposure to particulate matter (PM) promotes epigenetic alterations associated with FCER1G expression in placental macrophages, which may subsequently lead to the re-emergence of FCER1Ghi M2 macrophages in the skin, contributing to the development of atopic dermatitis (AD). The authors have strengthened their argument by demonstrating epigenetic changes at the FCER1G locus in both PBMCs from AD patients and in PM-exposed placental tissues. Additionally, the inclusion of a mendelian randomization analysis provides further, albeit indirect, support for an association between these epigenetic alterations and AD development.
While these additions partially strengthen the proposed link between epigenetic changes and the increased frequency of FCER1Ghi M2 macrophages in AD, the causal relationship between prenatal PM exposure and the emergence of these M2 macrophages remains unclear. Specifically, Figures 1–3 support the notion that high levels of PM exposure may epigenetically regulate FCER1G expression. In contrast, Figures 4–7 focus primarily on the presence of FCER1Ghi M2 macrophages in AD lesional skin, without a clear connection to PM exposure levels. This shift in focus makes it difficult to trace the causal path from prenatal exposure to disease phenotype.
Moreover, the authors have not adequately addressed earlier concerns about the lack of experimental validation for their single-cell RNA-seq findings, which are based primarily on publicly available datasets. This reliance limits the robustness of their claims.
In addition to these general concerns, I would like to raise the following specific points:

1. Although the authors have revised some aspects of their "epigenetic scar" hypothesis, the term still appears in lines 609–610. While they demonstrate that similar epigenetic changes are observed in both PM-exposed placental tissues and PBMCs from AD patients, they do not provide evidence that these changes persist postnatally or are retained specifically in FCER1Ghi M2 macrophages. As such, the continued use of the term "epigenetic scar" is not supported by the data and should be revised.

2. In Fig. 2c and Extended Fig. 2h, the authors show FCER1G hypomethylation and increased chromatin accessibility in AD patients compared to healthy controls. However, these analyses were conducted across all AD patients regardless of PM exposure level. This is inconsistent with the claim that such epigenetic changes are characteristic of high PM exposure. The authors should clarify whether these results reflect PM-specific effects or general features of AD.

3. Figures 6i–j show that particulate matter can alter the phenotype of M2-like macrophages in vitro. However, if the central hypothesis is that PM exposure induces epigenetic modifications that persist and re-emerge in skin macrophages, then it would be important to demonstrate whether such epigenetic alterations also occur in these in vitro experiments. Without this validation, the link between PM-induced epigenetic changes and the observed macrophage phenotype remains speculative.

4. As previously noted, the authors do not provide experimental validation for key findings derived from scRNA-seq analyses. While prior studies may support increased M2 macrophages in AD in general, these study does not offer direct evidence showing an increase in Hofbauer cells in high PM-exposed placentas or an increase in FCER1Ghi macrophages in the skin of AD patients specifically with high PM exposure. These validations are essential to substantiate the proposed causal link between prenatal PM exposure and macrophage-mediated inflammation in AD.

Reviewer #2

(Remarks to the Author)
The authors have sufficiently and extensively responded to the reviewers' comments, and have thus clarified previously unclear approaches and results of their study.

Reviewer #3

(Remarks to the Author)
Thank you for your thoughtful revisions and responses to my previous comments. The revisions have considerably enhanced the manuscript.

I have one remaining concern regarding the definition of “low exposure” for PM2.5. The cut-off used (<24.70 μg/m^3) is significantly higher than health-based standards set by the WHO, US EPA, and Korea. Thus, “low exposure” group may represent relatively high exposure compared to guidelines or other air pollution studies. I would recommend noting this limitation in the discussion, as well as the implications for the interpretation and generalizability of your findings.

Reviewer #4

(Remarks to the Author)
The authors have provided detailed point-by-point responses to my comments. However, I remain concerned about several important issues. Specifically, this study may have low statistical power and lacks a validation study. In the context of omics analyses, the absence of appropriate multiple-testing correction raises the likelihood that some reported associations may be due to chance.
1. MR analyses and IV selection: The Mendelian Randomization (MR) analyses were conducted in 24 samples, which raises concerns about the reliability of the findings. Moreover, the validity of MR analysis critically depends on the strength and appropriateness of the genetic variants used as instrumental variables (IVs). Could the authors clarify in detail how the SNPs were identified and selected as IVs for each CpG site in this study?
2. The authors stated in their response that, after adjustment for multiple testing, the association between first-trimester PM2.5 exposure and AD incidence was no longer statistically significant (P = 0.118), while the association for exposure across the entire pregnancy remained significant (P = 0.01). Given this result, why did the authors still choose to use first-trimester PM2.5 exposure in the omics analyses, rather than focusing on the exposure window that remained significant?
3. Confounding by year of measurement: The author mentioned in the response that both AD incidence and PM2.5 levels may vary by calendar year. Therefore, confounding by year of measurement remains a concern. At a minimum, the authors should illustrate how PM2.5 levels changed across years (e.g., with descriptive plots) and consider stratified analyses by year (e.g., grouping years into two or three categories) to assess whether the observed associations are robust to this potential confounding factor.

Version 2:

Reviewer comments:

Reviewer #1

(Remarks to the Author)
The authors have adequately addressed the major concerns raised previously. However, this reviewer has one remaining minor concern regarding the description in the Abstract.
As noted in Concern #1, the authors demonstrate that epigenetic alterations are observed in both PM-exposed placental tissues and PBMCs from patients with AD. However, the study does not provide direct evidence demonstrating the persistence of these alterations across developmental stages, nor their association with FCER1G^{hi} M2 macrophages in AD

skin. Rather, the persistence of epigenetic alterations appears to be inferred from the presence of *FCER1G*^{hi} macrophages in those tissues.
In lines 67–69, the statement that "*This epigenetic alteration was recapitulated across developmental stages in independent cohorts, as demonstrated through single-cell RNA sequencing of 404,169 cells from placenta, fetal, and adult skin samples*" may therefore be misleading. This wording could give readers the impression that the epigenetic alteration itself was directly observed in placenta as well as fetal and adult skin within this study, which is not supported by the presented data.
The authors should revise this description and similar statements throughout the manuscript, to more clearly distinguish between experimentally demonstrated findings and interpretations or predictions.

Reviewer #4

(Remarks to the Author)
The revised manuscript has been improved as expected. I still have one concern
The authors aim to investigate mechanisms linking prenatal PM2.5 exposure to AD by examining epigenetic alterations that are sustained and re-emerge across developmental stages. Their hypothesis is that prenatal PM2.5 exposure induces specific epigenetic modifications, primarily hypomethylation of immune-related genes in certain placental immune cell types. However, the analytical approach does not directly test this hypothesis. Rather than first identifying epigenetic modifications that are significantly associated with PM2.5 exposure, the authors excluded differentially methylated CpGs (DMCs) and differentially expressed genes (DEGs) overlapping with the low-exposure group, thereby focusing on epigenetic signatures associated with AD only among children with high PM2.5 exposure. While this strategy identifies epigenetic markers associated with AD in the context of high exposure, it does not provide direct evidence that these markers are induced by PM2.5 exposure itself. Consequently, the current analyses do not fully support the interpretation that PM2.5 exposure induces the observed epigenetic modifications; rather, they suggest that the associations between epigenetic modifications and AD development may be modified by PM2.5 exposure, with such associations observed only among children with high exposure. I would suggest that the author add a table or figure indicating the associations of the identified primary signature (DMC and DEG) with PM2.5 (which can be treated as continuous or binary variables) in figure 1 using all samples (n=986), to support that these reported signatures are "induced" by high PM2.5 exposure.

Version 3:

Reviewer comments:

Reviewer #1

(Remarks to the Author)
The authors have adequately revised the manuscript to address previously misleading statements. This reviewer has no further concerns and supports its publication in this journal.

Reviewer #4

(Remarks to the Author)
The authors have answered my questions. I have no further comments

Open Access This Peer Review File is licensed under a Creative Commons Attribution 4.0 International License, which permits use, sharing, adaptation, distribution and reproduction in any medium or format, as long as you give appropriate credit to the original author(s) and the source, provide a link to the Creative Commons license, and indicate if changes were made.
In cases where reviewers are anonymous, credit should be given to 'Anonymous Referee' and the source.
The images or other third party material in this Peer Review File are included in the article's Creative Commons license, unless indicated otherwise in a credit line to the material. If material is not included in the article's Creative Commons license and your intended use is not permitted by statutory regulation or exceeds the permitted use, you will need to obtain permission directly from the copyright holder.
To view a copy of this license, visit https://creativecommons.org/licenses/by/4.0/

REVIEWER COMMENTS

Reviewer #1 (Remarks to the Author):

In this manuscript, the authors aim to assess the effects of prenatal exposure to particulate matter (PM) on the development of pediatric atopic dermatitis (AD). They conducted DNA methylome and transcriptome analyses of placental tissue and found that placentae exposed to high levels of PM exhibited hypomethylation in several regulatory regions, potentially leading to overexpression of the FCER1G gene. Re-analysis of publicly available transcriptomic data showed that FCER1G is predominantly expressed in fetal macrophage-like Hofbauer cells. Given that the expression profile of placental Hofbauer cells is similar to that of M2 macrophages in the skin, the authors hypothesize that FCER1Ghi M2 macrophages may persist from the prenatal period and function as inflammation hubs, possibly through the induction of ROS signaling.
However, the evidence supporting the authors’ main hypothesis remains very weak, particularly in relation to the central claims presented in the manuscript’s title: "Prenatal particulate matter exposure imprints an epigenetic scar on FCER1G in fetal macrophages, reemerging in M2 macrophages to drive atopic dermatitis development." While the authors report hypomethylation of FCER1G in placentae exposed to PM, they provide no direct evidence that this so-called “epigenetic scar” persists into postnatal life, nor that it reappears in dermal M2 macrophages during the course of AD. The study does not demonstrate whether the epigenetic alterations observed in fetal Hofbauer cells are retained in the macrophages of pediatric or adult AD patients, especially in the skin. Thus, the proposed mechanistic link between prenatal PM exposure and epigenetic imprinting in macropahges remains largely speculative. Below are specific concerns:

Thank you for the reviewer’s valuable comments for the weakness points of our manuscript. As suggested by the reviewer, we tone down of the concept “epigenetic scar” in the title and manuscript as “epigenetic alteration” and revised the title as "Prenatal particulate matter exposure induces an epigenetic alteration in FCER1G in fetal macrophages, influencing M2 macrophage function in atopic dermatitis development."

Sorry for lack of epigenomics longitudinal data and single-cell epigenome data across development stages associated with AD status due to too challenging to sampling, we gave an effort to integrate partial evidence of epigenetic alteration in the regulatory region of FCER1G was recapitulated across AD developmental stages. Specifically, we performed analyses for novel data and added functional experimental evidences to confirm the active epigenetic regulation of FCER1G associated AD development in both PBMCs and skin level as described in the response of Major and Minor Concerns.

To strengthen the causal relationship between FCER1G epigenetic alteration and AD development, we performed a Mendelian randomization (MR) analysis in our cohort, as detailed in the response to Comment #17 of Reviewer 3 and Comment #9 of Reviewer 4. We specifically evaluated two

regulatory CpG loci (cg05656486 and cg20806175) previously identified as hypomethylated under high PM2.5 exposure. The MR analysis demonstrated that reduced methylation at both loci was significantly associated with increased AD risk, and this association remained robust after adjusting for key covariates, including PM2.5 exposure levels. These results support a potential causal role of PM2.5-induced hypomethylation in AD pathogenesis.

We further established the clinical relevance of FCER1G$^+$ M2 macrophages by linking their signature to SCORAD, a validated index of AD severity. Enrichment scores were significantly higher in severe cases, supporting their association with disease progression. To elucidate functional consequences, we analyzed ROS pathway activity, observing strong correlations between FCER1G module expression and ROS signaling across developmental stages and skin lesions. Additionally, CCL13, a key Th2-associated chemokine, was progressively upregulated in FCER1G$^+$ M2 macrophages and spatially colocalized with ACKR1-expressing VE3 endothelial cells, forming a localized immunological axis. These findings collectively support the role of FCER1G$^+$ M2 macrophages in promoting AD inflammation through ROS activation and CCL13–ACKR1-mediated interactions.

We attach a detailed point-by-point response to all reviewer comments. We would be grateful if you would consider allowing us for re-evaluation.

Major Concerns
Major Concern #1:
Although the authors suggest that the “epigenetic scar” observed in placental samples persists in adult AD skin lesions, they did not directly assess the epigenetic profiles of adult AD skin tissue. The authors should examine whether similar epigenetic alterations are present in dermal macrophages of AD patients.

We thank the reviewer for this important and constructive comment. We fully agree with the concern regarding the inference of a persistent “epigenetic scar” without direct methylation analysis in adult skin tissue. In response, we have revised the manuscript title and conclusions to replace the term “epigenetic scar” with “epigenetic alteration” to avoid a definitive causal implication. We have also explicitly acknowledged in the Discussion the limitation that our study lacks longitudinal or tissue-matched epigenomic data across developmental stages.

To address this limitation, we performed several complementary analyses that provide supporting evidence for the presence of similar FCER1G epigenetic alterations in AD-associated dermal macrophages:

As shown in Fig. 2c, using an independent pediatric cohort, we confirmed that FCER1G hypomethylation occurs in PBMCs from AD patients compared to healthy controls at the same loci identified in placental tissues. Correspondingly, FCER1G expression was progressively elevated in AD skin lesions across infancy, childhood, and adulthood (Fig. 2d). Furthermore, this

expression significantly correlated with the inferred density of macrophages in AD skins across developmental stages compared to other cell types, suggesting a functionally relevant link between epigenetic regulation and macrophage activation in disease context. This trend was recapitulated in our cohort (Fig. 3F).

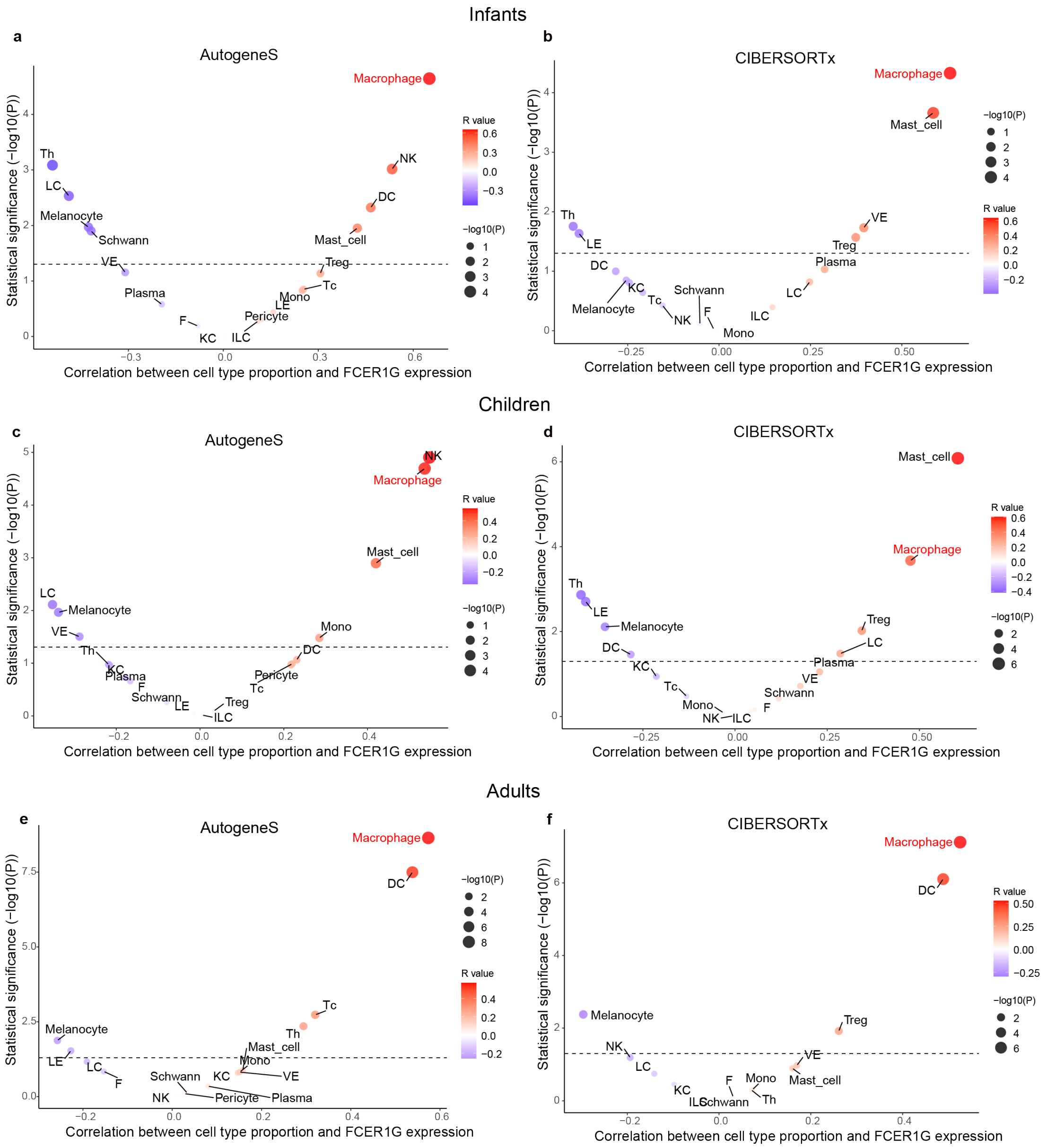

Extended Data Fig. 2a–2f. Correlation between FCER1G expression and macrophage proportion across healthy and AD skin samples. Correlation plots between FCER1G expression and macrophage proportion across healthy and AD skin samples. Cell type proportions were inferred from skin transcriptomes using AutogeneS (**a, c, e**) and CIBERSORTx (**b, d, f**). Panels **a** and **b** represent infant samples, **c** and **d** represent children, and **e** and **f** represent adults. The dashed line along the y-axis indicates $-\log_{10}(P = 0.05)$. Statistical analyses include Spearman correlation.

We also refer to the study by Liang et al. (PMID: 22150093), which demonstrated FCER1G promoter hypomethylation in monocytes of adult AD patients, inversely correlated with gene expression. Consistent with this, our analysis of publicly available ATAC-seq data (GSE255396) revealed increased chromatin accessibility at FCER1G regulatory regions in AD monocytes compared to controls. Given that monocytes are precursors of skin macrophages, these findings support the relevance of systemic FCER1G epigenetic alterations in tissue-resident macrophage populations.

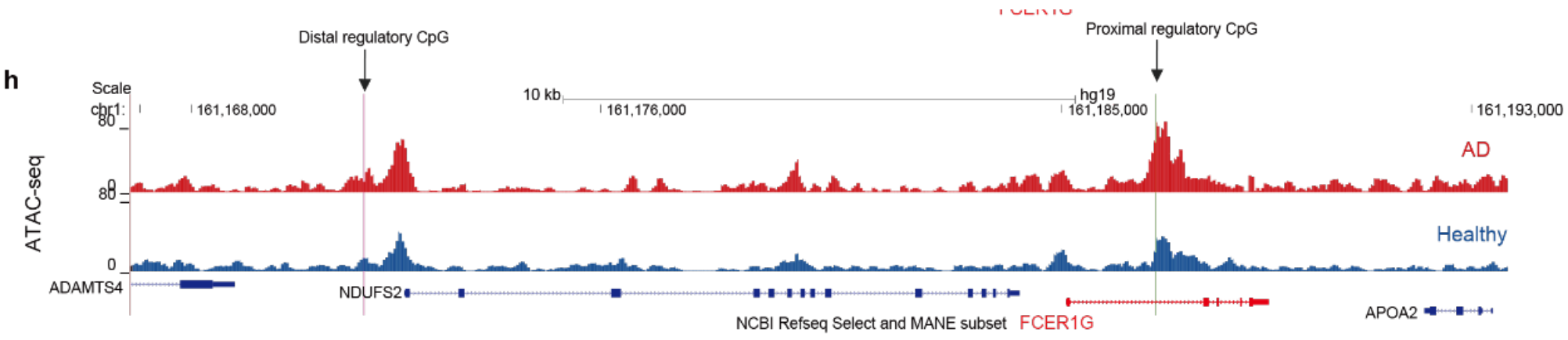

Extended Data Fig. 2h. Increased chromatin accessibility at FCER1G regulatory regions in PBMC-derived monocytes from AD patients. Average chromatin accessibility profiles of PBMC-derived monocytes from AD patients (n = 6, red) and healthy (n = 4, blue) based on ATAC-seq data (GSE255396). Green and purple marks indicate the proximal and distal regulatory CpG sites, respectively.

Although direct methylation profiling in skin macrophages remains technically challenging, these results collectively reinforce the functional significance and probable persistence of FCER1G epigenetic dysregulation in the myeloid lineage of AD patients.

We have incorporated these findings in the revised manuscript and clarified their implications in the Results and Discussion sections.

Line 261-: To determine cell-type specificity, digital cytometry inference[21,22] of our transcriptome data indicated increased macrophage and NK cell enrichment score in the placentae of offspring with AD exposed to high $PM_{2.5}$, with macrophages showing a significant association with FCER1G expression and signaling activation (Figs. 3e and 3f). This positive correlation between FCER1G expression and macrophage enrichment was consistently observed across developmental stages—including infants, children, and adults—using independent skin transcriptome datasets from healthy controls and AD patients[18,19] (Extended Data Fig. 2a-2f).

Line 268-: Additionally, epigenomic analysis confirmed high regulatory potential specific to macrophages, as loci of epigenetic disruption, both proximal and distal to FCER1G, exhibited the strongest DNase-seq signals in macrophages, compared to other immune cells (Fig. 3g and Extended Data Fig. 2g). Furthermore, our analysis of public ATAC-seq data (GEO accession GSE255396) demonstrated increased chromatin accessibility at these FCER1G regulatory regions in PBMC monocytes from AD patients (Extended Data Fig. 2h). Supporting our findings, Liang et al.[23] reported hypomethylation of the FCER1G promoter in peripheral monocytes from adult AD patients, which was inversely correlated with gene expression. As monocytes are precursors of tissue-resident macrophages, these results suggest that

epigenetic alterations in circulating myeloid cells may be transmitted to skin macrophages. This supports the relevance of FCER1G dysregulation in dermal macrophage-mediated inflammation and reinforces its role in triggering allergy-related pathways, particularly in placental macrophages of offspring with AD.

Line 691-: Collection of large quantities of placental tissue is challenging, and comprehensive profiling methylome and transcriptome profiling requires highly specialized tissue processing. Additionally, we acknowledge that we did not directly assess the epigenetic profiles of dermal macrophages in adult AD skin tissue, which limits our ability to fully validate the persistence of placental epigenetic alterations in affected skin. To mitigate these constraints, we leveraged Mendelian randomization analysis to validate the causal relationship between $PM_{2.5}$-associated methylation loci and AD status, incorporating sex as a covariate. Furthermore, we supplemented our findings with independent datasets spanning multiple developmental stages and disease contexts of AD. These included DNA methylome, chromatin accessibility, and transcriptome data, encompassing both bulk tissues and single-cell resolution, to reinforce the robustness and biological relevance of our conclusions.

Major Concern #2:
A large portion of the study relies on publicly available data from AD patients. The authors must validate the inferences drawn from transcriptomic analysis. For example, in Figure 4c, the authors predicted an increased frequency of Hofbauer cells in high PM-exposed AD patients using deconvolution analysis of bulk RNA-seq data. Similarly, they predicted an increase in FCER1Ghi M2 macrophages in AD skin lesions under high PM exposure. These findings should be validated through experimental methods such as immunostaining.

We sincerely appreciate the reviewer's insightful comment and fully agree with the importance of experimentally validating transcriptomic inferences. As the reviewer correctly points out, Figure 4 demonstrates an increased abundance of Hofbauer cells with M2-like features in high PM-exposed AD samples, as inferred through deconvolution of bulk RNA-seq data. While this result is based on computational analysis, it is supported by previous cell-type–specific experimental studies. For instance, Reynolds et al. (PMID: 33479125) demonstrated that M2 macrophages are enriched in the skin of AD patients compared to healthy controls, using immunohistochemistry targeting the F13A protein — a known M2 macrophage marker that also overlaps with the $FCER1G^{+}$ M2 macrophage signature we identified. We now highlight this supporting evidence in the revised Result section to provide context for our computational findings.

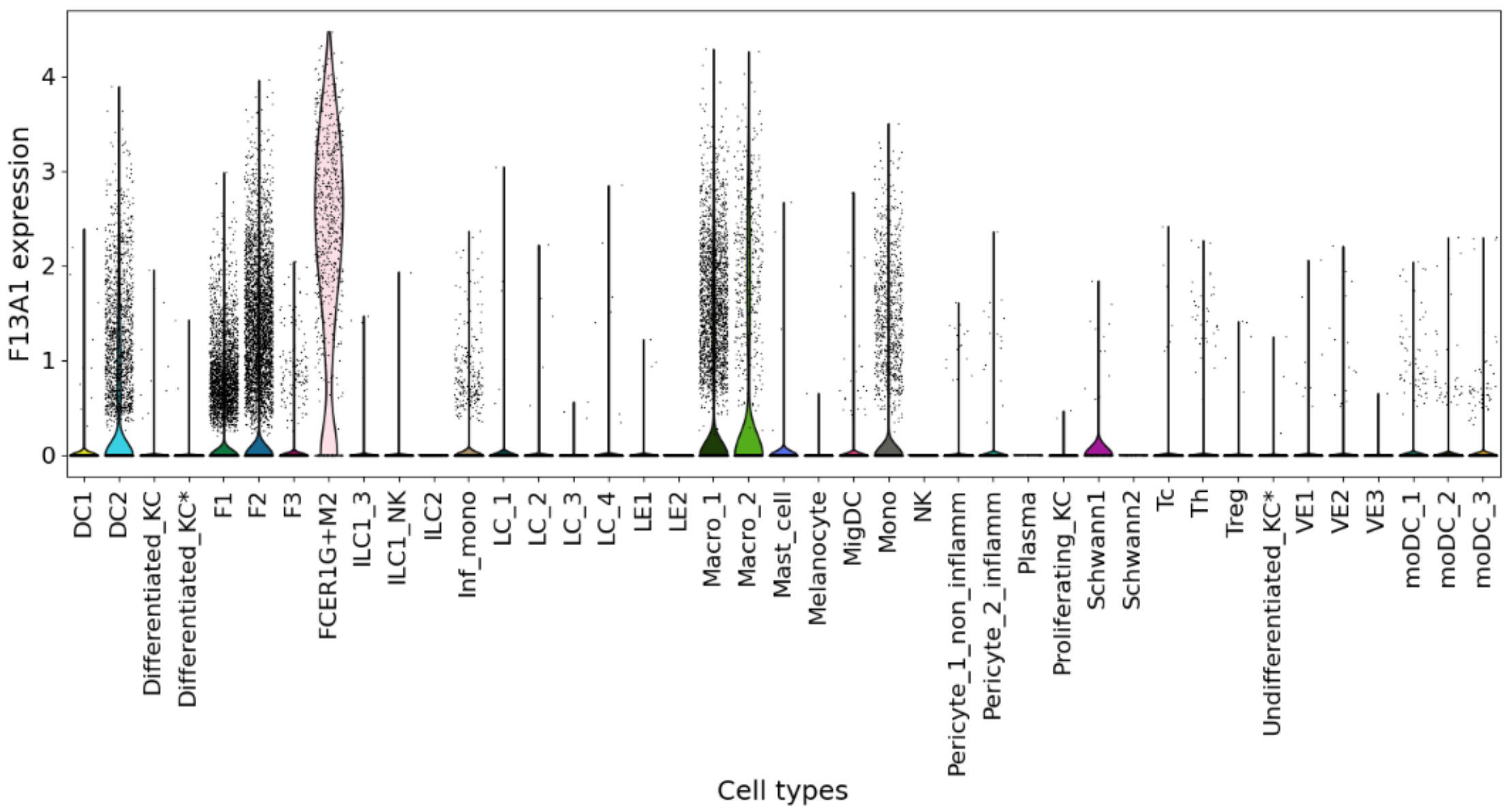

F13A1 expression is specifically enriched in FCER1G⁺ M2 macrophages. Violin plot showing single-cell expression levels of F13A1 across annotated cell types in AD skin. F13A1 is highly expressed in the FCER1G⁺ M2 macrophage cluster, but not in other immune or stromal cell populations, supporting its role as a specific marker for this macrophage subpopulation.

To further complement our transcriptomic predictions, we present converging evidence from human, murine, and in vitro experimental studies:

- In human AD skin: He et al. (PMID: 32709423) analyzed tape-stripped skin from AD patients and reported elevated expression of FCER1G, CYBB, and MS4A4A specifically in lesional areas. These genes are core components of the FCER1G⁺ M2 macrophage module we identified, providing further experimental validation of our predicted macrophage population under AD conditions. In another study, Behr et al. (PMID: 40442215) reported that CD163⁺ and CD206⁺ M2-like macrophages were highly abundant in AD patient skin, comprising over 90% of CD14⁺ myeloid cells, based on quantitative analysis using high-content multiplex immunohistochemistry (MELC) for single-cell classification. CD163 and CD206 were also among the overexpressed genes in the FCER1G⁺ M2 macrophages identified in our analysis.
- In vivo: Kim et al. (PMID: 32846909) showed that PM10 exposure aggravates skin inflammation in an OVA-induced AD mouse model. Notably, FCER1G expression was significantly increased in the skin of PM10-exposed mice, directly supporting our finding that PM exposure enhances FCER1G⁺ macrophage activity.
- In vitro: We experimentally confirmed that FCER1G and other FCER1G⁺ M2–specific markers (CYBB, MRC1, MS4A4A) are significantly upregulated in macrophages exposed to both urban and diesel PM2.5, compared to unexposed controls. These results confirm that particulate matter exposure alone is sufficient to induce the FCER1G⁺ M2 macrophage gene program, as predicted by our transcriptomic analysis. A schematic summarizing this experimental validation is shown in Fig. 6i.

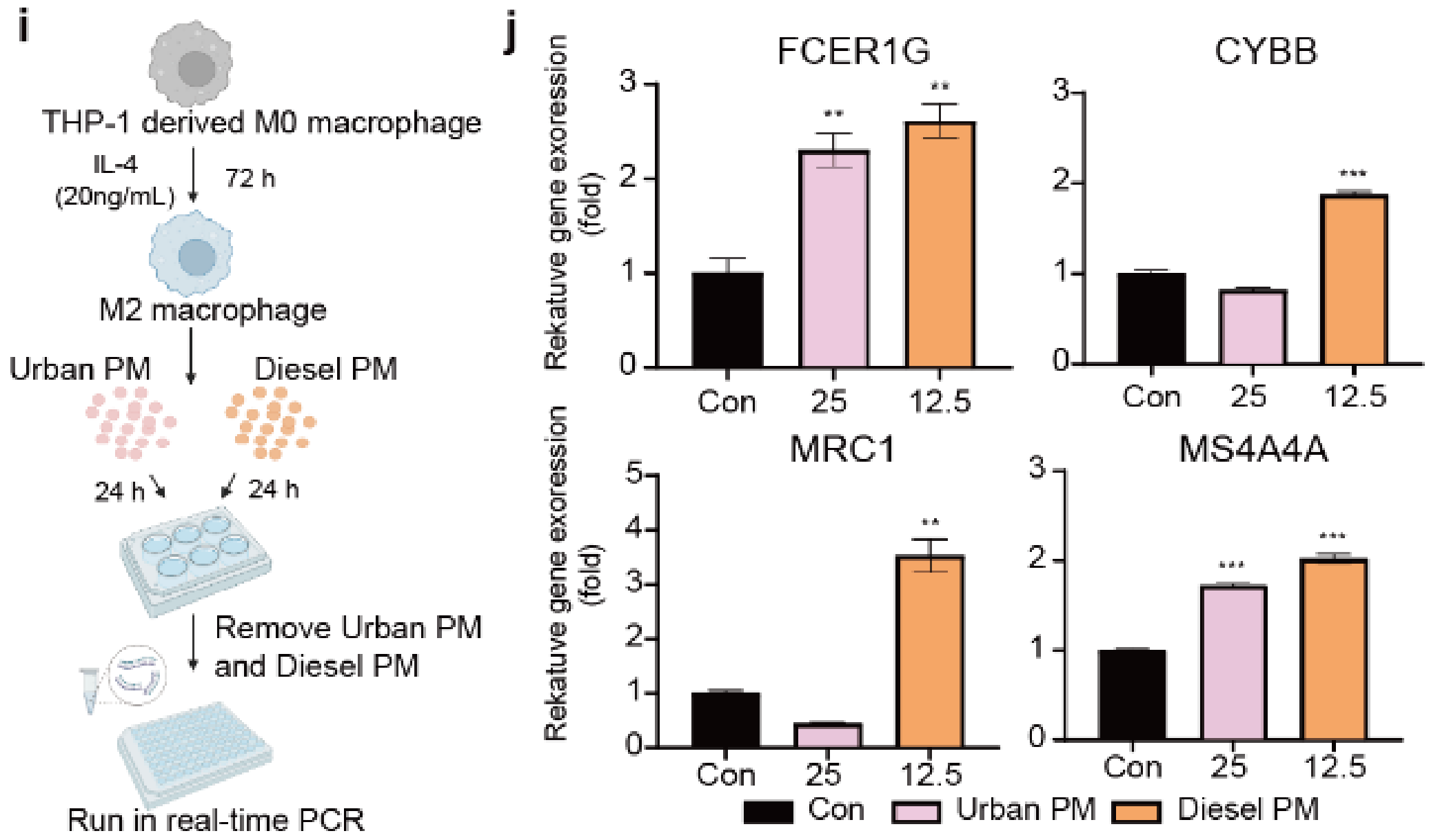

Taken together, these independent human, murine, and in vitro findings collectively reinforce our interpretation that FCER1G^{+} M2 macrophage enrichment is a biologically relevant phenomenon in AD and can be induced by PM exposure. These supporting data have been explicitly integrated into the revised Results sections and are illustrated with an experimental design schematic.

We acknowledge that direct immunostaining of FCER1G in human lesional skin under PM exposure conditions would provide further definitive evidence. However, we kindly ask for the reviewer's understanding that the availability of human skin samples used in this study is extremely limited, and the current conditions do not permit additional histological analyses. We have noted this as a limitation and a direction for future studies in the revised manuscript.

Line 333-: At the gene regulatory level, co-expression network analysis revealed a conserved gene module, including FCER1G, that was overexpressed across developmental stages in placental macrophages, fetal skin macrophages, and adult M2 macrophages (Fig. 4d). Notably, several genes within this conserved module, including CD163, F13A1, and MRC1, have been experimentally validated to be overexpressed in AD skin lesions through immunohistochemistry[28,29]. This module, designated "module A," was selectively activated in the placentae of offspring with AD displaying an epigenetic alteration, but remained inactive in those without such epigenetic alterations (Fig. 4e and 4f).

Line 471-: Among the genes directly interacting with FCER1G, CD68, CYBB, MRC1, and MS4A4A were identified as key players involved in NADPH oxidase-mediated ROS signaling, a pathway implicated in macrophage-driven inflammatory responses and increased risk of AD[33–35] (Fig. 6h, left). Consistently, elevated expression of FCER1G, CYBB, and MS4A4A has been reported in tape-stripped atopic dermatitis lesional skin[36]. Single-cell trajectory analyses further demonstrated a progressive upregulation of these genes in M2 macrophages transitioning from

healthy to AD states, confirmed by both gene expression and splicing-based status inference analyses (Fig. 6h, right).

Line 487-: Additionally, FCER1G-associated signaling molecules, such as CYBB, MRC1, and MS4A4A, were significantly overexpressed under diesel $PM_{2.5}$ exposure. Experimental evidence from PM_{10} exposure in an OVA-induced atopic dermatitis mouse model further supports these in vitro findings, demonstrating significantly increased FCER1G expression in the skin of PM10-exposed mice[37] . Collectively, these results underscore FCER1G as a critical regulator within M2 macrophages, driving aberrant ROS signaling and promoting AD progression across developmental stages.

Major Concern #3:
While FCER1Ghi dermal macrophages appear to be more frequent in AD patients than in healthy controls, their functional role in skin allergic inflammation remains unclear. Is the increased frequency of FCER1Ghi dermal macrophages associated with AD severity or type 2 inflammatory markers such as IL-4 and IL-13?

Thank you to remind us the crucial aspect to define the functional role in skin AD of our proposed mechanism. In the cellular composition inference, FCER1G+ M2 macrophages are only increased from healthy to AD condition (Figure 5c-f), in addition to increasing from non-lesional AD to lesional AD (Figure 5g) as their clinical relevance of association with AD severity. To further elucidate their link to clinical severity, we stratified lesional AD patients based on SCORAD, a validated clinical index of AD severity. FCER1G$^+$ M2 macrophage enrichment scores were significantly higher in patients with severe AD than in those with mild to moderate disease, indicating a positive correlation between FCER1G$^+$ M2 macrophage density and AD severity. These findings support the contribution of FCER1G$^+$ M2 macrophages to the pathophysiology and clinical progression of AD.

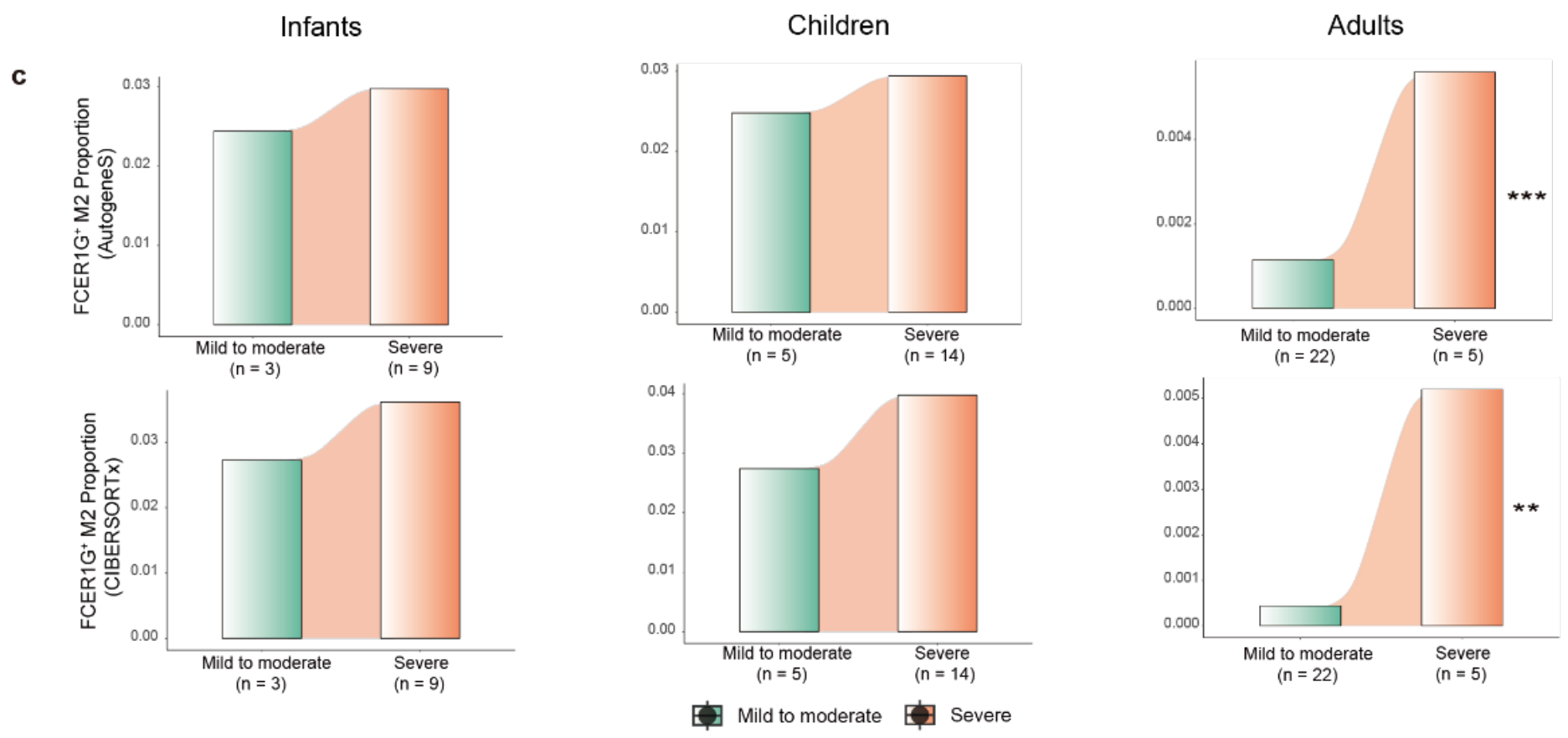

Extended Data Fig. 5c. SCORAD-based comparison of FCER1G$^+$ M2 macrophage cell proportions in AD skin samples.

Proportion of FCER1G⁺ M2 cell types across developmental stages in AD lesional samples, stratified into mild-to-moderate and severe groups based on SCORAD scores (cutoff = 50 for infants and children, 40 for adults), estimated from bulk transcriptomes using AutogeneS (top) and CIBERSORTx (bottom). Statistical analysis includes likelihood ratio test.

Building on our proposed mechanism implicating ROS signaling as a functional mediator of FCER1G+ M2 macrophage-driven AD severity, we observed a progressive enrichment of key ROS pathway components in lesional skin relative to healthy controls (Fig. 7). Spatial transcriptomic analysis revealed a stepwise increase in FCER1G+ M2 macrophage abundance and ROS-associated gene expression scores from healthy to non-lesional and lesional AD regions, coinciding with areas of leukocyte infiltration. This pattern was conserved across developmental stages, with FCER1G-associated gene module scores markedly elevated in lesional AD skin compared to matched non-lesional samples. Moreover, NADPH oxidase–mediated ROS signaling activity exhibited a parallel trajectory. Correlation analysis confirmed a robust positive association between FCER1G module expression and ROS signaling within individual skin samples across all developmental stages, supporting the spatial and functional convergence of these signatures in AD lesions.

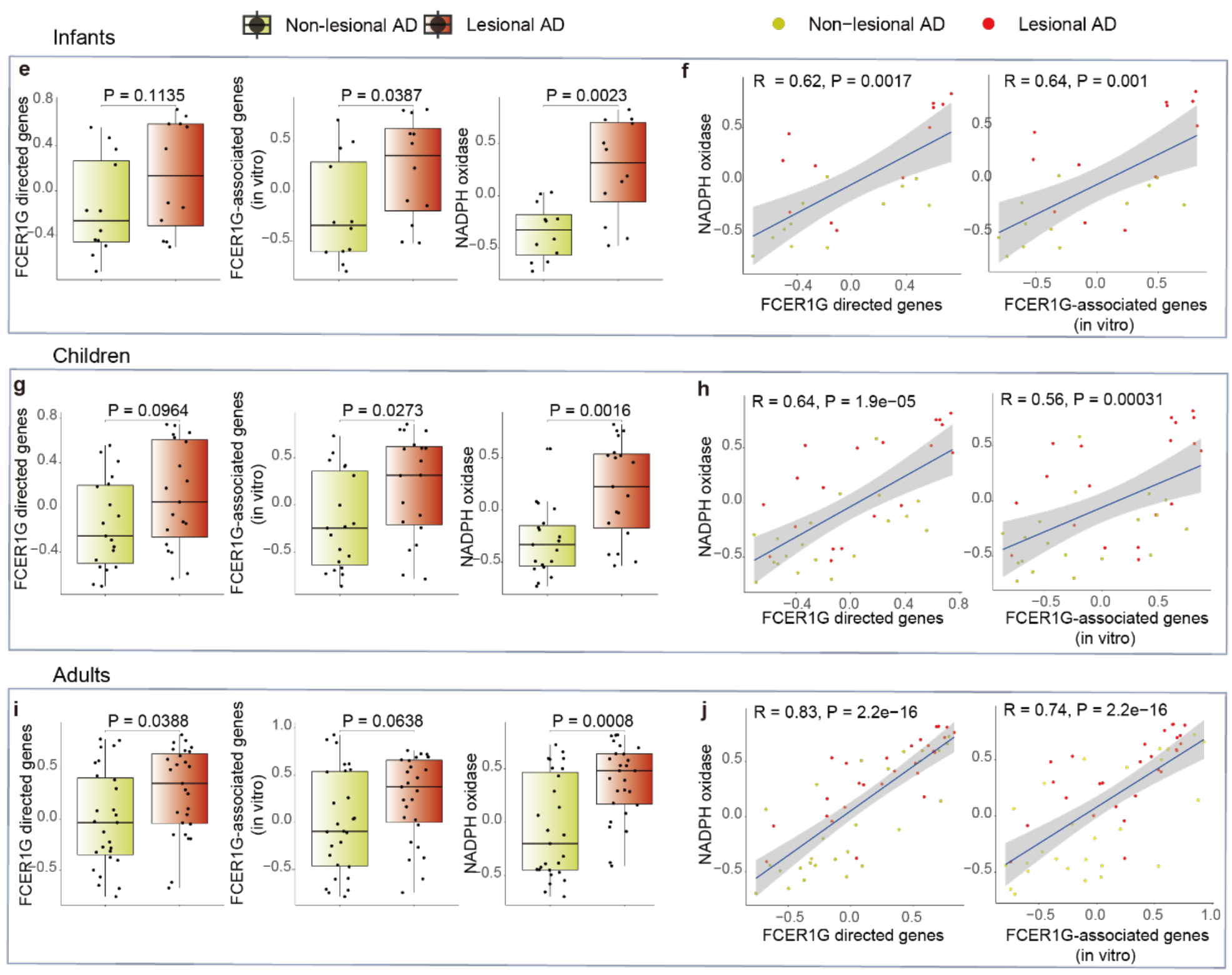

Extended Data Fig. 9e-i . Increased FCER1G⁺ M2 macrophage signature and NADPH oxidase scores in AD skin across developmental stages.
e, g, i, Box plots represent score comparisons between lesional and non-lesional AD skin across developmental stages: infants (n = 12 per group), children (n = 19 per group), and adults (n = 27 per group). FCER1G-directed gene scores (left) were derived from the network in Fig. 6f. In vitro–identified

FCER1G-associated gene scores (middle) from Fig. 6j, NADPH oxidase complex gene scores (right) calculated using the expression of CYBA, CYBB, NCF1, NCF2, NCF4, RAC1, and RAC2. **f, h, j**, Correlation plots illustrate the association between each FCER1G-related score and NADPH oxidase score. Red dots represent AD lesional samples and yellow dots represent non-lesional samples. Statistical analyses include Wilcoxon rank-sum test (**e, g, i**) and Spearman correlation (**f, h, j**).

To assess the functional implications of FCER1G+ M2 macrophages in type 2 inflammation, as recommended by the reviewer, we examined the expression of key Th2-associated interleukins and chemokines. Notably, CCL13, previously identified as central immunogenic drivers in AD pathogenesis [PMID: 37153575 and PMID: 22951056], was progressively upregulated in FCER1G+ M2 macrophages from healthy to non-lesional and lesional AD skin. This expression trajectory mirrors its reported positive correlation with SCORAD, an established clinical measure of AD severity [PMID: 27825969; PMID: 22951056]. In parallel, previous reports have implicated M2 macrophages in functional interactions with vascular endothelial (VE) cells in AD [PMID: 33479125]. Leveraging spatial transcriptomics, we identified CCL13 as being specifically overexpressed in FCER1G+ M2 macrophages and spatially co-localized with its receptor ACKR1, which was enriched in VE3 cells, a subtype of VE cells. Importantly, ACKR1 expression in VE3 cells also increased progressively from healthy to lesional AD skin. These findings highlight a spatially coordinated immunological axis linking FCER1G+ M2 macrophages and VE3 cells via CCL13–ACKR1 signaling, supporting their role in AD lesion development and maintenance.

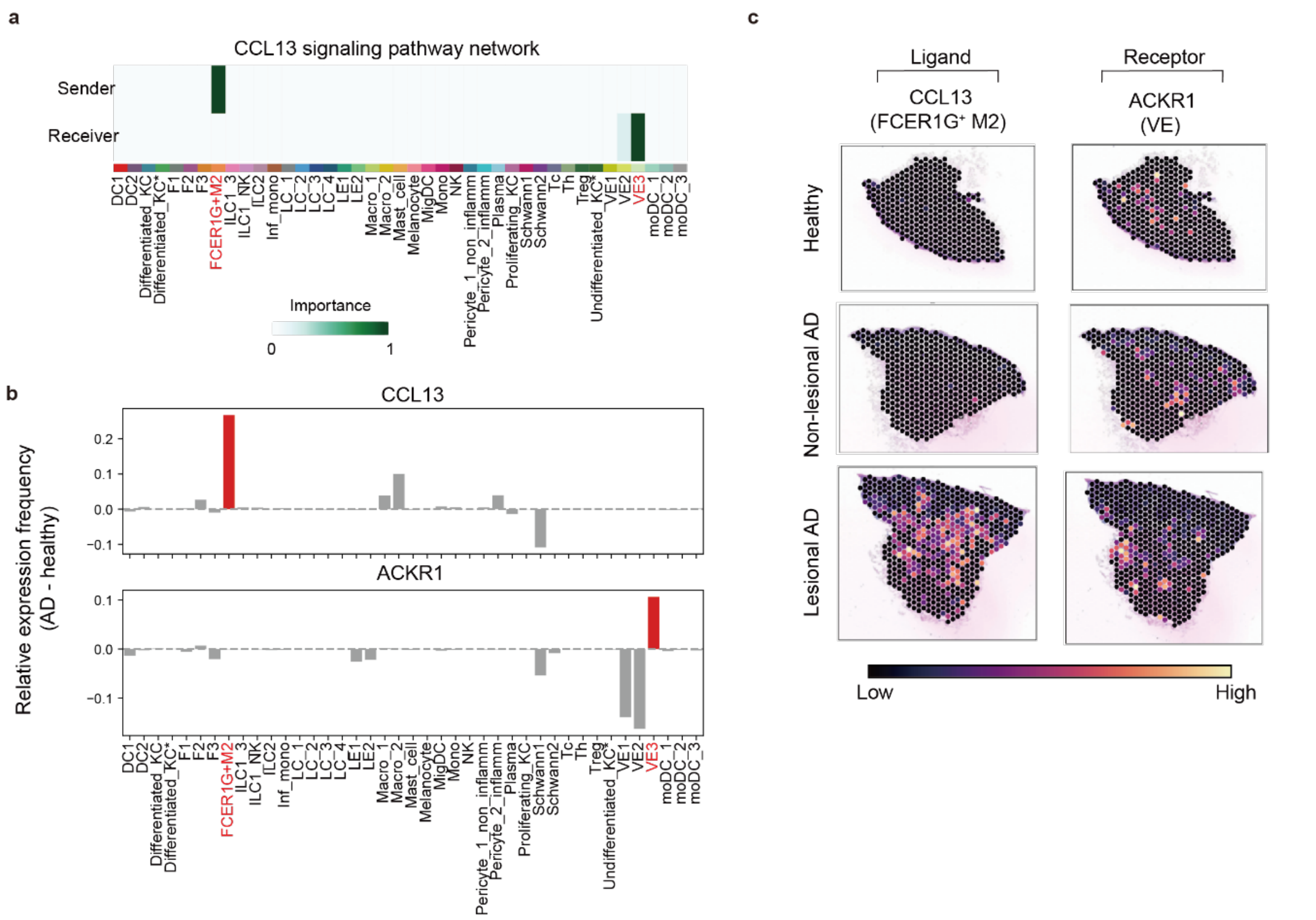

Extended Data Fig. 11. FCER1G⁺ M2 macrophages mediate cell–cell interactions as ligand-producing cells via CCL13 in AD skin.
a, Heatmaps showing the expression levels of ligands and their corresponding receptors across each cell type. b, bar plots represent the difference in the proportion of cells expressing CCL13 (top) and ACKR1 (bottom) in each cell type. c, Spatial expression maps of the most enriched sender and receiver cell types involved in the CCL13 interactions, across healthy, non-lesional AD, and lesional AD skin sections.

Corresponding updates have been incorporated into the revised Results and Discussion sections.

Line 71-: Network analysis and in vitro PM exposure experiments identified a mechanistic axis involving FCER1G, CD68, CYBB, MRC1, and MS4A4A, promoting NADPH oxidase–driven reactive oxygen species signaling. Additionally, CCL13 is upregulated in $FCER1G^+$ M2 macrophages and spatially colocalized with $ACKR1^+$ endothelial cells, suggesting a localized Th2 inflammatory circuit.

Line 401-: In contrast, FCER1G− M2 macrophages and M1 macrophages did not exhibit significant increases across developmental stages. To assess the association with clinical severity, we stratified lesional AD patients by SCORAD, a validated index of AD severity. $FCER1G^+$ M2 macrophage enrichment scores were markedly elevated in patients with severe AD compared to those with mild to moderate disease (Extended Data Fig. 5c), establishing a positive correlation between $FCER1G^+$ M2 macrophage abundance and disease severity. These findings suggest that the re-emergence of FCER1G+ M2 macrophages contributes to the development of AD at both cellular and developmental trajectory levels.

Line 475-: Single-cell trajectory analyses further demonstrated a progressive upregulation of these genes in M2 macrophages transitioning from healthy to AD states, confirmed by both gene expression and splicing-based status inference analyses (Fig. 6h, right). At the tissue level, gene set enrichment analysis demonstrated a marked elevation of FCER1G module scores in lesional AD skin relative to paired non-lesional samples (Extended Data Fig. 9e-j). Concordantly, NADPH oxidase–mediated ROS signaling exhibited a parallel activation trajectory. Correlation analysis across individual samples confirmed a strong positive association between FCER1G module activity and ROS signaling across all developmental stages. This cellular signaling cascade could be imprinted by early-stage PM exposure.

Line 559-: Further histopathological analysis confirmed a spatially coordinated increase in $FCER1G^+$ M2 macrophages and ROS signaling factors across different skin samples, demonstrating a consistent enrichment pattern from healthy to lesional AD skin (Fig. 7e). To explore their role in type 2 inflammation, we assessed the expression of Th2-associated chemokines. CCL13, previously implicated in AD severity[39,40], were progressively upregulated in FCER1G+ M2 macrophages along the healthy-to-lesional axis (Extended Data Fig. 11). This expression trajectory aligns with SCORAD severity correlations reported in prior studies[40,41]. Spatial co-

localization analysis revealed that both chemokines were enriched in FCER1G+ M2 macrophages and colocalized with their receptor, ACKR1, which was predominantly expressed in VE3 cells, a vascular endothelial subset. ACKR1 expression in VE3 cells also increased progressively with disease severity. These results establish a spatially coordinated interaction between FCER1G+ M2 macrophages and VE cells via the CCL13–ACKR1 axis, suggesting a mechanistic role in ROS-driven Th2 inflammation during AD progression.

Line 616-: Our findings underscore the potential role of epigenetically modified $FCER1G^+$ M2 macrophages in immune priming during early fetal development, contributing to an increased risk of AD in offspring via ROS activation. Beyond ROS activation, spatial transcriptomics revealed that $FCER1G^+$ M2 macrophages progressively upregulated the Th2-associated chemokine CCL13, which colocalized with ACKR1-expressing VE3 endothelial cells, establishing a localized immunological axis within AD lesions. Together, these findings highlight $FCER1G^+$ M2 macrophages as a critical cellular population linking prenatal PM2.5 exposure to AD pathogenesis through sustained epigenetic dysregulation, ROS-mediated signaling, and CCL13–ACKR1-driven Th2 inflammation.

Line 1035-: These signatures were then used to estimate the abundance of cell states in the spatial data. Cell-type-specific gene expression in the spatial data was estimated using the NCEM model[84]. We used CellChat[85] to identify the potential ligand–receptor interactions.

Minor Concerns

Minor Concern #1:

In most parts of the study, the authors compare AD patients and healthy subjects who were exposed to high levels of PM. However, given the study's objective of assessing the effects of PM exposure on AD development, it would be more appropriate to compare AD patients with high versus low PM exposure.

We thank the reviewer for this insightful comment. While comparing AD patients with high versus low PM exposure may elucidate differential effects within an established disease state, our study was designed to investigate how high PM exposure contributes to the development of AD. To this end, we focused on identifying AD-specific molecular signatures induced by high PM exposure by comparing PM^+AD^+ and PM^+AD^- groups. We hypothesized that high PM exposure generates widespread epigenetic alterations, a subset of which may promote AD pathogenesis, whereas AD cases under low PM exposure may be more influenced by genetic predisposition.

Consistent with this hypothesis, the number of differentially methylated and expressed features in PM^+AD^+ versus PM^+AD^- comparisons were over sixfold greater than in corresponding comparisons under low PM conditions. Moreover, the molecular signatures observed in the low PM setting did not overlap with those in the high PM context, reinforcing the specificity of high-PM-driven AD responses.

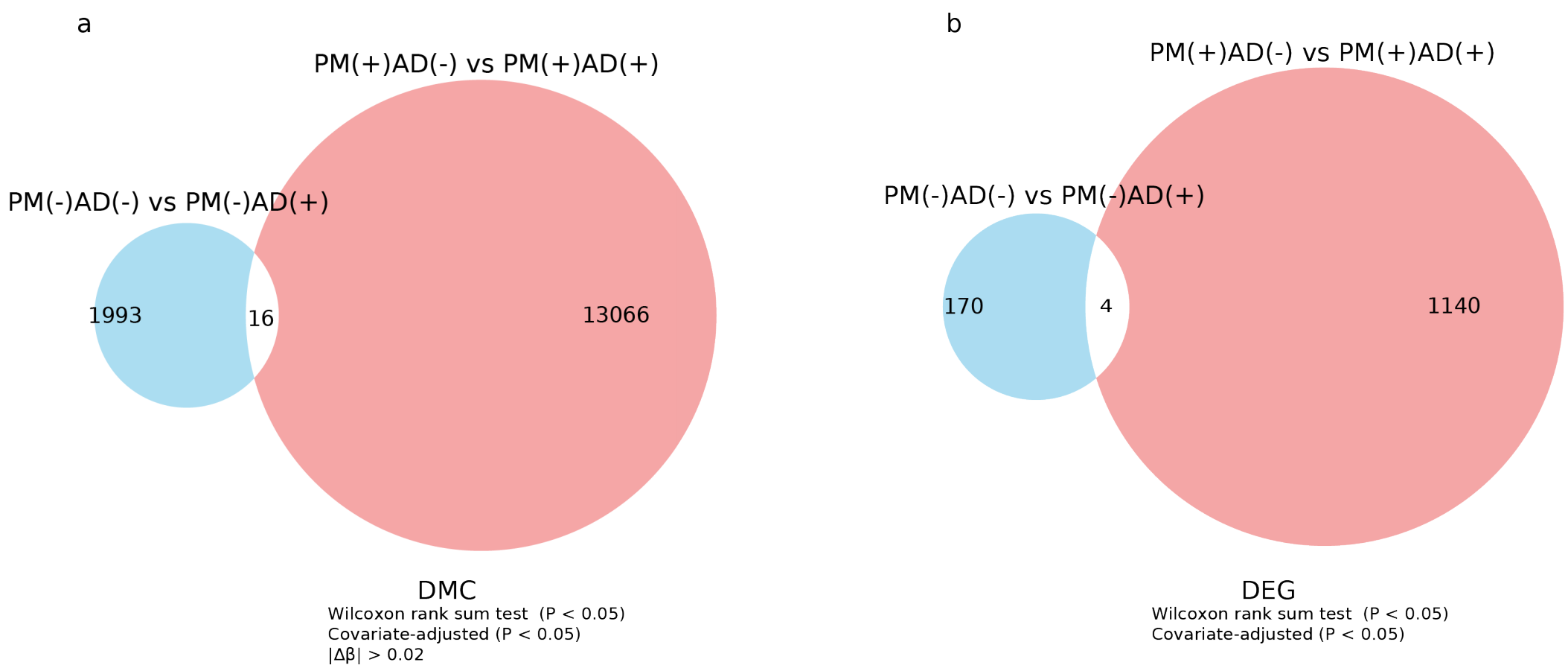

Venn diagram showing the number of differentially methylated CpGs (DMCs) and differentially expressed genes (DEGs) identified from comparisons between AD and healthy controls under low PM exposure ($PM^{-}AD^{-}$ vs $PM^{-}AD^{+}$) and high PM exposure ($PM^{+}AD^{-}$ vs $PM^{+}AD^{+}$).

Minor Concern #2:
Recent advances in single-cell RNA sequencing have revealed that the traditional M1/M2 macrophage classification oversimplifies the complexity of macrophage heterogeneity. The authors should consider using a more refined classification to characterize dermal macrophages in AD skin lesions.

We acknowledge the growing evidence from recent single-cell studies that challenge the binary M1/M2 macrophage paradigm and reveal a broader phenotypic continuum. To further contextualize the identity of FCER1G+ M2 macrophages, we integrated single-cell transcriptomic data from an independent AD cohort comprising 19 adult individuals, as reported by Fiskin et al. (PMID: 37961084). Within this dataset, FCER1G+ M2 macrophage signatures were projected onto the cell census and corresponded most closely with the C1QA+ macrophage subset, one of six macrophage subtypes annotated in that study. Notably, C1QA+ macrophages were enriched in lesional AD samples relative to healthy controls and characterized by the secretion of CCL13 chemokine that aligned with the functional phenotype of FCER1G+ M2 macrophages observed in our study.

We note, however, that the sample size in our current dataset (n = 9) limits the resolution of fine-grained macrophage subtypes at the individual level. While the total number of cells is substantial, this remains insufficient for atlas-scale classification across diverse individuals. Moreover, downstream analyses such as cell type deconvolution and spatial transcriptomics often benefit from more parsimonious classifications, as excessive granularity may dilute signal strength and reduce biological interpretability. Nonetheless, we concur with the reviewer that future studies leveraging larger cohorts with atlas-level depth will be essential for achieving a more refined and systematic delineation of dermal macrophage heterogeneity in AD.

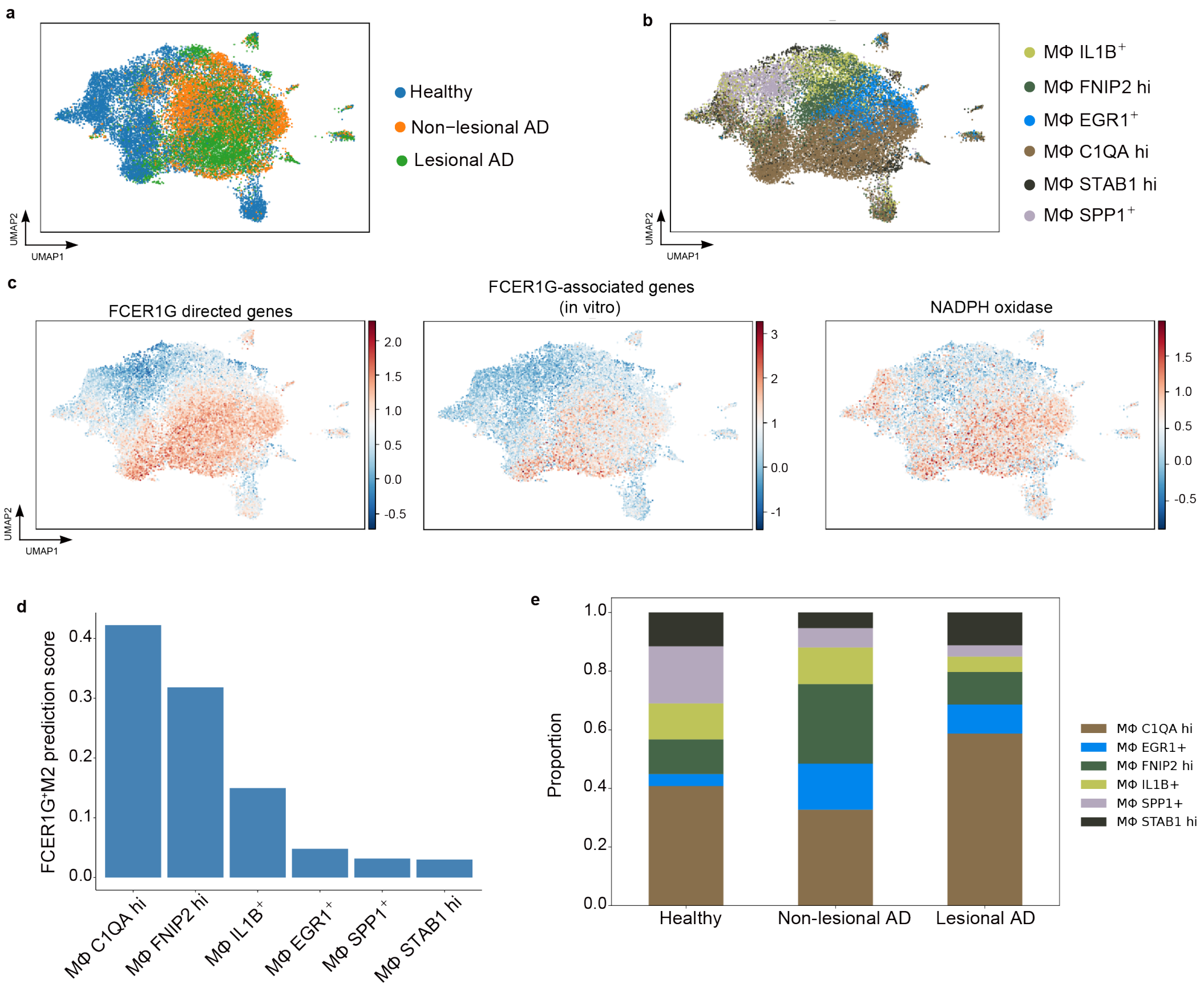

Identification of FCER1G⁺ M2 macrophages within the macrophage landscape defined in the AD skin atlas by Fiskin et al.

a, b, UMAP plots display the distribution of cells colored by atopic dermatitis status (a) and macrophage cell states as defined in the Fiskin et al. atlas (b).**c,** UMAP plots illustrate gene signature scores projected onto the macrophage landscape; FCER1G-directed gene scores derived from the network in Fig. 6f (left), in vitro–identified FCER1G-associated gene scores from Fig. 6i (middle), and NADPH oxidase complex gene scores calculated using the expression levels of CYBA, CYBB, NCF1, NCF2, NCF4, RAC1, and RAC2 (right). **d,** Bar plots display the mean prediction score of $FCER1G^{+}$ M2 macrophage, inferred using single cell data used in this manuscript and the macrophage states from Fiskin's atopic dermatitis atlas as reference datasets. The prediction scores were obtained using the TransferAnchors function in Seurat. **e,** Stacked bar plots represent the proportions of macrophage states across different atopic dermatitis conditions based on the dataset from Fiskin et al.

Reviewer #2 (Remarks to the Author):

The authors address a potentially very important, yet still insufficiently understood phenomenon of AD, namely prenatal imprinting of the immune system.

Major

The authors need to explain better what the nature of the particulate matter is. It remains unclear to the reader.

It remains unclear whether the increase in PM is rooted in a true causality, or a confounder. Please address better potential confounding factors. This remains unclear to the reader.

What is the chemical nature of PM?

#1. The nature of fine particulate matter ($PM_{2.5}$**):**
We thank the reviewer for their thoughtful feedback and valuable suggestions. We added the nature of $PM_{2.5}$ in the revised manuscript.

Line 85-: Accumulating evidence has implicated air pollution, specifically fine particulate matter ($PM_{2.5}$), in the onset and progression of AD[3]. $PM_{2.5}$ consists of both primary particles, which are directly emitted into the atmosphere, and secondary particles, formed through atmospheric chemical reactions involving precursor gases. Primary $PM_{2.5}$ originates from natural sources, such as dust storms and wildfires, as well as anthropogenic sources, including fossil fuel combustion, cigarette smoke, and biomass burning. Secondary $PM_{2.5}$ is generated via reactions between precursor gases emitted from both anthropogenic and biogenic sources. The composition of $PM_{2.5}$ is complex and heterogeneous, comprising black carbon, polycyclic aromatic hydrocarbons, aryl hydrocarbons, volatile organic compounds, heavy metals, inorganic ions, mineral dust, organic compounds, and biological materials.[4]

#2. Causality vs. Confounding factors:
Regarding the issue of causality versus confounding factors related to the increase in $PM_{2.5}$, we have addressed potential confounders, including sex, parental allergic history, maternal age, maternal education, maternal body mass index, secondhand smoke exposure during pregnancy and infancy, which may influence both $PM_{2.5}$ levels and health outcomes. Nevertheless, we acknowledge that residual confounding cannot be entirely excluded. Therefore, we emphasize the limitation of our study in the discussion and the need for future research employing longitudinal data and experimental designs to more thoroughly validate the causal links. While our epidemiological study in humans cannot control for all possible confounders, we would like to emphasize that, unlike animal model studies, our approach offers the advantage of capturing the real-world impact on human populations. This provides valuable insights into the actual exposure-response relationship in a natural setting.

Importantly, to strengthen causal inference, we conducted a one-sample Mendelian randomization (MR) analysis targeting two regulatory CpG loci (cg05656486 and cg20806175), which were hypomethylated under high PM2.5 exposure. This analysis revealed that hypomethylation at both loci was significantly associated with increased AD risk, independent of PM2.5 levels and other covariates. These findings suggest a potential mediating role of PM2.5-induced epigenetic alterations in disease etiology. We note, however, that the MR analysis was conducted on a limited sample size (n = 24), and future replication in larger cohorts is warranted. The corresponding results and interpretations have been incorporated into the revised manuscript.

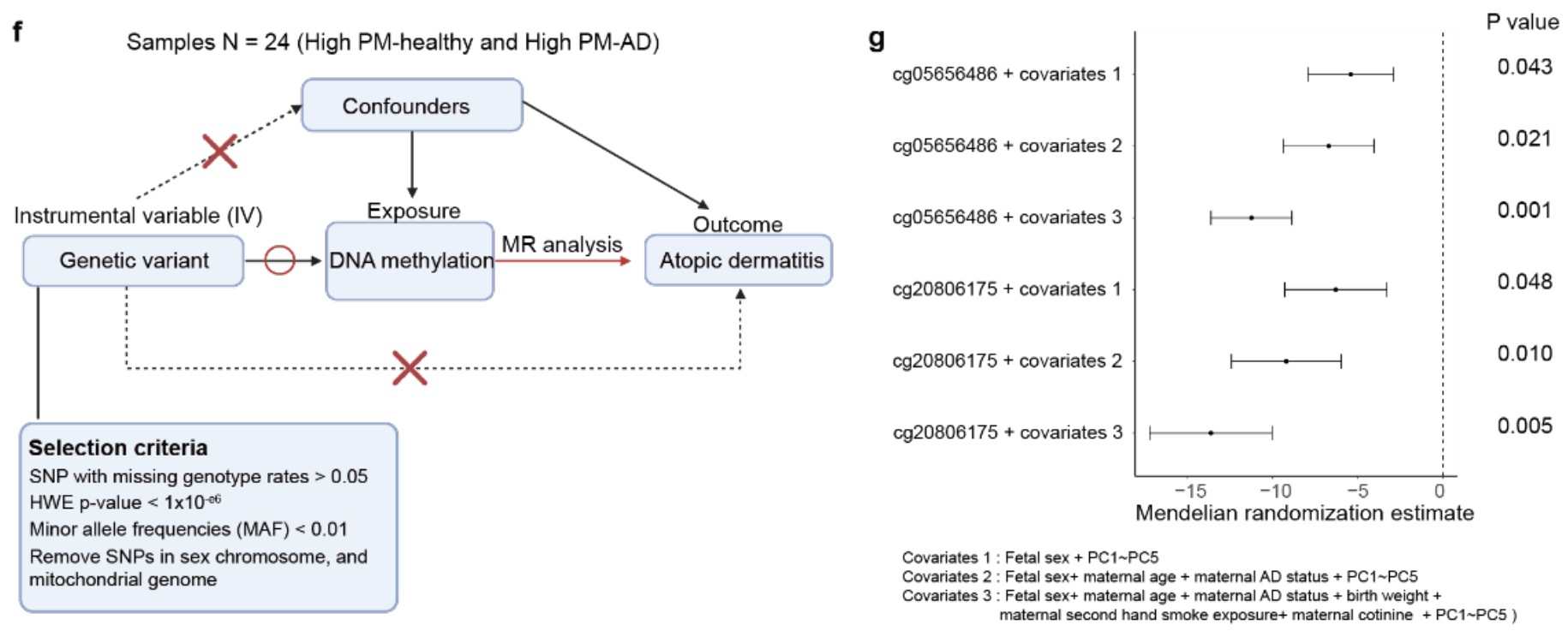

Extended Data Fig. 1f and 1g. Mendelian Randomization (MR) analysis to assess the causal role of DNA methylation in AD under high prenatal PM exposure. a, Schematic of the MR analytic pipeline. The analysis was performed using placental samples from the high prenatal PM exposure group in our cohort. b, Error bar plot showing the MR estimates for the CpG sites cg05656486 and cg20806175. P-values corresponding to each estimate are shown to the right of the plot. Covariate combinations included in the analysis are detailed below. The statistic was calculated using the ivreg function in R.

Line 201-: The regulatory CpG sites, located 13,693 bp upstream and within an intronic region of FCER1G, were located within the same topologically associated domain and overlapped with active histone marks (H3K27ac, H3K4me1, and H3K4me3) in placental tissue, but not in the maternal basal plate of the maternal side (Fig. 2e). To assess the potential causal role of these epigenetic modifications, we applied a one-sample Mendelian Randomization (MR) approach using 24 placental samples. This analysis revealed that hypomethylation at cg05656486 and cg20806175 was significantly associated with increased risk of infantile AD, suggesting a direct contribution of these loci to disease susceptibility (Extended Data Figs. 1f, 1g; Supplementary Table 5). Notably, these associations remained statistically robust after adjusting for PM2.5 exposure levels and other key covariates.

Line 695-: To mitigate these constraints, we leveraged Mendelian randomization analysis to validate the causal relationship between $PM_{2.5}$-associated methylation loci and AD status, incorporating sex, maternal age, maternal AD status, birth weight, maternal secondhand smoke exposure and maternal cotinine as a covariate.

Line 158-: $PM_{2.5}$ levels were categorized into tertiles (T1–T3) (n = 986), with T1 being the lowest and T3 being the highest. Models were adjusted for sex, parental allergic history, maternal age, maternal education, maternal body mass index, secondhand smoke exposure during pregnancy and infancy, and breastfeeding for six months.

Line 668-: Moreover, the use of a general population-based, prospective birth cohort study enhances the generalizability of our findings to the broader population[55,56]. Second, by focusing solely on $PM_{2.5}$, we cannot completely exclude the influence of other environmental factors on the development of AD, such as indoor pollutants, pollens, fungal spores, and meteorological conditions, which may confound or modify the effects of $PM_{2.5}$. Among these potential confounders, we adjusted for season of birth, as well as NO_2 and ozone concentrations, and the significant association between $PM_{2.5}$ and AD remained significant. Nevertheless, we acknowledge the complexity of air pollution exposure and highlight the need for future studies to apply refined multi-pollutant modeling approaches to better disentangle the interactive effects of various environmental factors on AD.

Minor

Please explain in abstract what PM2.5 in fact is.

We have revised the abstract to include a concise definition of $PM_{2.5}$. The updated abstract now reads as follows.

Line 59-: Prenatal environmental exposures may influence the development of atopic dermatitis (AD), yet the underlying mechanisms remain poorly understood. Fine particulate matter ($PM_{2.5}$), defined as airborne particles with an aerodynamic diameter ≤ 2.5 μm, comprises a heterogeneous mixture of metals, inorganic ions, organic compounds, and biological materials, and has been implicated in diverse environmental and health risks, including AD.

Reviewer #3 (Remarks to the Author):

This manuscript addresses the key research question of how prenatal exposure to PM2.5 may contribute to the development of atopic dermatitis (AD) in childhood. The authors used a multi-faceted analytical approach and multi-omics data to examine prenatal environmental exposures in the early-life risk of atopic dermatitis. Integrated omics approach and focusing both methylation and gene expression in relation to AD is novel and adds meaningful knowledge to the literature.

The authors perform a epidemiological analysis, examining a COCOA cohort of 986 children to assess whether first-trimester PM2.5 exposure is associated with a significantly increased risk of AD by age three. Furthermore, the study leverages comprehensive epigenomic and transcriptomic profiling of 48 placentas to investigate the biological mechanisms linking prenatal air pollution exposure to subsequent AD risk. They found an epigenetic scar involving FCER1G, which was hypomethylated and overexpressed in the placenta of children who later develop AD. The authors identified FCER1G^{+} M2 macrophages in the placenta as key cellular carriers of this epigenetic signature. These macrophages show enriched expression of genes involved in ROS signaling, a pathway associated with inflammatory responses and AD development. Their in vitro experiments showed that high $PM_{2.5}$ exposure may induce this molecular profile. Their findings suggest that prenatal air pollution may epigenetically program immune responses, increasing AD risk through ROS-activated M2 macrophages.

However, there are several major issues that need to be addressed before the manuscript can be considered for publication. Most importantly, the manuscript should clearly explain how PM2.5 exposure was dichotomized and provide the rationale for this classification. Without clarifying what is considered ‘low’ and ‘high’ exposure, it is difficult to interpret and draw conclusions from the findings.

We thank the reviewer for pointing out the need to clarify the classification of $PM_{2.5}$ exposure levels. In our epidemiological analysis of a cohort of 986 children, $PM_{2.5}$ exposure was assessed as a continuous variable as well as categorized based on the median, tertiles, quartile, and interquartile range (IQR). Across these approaches, higher $PM_{2.5}$ exposure was consistently associated with a statistically significant increase in the risk for AD incidence, as shown in the table below.

	a OR	95% CI		p value
PM2.5_ entire pregnancy (continuous)	1.04	1.01	1.06	0.01
PM2.5_ first trimester (continuous)	1.02	1.00	1.04	0.02
PM2.5_ second trimester (continuous)	1.02	1.00	1.04	0.04
PM2.5_ third trimester (continuous)	1.01	0.99	1.03	0.35
PM2.5_ entire pregnancy (median)	1.33	0.99	1.80	0.06
PM2.5_ first trimester (median)	1.31	0.97	1.76	0.07

PM2.5_ second trimester (median)	1.44	1.08	1.94	0.01
PM2.5_ third trimester (median)	1.10	0.82	1.48	0.53
PM2.5_ entire pregnancy_T1	1.00			
PM2.5_ entire pregnancy_T2	1.42	0.97	2.08	0.07
PM2.5_ entire pregnancy_T3	1.68	1.16	2.44	0.01
PM2.5_ first trimester_T1	1.00			
PM2.5_ first trimester_T2	1.24	0.85	1.80	0.26
PM2.5_ first trimester_T3	1.48	1.02	2.14	0.04
PM2.5_ second trimester_T1	1.00			
PM2.5_ second trimester_T2	1.02	0.71	1.47	0.91
PM2.5_ second trimester_T3	1.39	0.97	1.99	0.07
PM2.5_ third trimester_T1	1.00			
PM2.5_ third trimester_T2	1.11	0.77	1.60	0.58
PM2.5_ third trimester_T3	1.15	0.80	1.67	0.44
PM2.5_ entire pregnancy_Q1	1.00			
PM2.5_ entire pregnancy_Q2	1.24	0.81	1.90	0.32
PM2.5_ entire pregnancy_Q3	1.28	0.83	1.96	0.27
PM2.5_ entire pregnancy_Q4	1.84	1.20	2.84	0.01
PM2.5_ first trimester_Q1	1.00			
PM2.5_ first trimester_Q2	1.43	0.94	2.18	0.09
PM2.5_ first trimester_Q3	1.22	0.80	1.85	0.36
PM2.5_ first trimester_Q4	1.66	1.09	2.54	0.02
PM2.5_ second trimester_Q1	1.00			
PM2.5_ second trimester_Q2	0.70	0.46	1.06	0.09
PM2.5_ second trimester_Q3	1.19	0.79	1.79	0.42
PM2.5_ second trimester_Q4	1.28	0.85	1.94	0.24
PM2.5_ third trimester_Q1	1.00			
PM2.5_ third trimester_Q2	0.91	0.60	1.38	0.65
PM2.5_ third trimester_Q3	0.97	0.64	1.46	0.87
PM2.5_ third trimester_Q4	1.14	0.75	1.73	0.54
PM2.5_ entire pregnancy (IQR)	1.29	1.06	1.57	0.01
PM2.5_ first trimester (IQR)	1.27	1.04	1.54	0.02
PM2.5_ second trimester (IQR)	1.26	1.02	1.56	0.04
PM2.5_ third trimester (IQR)	1.11	0.90	1.37	0.35

For the omics analysis of 48 placental samples, due to the relatively small sample size and to facilitate analytical feasibility, we dichotomized $PM_{2.5}$ exposure using tertiles, defining the lowest tertile (< 24.70 μg/m^3) as the 'low-exposure group' and the highest tertile (> 29.21 μg/m^3) as the 'high-exposure group'. The specific cut-off values defining these tertiles are now stated in the revised manuscript. We also acknowledge the potential limitations of this dichotomization and suggest that future studies may consider using

continuous exposure metrics or exploring alternative classifications. The following description has been incorporated into the revised manuscript:

Line 685-: To further validate these findings, we conducted *in vitro* experiments exposing M2 macrophages to PM, directly assessing its effects on their transcriptional profiles Third, the relatively small sample size for placental methylome and transcriptome analyses, coupled with the dichotomization of $PM_{2.5}$ exposure, likely reduced statistical power and constrained the generalizability of our results.

Line 706-: These compensatory strategies, including enhanced exposure modeling, a targeted focus on $PM_{2.5}$ as an environmental proxy, experimental validation, and the integration of multiple analyses and datasets, strengthen our findings and support the hypothesis that $PM_{2.5}$ exposure induces durable epigenetic changes, particularly in M2 macrophages, which could play a central role in the development of AD. Future studies with larger sample sizes are warranted and should consider utilizing continuous exposure metrics or exploring alternative classification methods to more accurately capture the complexity of exposure–response relationships.

Line 863-: To examine the impact of prenatal PM exposure on the epigenome and transcriptome and its association with AD, we compared the AD status of offspring between low and high PM exposure levels. $PM_{2.5}$ exposure during the first trimester was dichotomized based on tertile distribution, with the lowest tertile (< 24.70 μg/m³) designated as the low-exposure group and the highest tertile (> 29.21 μg/m³) as the high-exposure group, to accommodate the limited sample size and facilitate robust statistical comparisons.

Major issues:
Data:
1. It is unclear which datasets were used for each analysis. The study population is described as a cohort study using the COCOA cohort, which was then matched with air pollution data. My understanding is 986 children was only used for figure 1a, and then 48 placenta and cord blood were used for other analysis. However, it is confusing where the adult samples are from. COCOA only follow up until 10 years according to their descriptions, so I guess adults are not from this cohort. There is no description of what the adult samples are or where they come from in study population section. Additionally, there are no summary statistics or explanations for the healthy and AD adult groups. How are AD adults defined? Is the definition consistent with the criteria used for children? Also, are the placental and fetal datasets derived from the COCOA cohort?

We thank the reviewer for this important question regarding dataset sources and sample definitions. The placental and fetal datasets analyzed in this study were derived from the COCOA cohort, and additional details regarding these samples have been included in the revised manuscript.

In contrast, adult samples were obtained from three independent datasets—Tsoi LC et al., Reynolds G et al., and Mitamura Y et al.—which are not part of the COCOA study. These datasets were used to validate $FCER1G^{+}$-

associated patterns in skin across different developmental stages at the bulk transcriptome, single-cell transcriptome, and spatial transcriptome levels, respectively. The coordination of these samples and their data sources has been clarified in the revised experimental design diagram (Figure 1b) to improve transparency regarding the study workflow and dataset origins.

Line 775-: To investigate the mechanisms underlying AD associated with prenatal $PM_{2.5}$ exposure, we selected 48 placental samples and 46 cord blood samples from the COCOA cohort, based on $PM_{2.5}$ exposure levels during the first trimester of pregnancy and the diagnosis of AD in offspring at 2 and 3 years of age.

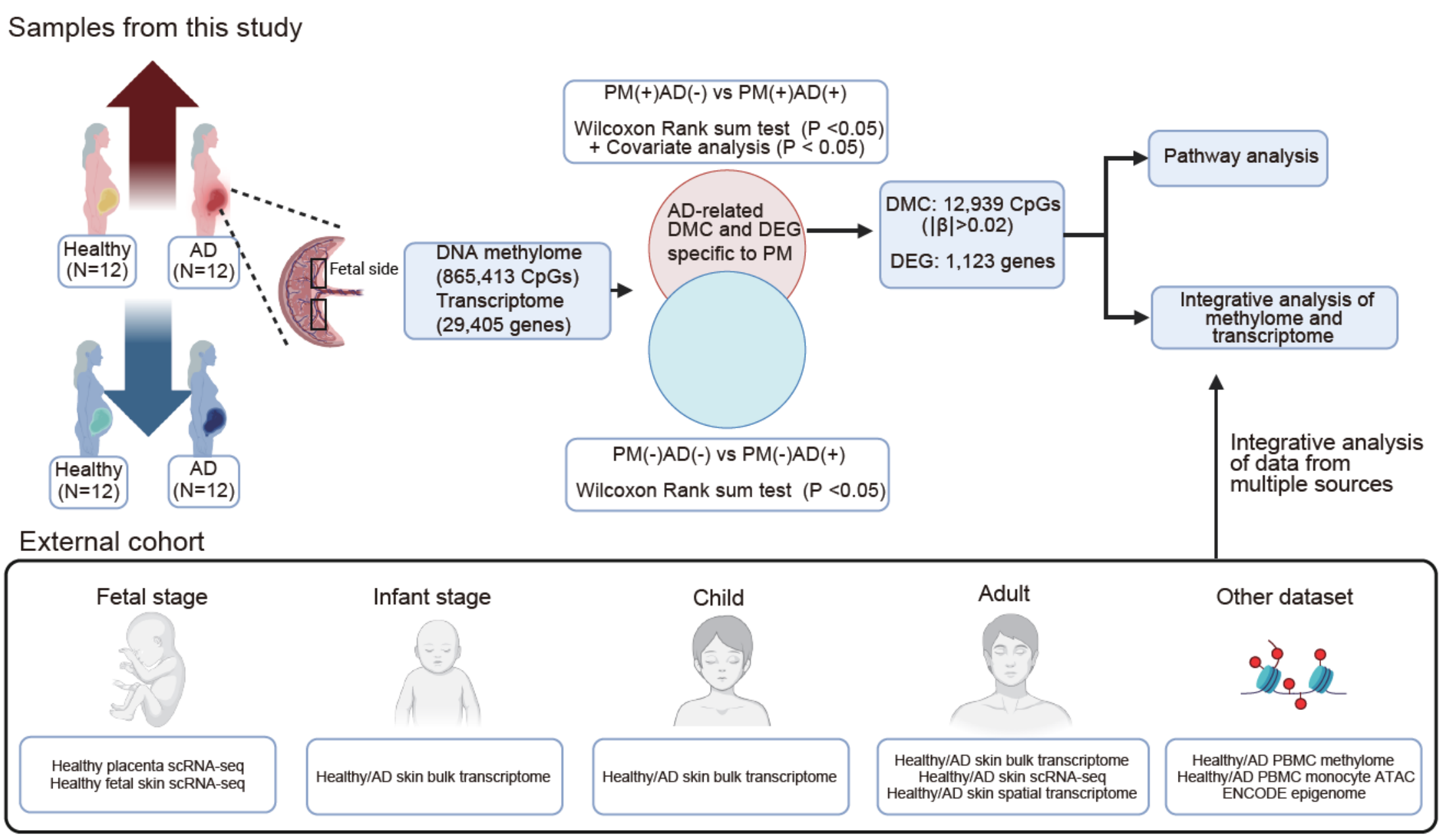

Fig. 1b. Schematic of the experimental design, analytic pipeline, and external resources. Placental samples were collected from the fetal side and profiled by DNA methylome and transcriptome analyses. Samples were stratified by $PM_{2.5}$ exposure levels and AD status (n = 12 per group) to identify $PM_{2.5}$-associated epigenetic and transcriptional patterns related to AD. The stage-specific external datasets are listed in the bottom panel; these were used for cross-cohort validation and multi-source integration. The schematic in panel b was created using BioRender.com

Abstract:

2. The statement in abstract, "We hypothesize that prenatal PM2.5 exposure induces specific epigenetic modifications, primarily hypomethylation of immune-related genes in certain placental immune cell types. This epigenetic scar could later re-emerge in offspring and adult immune cells, potentially priming them for heightened inflammatory responses and increased AD risk." may overstate the evidence provided in this study.

We thank the reviewer for this valuable comment highlighting important limitations of our study. In response, we have revised the title, abstract, and relevant sections to avoid the definitive causal implication of the term

“epigenetic scar,” replacing it with the more cautious term “epigenetic alteration” throughout the manuscript. We also explicitly acknowledge the absence of longitudinal epigenomic or single-cell epigenome data tracking individuals from prenatal exposure through childhood to adulthood, as such sampling is highly challenging. The revised title now reads:

“Prenatal particulate matter exposure induces an epigenetic alteration in FCER1G in fetal macrophages, influencing M2 macrophage function in atopic dermatitis development.”

Specifically, it is not clear whether the COCOA data set includes follow-up of children into adulthood, or whether your adult skin cell samples include corresponding prenatal exposure data. Without longitudinal data linking prenatal exposure, childhood, and adult outcomes within the same individuals, it is difficult to assert that the observed epigenetic modifications re-emerge in adult immune cells as a consequence of prenatal PM2.5 exposure. There are other potential confounding factors and exposures that could influence the epigenome after the prenatal period.

The adult skin samples analyzed in this study are independent of the COCOA cohort and are not linked to corresponding prenatal PM exposure data. Therefore, we cannot directly demonstrate re-emergence of these epigenetic changes in adult immune cells as a consequence of prenatal exposure, and residual confounding from postnatal exposures cannot be excluded.

Nonetheless, our multi-omics integration provides indirect but converging evidence that prenatal PM2.5 exposure–associated hypomethylation in the regulatory region of FCER1G may contribute to its overexpression in M2 macrophages. The FCER1G$^+$ M2 macrophage signature identified in the fetal stage was consistently observed during AD pathogenesis across developmental stages, as demonstrated by single-cell RNA sequencing of 404,169 cells from placental, fetal, and adult skin samples. Additional functional analyses confirmed the association of FCER1G expression with ROS pathway activation and CCL13–ACKR1–mediated immune interactions, both relevant to AD inflammation.

To further strengthen causal inference, as also noted in our response to Reviewer 1, we conducted a one-sample Mendelian randomization analysis within our cohort, which supported the association between FCER1G methylation patterns and AD status. Clinically, FCER1G$^+$ M2 macrophage signatures were significantly enriched in severe cases, as measured by SCORAD, linking them to disease progression.

We have tempered the language across the manuscript to reflect the observational nature of these findings, the absence of longitudinal linkage between prenatal exposure and adult outcomes, and the potential influence of other environmental factors after birth, while retaining the scientific relevance of our integrated multi-omic and functional evidence.

Line 65-: Integrated epigenomic and transcriptomic profiling of 48 placentae identified $PM_{2.5}$-associated hypomethylation driving FCER1G overexpression, predominantly in fetal macrophages. This epigenetic alteration was recapitulated across developmental stages in independent cohorts, as demonstrated through single-cell RNA sequencing of 404,169 cells from placenta, fetal, and adult skin samples. In adult AD skin, FCER1G was progressively overexpressed in M2 macrophages, particularly within AD-specific cell states.

Line 71-: Network analysis and in vitro PM exposure experiments identified a mechanistic axis involving FCER1G, CD68, CYBB, MRC1, and MS4A4A, promoting NADPH oxidase–driven reactive oxygen species signaling. Additionally, CCL13 is upregulated in $FCER1G^{+}$ M2 macrophages and spatially colocalized with $ACKR1^{+}$ endothelial cells, suggesting a localized Th2 inflammatory circuit.

Line 201: The regulatory CpG sites, located 13,693 bp upstream and within an intronic region of FCER1G, were located within the same topologically associated domain and overlapped with active histone marks (H3K27ac, H3K4me1, and H3K4me3) in placental tissue, but not in the maternal basal plate of the maternal side (Fig. 2e). To assess the potential causal role of these epigenetic modifications, we applied a one-sample Mendelian Randomization (MR) approach using 24 placental samples. This analysis revealed that hypomethylation at cg05656486 and cg20806175 was significantly associated with increased risk of infantile AD, suggesting a direct contribution of these loci to disease susceptibility (Extended Data Figs. 1f, 1g; Supplementary Table 5). Notably, these associations remained statistically robust after adjusting for PM2.5 exposure levels and other key covariates.

Line 401-: In contrast, $FCER1G^{-}$ M2 macrophages and M1 macrophages did not exhibit significant increases across developmental stages. To assess the association with clinical severity, we stratified lesional AD patients by SCORAD, a validated index of AD severity. $FCER1G^{+}$ M2 macrophage enrichment scores were markedly elevated in patients with severe AD compared to those with mild to moderate disease (Extended Data Fig. 5c), establishing a positive correlation between $FCER1G^{+}$ M2 macrophage abundance and disease severity. These findings suggest that the re-emergence of FCER1G+ M2 macrophages contributes to the development of AD at both cellular and developmental trajectory levels.

Line 475-: Single-cell trajectory analyses further demonstrated a progressive upregulation of these genes in M2 macrophages transitioning from healthy to AD states, confirmed by both gene expression and splicing-based status inference analyses (Fig. 6h, right). At the tissue level, gene set enrichment analysis demonstrated a marked elevation of FCER1G module scores in lesional AD skin relative to paired non-lesional samples (Extended Data Fig. 9e-j). Concordantly, NADPH oxidase–mediated ROS signaling exhibited a parallel activation trajectory. Correlation analysis across individual samples confirmed a strong positive association between FCER1G module activity and ROS signaling across all developmental stages. This cellular signaling cascade could be imprinted by early-stage PM exposure.

Line 559-: Further histopathological analysis confirmed a spatially coordinated increase in $FCER1G^{+}$ M2 macrophages and ROS signaling factors across different

skin samples, demonstrating a consistent enrichment pattern from healthy to lesional AD skin (Fig. 7e). To explore their role in type 2 inflammation, we assessed the expression of Th2-associated chemokines. CCL13, previously implicated in AD severity[39,40], were progressively upregulated in FCER1G+ M2 macrophages along the healthy-to-lesional axis (Extended Data Fig. 11). This expression trajectory aligns with SCORAD severity correlations reported in prior studies[40,41]. Spatial co-localization analysis revealed that both chemokines were enriched in FCER1G+ M2 macrophages and colocalized with their receptor, ACKR1, which was predominantly expressed in VE3 cells, a vascular endothelial subset. ACKR1 expression in VE3 cells also increased progressively with disease severity. These results establish a spatially coordinated interaction between FCER1G+ M2 macrophages and VE cells via the CCL13–ACKR1 axis, suggesting a mechanistic role in ROS-driven Th2 inflammation during AD progression.

Line 616-: Our findings underscore the potential role of epigenetically modified $FCER1G^+$ M2 macrophages in immune priming during early fetal development, contributing to an increased risk of AD in offspring via ROS activation. Beyond ROS activation, spatial transcriptomics revealed that $FCER1G^+$ M2 macrophages progressively upregulated the Th2-associated chemokine CCL13, which colocalized with ACKR1-expressing VE3 endothelial cells, establishing a localized immunological axis within AD lesions. Together, these findings highlight $FCER1G^+$ M2 macrophages as a critical cellular population linking prenatal PM2.5 exposure to AD pathogenesis through sustained epigenetic dysregulation, ROS-mediated signaling, and CCL13–ACKR1-driven Th2 inflammation.

Line 687-: Third, the relatively small sample size for placental methylome and transcriptome analyses, coupled with the dichotomization of $PM_{2.5}$ exposure, likely reduced statistical power and constrained the generalizability of our results. Collection of large quantities of placental tissue is challenging, and comprehensive profiling methylome and transcriptome profiling requires highly specialized tissue processing. Additionally, we acknowledge that we did not directly assess the epigenetic profiles of dermal macrophages in adult AD skin tissue, which limits our ability to fully validate the maintenance of placental epigenetic alterations in affected skin. To mitigate these constraints, we leveraged Mendelian randomization analysis to validate the causal relationship between $PM_{2.5}$-associated methylation loci and AD status, incorporating sex, maternal age, maternal AD status, birth weight, maternal secondhand smoke exposure and maternal cotinine as a covariate. Furthermore, we supplemented our findings with independent datasets spanning multiple developmental stages and disease contexts of AD. These included DNA methylome, chromatin accessibility, and transcriptome data, encompassing both bulk tissues and single-cell resolution, to reinforce the robustness and biological relevance of our conclusions.

Method:

3. Did you account for any changes in participants' residential addresses during pregnancy? If so, how was this handled in the exposure assignment?

We would like to clarify that in cases where participants moved during the study period, we did incorporate the exposure estimates from the new

residential address. When a relocation occurred, we updated the exposure assignment to reflect the $PM_{2.5}$ levels at the new address, ensuring that the exposure estimate was as accurate as possible during the remainder of the pregnancy. We have revised the manuscript to clearly describe this approach and the steps taken to account for residential address changes.

Line 760-: When residential addresses changed during the exposure period, updated addresses were used for assessment.

4. You mention using "station records routinely"—could you clarify the time interval for PM2.5 measurements (e.g., hourly, daily)? What kind a land-use regression (LUR) model or another type of exposure model used?

We appreciate the reviewer's helpful comment and the request for further clarification regarding the time interval for $PM_{2.5}$ measurements and the exposure model employed. The $PM_{2.5}$ measurements used in this study were obtained from station records provided by local environmental monitoring networks, which report data on a daily basis. We have clarified this in the revised manuscript to specify that the data used in our analysis were averaged daily $PM_{2.5}$ concentrations over the study period. For exposure estimation, we used a land-use regression (LUR) model with a previously described standardized method to predict $PM_{2.5}$ concentrations at specific residential locations (PMID: 29463050 and PMID: 31746563). This model incorporates data on known pollution sources, land-use types, and other geographical features to provide a spatially detailed estimate of $PM_{2.5}$ exposure. The LUR model was developed using geospatial data from various environmental monitoring stations in combination with local land-use data, and it provides estimates that account for the variability of exposure across different areas. We have updated the manuscript to include this additional information regarding the time interval for $PM_{2.5}$ measurements and the specific exposure modeling techniques used.

Line 748-: Residential exposure to $PM_{2.5}$ was estimated using land-use regression (LUR) models, following a previously validated standardized methodology[59]. Briefly, LUR models were developed using data from 37 regulatory monitoring stations in Seoul, operated by the Korean Ministry of Environment (http://www.airkorea.or.kr/web). Geographic predictor variables—including traffic indicators, land use, topography, and spatial trends—were derived using ArcGIS v9.3 (ESRI, Redlands, CA).
Multiple linear regression models were constructed using a supervised forward stepwise procedure. Final models included predictors such as total road length, traffic intensity on the nearest roads, heavy-duty traffic load, and a spatial trend variable. The models explained 66–81% of the variability in measured concentrations. Specifically, the adjusted R^2 and leave-one-out cross-validation (LOOCV) R^2 values were 0.66 and 0.56 for $PM_{2.5}$[59].
Daily exposure estimates were averaged over pregnancy and the first 3 years of life. When residential addresses changed during the exposure period, updated addresses were used for assessment. Prenatal exposure was assessed by calculating the average pollutant concentrations over the entire pregnancy period and separately for the third

trimester. The prenatal period was divided into trimesters as follows: weeks 1–13 (first trimester), 14–27 (second), and 28–40 (third). Postnatal exposure was estimated as the annual average concentration for each year from birth to age 3.

5. How were the 1–3 year PM2.5 exposure data collected? Were these based on participants' residential addresses during pregnancy or at another time point?

The 1–3 year $PM_{2.5}$ exposure data were assessed based on the current residential address at the time of the follow-up. This allowed us to more accurately reflect the $PM_{2.5}$ exposure during the early childhood years. We have updated the manuscript to clarify this point and ensure that the methodology regarding exposure assessment at this time point is clear.

Line 759-: Daily exposure estimates were averaged over pregnancy and the first 3 years of life. When residential addresses changed during the exposure period, updated addresses were used for assessment. Prenatal exposure was assessed by calculating the average pollutant concentrations over the entire pregnancy period and separately for the third trimester. The prenatal period was divided into trimesters as follows: weeks 1–13 (first trimester), 14–27 (second), and 28–40 (third). Postnatal exposure was estimated as the annual average concentration for each year from birth to age 3.

6. PM2.5 levels were categorized into tertiles (T1–T3, n = 138) but the overall cohort size is stated as 986. Please clarify this discrepancy: how were exposure groups constructed and how do they relate to the full cohort size? Also, why used tertiles in logistic regression analysis and then use two low vs high group in other analysis.

Thank you for your careful review. We would like to clarify that the cohort size is indeed 986, as stated in the manuscript. The mention of n = 138 appears to be a misunderstanding. We appreciate your attention to this detail and hope this clarifies the discrepancy.

Line 156-: Pregnancy was divided into three trimesters, as follows: 1 to 13 weeks (first trimester), 14 to 27 weeks (second trimester), and 28 to 40 weeks (third trimester). $PM_{2.5}$ levels were categorized into tertiles (T1–T3) (n = 986), with T1 being the lowest and T3 being the highest.

In our epidemiological analysis of 986 cohort participants, $PM_{2.5}$ exposure was assessed as a continuous variable as well as categorized based on the median, tertiles, and interquartile range (IQR). Across these approaches, higher $PM_{2.5}$ exposure was consistently associated with a statistically significant increase in the odds ratio (OR) for atopic dermatitis (AD) incidence. For omics analysis of 48 placental samples, we dichotomized $PM_{2.5}$ exposure based on tertiles, defining the lowest tertile (< 24.70 μg/m³) as the low-exposure group and the highest tertile (> 29.21 μg/m³) as the high-exposure group, primarily due to limited sample size and to simplify the analysis. The specific cut-off values defining these tertiles are now stated in the revised

manuscript. We also acknowledge the potential limitations of this dichotomization and suggest that future studies may consider using continuous exposure metrics or exploring alternative classifications.

Line 685-: To further validate these findings, we conducted *in vitro* experiments exposing M2 macrophages to PM, directly assessing its effects on their transcriptional profiles. Third, the relatively small sample size for placental methylome and transcriptome analyses, coupled with the dichotomization of $PM_{2.5}$ exposure, likely reduced statistical power and constrained the generalizability of our results.

Line 706-: These compensatory strategies, including enhanced exposure modeling, a targeted focus on $PM_{2.5}$ as an environmental proxy, experimental validation, and the integration of multiple analyses and datasets, strengthen our findings and support the hypothesis that $PM_{2.5}$ exposure induces durable epigenetic changes, particularly in M2 macrophages, which could play a central role in the development of AD. Future studies with larger sample sizes are warranted and should consider utilizing continuous exposure metrics or exploring alternative classification methods to more accurately capture the complexity of exposure–response relationships.

Line 863-: To examine the impact of prenatal PM exposure on the epigenome and transcriptome and its association with AD, we compared the AD status of offspring between low and high PM exposure levels. $PM_{2.5}$ exposure during the first trimester was dichotomized based on tertile distribution, with the lowest tertile (< 24.70 μg/m³) designated as the low-exposure group and the highest tertile (> 29.21 μg/m³) as the high-exposure group, to accommodate the limited sample size and facilitate robust statistical comparisons.

7. Figure 1a states that models were adjusted for sex, parental allergic history, maternal age, education, BMI, secondhand smoke, and breastfeeding. Elsewhere, only fetal sex, weight, and maternal AD status are mentioned. Please clarify which confounders were included in which models.

We acknowledge the opportunity to clarify the covariate adjustments applied in our analyses. The epidemiological analysis presented in Fig. 1a was adjusted for sex, parental allergic history, maternal age, maternal education, maternal body mass index, secondhand smoke exposure during pregnancy and infancy, and breastfeeding for six months. In contrast, the methylome and transcriptome analyses were adjusted for three variables—fetal sex, maternal age, and maternal AD status—because other covariates contained substantial missing values in these datasets. To assess the potential impact of residual confounding, particularly in relation to FCER1G methylation and expression, we performed additional analyses incorporating the available variables: birthweight, secondhand smoke exposure during pregnancy, and maternal cotinine levels. As summarized in the table below, the associations between FCER1G-related CpG methylation, gene expression, and AD status remained statistically significant after these additional adjustments, supporting the robustness of our findings.

	Covariate-adjusted P value	Fetal Sex P value	Maternal Age P value	Maternal AD P value	Birth weight P value	Maternal ETS P value	Maternal Cotinine P value
**FCER1G (gene expression)**	0.0305	0.3787	0.8704	0.2927	0.4780	0.3121	0.4897
**cg20806175**	0.0021	0.1455	0.5432	0.1732	0.5121	0.3606	0.2329
**cg05656486**	0.0015	0.0227	0.3949	0.3313	0.3923	0.6012	0.4699

8. Did you adjust for the effects of other air pollutants or seasonality in your analysis? This is important, as both factors can influence PM2.5 levels and the incidence of AD. For example, in Korea, springtime often brings elevated PM2.5 and dust pollution due to wind patterns. Failing to account for these variables could confound your results. Please clarify whether and how these factors were considered in your exposure assessment and statistical models.

Thank you for this important comment. We agree that other air pollutants and seasonal variation can influence both $PM_{2.5}$ exposure and the incidence of AD. In our study, we focused on $PM_{2.5}$ as the primary pollutant of interest. However, we acknowledge that not adjusting for other pollutants such as NO_2 or ozone, as well as for seasonality, may introduce potential confounding. To address these concerns, we conducted additional analyses adjusting for season of birth, which serves as a proxy for seasonal variation in exposure. The association between $PM_{2.5}$ exposure and AD incidence remained statistically significant after this adjustment, as presented in the table below. Furthermore, when NO_2 and ozone were included in the models, the association between $PM_{2.5}$ exposure and the incidence of AD showed attenuated statistical significance but remained suggestive of a meaningful trend, as shown in the table below. We further acknowledge the complexity of air pollution exposure and emphasize the need for future studies to apply more refined multi-pollutant modeling approaches to disentangle the interactive effects of environmental factors on AD.

aOR with additional control for season of birth

	a OR	95% CI		p value
PM2.5_ entire pregnancy_T1	1.00			
PM2.5_ entire pregnancy_T2	1.43	0.97	2.09	0.07
PM2.5_ entire pregnancy_T3	1.70	1.16	2.50	0.01
PM2.5_ first trimester_T1	1.00			
PM2.5_ first trimester_T2	1.51	0.98	2.33	0.06
PM2.5_ first trimester_T3	1.93	1.19	3.12	0.01
PM2.5_ second trimester_T1	1.00			
PM2.5_ second trimester_T2	1.04	0.68	1.59	0.86
PM2.5_ second trimester_T3	1.45	0.93	2.29	0.11
PM2.5_ third trimester_T1	1.00			
PM2.5_ third trimester_T2	1.26	0.82	1.93	0.28

PM2.5_ third trimester_T3	1.34	0.85	2.13	0.21

aOR with additional control for other pollutants (NO_2 and ozone)

	a OR	95% CI		p value
PM2.5_ entire pregnancy_T1	1.00			
PM2.5_ entire pregnancy_T2	1.36	0.91	2.04	0.14
PM2.5_ entire pregnancy_T3	1.55	1.04	2.32	0.03
PM2.5_ first trimester_T1	1.00			
PM2.5_ first trimester_T2	1.22	0.81	1.84	0.33
PM2.5_ first trimester_T3	1.51	0.98	2.32	0.06
PM2.5_ second trimester_T1	1.00			
PM2.5_ second trimester_T2	0.99	0.67	1.47	0.97
PM2.5_ second trimester_T3	1.31	0.86	1.99	0.21
PM2.5_ third trimester_T1	1.00			
PM2.5_ third trimester_T2	1.13	0.76	1.69	0.55
PM2.5_ third trimester_T3	1.10	0.73	1.67	0.65

Line 668-: Moreover, the use of a general population-based, prospective birth cohort study enhances the generalizability of our findings to the broader population[55,56]. Second, by focusing solely on $PM_{2.5}$, we cannot completely exclude the influence of other environmental factors on the development of AD, such as indoor pollutants, pollens, fungal spores, and meteorological conditions, which may confound or modify the effects of $PM_{2.5}$. Among these potential confounders, we adjusted for season of birth, as well as NO_2 and ozone concentrations, and the significant association between $PM_{2.5}$ and AD remained significant. Nevertheless, we acknowledge the complexity of air pollution exposure and highlight the need for future studies to apply refined multi-pollutant modeling approaches to better disentangle the interactive effects of various environmental factors on AD.

9. How did your account for potential PM2.5 exposure after birth (i.e., in infancy or early childhood)? Postnatal exposure can also increase the risk of AD. How did you adjust for this?

We appreciate the reviewer's insightful question. Our analyses indicated that $PM_{2.5}$ exposure during early childhood (1-3 years) did not show a significant association with AD risk. Upon adjustment for postnatal exposure, the relationship between $PM_{2.5}$ exposure across the entire pregnancy and AD incidence remained statistically significant, while the effect of first-trimester exposure was attenuated and lost significance, as shown in the table below. These findings suggest that prenatal $PM_{2.5}$ exposure exerts a more substantial influence on AD development compared to exposure the postnatal period. We have included this finding in the discussion section.

Line 673-: Among these potential confounders, we adjusted for season of birth, as well as NO_2 and ozone concentrations, and the significant association between $PM_{2.5}$ and AD remained significant. $PM_{2.5}$ exposure during early childhood did not affect the

risk of AD, and even after adjusting for this exposure, the impact of prenatal $PM_{2.5}$ exposure on AD remained significant, highlighting the importance of prenatal exposure.

	a OR	95% CI		p value
PM2.5_ entire pregnancy_T1	1.00			
PM2.5_ entire pregnancy_T2	1.43	0.96	2.11	0.08
PM2.5_ entire pregnancy_T3	1.71	1.07	2.73	0.03
PM2.5_ first trimester_T1	1.00			
PM2.5_ first trimester_T2	1.21	0.83	1.77	0.31
PM2.5_ first trimester_T3	1.38	0.92	2.08	0.12
PM2.5_ second trimester_T1	1.00			
PM2.5_ second trimester_T2	1.00	0.69	1.45	1.00
PM2.5_ second trimester_T3	1.30	0.86	1.96	0.22
PM2.5_ third trimester_T1	1.00			
PM2.5_ third trimester_T2	1.09	0.76	1.58	0.63
PM2.5_ third trimester_T3	1.03	0.69	1.53	0.90

10. Please explain your rationale and logic for dividing the exposure groups (low and high). What exposure do you use? Entire? First trimester? It is not clear. What does high mean? It is well known that there is no safe level for AAP, and without showing how you defined the low vs high dichotomize the exposure is not biologically justified. While the dichotomization is necessary given the small sample size and limited power (which the authors may need to mention), the authors may explore other outpoints to assess the robustness of the results. Also, without showing how you set low vs high, the statement that high exposure may lead the AD risk cannot be jsutified. Did you follow the WHO guideline? The World Health Organization (WHO) also recommends that annual average PM2.5 concentrations should not exceed 5 μg/m³, and 24-hour average exposures should not exceed 15 μg/m³ more than 3-4 days per year

We thank the reviewer for pointing out the need to clarify the classification of $PM_{2.5}$ exposure levels. In our epidemiological analysis, $PM_{2.5}$ exposure was assessed as a continuous variable as well as categorized based on the median, tertiles, and interquartile range (IQR). As detailed in our primary response, all approaches consistently demonstrated a statistically significant increase in the odds ratio (OR) for atopic dermatitis (AD) incidence with higher $PM_{2.5}$ exposure. In regards with the WHO Air Quality Guidelines, the average $PM_{2.5}$ concentrations in Korea, including those observed among our study participants, far exceed these recommended levels. Moreover, our exposure assessment was based on averaged concentrations over a specific study period (e.g., yearly or trimester averages), rather than daily concentrations. Given these constraints, it was not feasible to directly apply the WHO short-term (24-hour) guideline in our analysis.

For the omics analyses, including methylome correlation profiles, we dichotomized first-trimester PM2.5 exposure into tertiles to address the relatively small sample size and ensure analytical feasibility. The lowest tertile (< 24.70 μg/m³) was designated as the low-exposure group, and the highest tertile (> 29.21 μg/m³) as the high-exposure group. We recognize the potential limitations of this dichotomization and note that future studies with larger sample sizes may benefit from modeling PM2.5 as a continuous variable or employing alternative classification strategies to capture exposure gradients more precisely.

Line 685-: To further validate these findings, we conducted *in vitro* experiments exposing M2 macrophages to PM, directly assessing its effects on their transcriptional profiles. Third, the relatively small sample size for placental methylome and transcriptome analyses, coupled with the dichotomization of $PM_{2.5}$ exposure, likely reduced statistical power and constrained the generalizability of our results.

Line 706-: These compensatory strategies, including enhanced exposure modeling, a targeted focus on $PM_{2.5}$ as an environmental proxy, experimental validation, and the integration of multiple analyses and datasets, strengthen our findings and support the hypothesis that $PM_{2.5}$ exposure induces durable epigenetic changes, particularly in M2 macrophages, which could play a central role in the development of AD. Future studies with larger sample sizes are warranted and should consider utilizing continuous exposure metrics or exploring alternative classification methods to more accurately capture the complexity of exposure–response relationships.

Line 863-: To examine the impact of prenatal PM exposure on the epigenome and transcriptome and its association with AD, we compared the AD status of offspring between low and high PM exposure levels. $PM_{2.5}$ exposure during the first trimester was dichotomized based on tertile distribution, with the lowest tertile (< 24.70 μg/m³) designated as the low-exposure group and the highest tertile (> 29.21 μg/m³) as the high-exposure group, to accommodate the limited sample size and facilitate robust statistical comparisons.

11. Also, this is setting from Korea, it will be important to show what is average 2.5 exposure that pregnant women's confront and what would be considered as low or high, for readers not from Korea.

We appreciate the reviewer's insightful comment regarding the contextualization of $PM_{2.5}$ exposure levels for readers unfamiliar with the Korean setting. In this regard, previous studies conducted among pregnant women in Korea between 2010 and 2015 have reported average $PM_{2.5}$ concentrations during the entire pregnancy and across each trimester ranging from 26.72 to 26.84 μg/m³, which align closely with the exposure levels observed in our study. (PMID: 32768726.)

12. If you have categorized the groups based on first-trimester PM2.5 exposure, did you also examine whether there were differences in your results when using PM2.5 exposure from other periods of pregnancy?

In the epidemiological analysis, we evaluated $PM_{2.5}$ exposure across all pregnancy trimesters and observed a significant association with the outcome only for first-trimester exposure (Fig. 1a). Guided by this finding, and due to budgetary constraints and limited sample availability, the methylome and transcriptome analyses focused exclusively on first-trimester exposure, precluding assessment of exposure effects during later pregnancy periods in the omics datasets.

13. Air pollution exposures were assessed using modeled estimates based on an inverse distance-squared weighting, which is not considered as the gold standard model to estimate ambient air pollution. To minimize exposure misclassifications, the authors may use other well-validated modeled air pollution estimates with high spatiotemporal resolutions.

We would like to clarify that residential exposure to $PM_{2.5}$ in our study was not estimated using a simple inverse distance-squared weighting (IDW) method. Instead, we employed land-use regression (LUR) models, developed following a previously validated and standardized methodology (PMID: 29463050 and PMID: 31746563). These models incorporated data from 37 government-operated regulatory monitoring stations across Seoul and used multiple geographic predictor variables—such as traffic indicators, land use, topography, and spatial trends—derived using ArcGIS.

The LUR models were constructed using a supervised forward stepwise linear regression procedure, and the final models explained 66–81% of the variability in measured $PM_{2.5}$ concentrations. Specifically, the adjusted R^2 was 0.66, and the leave-one-out cross-validation (LOOCV) R^2 was 0.56, indicating good predictive performance. We believe that the use of this validated LUR modeling approach, along with averaging exposure estimates over relevant time windows (pregnancy and early childhood), helps minimize exposure misclassification. Nonetheless, we have added clarification about our modeling approach and acknowledged the potential for residual exposure misclassification in the revised Discussion section, as well as the potential benefits of integrating satellite-based or hybrid modeling approaches in future studies.

Line 658-: Our study has several limitations that should be acknowledged, along with compensatory strategies implemented to address them. First, to estimate individual-level exposure to ambient $PM_{2.5}$, we used land-use regression (LUR) models developed based on a previously validated and standardized methodology[54]. Personal monitoring of $PM_{2.5}$ exposure throughout pregnancy in large cohorts is logistically challenging and cost-prohibitive. The use of LUR modeling likely reduced exposure misclassification compared to simpler interpolation methods such as inverse distance weighting. Nevertheless, some degree of exposure misclassification may persist, particularly due to temporal variability or individual mobility, which we were not able to fully account for. Future studies may benefit from incorporating higher-resolution spatiotemporal models, including hybrid approaches that integrate satellite-

based data and chemical transport models, to further enhance exposure assessment accuracy.

14. Was multiple testing correction applied when evaluating significance (e.g., for several endpoints or pathways)? If so, please specify the method used. If you didn't use, why?

In our omics analyses, multiple testing correction using the Benjamini-Hochberg (BH) method did not yield statistically significant results. Previous studies have reported that FDR correction methods may reduce specificity and be unsuitable for analyses involving low-dimensional data or small sample sizes [PMID: 29499647, PMID: 16777905]. Therefore, we adopted an alternative approach based on nominal P values and implemented a two-step statistical filtering strategy using (1) the Wilcoxon rank-sum test and (2) ordinary least squares (linear regression), retaining only candidates that satisfied the significance threshold of $P < 0.05$ in both tests. Additionally, in our methylation data generated from EPIC microarray platform, we considered not only statistical significance but also biological effect size. Specifically, we selected CpG sites with absolute methylation differences ($|\Delta\beta|$) of at least 0.02, since smaller changes around 1% are likely to result from technical variability and lack biological relevance [PMID: 34863305]. For the pathway analyses, we performed with FDR correction and the results have been updated accordingly in the revised manuscript.

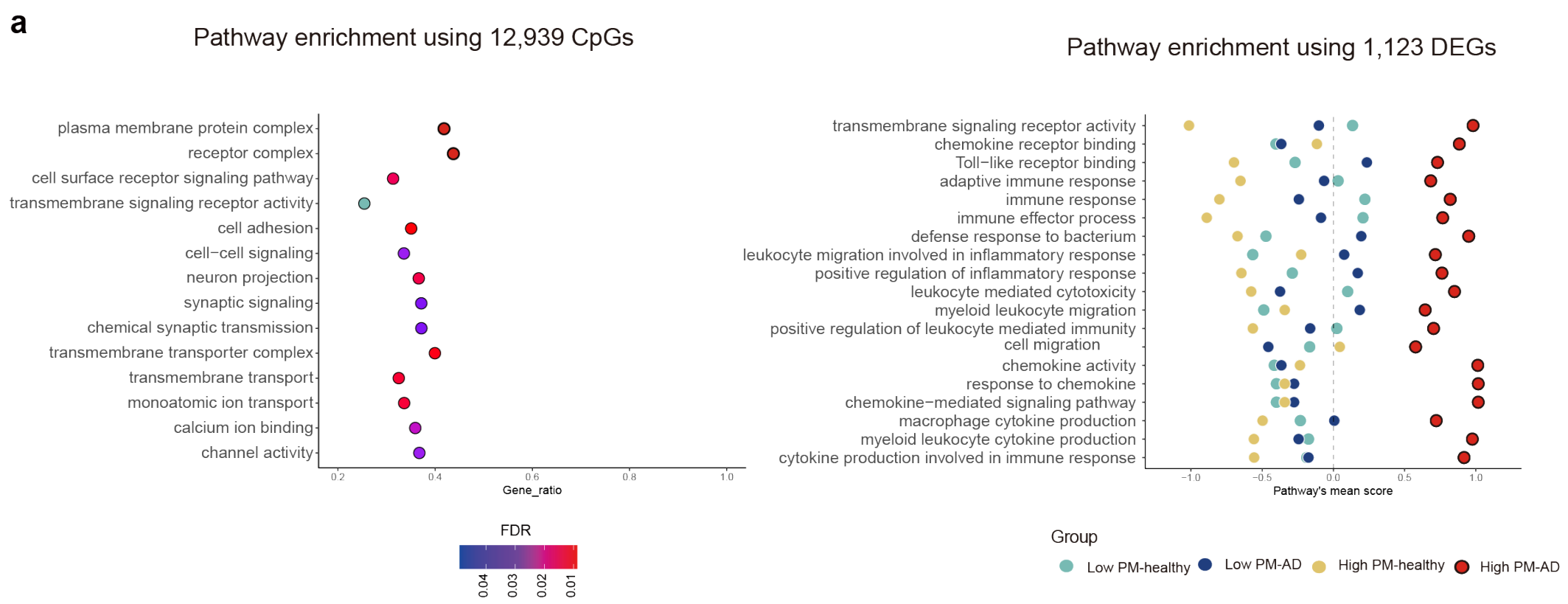

Extended Data Fig. 1a. Pathway analysis of differentially methylated CpG sites (DMCs) and differentially expressed genes (DEGs). Left: Pathway analysis of 12,939 DMCs was performed using the Gometh tool. The x-axis shows the gene ratio, representing the proportion of pathway-related genes relative to the total number of genes in a given pathway. Pathways with an FDR (Benjamini–Hochberg) < 0.05 were included. Right: The GSVA tool was used for pathway analysis of 1,123 DEGs based on the GO, KEGG, and Reactome databases. The x-axis displays the mean pathway scores across groups. Pathways with an FDR (Benjamini–Hochberg) < 0.05 were included. Statistical analyses included over-representation analysis for Gene Ontology (GO) and KEGG terms (left panel) and the Wilcoxon rank-sum test to compare the High PM-AD group with other groups (right panel).

15. Pathway enrichment analysis shows pronounced activation in AD offspring with high PM2.5, but low-PM/healthy and -AD groups have near-zero mean scores, while high-PM/healthy shows the lowest scores. It appears that PM2.5 exposure has opposing effects by AD status: activating in AD but deactivating in healthy. How do you interpret these findings? Please elaborate on possible biological explanations for this phenomenon.

For pathway analysis, we applied the GSVA algorithm to calculate pathway activity scores based on the expression levels of predefined gene sets, such that enrichment scores reflect the contribution of differentially expressed genes (DEGs) within each group. Our study was designed to elucidate how high PM exposure contributes to AD pathogenesis, focusing on AD-specific molecular signatures induced under high PM conditions by comparing high-PM/healthy and high-PM/AD groups. We hypothesized that high PM exposure induces broad epigenetic alterations, a subset of which may drive AD, whereas AD cases under low PM exposure are more likely to reflect genetic predisposition. Supporting this hypothesis, the number of differentially methylated CpGs and DEGs in the high-PM/healthy versus high-PM/AD comparison was over sixfold greater than in the corresponding low-PM comparisons. Furthermore, molecular signatures identified under low PM exposure did not overlap with those observed in the high PM context, underscoring the specificity of high-PM-driven AD responses. Consistently, pathway alterations between low-PM/healthy and low-PM/AD groups were minimal, with near-zero mean scores, whereas the high-PM/healthy versus high-PM/AD comparison revealed marked differences in pathway activity. Notably, allergy-related pathways were activated in patients with AD, as suggested by the reviewer and in agreement with previous reports of their broad activation following PM exposure in AD development (PMID: 22196522). This previously reported biological pattern has been incorporated into the revised manuscript.

Line 145-: Pathway enrichment analysis of DMCs and DEGs revealed substantial disruptions in allergy-related pathways (Fig. 1c and Extended Data Fig. 1a). Specifically, placentae from AD offspring exposed to high $PM_{2.5}$ showed pronounced activation of these pathways compared to other groups, consistent with previous reports describing their broad activation following PM exposure in AD development[16]. We designate this subgroup as the “placentae of AD offspring with an epigenetic alteration,” highlighting a potential mechanism by which $PM_{2.5}$-driven epigenetic modifications may elevate the risk of AD.

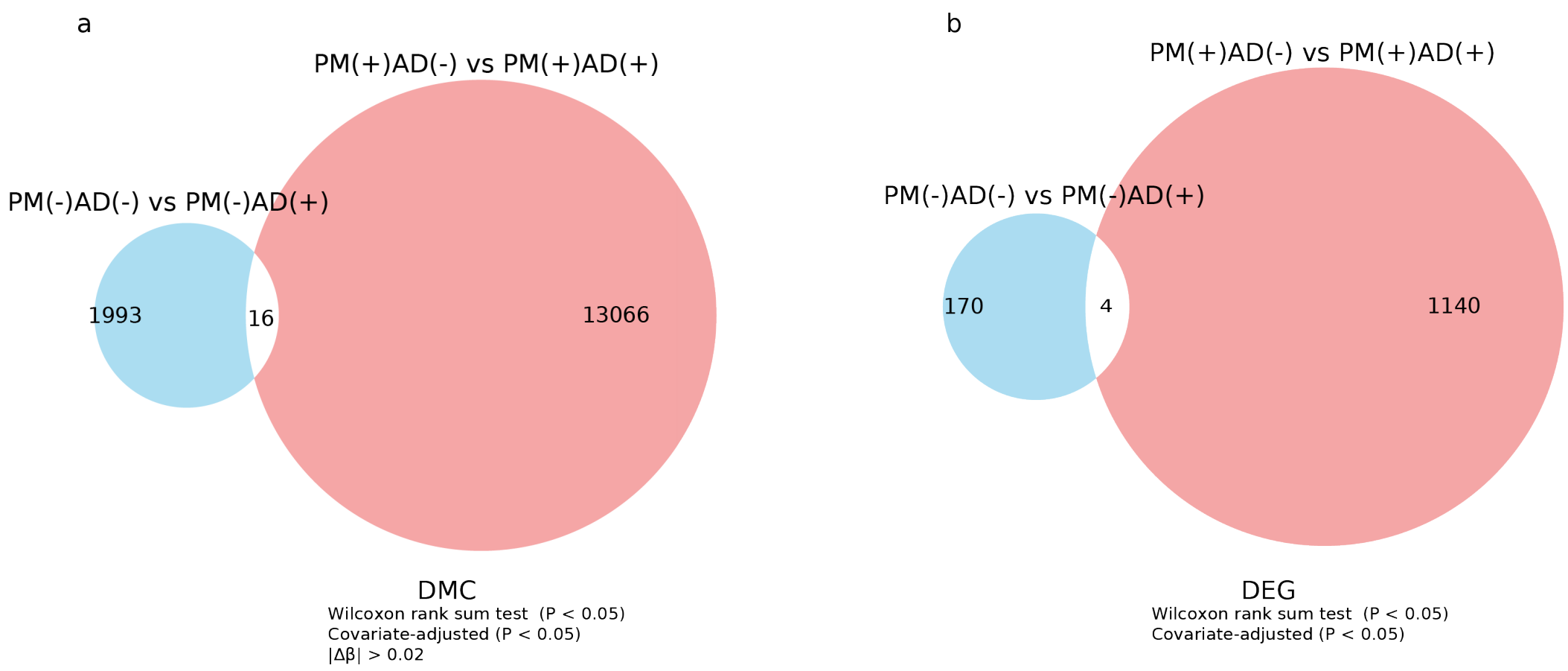

Venn diagram showing the number of differentially methylated CpGs (DMCs) and differentially expressed genes (DEGs) identified from comparisons between AD and healthy controls under low PM exposure (PM^-AD^- vs PM^-AD^+) and high PM exposure (PM^+AD^- vs PM^+AD^+).

16. Figure 3d -> boxplot doesn't distinct which one is low pm -healthy, low PM-ad, and High pm-healthy. Also, the boxplot shows that healthy child has lower count while AD has higher count. Also, not clear the p-value is indicating. Comparing from what? The effect of PM2.5 was same for both healthy and AD group?

As noted by the reviewer, we distinguished each group using different colors as described below and reported the statistical significance for all comparisons between healthy and AD groups under both low-PM and high-PM conditions. We highlighted that the difference in eosinophil counts between the high-PM/healthy and high-PM/AD groups was the most pronounced, with the greatest activation observed at 3 years of age, coinciding with the elevation of allergy-related molecular signatures.

d

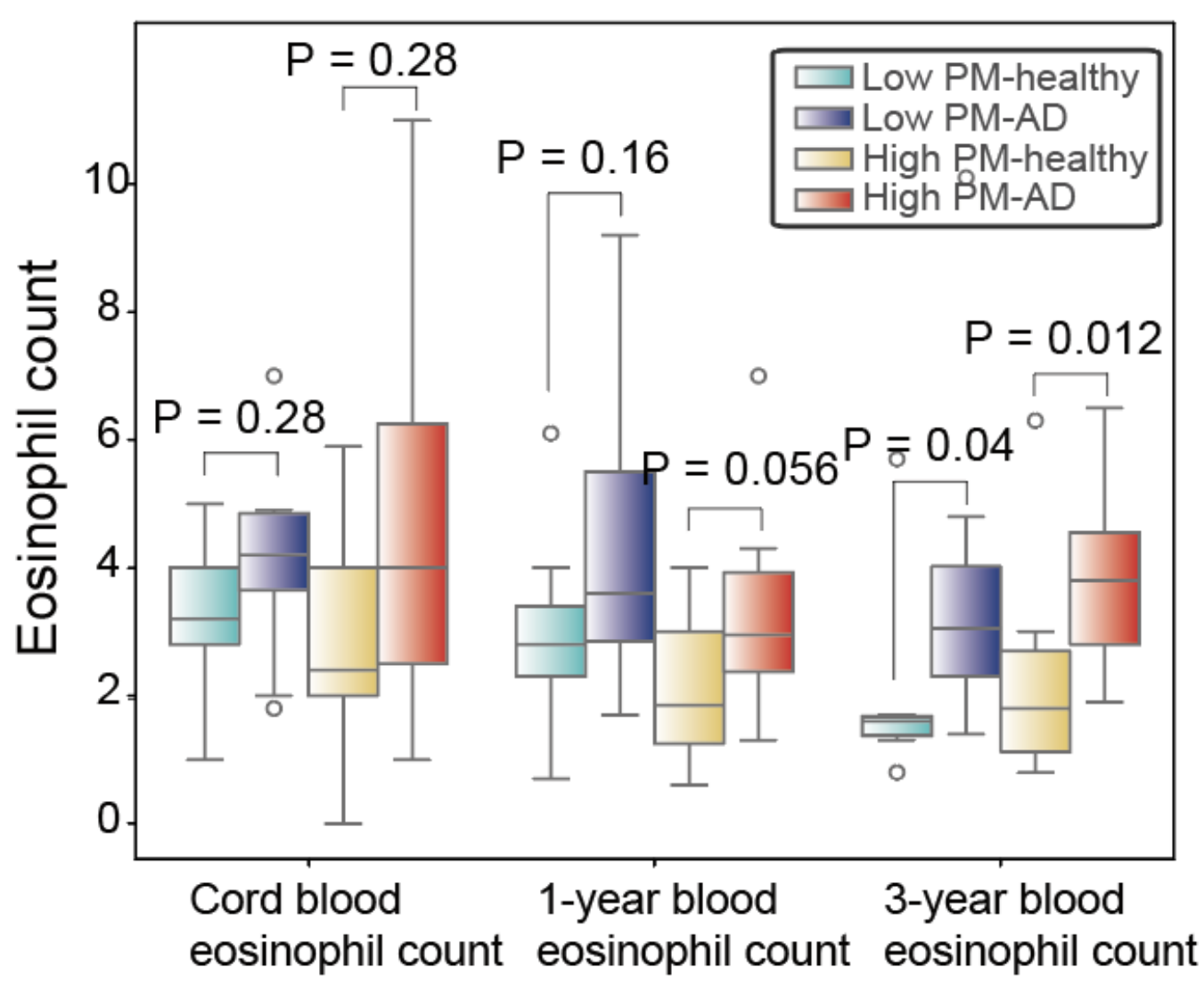

17. The “epigenetic scar” hypothesis would be best supported by formal mediation analysis, quantifying the role of methylation changes in mediating the PM2.5 → AD association. Have you tried this mediation analysis?

In response to the reviewer’s suggestion, we conducted a Mendelian Randomization (MR) analysis to assess the causal relationship between PM2.5-associated DNA methylation alterations and the risk of AD. Specifically, we evaluated the regulatory CpG loci cg05656486 and cg20806175, previously identified as hypomethylated under high PM2.5 exposure. The analysis revealed that decreased methylation at both loci was significantly associated with increased AD risk, supporting their potential causal role in disease pathogenesis. Importantly, this association remained robust after adjusting for key covariates, including PM2.5 exposure levels. While these findings suggest a plausible epigenetic mechanism linking prenatal environmental exposure to AD, further validation in larger cohorts is warranted given the limited sample size (n = 24) used in this MR analysis. This result has been incorporated into the revised manuscript.

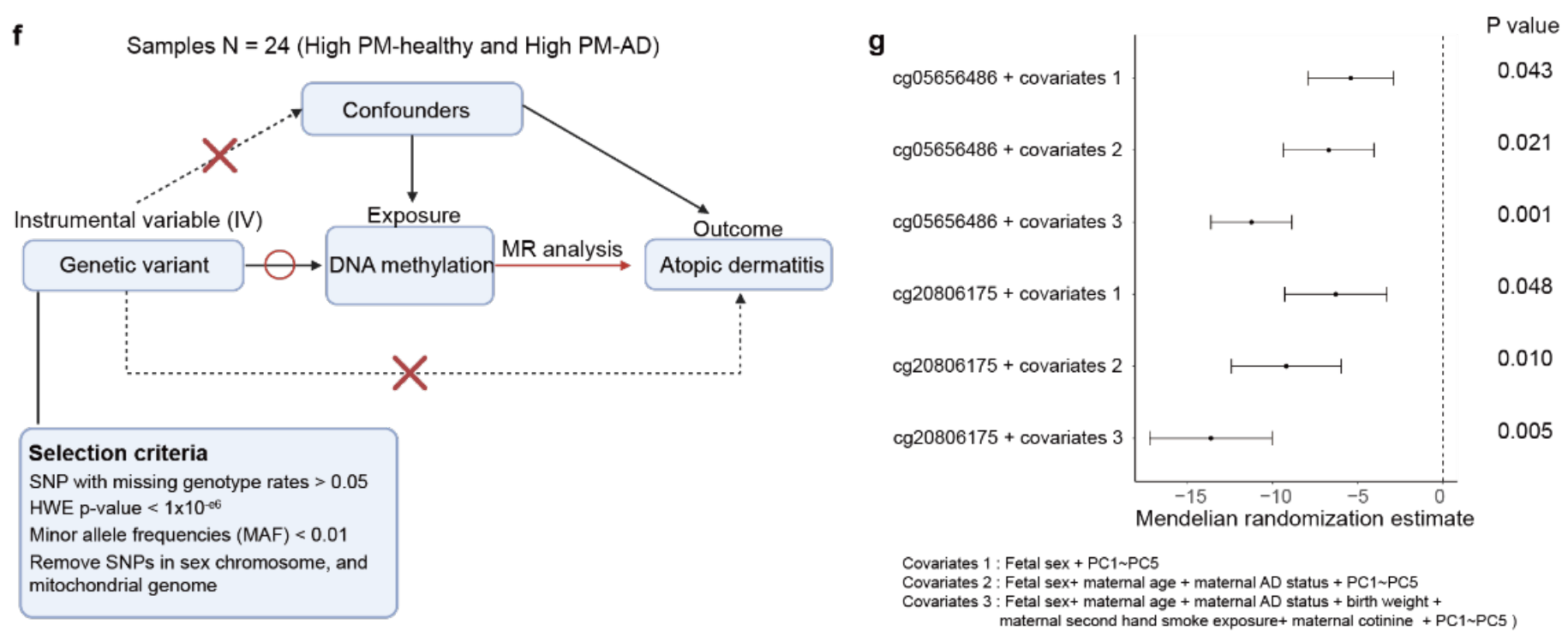

Extended Data Fig. 1f and 1g. Mendelian Randomization (MR) analysis to assess the causal role of DNA methylation in AD under high prenatal PM exposure. a, Schematic of the MR analytic pipeline. The analysis was performed using placental samples from the high prenatal PM exposure group in our cohort. b, Error bar plot showing the MR estimates for the CpG sites cg05656486 and cg20806175. P-values corresponding to each estimate are shown to the right of the plot. Covariate combinations included in the analysis are detailed below. The statistic was calculated using the ivreg function in R.

Line 201-: The regulatory CpG sites, located 13,693 bp upstream and within an intronic region of FCER1G, were located within the same topologically associated domain and overlapped with active histone marks (H3K27ac, H3K4me1, and H3K4me3) in placental tissue, but not in the maternal basal plate of the maternal side (Fig. 2e). To assess the potential causal role of these epigenetic modifications, we applied a one-sample Mendelian Randomization (MR) approach using 24 placental samples. This analysis revealed that hypomethylation at cg05656486 and cg20806175 was significantly associated with increased risk of infantile AD, suggesting a direct contribution of these loci to disease susceptibility (Extended Data Figs. 1f, 1g;

Supplementary Table 5). Notably, these associations remained statistically robust after adjusting for PM2.5 exposure levels and other key covariates.

Line 695-: To mitigate these constraints, we leveraged Mendelian randomization analysis to validate the causal relationship between $PM_{2.5}$-associated methylation loci and AD status, incorporating sex, maternal age, maternal AD status, birth weight, maternal secondhand smoke exposure and maternal cotinine as a covariate.

18. It would be helpful to draw a schematic diagram on causal path on pm to methylation change to immune alteration to ad to help readers follow the reasoning.

We thank the reviewer for this insightful suggestion. In line with the comment, we generated a schematic diagram illustrating the proposed causal pathway from prenatal PM2.5 exposure to placental epigenomic alterations, subsequent immune dysregulation, and ultimately the development of atopic dermatitis. This diagram has been incorporated as Fig. 7f, and a corresponding description has been added to the revised manuscript.

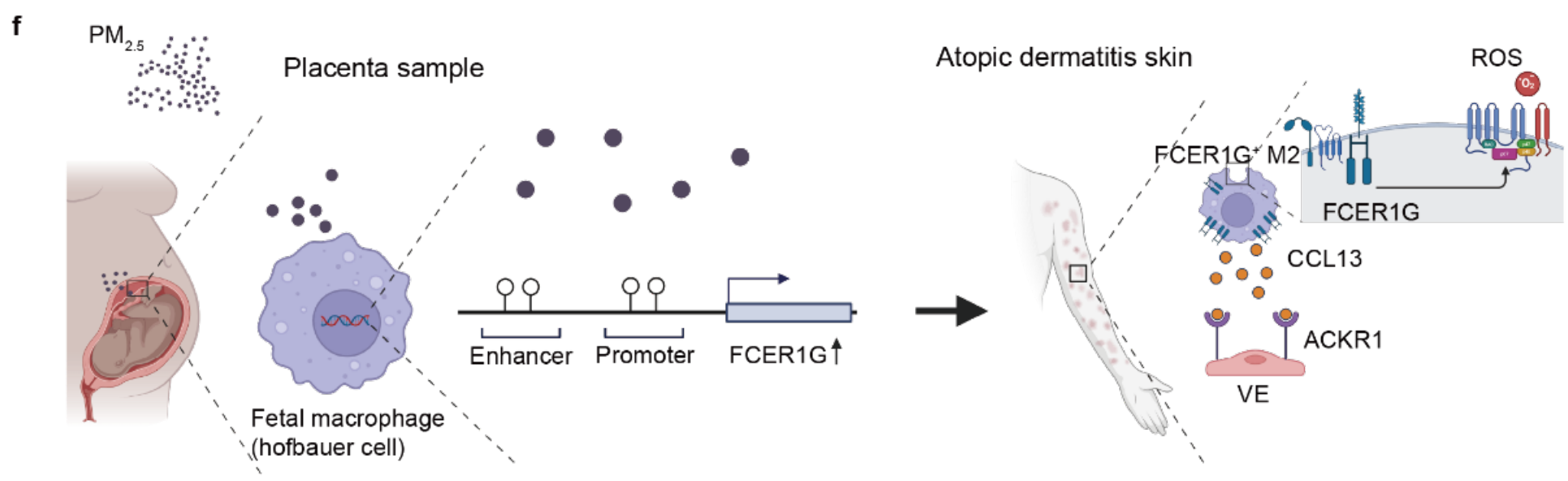

Fig. 7f. Schematic diagram represent the pathway from placental particulate matter exposure to M2 macrophage in AD skin. The schematic was created using BioRender.com

Line 570-: These results establish a spatially coordinated interaction between FCER1G+ M2 macrophages and VE cells via the CCL13–ACKR1 axis, suggesting a mechanistic role in ROS-driven Th2 inflammation during AD progression. Collectively, integrative single-cell and spatial transcriptomic analyses together with histopathological evidence support a model in which prenatal PM2.5 exposure driving the epigenetic alteration on placental macrophages that re-emerges as $FCER1G^{+}$ M2 macrophages in the skin, driving ROS-mediated signaling and Th2 inflammation during AD progression, as summarized in the proposed causal pathway (Fig. 7f).

19. Why supplementary table 2 and 3 only present PM10? PM2.5 info is missing.

Response to Reviewer's Comment:
Thank you for your comment. The data for $PM_{2.5}$ was incorrectly labeled as PM_{10} in Supplementary Tables 2 and 3. We have corrected this and updated the tables to reflect the accurate $PM_{2.5}$ information.

Minor
20. Line 214: **P ≤ 0.05 -> * P ≤ 0.05

We thank the reviewer for the careful observation. As suggested, we have corrected the notation in line 214 from "**P ≤ 0.05" to "*P ≤ 0.05" in the revised manuscript.

21. Boxplot 2-d. children (age between 1 and7??)

We apologize for the confusion. The age range of the children in Fig. 2d was between 0 and 7 years. We have updated the figure legend to accurately reflect this information. In addition, following Comment 7 from Reviewer 4, we reanalyzed the data using a nonparametric statistical method (Wilcoxon rank-sum test), as the data did not follow a normal distribution.

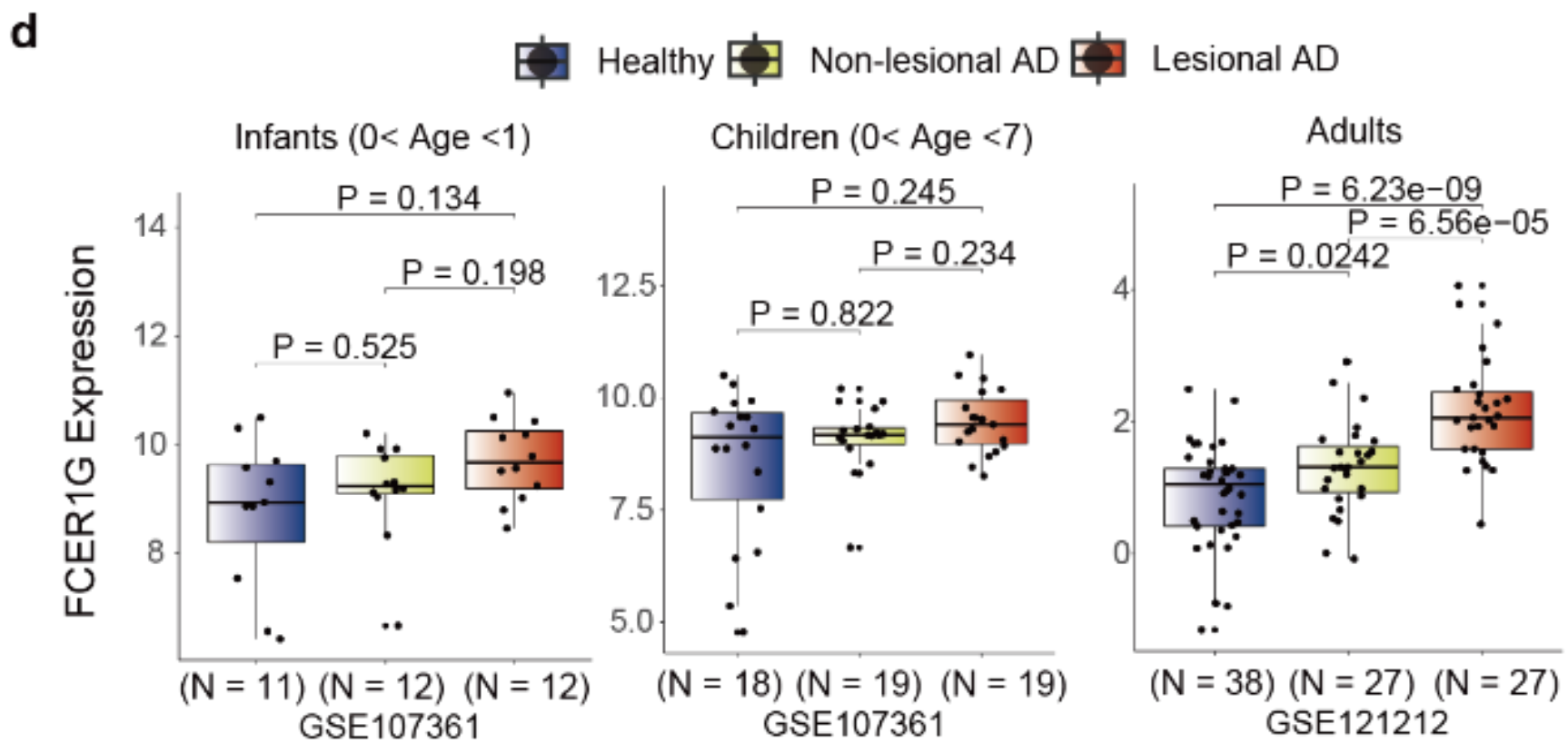

22. 2C is boxplot, not clear -> what is the sample?

The data in Fig. 2c were derived from peripheral blood samples collected from children aged 0.1 to 5.4 years. We have updated corresponding section in the manuscript to clarify the sample type and collection period.

Line 194-: A parallel analysis of cord blood methylome and transcriptome mirrored these trends but lacked statistical significance in the high $PM_{2.5}$ exposure groups (Extended Data Figs. 1d and 1e). Intriguingly, the FCER1G hypomethylation identified in placentae of offspring with AD and high prenatal $PM_{2.5}$ exposure was recapitulated in peripheral blood samples from children who later developed AD, collected between 0.1 and 5.4 years of age (GEO accession GSE152084) (Fig. 2c). This corresponded with a progressive overexpression of FCER1G in AD lesion samples across developmental stages, from infancy to adulthood[18,19] (Fig. 2d).

Reviewer #4 (Remarks to the Author):

The study by Yang et al aims to link prenatal PM2.5 exposure, FCER1G dysregulation and AD developments, by integrating analyses of placental epigenomic and transcriptomic data. The finding is interesting, but the statistical power is relatively low due to small sample size and no consideration of multiple-testing. The manuscript is well written. Attached please find my other comments.

We appreciate the reviewer's thoughtful comment regarding statistical power and multiple testing. To address concerns regarding statistical power and multiple testing, we conducted additional epidemiological analyses applying Bonferroni and false discovery rate (FDR) corrections. After adjustment, the association between first-trimester PM2.5 exposure and AD incidence was no longer statistically significant ($P = 0.118$), whereas the association for exposure across the entire pregnancy period remained significant ($P = 0.01$).

In our omics analyses, correction for multiple comparisons using the Benjamini-Hochberg (BH) method did not yield statistically significant results. Given the limited sample size and data dimensionality, we adopted a tailored approach in line with previous studies reporting that FDR correction can compromise specificity in small-scale datasets [PMID: 29499647, PMID: 16777905]. We therefore applied a two-step filtering strategy, selecting only candidates with nominal $P < 0.05$ in both the Wilcoxon rank-sum test and linear regression. For the methylation data, derived from the EPIC microarray platform, we additionally required an absolute methylation difference ($|\Delta\beta| \geq 0.02$) to ensure biological relevance and reduce the influence of technical noise [PMID: 34863305]. Pathway enrichment analyses were performed with FDR correction, and updated results are reflected in the revised manuscript.

1. In the analyses between air pollution and risk of AD in children, only sex, weight (at which time point?) and maternal AD status was considered as the covariates. However, other potential factors should also be considered, such as social-economic status, maternal/paternal smoking status, feeding pattern and season of birth (which may lead to variation in PM exposure) as well as PM2.5 exposure during early childhood. Some studies showed that PM2.5 concentration generally decline over years, does AD prevalence change over years in the study cohort? If it is, then years when PM2.5 was measured should as be considered as a covariate.

Thank you very much for your thoughtful comment. The epidemiological analysis presented in Figure 1a was adjusted for the following covariates: sex, parental allergic history, maternal age, maternal education, maternal body mass index, secondhand smoke exposure during pregnancy and infancy and and breastfeeding for six months. In contrast, the omics analyses were initially adjusted for fetal sex, birth weight, and maternal AD status; however, we have expanded the covariate set in the revised manuscript, as detailed in the final paragraph of this response.

For additional confounding factors, we conducted additional analyses adjusting for season of birth, which serves as a proxy for seasonal variation in exposure. The association between $PM_{2.5}$ exposure and AD incidence remained statistically significant after this adjustment. Upon adjustment for postnatal exposure, the relationship between $PM_{2.5}$ exposure across the entire pregnancy and AD incidence remained statistically significant, while the effect of first-trimester exposure was attenuated, as shown in the table below.

In our cohort, we did indeed observe a decline in both $PM_{2.5}$ concentrations and AD prevalence over the study period. Specifically, we found that the AD incidence at age 3, stratified by birth year, demonstrated a decreasing trend over time ($p = 0.01$). When we included the birth year as an additional covariate in our regression model to adjust for potential temporal confounding, the previously observed association between $PM_{2.5}$ exposure and AD incidence was attenuated, and it became statistically non-significant, as shown in the table below. Given that $PM_{2.5}$ concentration is the primary exposure of interest and varies naturally over time, we were concerned that adjusting for the year of measurement—especially since it is inherently correlated with $PM_{2.5}$ levels—could result in over-adjustment. Such an adjustment could inadvertently control for part of the exposure effect itself, potentially obscuring the true relationship between $PM_{2.5}$ exposure and AD risk. Moreover, unless there are additional time-related confounders that independently affect AD prevalence beyond the trends in $PM_{2.5}$ levels, we believe that controlling for the measurement year may not be methodologically appropriate in this context. Therefore, to avoid underestimating the effect of $PM_{2.5}$ exposure, we opted not to adjust for the measurement year in our main analysis. We hope this clarifies our approach, and we sincerely appreciate your valuable feedback.

birth season				
	a OR	95% CI		p value
PM2.5_ entire pregnancy_T1	1.00			
PM2.5_ entire pregnancy_T2	1.43	0.97	2.09	0.07
PM2.5_ entire pregnancy_T3	1.70	1.16	2.50	0.01
PM2.5_ first trimester_T1	1.00			
PM2.5_ first trimester_T2	1.51	0.98	2.33	0.06
PM2.5_ first trimester_T3	1.93	1.19	3.12	0.01
PM2.5_ second trimester_T1	1.00			
PM2.5_ second trimester_T2	1.04	0.68	1.59	0.86
PM2.5_ second trimester_T3	1.45	0.93	2.29	0.11
PM2.5_ third trimester_T1	1.00			
PM2.5_ third trimester_T2	1.26	0.82	1.93	0.28
PM2.5_ third trimester_T3	1.34	0.85	2.13	0.21

postnatal exposure				
	a OR	95% CI		p value
PM2.5_ entire pregnancy_T1	1.00			

PM2.5_ entire pregnancy_T2	1.43	0.96	2.11	0.08
PM2.5_ entire pregnancy_T3	1.71	1.07	2.73	0.03
PM2.5_ first trimester_T1	1.00			
PM2.5_ first trimester_T2	1.21	0.83	1.77	0.31
PM2.5_ first trimester_T3	1.38	0.92	2.08	0.12
PM2.5_ second trimester_T1	1.00			
PM2.5_ second trimester_T2	1.00	0.69	1.45	1.00
PM2.5_ second trimester_T3	1.30	0.86	1.96	0.22
PM2.5_ third trimester_T1	1.00			
PM2.5_ third trimester_T2	1.09	0.76	1.58	0.63
PM2.5_ third trimester_T3	1.03	0.69	1.53	0.90
birth year				
	a OR	95% CI		p value
PM2.5_ entire pregnancy_T1	1.00			
PM2.5_ entire pregnancy_T2	1.17	0.77	1.77	0.46
PM2.5_ entire pregnancy_T3	1.27	0.83	1.95	0.27
PM2.5_ first trimester_T1	1.00			
PM2.5_ first trimester_T2	1.12	0.76	1.64	0.58
PM2.5_ first trimester_T3	1.18	0.78	1.78	0.44
PM2.5_ second trimester_T1	1.00			
PM2.5_ second trimester_T2	0.96	0.66	1.39	0.82
PM2.5_ second trimester_T3	1.19	0.82	1.74	0.36
PM2.5_ third trimester_T1	1.00			
PM2.5_ third trimester_T2	1.18	0.82	1.70	0.38
PM2.5_ third trimester_T3	1.12	0.77	1.62	0.56

For the omics part, many covariate factors except for the three clinical variables (fetal sex, maternal age and maternal AD status) had missing values in the methylome and transcriptome datasets. Nevertheless, to evaluate the potential influence of confounding on our findings particularly those related to FCER1G methylation and expression we conducted additional analyses adjusting for the available covariates: birthweight, secondhand smoke exposure during pregnancy, and maternal cotinine levels. As shown in the table below, the associations for FCER1G-related CpG methylation and gene expression remained statistically significant even after adjusting for these potential confounders.

	**Covariate-adjusted P value**	**Fetal Sex P value**	**Maternal Age P value**	**Maternal AD P value**	**Birth weight P value**	**Maternal ETS P value**	**Maternal Cotinine P value**
**FCER1G (gene expression)**	0.0305	0.3787	0.8704	0.2927	0.4780	0.3121	0.4897
**cg20806175**	0.0021	0.1455	0.5432	0.1732	0.5121	0.3606	0.2329

	Covariate-adjusted P value	Fetal Sex P value	Maternal Age P value	Maternal AD P value	Birth weight P value	Maternal ETS P value	Maternal Cotinine P value
**cg05656486**	0.0015	0.0227	0.3949	0.3313	0.3923	0.6012	0.4699

2. More details are needed for PM2.5 measurement: such as, how dense are the monitor stations? How far in general the address of each studied participant from the nearest monitoring station? Are monitoring data available weekly, daily or hourly? Any records are available to reflect whether the participants changed address during pregnancy?

Thank you for your thoughtful comment regarding the spatial resolution of $PM_{2.5}$ measurements. In our study, land-use regression (LUR) models were developed using data from 37 regulatory air pollution monitoring stations distributed across Seoul, which covers an area of approximately 605 km². This corresponds to an average density of 1 monitoring station per ~16.4 km², reflecting a relatively high spatial resolution for an urban setting.
To assess the proximity between participants' residences and the monitoring network, we calculated the distance from each participant's geocoded residential address to the nearest monitoring station. The median distance was approximately 1.2 km, with an interquartile range (IQR) of 0.8–1.8 km, indicating that most participants lived within a relatively short distance from at least one regulatory monitoring site. Given this density and proximity, along with the use of multiple geographic predictors (e.g., traffic density, land use, spatial trends), we believe that our LUR models provide a robust estimation of long-term $PM_{2.5}$ exposure at the residential level.

Line 747-: Measurement of outdoor $PM_{2.5}$ exposure

Residential exposure to $PM_{2.5}$ was estimated using land-use regression (LUR) models, following a previously validated standardized methodology[59]. Briefly, LUR models were developed using data from 37 regulatory monitoring stations in Seoul, operated by the Korean Ministry of Environment (http://www.airkorea.or.kr/web). Geographic predictor variables—including traffic indicators, land use, topography, and spatial trends—were derived using ArcGIS v9.3 (ESRI, Redlands, CA).
Multiple linear regression models were constructed using a supervised forward stepwise procedure. Final models included predictors such as total road length, traffic intensity on the nearest roads, heavy-duty traffic load, and a spatial trend variable. The models explained 66–81% of the variability in measured concentrations. Specifically, the adjusted R^2 and leave-one-out cross-validation (LOOCV) R^2 values were 0.66 and 0.56 for $PM_{2.5}$[59].
Daily exposure estimates were averaged over pregnancy and the first 3 years of life. When residential addresses changed during the exposure period, updated addresses were used for assessment. Prenatal exposure was assessed by calculating the average pollutant concentrations over the entire pregnancy period and separately for the third trimester. The prenatal period was divided into trimesters as follows: weeks 1–13 (first trimester), 14–27 (second), and 28–40 (third). Postnatal exposure was estimated as the annual average concentration for each year from birth to age 3. Due to the

narrow range of exposure levels, $PM_{2.5}$ was categorized into tertiles to maximize exposure contrast. Analyses compared the middle and highest tertiles with the lowest tertile as the reference group.

3. Preterm birth may play a role in immune development and AD incidence. What is the percentage of preterm birth among the total 986 samples and among the 48 samples for epigenomic and transcriptomic analyses?

Thank you for your insightful comment. In the COCOA study (n=986), babies with a gestational age of less than 37 weeks, as well as those with major congenital anomalies or birth asphyxia requiring oxygen supplementation, are excluded at birth. Consequently, the percentage of preterm births is "zero" in both the total sample of 986 participants and the subset of 48 samples used for epigenomic and transcriptomic analyses.

4. How were the 48 samples selected from the 986 total samples? How high-PM2.5 and low-PM2.5 is defined? Are the population characteristics for the 48 samples and the rest 938 samples comparable?

Thank you for your attention to this critical aspect of our study. We meticulously selected participants to ensure they accurately represented the group's characteristics. Our goal was to conduct the study with a well-defined and appropriate population. We selected 48 placental samples was based on the levels of $PM_{2.5}$ exposure during the first trimester of pregnancy and the diagnosis of AD in offspring. We dichotomized $PM_{2.5}$ exposure using tertiles. The lowest tertile (<24.7µg/m^3) was defined as the low-exposure group, while the highest tertile (>29.21µg/m^3) was defined as the high-exposure group. This helped minimize the inclusion of participants with ambiguous exposure statuses between the low and high groups. Regarding the population characteristics, no significant differences were observed between the 48 samples and the rest 938 samples, aside from the exposure to secondhand smoke during pregnancy and infancy. Other characteristics were comparable between the two groups, as shown in the table below

Line 685-: To further validate these findings, we conducted *in vitro* experiments exposing M2 macrophages to PM, directly assessing its effects on their transcriptional profiles. Third, the relatively small sample size for placental methylome and transcriptome analyses, coupled with the dichotomization of $PM_{2.5}$ exposure, likely reduced statistical power and constrained the generalizability of our results. Nevertheless, clinical characteristics were comparable between the analyzed subset and the full cohort, and rigorous tissue processing ensured data quality. Collection of large quantities of placental tissue is challenging, and comprehensive profiling methylome and transcriptome profiling requires highly specialized tissue processing.

Line 775-: To investigate the mechanisms underlying AD associated with prenatal $PM_{2.5}$ exposure, we selected 48 placental samples and 46 cord blood samples from the COCOA cohort, based on $PM_{2.5}$ exposure levels during the first trimester of pregnancy and the diagnosis of AD in offspring at 2 and 3 years of age.

Line 863-: To examine the impact of prenatal PM exposure on the epigenome and transcriptome and its association with AD, we compared the AD status of offspring between low and high PM exposure levels. $PM_{2.5}$ exposure was dichotomized based on tertile distribution, with the lowest tertile (< 24.70 μg/m³) designated as the low-exposure group and the highest tertile (> 29.21 μg/m³) as the high-exposure group, to accommodate the limited sample size and facilitate robust statistical comparisons.

	938 samples	48 samples	p value
male, n (%)	492 (52.4%)	24 (50.0%)	0.768
Maternal education level, n (%)			
≤ Graduated high school	58 (6.2%)	0 (0%)	0.158
Graduated college	676 (72.1%)	40 (83.3%)	
≥ Graduated postgraduate school	204 (21.7%)	8 (16.7%)	
Parental history of allergic disease, n (%)	462 (49.2%)	28 (58.3%)	0.239
Maternal exposure to environmental tobacco smoke during pregnancy, n (%)	554 (59.1%)	20 (44.4%)	0.042
Infant exposure to environmental tobacco smoke, n (%)	239 (25.5%)	6 (14.0%)	0.025
Breast milk feeding for 6 months, n (%)	369 (58.6%)	24 (58.5%)	1
Birth season, n (%)			0.694
Spring	224 (23.9%)	11 (22.9%)	
Summer	190 (20.2%)	7 (14.6%)	
Autum	234 (25.0%)	15 (31.3%)	
Winter	290 (30.9%)	15 (31.3%)	
Maternal age (years)	32.9 ± 3.5	33.5 ± 3.7	0.268
Maternal BMI (kg/m^2)	20.6 ± 2.5	20.3 ± 2.2	0.297

5. More detailed information is needed for diagnosis of AD. For example, if a kid has a physician diagnosis of AD at age 2 but not age 3, is the kid still classified as the cases with AD? Any other tests have been performed to rule out other skin conditions？ Was IgE or skin tests applied to confirm AD cases?

Thank you for your thoughtful questions. In the COCOA study, AD was determined based on parental reports of physician-diagnosed AD at ages 6 months, 1 year, 2 years, and 3 years. The parental report was based on a positive response to the following written question: "Was your child diagnosed with AD by a doctor in the last 6 months or last year?" If a child was diagnosed with AD at age 2 but not at age 3, they would still be classified as a

case of AD if they had been diagnosed at any point during the study, as we considered AD incidence at any stage during the follow-up period, including at age 3.

During hospital follow-up visits, the presence of AD was clinically diagnosed by pediatric allergy specialists according to the criteria of Hanifin and Rajka. Additionally, no other specific tests, such as IgE or skin tests, were applied to confirm the diagnosis of AD. Total IgE levels were measured at both age 1 and age 3; however, skin prick test results were not available for age 3, because the children were too young to undergo the skin prick test procedure. The diagnosis was solely based on the clinical evaluation and the parental report, which was aligned with the diagnostic criteria used by the specialists.

Line 740-: AD was determined through parental reports of physician-diagnosed AD at ages 6 months, 1 year, 2 years, and 3 years. The parental report was based on a positive response to the following written question: "Was your child diagnosed with AD by a doctor in the last 6 months or last year?" During hospital follow-up visits, the presence of AD was clinically diagnosed by pediatric allergy specialists according to the criteria established by Hanifin and Rajka. (Hanifin JM, Rajka G. Diagnositic features of atopic dermatitis. Acta Derm Venereol (Stockh) 1980;92(Suppl):44–7) Children diagnosed with AD at any point during the study were considered to have AD incidence at age 3.

6. Identification of DMCs specific to PM2.5 did not account for multiple testing;

In our omics analyses, multiple testing correction using the Benjamini-Hochberg (BH) method did not yield statistically significant results. Previous studies have reported that FDR correction methods may reduce specificity and be unsuitable for analyses involving low-dimensional data or small sample sizes [PMID: 29499647, PMID: 16777905]. Therefore, we adopted an alternative approach based on nominal P values and implemented a two-step statistical filtering strategy using (1) the Wilcoxon rank-sum test and (2) ordinary least squares (linear regression), retaining only candidates that satisfied the significance threshold of $P < 0.05$ in both tests. Additionally, in our methylation data generated from EPIC microarray platform, we considered not only statistical significance but also biological effect size. Specifically, we selected CpG sites with absolute methylation differences ($|\Delta\beta|$) of at least 0.02, since smaller changes around 1% are likely to result from technical variability and lack biological relevance [PMID: 34863305]. For the pathway analyses, we performed with FDR correction and the results have been updated accordingly in the revised manuscript.

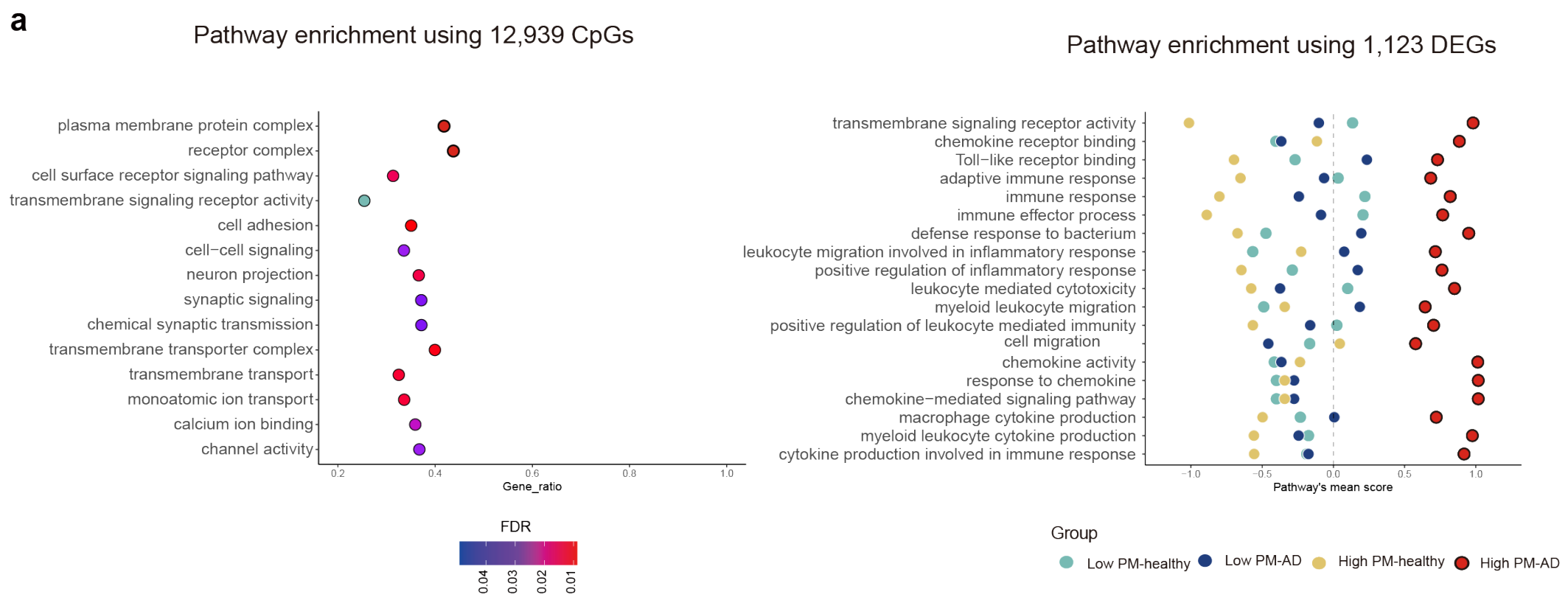

Extended Data Fig. 1a. Pathway analysis of differentially methylated CpG sites (DMCs) and differentially expressed genes (DEGs). Left: Pathway analysis of 12,939 DMCs was performed using the Gometh tool. The x-axis shows the gene ratio, representing the proportion of pathway-related genes relative to the total number of genes in a given pathway. Pathways with an FDR (Benjamini–Hochberg) < 0.05 were included. Right: The GSVA tool was used for pathway analysis of 1,123 DEGs based on the GO, KEGG, and Reactome databases. The x-axis displays the mean pathway scores across groups. Pathways with an FDR (Benjamini–Hochberg) < 0.05 were included. Statistical analyses included over-representation analysis for Gene Ontology (GO) and KEGG terms (left panel) and the Wilcoxon rank-sum test to compare the High PM-AD group with other groups (right panel).

Line 135-: These samples were grouped based on $PM_{2.5}$ exposure levels and AD status of offspring (Fig. 1b, see Methods). To capture exposure-specific mechanisms, we excluded differentially methylated CpGs (DMCs) and differentially expressed genes (DEGs) overlapping with the low-exposure group, thereby isolating signatures unique to high-exposure AD cases. This filtering strategy was guided by the hypothesis that widespread epigenetic perturbations induced by high PM2.5 exposure drive AD pathogenesis, whereas AD under low exposure may arise from genetic predisposition. Using this approach, we identified 12,939 differentially methylated CpGs (DMCs) and 1,123 DEGs in the placentae of AD-affected offspring with high $PM_{2.5}$ exposure (Supplementary Table 4).

In Figure 1c, why comparison between two groups was shown for pathway enrichment using 12939 CpGs, while comparison among four groups was shown for pathway enrichment using 1.123 DECs? At the left panel, does gene ratio (the x axis) actually represents the difference of gene ratio between the two groups? More annotation is needed for broader readers.

We thank the reviewer for pointing out the need for additional clarification. For pathway analysis of the methylation data, we used the Gometh tool, which detects pathway-level perturbations but does not provide information on directionality. Accordingly, we focused on pathways epigenetically disturbed in AD-related samples under high PM exposure, as outlined in the analysis pipeline (Fig. 2b). Our study design specifically aimed to identify AD-specific

molecular signatures induced by high PM exposure by comparing high-PM/healthy and high-PM/AD groups. We hypothesized that high PM exposure induces broad epigenetic alterations, a subset of which drive AD, whereas AD cases under low PM exposure are more likely attributable to genetic predisposition (also addressed in our response to Comment #15 from Reviewer 3).

As the reviewer suggested, in the left panel of Fig. 1c, the x-axis represents the ratio of DMC-related genes within each pathway, relative to the total number of genes in that pathway, as noted in the figure legend. In contrast, for the transcriptomic data, pathway enrichment was assessed using GSVA, which calculates sample-level enrichment scores and captures directionality. We reported mean activity scores across groups, highlighting that the most pronounced differences occurred between high-PM/healthy and high-PM/AD groups, in line with the observed epigenetic alterations.

7. When t-tests (line 214) were applied to compare difference of a continuous variable between two groups, did the authors check the distribution of the analyzed variables? Or any transformation was applied to approximate normal distribution?

We thank the reviewer for this important point. We initially applied the t-test under the assumption of normal distribution; however, normality testing using the Shapiro–Wilk method confirmed that the data did not follow a normal distribution. Accordingly, we corrected the statistical analyses by applying the Wilcoxon rank-sum test, a nonparametric approach, for the results shown in Figures 2c and 2d. With this adjustment, the results in Figure 2c remained statistically significant, while those in Figure 2d showed partial statistical significance but retained the overall trend of progressive FCER1G upregulation from healthy to lesional AD samples. These revised statistical results have been updated in the corresponding figures.

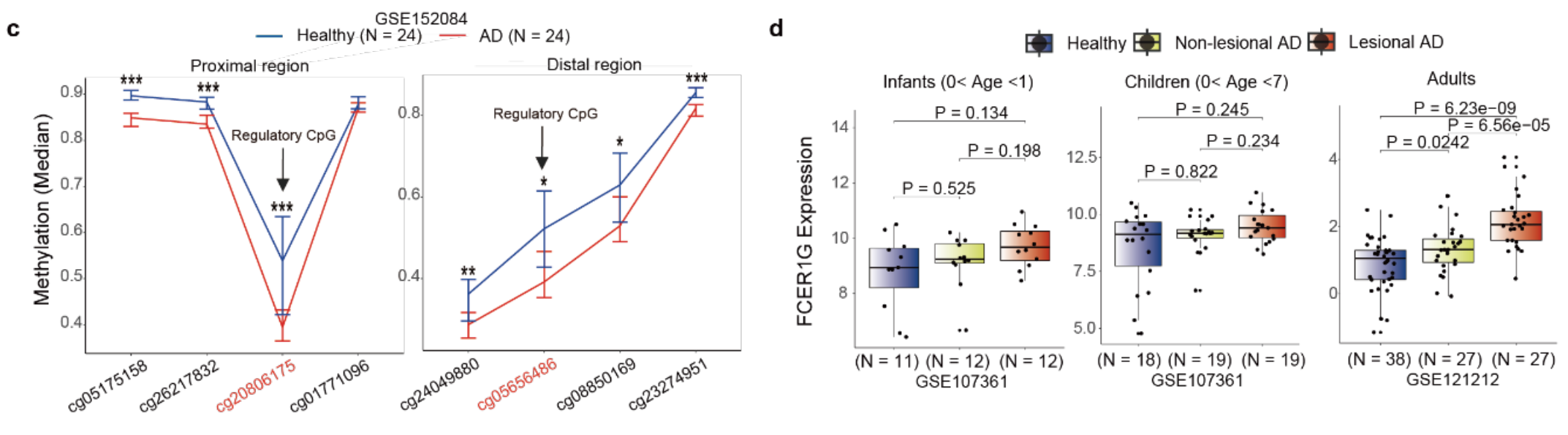

8. When analyzing epigenome using DNA from cord blood, cell types should be adjusted as covariates.

Extended Data Fig 1d and 1e were about the result of epigenomics analysis from cord blood, but as described in the figure, the associations in cord blood were not statistically significant even before cell type adjustment; therefore,

we present these results only as supporting evidence to illustrate the trend recapitulated from placental samples.

9. Given that most of the findings from this study are based on association analyses or correlation analyses which is observational, the authors may not be able to strengthen that the identified hypomethylations or altered gene expressions are induced or caused by high PM2.5 exposure (such as, Line 182: “.. between altered methylation induced by high PM2.5 exposure.).

We thank the reviewer for this important comment. To strengthen causal inference, we performed a Mendelian randomization (MR) analysis to assess the relationship between PM2.5-associated DNA methylation and AD risk. Specifically, we examined two regulatory CpG loci (cg05656486 and cg20806175) previously identified as hypomethylated under high PM2.5 exposure. The MR analysis demonstrated that reduced methylation at both loci was significantly associated with increased AD risk, and this association remained robust after adjusting for key covariates, including PM2.5 exposure levels. These findings support a potential causal role of PM2.5-induced hypomethylation in disease pathogenesis. Nevertheless, we acknowledge that the MR analysis was based on a limited sample size (n = 24), and further validation in larger cohorts will be required. This result has been incorporated into the revised manuscript.

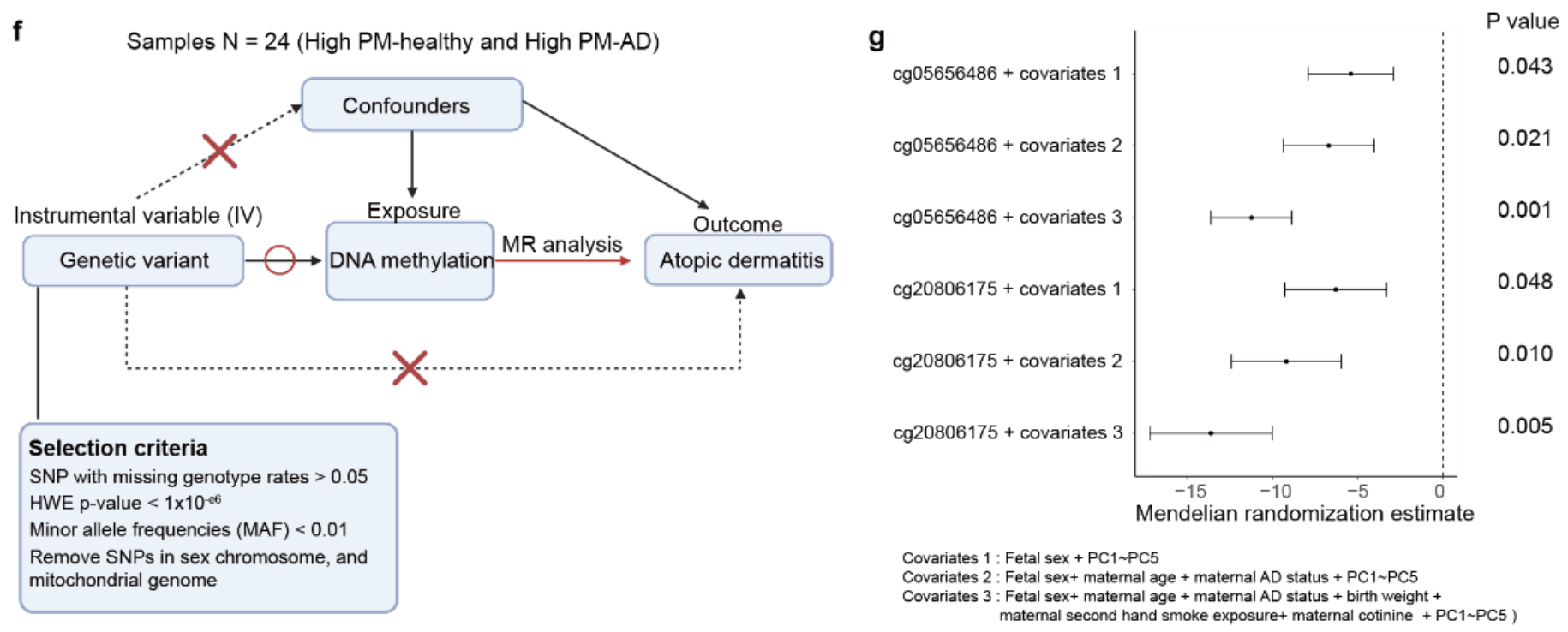

Extended Data Fig. 1f and 1g. Mendelian Randomization (MR) analysis to assess the causal role of DNA methylation in AD under high prenatal PM exposure. a, Schematic of the MR analytic pipeline. The analysis was performed using placental samples from the high prenatal PM exposure group in our cohort. b, Error bar plot showing the MR estimates for the CpG sites cg05656486 and cg20806175. P-values corresponding to each estimate are shown to the right of the plot. Covariate combinations included in the analysis are detailed below. The statistic was calculated using the ivreg function in R.

Line 201-: The regulatory CpG sites, located 13,693 bp upstream and within an intronic region of FCER1G, were located within the same topologically associated domain and overlapped with active histone marks (H3K27ac, H3K4me1, and H3K4me3) in placental tissue, but not in the maternal basal plate of the maternal side (Fig. 2e). To assess the potential causal role of these epigenetic modifications, we applied a one-sample Mendelian Randomization (MR) approach using 24 placental samples. This analysis revealed that hypomethylation at cg05656486 and cg20806175 was significantly associated with increased risk of infantile AD, suggesting a direct

contribution of these loci to disease susceptibility (Extended Data Figs. 1f, 1g; Supplementary Table 5). Notably, these associations remained statistically robust after adjusting for PM2.5 exposure levels and other key covariates.

Line 695-: To mitigate these constraints, we leveraged Mendelian randomization analysis to validate the causal relationship between $PM_{2.5}$-associated methylation loci and AD status, incorporating sex, maternal age, maternal AD status, birth weight, maternal secondhand smoke exposure and maternal cotinine as a covariate.

18. It would be helpful to draw a schematic diagram on causal path on pm to methylation change to immune alteration to ad to help readers follow the reasoning.

Reviewer #1 (Remarks to the Author):

In the revised manuscript, the authors have modified the main claims and the title, now proposing that prenatal exposure to particulate matter (PM) promotes epigenetic alterations associated with FCER1G expression in placental macrophages, which may subsequently lead to the re-emergence of FCER1Ghi M2 macrophages in the skin, contributing to the development of atopic dermatitis (AD). The authors have strengthened their argument by demonstrating epigenetic changes at the FCER1G locus in both PBMCs from AD patients and in PM-exposed placental tissues. Additionally, the inclusion of a mendelian randomization analysis provides further, albeit indirect, support for an association between these epigenetic alterations and AD development. While these additions partially strengthen the proposed link between epigenetic changes and the increased frequency of FCER1Ghi M2 macrophages in AD, the causal relationship between prenatal PM exposure and the emergence of these M2 macrophages remains unclear. Specifically, Figures 1–3 support the notion that high levels of PM exposure may epigenetically regulate FCER1G expression. In contrast, Figures 4–7 focus primarily on the presence of FCER1Ghi M2 macrophages in AD lesional skin, without a clear connection to PM exposure levels. This shift in focus makes it difficult to trace the causal path from prenatal exposure to disease phenotype. Moreover, the authors have not adequately addressed earlier concerns about the lack of experimental validation for their single-cell RNA-seq findings, which are based primarily on publicly available datasets. This reliance limits the robustness of their claims.
In addition to these general concerns, I would like to raise the following specific points:

We appreciate the reviewer's thoughtful general assessment and fully acknowledge the remaining concerns regarding causal continuity, mechanistic linkage, and experimental validation. Specifically, we thank the reviewer for highlighting the perceived discontinuity between Figures 1–3 and Figures 4–7, and we agree that the transition from prenatal PM exposure to the emergence of $FCER1G^+$ M2 macrophages in AD required clearer justification.

1. Clarifying the causal linkage between PM exposure and $FCER1G^+$ macrophages

In our study, PM exposure during the prenatal stage was treated as a representative environmental factor capable of inducing epigenetic alterations. Our primary goal was to identify the molecular and cellular consequences of such alterations rather than to evaluate PM exposure as a continuous variable throughout life. Accordingly, Figures 1–3 focus on defining $PM_{2.5}$-induced epigenetic changes that drive FCER1G overexpression in the placenta, while Figures 4–7 explore how this FCER1G activation re-emerges in analogous macrophage populations within AD lesions. This developmental recapitulation implies that $FCER1G^+$ macrophages induced by early-life environmental exposure may persist or reappear later as AD-associated macrophages.

To make this conceptual continuity explicit, we have revised the manuscript to include a bridging rationale before describing the results of Figures 4–7,

clarifying how the prenatal epigenetic event mechanistically links to the re-emergence of FCER1G+ M2 macrophages in adult AD skin.

Line 315-: Prenatal exposure to $PM_{2.5}$ was conceptualized as an early-life environmental stressor capable of inducing epigenetic programming in developing immune cells. To delineate the downstream cellular consequences of this programming, we traced the reactivation of FCER1G-driven macrophage networks across developmental stages, testing whether these programs re-emerge in AD. This developmental recapitulation suggests a mechanistic continuum linking prenatal environmental exposure to postnatal disease phenotypes, even in the absence of direct postnatal exposure data. To define the cellular specificity of FCER1G within the placental immune niche, we analyzed single-cell transcriptomic data from consortium-based placental atlas datasets[24]. This analysis confirmed that FCER1G was most prominently expressed in Hofbauer cells, the fetal macrophages located in the villous stroma at all gestational stages (Extended Data Fig. 3a).

2. Experimental validation of PM–epigenetic–macrophage linkage
We agree with the reviewer that experimental validation is critical to strengthen this connection. In the revised manuscript, we added in vitro evidence showing that PM exposure in macrophages directly induces FCER1G expression along with its downstream targets (CYBB, MRC1, and MS4A4A), which are known AD-associated factors. Moreover, we newly demonstrate that PM exposure also induces epigenetic alterations in FCER1G regulatory regions, reinforcing the mechanistic link between PM exposure, epigenetic dysregulation, and macrophage activation. Although in vivo cellular validation was limited by the availability of placenta or skin samples with documented PM exposure, these experimental results provide a complementary validation of our data-driven and single-cell multi-omics findings. Detailed information is provided in Response 3.

3. Sample limitation and interpretative context
We acknowledge that performing single-cell transcriptomic or methylomic profiling on placental or skin tissues with directly quantified PM exposure is technically and logistically challenging because of limited sample availability and the lack of biobanked specimens with detailed environmental exposure metadata. We now emphasize this limitation in the Discussion and note that our integrative strategy—combining cohort-based exposure data, multi-omic analyses, and complementary experimental systems—represents a feasible and biologically coherent framework for investigating links between early-life exposure and later immune dysfunction.

We further agree that experimental validation is important for strengthening the connection between our single-cell transcriptomic findings and the proposed mechanism. As an alternative approach, we tried to assess the expression of FCER1G and its downstream targets using skin tape-strip samples of non-lesional skin from COCOA birth cohort. However, RNA yield from these samples was insufficient for reliable quantification of target gene expression, likely reflecting technical limitations inherent to tape-strip collection and processing of stratum corneum–derived material. Consequently, further direct validation using the skin biopsy samples from our

cohort was not feasible. We hope you understand this situation. Our single-cell RNA-seq analyses indicate that an FCER1G^{+} M2 macrophage transcriptional program is initially primed in placental macrophages and subsequently reappears in AD skin lesions, consistent with a developmental recapitulation model rather than direct lineage persistence. Because direct PM exposure data are not available for adult AD skin samples, we did not interpret these observations as evidence of PM-specific effects in adulthood.

To support the specificity of this re-emerging macrophage program in AD skin, we analyzed the largest available AD skin single-cell consortium dataset, comprising 710,704 cells from 91 AD patients and 62 healthy controls. This analysis confirmed that representative components of the FCER1G-centered program, including CYBB, MRC1, and MS4A4A, are selectively enriched in FCER1G^{+} M2 macrophages and show a stepwise increase from healthy to lesional AD skin, consistent with our single-cell trajectory and spatial transcriptomic analyses. Convergent evidence from independent transcriptomic studies further supports elevated expression of FCER1G and its downstream signaling components in AD lesions.

Importantly, because most AD skin datasets lack prenatal PM exposure information, we interpret these findings as evidence for the recurrence of a shared FCER1G-centered transcriptional program across developmental contexts, rather than as direct validation of PM-specific effects in adult skin. Accordingly, we have revised the manuscript to clearly frame these results within a developmental recapitulation model and to avoid overstatement of causality.

1. Although the authors have revised some aspects of their "epigenetic scar" hypothesis, the term still appears in lines 609–610. While they demonstrate that similar epigenetic changes are observed in both PM-exposed placental tissues and PBMCs from AD patients, they do not provide evidence that these changes persist postnatally or are retained specifically in FCER1Ghi M2 macrophages. As such, the continued use of the term “epigenetic scar” is not supported by the data and should be revised.

We sincerely thank the reviewer for this careful suggestion. We agree that our data do not directly support the use of the term “epigenetic scar.” Accordingly, we have replaced it with “epigenetic alteration” in lines 628-629 of the revised manuscript, ensuring consistency throughout the text.

Line 626 -: To further elucidate the cellular context, we identified M2 macrophages enriched with FCER1G overexpression within the placentae of offspring with AD. Integrative analysis across multiple cohorts of AD development, including our own, revealed the re-emergence of this epigenetic alteration in FCER1G^{+} M2 macrophages.

2. In Fig. 2c and Extended Fig. 2h, the authors show FCER1G hypomethylation and increased chromatin accessibility in AD patients compared to healthy controls. However, these analyses were conducted across all AD patients regardless of PM exposure level. This is inconsistent with the claim that such epigenetic changes are characteristic of high PM exposure. The authors should clarify whether these results reflect PM-specific effects or general features of AD.

We thank the reviewer for this important clarification request. Our intention in presenting these datasets was not to imply that all FCER1G epigenetic changes in AD arise from PM exposure, but rather to demonstrate that the specific PM-associated epigenetic signature we identified in placentae—hypomethylation at FCER1G regulatory loci—reappears in AD patients, even in independent cohorts without exposure data. This finding supports the idea that both prenatal PM-induced and AD-associated epigenetic alteration converge on the same FCER1G regulatory regions. To clarify this distinction in the manuscript, we have now explicitly stated that:

1. Figure 2c and Extended Fig. 2h represent AD-general epigenetic features from external datasets that do not include PM exposure variables.
2. The overlap between PM-induced loci and AD-general loci suggests that FCER1G regulatory regions are epigenetically vulnerable, and PM exposure during prenatal development may act as an initiating event that later re-emerges within AD pathogenesis.

This clarification has been added to the revised manuscript.

Line 272-: Furthermore, our analysis of public ATAC-seq data (GEO accession GSE255396) demonstrated increased chromatin accessibility at these FCER1G regulatory regions in PBMC monocytes from AD patients (Extended Data Fig. 2h). Although these external datasets lack prenatal PM exposure information, the recurrence of FCER1G hypomethylation and chromatin accessibility at key regulatory elements underscores the likelihood of reactivation of early-life epigenetic programming in AD.

To directly address the distinction, we now include in vitro PM-exposure experiments showing that PM exposure induces FCER1G upregulation together with epigenetic alterations at the same regulatory regions identified in our PM-stratified placenta dataset. These experimental data provide evidence for a PM-responsive mechanism that is not captured in the AD-general datasets. Taken together, the public datasets reflect AD-general epigenetic patterns, whereas the PM-exposure experiments support PM-specific induction of the same regulatory alterations. Detailed descriptions of these experiments have been added in Response 3.

3. Figures 6i–j show that particulate matter can alter the phenotype of M2-like macrophages in vitro. However, if the central hypothesis is that PM exposure induces epigenetic modifications that persist and re-emerge in skin macrophages, then it would be important to demonstrate whether such

epigenetic alterations also occur in these in vitro experiments. Without this validation, the link between PM-induced epigenetic changes and the observed macrophage phenotype remains speculative.

We appreciate the reviewer's comment and fully agree that directly linking PM-induced phenotypic changes to corresponding epigenetic alterations is essential to support our central hypothesis. To address this, we expanded our in vitro PM-exposure experiments to include DNA methylation profiling. Using the same FCER1G regulatory CpG loci identified in our PM-stratified placental cohort (Fig. 1b), we found that diesel PM exposure induced significant hypomethylation at a distal regulatory locus in THP-1–derived M2 macrophages compared with untreated controls. This epigenetic change coincided with increased FCER1G expression following diesel PM exposure, providing functional evidence that PM-induced epigenetic modification directly contributes to the $FCER1G^{+}$ M2-like macrophage phenotype observed in Figures 6i–j.

These results provide direct mechanistic evidence that PM exposure can drive coordinated epigenetic and transcriptional activation of the $FCER1G^{+}$ M2-like program, thereby strengthening the biological plausibility of our developmental recapitulation model. Details of these experiments have been incorporated into the revised manuscript.

Line 490-: Correlation analysis across individual samples confirmed a strong positive association between FCER1G module activity and ROS signaling across all developmental stages. This cellular signaling cascade could be imprinted by early-stage PM exposure. To validate this, we performed *in vitro* experiments assessing M2 macrophage transcriptomic and epigenomic dynamics under urban and diesel $PM_{2.5}$ exposure conditions. In THP-1-derived M2 cells, FCER1G mRNA expression was significantly increased under both $PM_{2.5}$ conditions (Fig. 6i and 6j). In parallel, DNA methylome profiling revealed that PM exposure induced hypomethylation at the same distal FCER1G regulatory regions identified in high PM-exposed placentae, coinciding with FCER1G transcriptional activation.

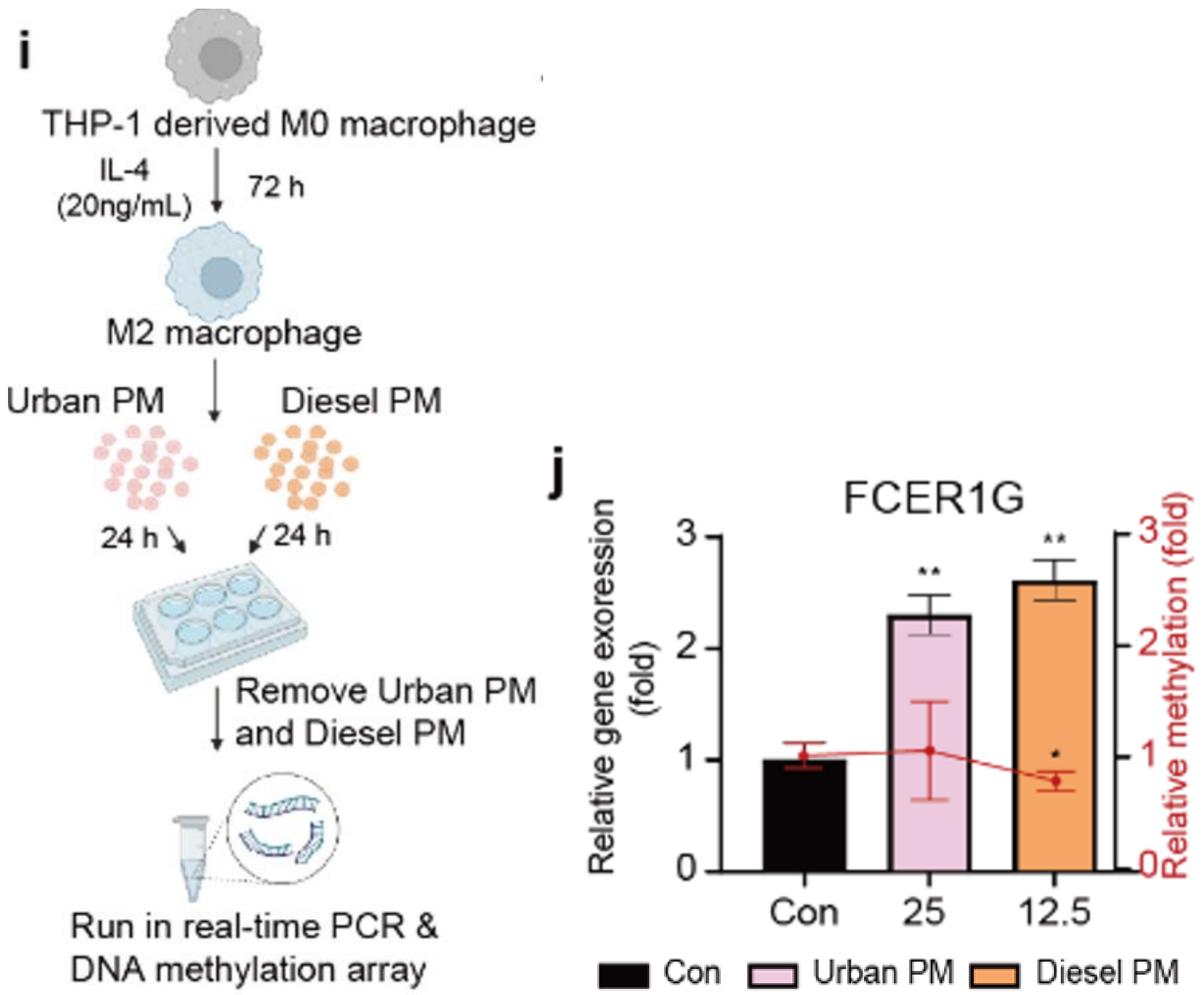

Fig. 6i. Schematic of the experimental design to assess $PM_{2.5}$ effects on M2 macrophages. **Fig. 6j.** Relative expression of FCER1G, CYBB, MRC1, and MS4A4A under urban PM (25 µg/mL) and diesel PM (12.5 µg/mL) conditions in M2 macrophages. Control (Con): M2 macrophages were not exposed to PM. The relative methylation level of a distal regulatory CpG (cg05656486, located -13,639 bp from the TSS) associated with FCER1G is indicated by a red line with error bars.

Line 896-: DNA methylation analysis in M2 macrophages

DNA samples were assessed for quality using a NanoDrop® ND-2000 UV–Vis spectrophotometer and further evaluated by agarose gel electrophoresis. Only samples showing intact genomic DNA without evidence of degradation were selected for downstream analysis. Genomic DNA was quantified using the Quant-iT PicoGreen assay (Invitrogen) and diluted to a final concentration of 50 ng/µL. All samples were bisulfite-converted according to the manufacturer's protocol using the Zymo EZ DNA Methylation Kit. DNA methylation profiling was performed using the Infinium MethylationEPIC BeadChip. Beta values were obtained after background correction and normalization using the ENmix pipeline, followed by quantile normalization.

4. As previously noted, the authors do not provide experimental validation for key findings derived from scRNA-seq analyses. While prior studies may support increased M2 macrophages in AD in general, these study does not offer direct evidence showing an increase in Hofbauer cells in high PM-exposed placentas or an increase in FCER1Ghi macrophages in the skin of

AD patients specifically with high PM exposure. These validations are essential to substantiate the proposed causal link between prenatal PM exposure and macrophage-mediated inflammation in AD.

We sincerely appreciate the reviewer's comment and fully agree that direct experimental validation would further strengthen the link between prenatal PM exposure and the re-emergence of $FCER1G^{+}$ macrophages in AD. For placental tissues, we emphasize that performing transcriptomics on placentae with documented PM exposure is technically and logistically constrained, given the rarity of such specimens and the lack of bio-banked placentae with recorded environmental exposure metadata. Accordingly, we applied cellular inference approaches to PM-stratified placental bulk datasets using Hofbauer cell gene signatures derived from public single-cell placental atlases, representing the most feasible strategy under current limitations.

For AD skin, we newly analyzed the largest available AD skin single-cell consortium dataset (710,704 cells from 91 AD patients and 62 healthy controls) and confirmed that representative gene signatures of $FCER1G^{+}$ M2 macrophages, including CYBB, MRC1, and MS4A4A, are selectively enriched within $FCER1G^{+}$ M2 macrophages and exhibit a stepwise increase from healthy to lesional AD skin. These observations are consistent with prior reports and are supported by our single-cell trajectory and spatial transcriptomic analyses. In addition, convergent evidence from independent transcriptomic studies has demonstrated elevated expression of FCER1G and its downstream signaling components in lesional AD skin, as well as increased FCER1G expression in the skin of PM-exposed mice in an ovalbumin-induced atopic dermatitis model.

Importantly, most AD skin datasets do not contain prenatal PM exposure information, and we therefore do not interpret these findings as evidence of PM-specific effects in adulthood. Rather, the recurrence of a shared FCER1G-centered transcriptional program across placental macrophages and AD-associated skin macrophages supports a developmental recapitulation model, in which early-life epigenetic priming may predispose to the reactivation of this macrophage state under inflammatory conditions. We have revised the manuscript to clearly frame this interpretation and to avoid overstatement of causality.

Line 315-: Prenatal exposure to $PM_{2.5}$ was conceptualized as an early-life environmental stressor capable of inducing epigenetic programming in developing immune cells. To delineate the downstream cellular consequences of this programming, we traced the reactivation of FCER1G-driven macrophage networks across developmental stages, testing whether these programs re-emerge in AD. This developmental recapitulation suggests a mechanistic continuum linking prenatal environmental exposure to postnatal disease phenotypes, even in the absence of direct postnatal exposure data. To define the cellular specificity of FCER1G within the placental immune niche, we analyzed single-cell transcriptomic data from consortium-based placental atlas datasets[24]. This analysis confirmed that FCER1G was most prominently expressed in Hofbauer cells, the fetal macrophages located in the villous stroma at all gestational stages (Extended Data Fig. 3a).

Line 574-: Further histopathological analysis confirmed a spatially coordinated increase in $FCER1G^+$ M2 macrophages and ROS signaling factors across different skin samples, demonstrating a consistent enrichment pattern from healthy to lesional AD skin (Fig. 7e). The selective enrichment of the $FCER1G^+$ M2 macrophage gene signature in AD skin was further validated using the largest available AD skin single-cell consortium dataset, comprising 710,704 cells from 91 AD patients and 62 healthy controls[39]. This independent dataset demonstrated a stepwise increase in $FCER1G^+$ M2 macrophages from healthy to lesional AD skin (Extended Data Fig. 11).

Line 1096-: scRNA-seq data in this study have been deposited in the Gene Expression Omnibus under accession code GSE171381 (term placenta data) and the ArrayExpressdatabase under accession code E-MTAB-6701 (early placenta data), E-MTAB-7407 (healthy fetal skin data), and E-MTAB-8142 (healthy and AD adult data). Additional scRNA-seq data for healthy and AD adult skin are available via the Single Cell Portal under accession code SCP2738.

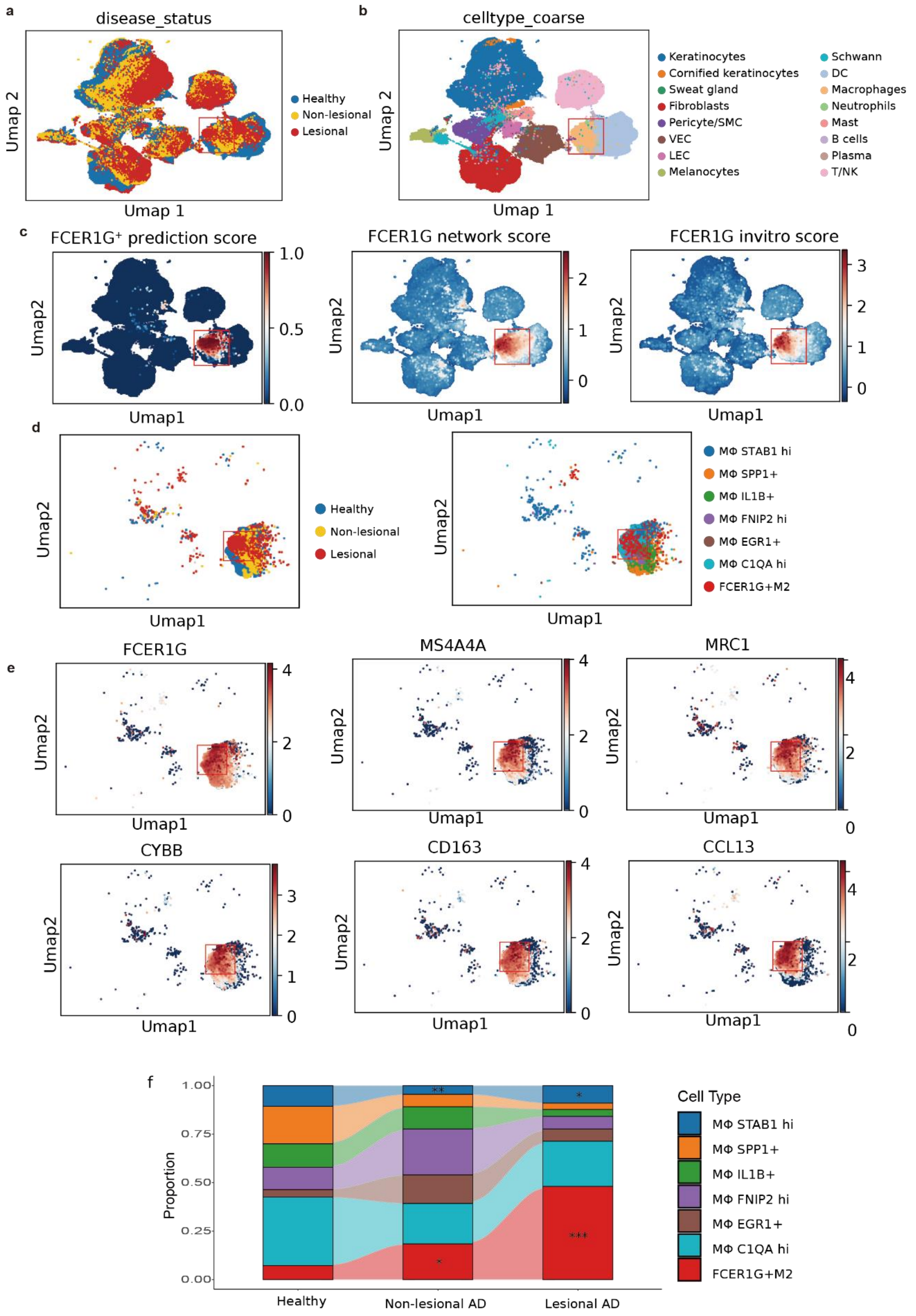

Extended Data Fig. 11| Identification of FCER1G⁺ M2 macrophages and their representative gene signatures within the AD skin atlas by Fiskin et al. a, b, UMAP plots display the distribution of cells colored by atopic dermatitis (AD) status (**a**) and by each cell state as defined in the Fiskin et al. skin atlas (**b**). **c,** UMAP projections showing FCER1G⁺ M2 macrophage–associated gene signature scores across the cellular landscape. The FCER1G⁺ M2 prediction score was calculated using the FindTransferAnchors function in Seurat (left). The FCER1G network score

was derived from the inferred regulatory network centered on FCER1G (Fig. 6f; middle). The FCER1G score was computed using scanpy.tl.score_genes based on the expression of FCER1G, MS4A4A, MRC1, CYBB, CD163, and CCL13 (right). MS4A4A, MRC1, and CYBB were selected based on in vitro PM exposure validation experiments (Fig. 6i, j), CD163 was included as a canonical M2 macrophage marker, and CCL13 was identified as an $FCER1G^{+}$ M2–associated ligand through cell–cell interaction analysis. **d,** UMAP visualization restricted to macrophages, showing AD status and corresponding macrophage subclusters. **e,** Expression levels of representative $FCER1G^{+}$ M2 marker genes across macrophages. **f,** Proportional composition of macrophage subtypes in the Fiskin atlas, compared across AD disease states. Statistical analyses included generalized linear modeling with a quasibinomial distribution for proportion comparisons and likelihood ratio tests assessing differences of Lesional and Non-lesional AD groups relative to Healthy (f). $^{*}P \le 0.05$, $^{**}P \le 0.001$, $^{***}P \le 0.0001$

Line 342-: At the gene regulatory level, co-expression network analysis revealed a conserved gene module, including FCER1G, that was overexpressed across developmental stages in placental macrophages, fetal skin macrophages, and adult M2 macrophages (Fig. 4d). Notably, several genes within this conserved module, including CD163, F13A1, and MRC1, have been experimentally validated to be overexpressed in AD skin lesions through immunohistochemistry[28,29].

Line 480-: Among the genes directly interacting with FCER1G, CD68, CYBB, MRC1, and MS4A4A were identified as key players involved in NADPH oxidase-mediated ROS signaling, a pathway implicated in macrophage-driven inflammatory responses and increased risk of AD[33–35] (Fig. 6h, left). Consistently, elevated expression of FCER1G, CYBB, and MS4A4A has been reported in tape-stripped atopic dermatitis lesional skin[36].

Line 499-: Additionally, FCER1G-associated signaling molecules, such as CYBB, MRC1, and MS4A4A, were significantly overexpressed under diesel $PM_{2.5}$ exposure. Experimental evidence from PM_{10} exposure in an OVA-induced atopic dermatitis mouse model further supports these in vitro findings, demonstrating significantly increased FCER1G expression in the skin of PM_{10}-exposed mice[37] .

Reviewer #2 (Remarks to the Author):

The authors have sufficiently and extensively responded to the reviewers' comments, and have thus clarified previously unclear approaches and results of their study.

We sincerely thank the reviewer for their positive and encouraging evaluation. We appreciate the constructive feedback provided throughout the review process, which has greatly improved the clarity and quality of our manuscript.

Reviewer #3 (Remarks to the Author):

Thank you for your thoughtful revisions and responses to my previous comments. The revisions have considerably enhanced the manuscript.

I have one remaining concern regarding the definition of “low exposure” for PM2.5. The cut-off used (<24.70 μg/m³) is significantly higher than health-based standards set by the WHO, US EPA, and Korea. Thus, “low exposure” group may represent relatively high exposure compared to guidelines or other air pollution studies. I would recommend noting this limitation in the discussion, as well as the implications for the interpretation and generalizability of your findings.

We thank the reviewer for this important point. We acknowledge that the cut-off used for the “low exposure” group (<24.70 μg/m³) is indeed higher than the WHO, US EPA, and Korean air quality guidelines. Our categorization was based on the distribution of $PM_{2.5}$ concentrations within our cohort, which reflects real-world urban exposure levels in the study region. We have now clarified this limitation in the Discussion section, emphasizing that the “low exposure” group in our study may still represent moderate exposure relative to global health standards. We also discuss that this may affect the generalizability of our findings to populations with lower background $PM_{2.5}$ levels.

Line 911-: $PM_{2.5}$ exposure during the first trimester was dichotomized based on tertile distribution, with the lowest tertile (< 24.70 μg/m³) designated as the low-exposure group and the highest tertile (> 29.21 μg/m³) as the high-exposure group, to accommodate the limited sample size and facilitate robust statistical comparisons. These cut-offs reflect the actual distribution of urban $PM_{2.5}$ exposure in our study population, rather than strict health-based guidelines.

Line 709-: Third, the relatively small sample size for placental methylome and transcriptome analyses, coupled with the dichotomization of $PM_{2.5}$ exposure, likely reduced statistical power and constrained the generalizability of our results. It is also important to note that the low-exposure group in our cohort (<24.70 μg/m³) exceeded WHO, US EPA, and Korean guideline thresholds, indicating that even the lower category reflects moderate urban exposure and should be considered when generalizing to populations with cleaner air.

Reviewer #4 (Remarks to the Author):

The authors have provided detailed point-by-point responses to my comments. However, I remain concerned about several important issues. Specifically, this study may have low statistical power and lacks a validation study. In the context of omics analyses, the absence of appropriate multiple-testing correction raises the likelihood that some reported associations may be due to chance.

We thank the reviewer for the careful reassessment of our work. As detailed in our responses, we have clarified these points and strengthened the manuscript accordingly.

As outlined in Response 1, we fully recognize the limitations imposed by the small sample size on the MR analysis. We therefore provided a detailed description of SNP identification, IV filtering, and diagnostic testing to ensure transparency. Although exploratory, the MR models that passed weak-instrument and overidentification tests support the directionality of our primary findings.

Regarding multiple-testing correction and the exposure window, Response 2 clarifies that the first trimester was selected for mechanistic omics analyses based on strong biological plausibility and prior evidence of early gestational immune programming, while we now clearly acknowledge that this window did not retain statistical significance after correction.

To directly address potential temporal confounding, Response 3 includes new analyses demonstrating declining $PM_{2.5}$ levels over time and consistent associations between whole-pregnancy exposure and AD across year-stratified groups, indicating that year-of-measurement does not account for the observed effects.

Finally, we now explicitly note in the third subsection of the Discussion that omics-derived associations should be interpreted with caution and are emphasized only when supported by multi-layer convergence across methylation, transcription, single-cell analyses, in vitro assays, and exploratory MR estimates, a framework that appropriately reflects the statistical constraints while underscoring the biological coherence of the central findings.

1. MR analyses and IV selection: The Mendelian Randomization (MR) analyses were conducted in 24 samples, which raises concerns about the reliability of the findings. Moreover, the validity of MR analysis critically depends on the strength and appropriateness of the genetic variants used as instrumental variables (IVs). Could the authors clarify in detail how the SNPs were identified and selected as IVs for each CpG site in this study?

We appreciate the reviewer's careful evaluation of our MR analyses and fully agree that the limited sample size imposes constraints on the robustness of causal inference. Consistent with this concern, we now emphasize in the Discussion that these MR results should be interpreted cautiously and require validation in larger, independent cohorts.

To address the reviewer's question regarding IV selection, we have added a detailed description of the SNP identification and filtering procedure. The goal of the MR analysis was to estimate whether methylation at each CpG site exerts a causal effect on infantile AD. For each CpG, SNPs associated with methylation levels were identified using linear regression under three covariate sets:
(1) child's sex;
(2) child's sex, parental age, and maternal AD; and
(3) the variables in set 2 plus birth weight, maternal secondhand smoke exposure, or maternal cotinine level.
Principal components (PC1–PC5) were included in all models to account for population structure. SNPs associated with methylation at raw $p < 1\times10^{-4}$ were retained as candidate instruments. To ensure instrument independence, linkage disequilibrium clumping was performed in PLINK using --clump-p1 1e-4, --clump-kb 1000, and --clump-r2 0.2. SNPs associated with clinical outcomes or covariates (raw $p < 0.05$) were removed to satisfy MR assumptions. The final MR estimation was conducted using two-stage least squares in the ivreg R package (version 0.6.4).

We also performed diagnostic tests to evaluate the strength and validity of the instruments (table attached below). The weak-instrument test rejected the null hypothesis for cg05656486 (covariate set 2) and for cg20806175 (covariate sets 1–3), indicating sufficient instrument strength in these models. Overidentification was assessed using the Sargan test; except for cg20806175 under covariate set 3, all models satisfied the overidentification restrictions. Nevertheless, we acknowledge that given the small sample size, full verification of instrument validity is limited. All methodological details have been added to the revised Methods, and the limitations are now clearly noted in the Discussion.

Diagnostic tests for instrumental variable in Mendelian Randomization

Exposure	Estimate	Std. Error	t value	Pr(>|t|)	Number of snps	Weak instruments	Weak instruments *P*	Sargan	Sargan *P*
cg05656486 + covariates 1	-5.405	2.515	-2.149	0.043	15	2.864	0.082	9.011	0.83
cg20806175 + covariates 1	-6.287	3.006	-2.091	0.049	12	8.625	0.001	5.203	0.921
cg05656486 + covariates 2	-6.703	2.666	-2.515	0.021	11	6.38	0.005	5.198	0.878
cg20806175 + covariates 2	-9.193	3.23	-2.846	0.01	10	13.015	0	14.727	0.099
cg05656486 + covariates 3	-11.244	2.381	-4.722	0.001	14	NA	NA	16	0.249
cg20806175 + covariates 3	-13.629	3.599	-3.787	0.005	4	12.671	0.008	8.729	0.033

Line 718-: Additionally, we acknowledge that we did not directly assess the epigenetic profiles of dermal macrophages in adult AD skin tissue, which limits our ability to fully validate the maintenance of placental epigenetic alterations in affected skin. To address these limitations, we implemented complementary strategies including one-sample Mendelian randomization, leveraging placental genotypes to assess the causal relationship between FCER1G methylation and AD. Although exploratory due to sample size, these results aligned with our primary findings.

Line 839-: One-sample bidirectional Mendelian randomization analysis
To investigate the causal relationship between altered methylation induced by high PM and AD status, one-sample Mendelian randomization (MR) analysis was conducted. We assessed causality using high-PM2.5 exposed methylation pattern as exposure variables (X) and clinical indicators (i.e, AD or control) as outcome variables (Y). To assess the causal effect of methylation levels on clinical outcomes, SNPs associated with each CpG site were identified using linear regression at a significance threshold of raw p-value < 1e–4. Three covariate sets were considered: set 1 included the child's sex; set 2 included the child's sex, parental age, and maternal AD status; and set 3 additionally included birth weight, maternal secondhand smoke exposure, or maternal cotinine level. Principal components (PC1–PC5) were also included as covariates. Independent SNPs were identified using clumping analysis in PLINK, with following set parameters: raw p-value (--clump-p1 1e-04), genomic distance (--clump-kb 1000), and linkage disequilibrium (LD, --clump-r2 0.2). SNPs significantly associated with clinical indicators or confounders (raw-p value < 5x10-2) were excluded to satisfy the key assumptions of MR. Subsequently, a two-stage least square regression (TSLS) analysis was implemented using the R package ivreg (version 0.6.4).

2. The authors stated in their response that, after adjustment for multiple testing, the association between first-trimester PM2.5 exposure and AD incidence was no longer statistically significant (P = 0.118), while the association for exposure across the entire pregnancy remained significant (P = 0.01). Given this result, why did the authors still choose to use first-trimester PM2.5 exposure in the omics analyses, rather than focusing on the exposure window that remained significant?

We thank the reviewer for this insightful comment. We acknowledge that, after adjustment for multiple testing, the association between first-trimester PM2.5 exposure and AD incidence was no longer statistically significant (P = 0.118), whereas the association across the entire pregnancy remained significant (P = 0.01).

Our focus on first trimester $PM_{2.5}$ exposure in the omics analyses was driven by biological plausibility and mechanistic relevance rather than statistical significance alone. The first trimester represents a critical window for fetal immune maturation and epigenetic programming, during which environmental perturbations can induce durable alterations in immune function (PMID: 20678227; PMID: 29979694). This developmental period also coincides with early epidermal and dermal differentiation and the initial establishment of

cutaneous immune cell populations (PMID: 32173857). Although the epidemiologic association did not remain statistically significant after correction for multiple testing, the first trimester remains a window of strong mechanistic interest. This interpretation is further supported by prior epidemiologic studies reporting associations between first trimester $PM_{2.5}$ exposure and increased risk of atopic dermatitis (PMID: 33432626; PMID: 32006726; PMID: 32971247).

We agree with the reviewer that this distinction should be clearly acknowledged. Accordingly, we have added statements in the Methods to clarify our rationale for selecting the first-trimester exposure window for omics analyses.

Line 801-: To investigate the mechanisms underlying AD associated with prenatal $PM_{2.5}$ exposure, we selected 48 placental samples and 46 cord blood samples from the COCOA cohort, based on $PM_{2.5}$ exposure levels during the first trimester of pregnancy and the diagnosis of AD in offspring at 2 and 3 years of age. The first trimester was chosen based on biological plausibility, as this period encompasses critical stages of fetal immune maturation and epigenetic programming, while also marking the initiation of epidermal and dermal development as well as the establishment of cutaneous immune cell populations[62,63,64].

3. Confounding by year of measurement: The author mentioned in the response that both AD incidence and PM2.5 levels may vary by calendar year. Therefore, confounding by year of measurement remains a concern. At a minimum, the authors should illustrate how PM2.5 levels changed across years (e.g., with descriptive plots) and consider stratified analyses by year (e.g., grouping years into two or three categories) to assess whether the observed associations are robust to this potential confounding factor.

We appreciate the reviewer's insightful comment regarding potential confounding by year of measurement. To address this concern, we performed additional analyses to characterize temporal trends in $PM_{2.5}$ levels and to evaluate whether year-to-year variation affected the observed associations. Descriptive analyses showed a significant decline in $PM_{2.5}$ concentrations across the study period (p for trend < 0.001), consistent with national reductions in ambient particulate matter.

To further evaluate the potential impact of temporal confounding, we conducted stratified analyses by calendar year, grouping the cohort into early and late study periods. Across both strata, the association between whole-pregnancy $PM_{2.5}$ exposure and AD incidence remained consistent, whereas the association for first-trimester exposure did not reach statistical significance in either subgroup. These findings indicate that, although $PM_{2.5}$ concentrations declined over time, the central conclusion of our study—namely, the relationship between cumulative prenatal $PM_{2.5}$ exposure and AD risk—remains robust and is unlikely to be explained by year-of-measurement

effects. The detailed results of these additional temporal analyses are provided below:

Temporal trend of $PM_{2.5}$ concentrations during the study period

	year	mean	95% CI	
PM2.5_ entire pregnancy	2007	34.54	30.88	38.2
	2008	31.92	30.63	33.22
	2009	30.6	29.77	31.43
	2010	28.52	27.84	29.19
	2011	29.16	28.53	29.79
	2012	25.22	24.48	25.95
	2013	24.12	23.35	24.89
	2014	23.99	23.11	24.86
PM2.5_ first trimester	2007	44.89	40.6	49.18
	2008	35	33.07	36.93
	2009	33.58	32.37	34.79
	2010	29.88	29.12	30.63
	2011	28.37	27.33	29.4
	2012	26.13	25.06	27.19
	2013	23.59	22.47	24.7
	2014	22.1	21.02	23.18
PM2.5_ second trimester	2007	26.88	23.59	30.16
	2008	30.1	28.41	31.79
	2009	29.79	28.75	30.84
	2010	28.19	27.27	29.1
	2011	29.91	28.92	30.89
	2012	24.41	23.43	25.38
	2013	23.71	22.66	24.77
	2014	21.09	19.89	22.28
PM2.5_ third trimester	2007	33.07	29.09	37.04
	2008	30.78	28.94	32.62
	2009	28.29	27.02	29.55
	2010	27.33	26.31	28.35
	2011	29.14	28.02	30.26
	2012	25.33	24.32	26.34
	2013	25.22	24.23	26.21
	2014	29.98	28.86	31.1

Temporal trend of $PM_{2.5}$ concentrations during the study period. The line shows the mean, and the error bars indicate the 95% CI.

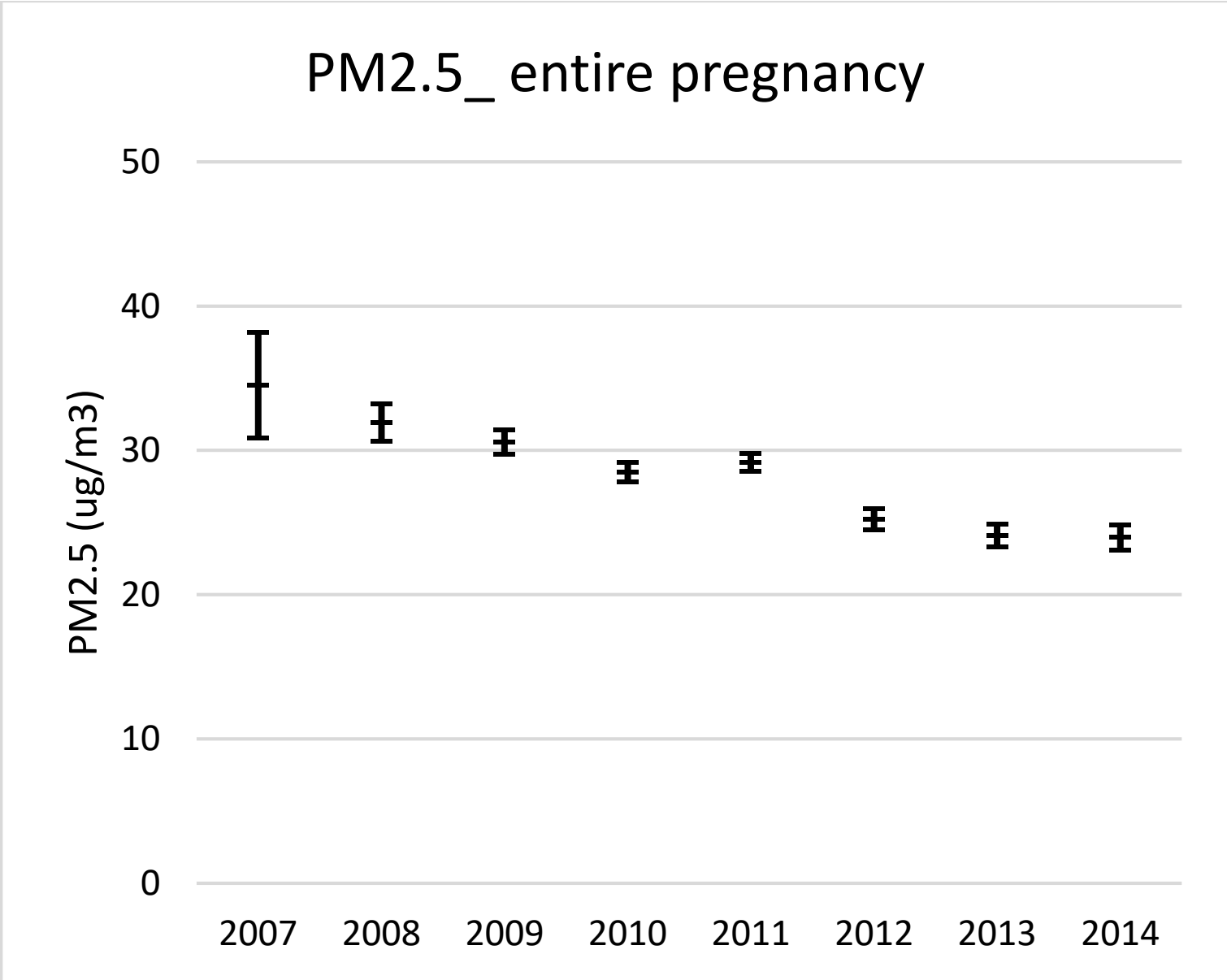

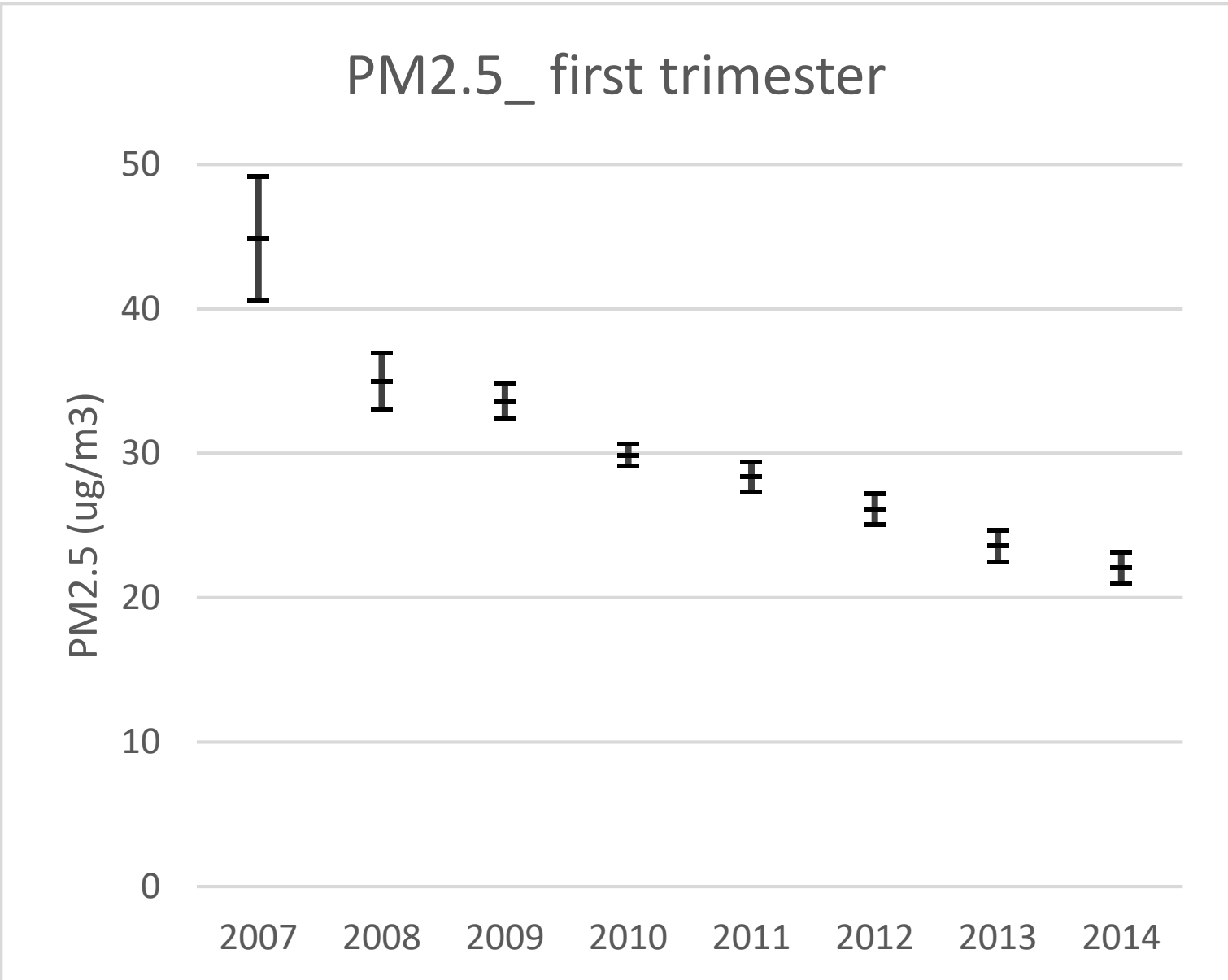

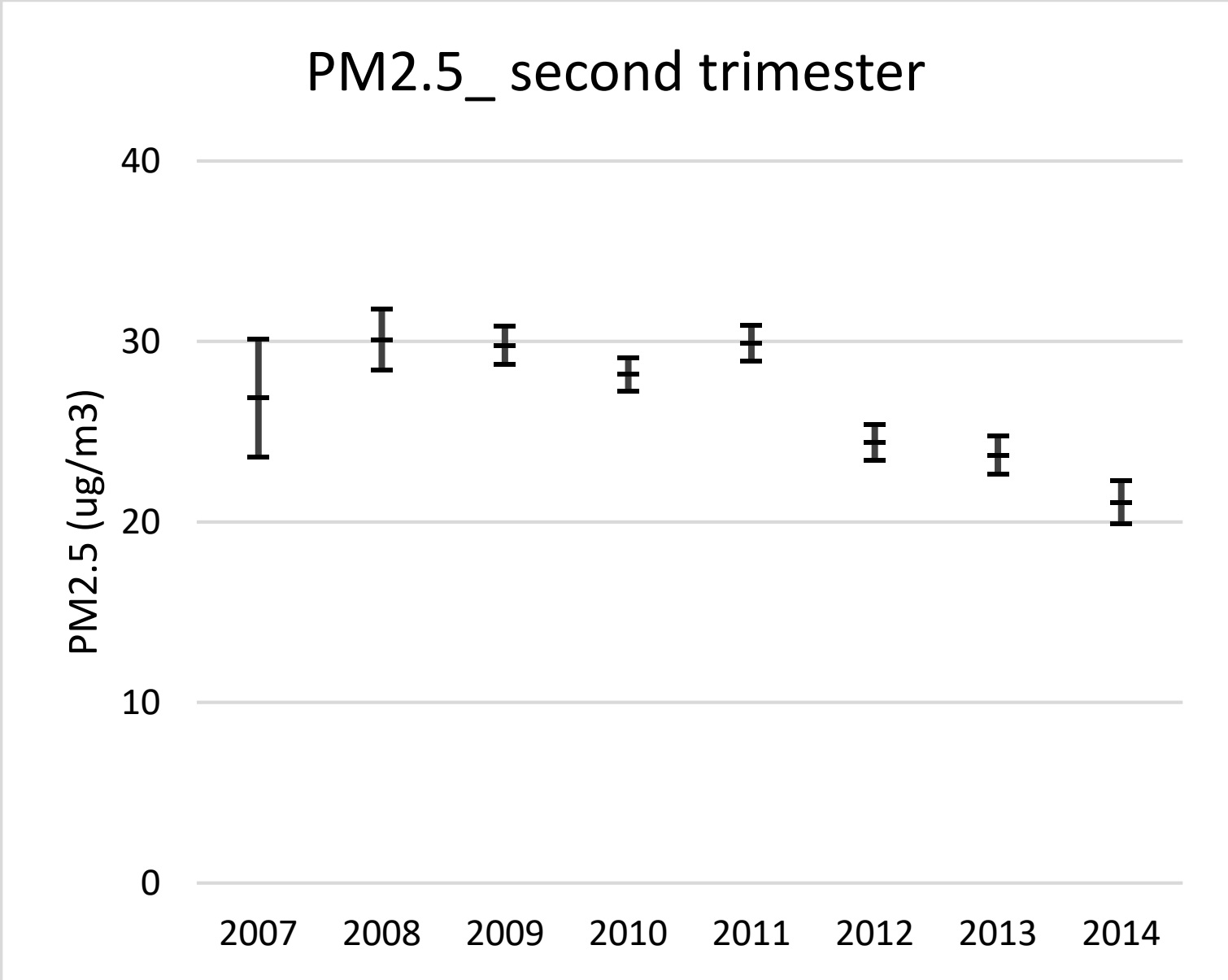

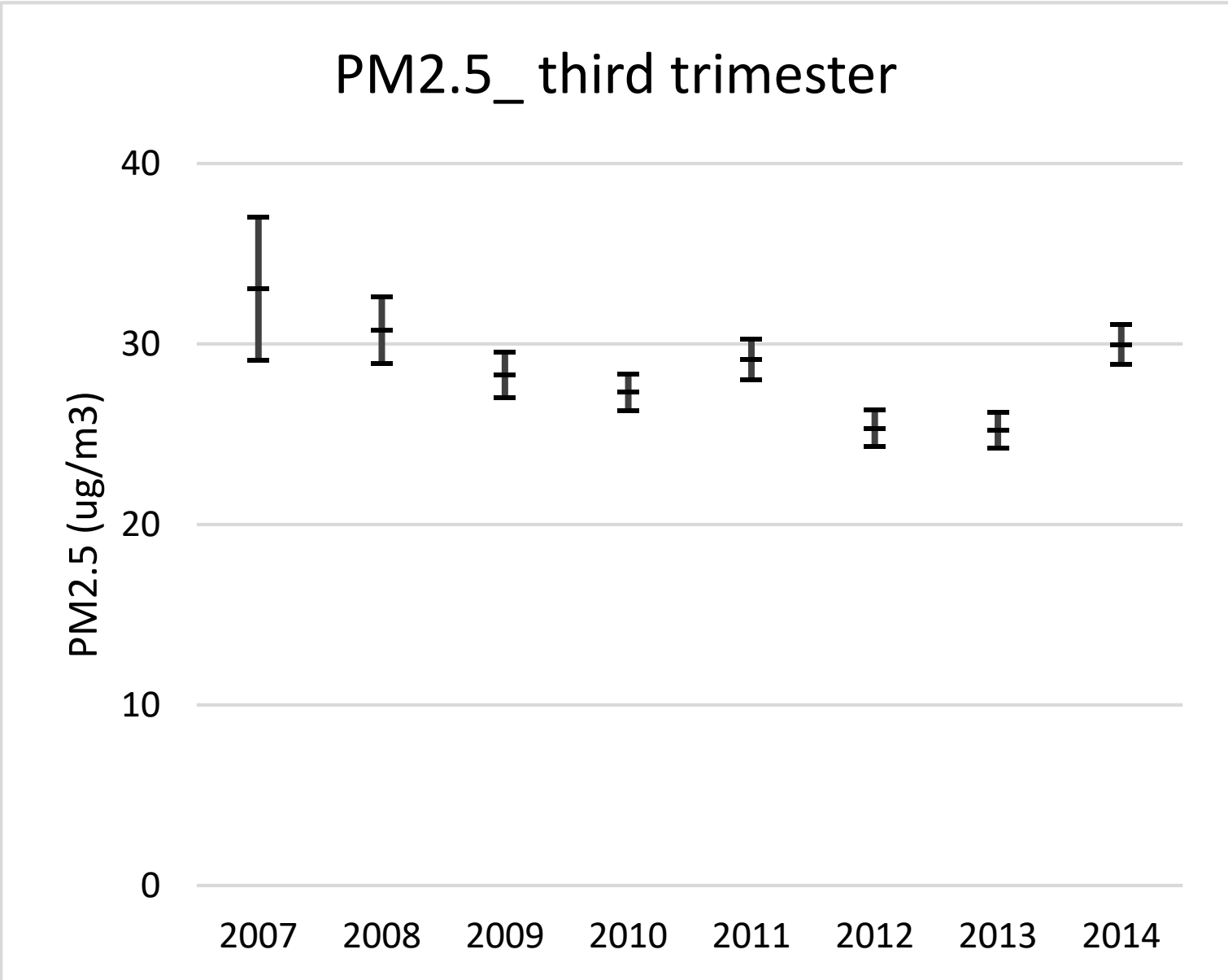

Stratified analysis of association between PM2.5 exposure and AD incidence by study period

	earlier years (2007-2010)				later years (2011-2014)			
	a OR	95% CI		p value	a OR	95% CI		p value
PM2.5_ entire pregnancy_T1	1.00				1.00			
PM2.5_ entire pregnancy_T2	2.39	1.06	5.40	0.036	1.14	0.69	1.89	0.600
PM2.5_ entire pregnancy_T3	2.41	1.09	5.34	0.030	1.69	1.03	2.77	0.039
PM2.5_ first trimester_T1	1.00				1.00			
PM2.5_ first trimester_T2	1.04	0.49	2.18	0.923	1.20	0.75	1.91	0.444
PM2.5_ first trimester_T3	1.16	0.57	2.36	0.682	1.61	0.95	2.74	0.077
PM2.5_ second trimester_T1	1.00				1.00			
PM2.5_ second trimester_T2	1.09	0.60	1.98	0.767	0.98	0.61	1.58	0.928
PM2.5_ second trimester_T3	1.32	0.76	2.30	0.330	1.49	0.90	2.47	0.124
PM2.5_ third trimester_T1	1.00				1.00			

PM2.5_ third trimester_T2	1.09	0.62	1.92	0.764	1.13	0.69	1.85	0.625
PM2.5_ third trimester_T3	1.27	0.76	2.15	0.365	1.06	0.62	1.79	0.844

We added a description of this issue to the revised Discussion section.

Line 693-: Among these potential confounders, we adjusted for season of birth, as well as NO_2 and ozone concentrations, and the significant association between $PM_{2.5}$ and AD remained significant. $PM_{2.5}$ exposure during early childhood did not affect the risk of AD, and even after adjusting for this exposure, the impact of prenatal $PM_{2.5}$ exposure on AD remained significant, highlighting the importance of prenatal exposure. Stratified analyses across exposure years further confirmed the consistency of these findings, despite potential temporal variation in $PM_{2.5}$ levels.

Reviewer #1 (Remarks to the Author):

The authors have adequately addressed the major concerns raised previously. However, this reviewer has one remaining minor concern regarding the description in the Abstract.
As noted in Concern #1, the authors demonstrate that epigenetic alterations are observed in both PM-exposed placental tissues and PBMCs from patients with AD. However, the study does not provide direct evidence demonstrating the persistence of these alterations across developmental stages, nor their association with FCER1Ghi M2 macrophages in AD skin. Rather, the persistence of epigenetic alterations appears to be inferred from the presence of FCER1Ghi macrophages in those tissues.
In lines 67–69, the statement that "This epigenetic alteration was recapitulated across developmental stages in independent cohorts, as demonstrated through single-cell RNA sequencing of 404,169 cells from placenta, fetal, and adult skin samples" may therefore be misleading. This wording could give readers the impression that the epigenetic alteration itself was directly observed in placenta as well as fetal and adult skin within this study, which is not supported by the presented data.
The authors should revise this description and similar statements throughout the manuscript, to more clearly distinguish between experimentally demonstrated findings and interpretations or predictions.

We thank the reviewer for this insightful comment and for emphasizing the importance of clearly distinguishing experimentally demonstrated findings from interpretative inferences.

As noted, we did not perform epigenetic profiling in adult AD skin or dermal macrophages. Direct methylation analyses were conducted in placenta and cord blood (Fig. 2b and Extended Data Fig. 1d) and independently validated in pediatric PBMCs (GSE152084; Fig. 2c), demonstrating FCER1G hypomethylation in these compartments. We did not assess methylation in fetal or adult skin macrophages. Accordingly, we revised the Abstract to clearly separate demonstrated epigenetic findings in placenta and PBMCs from transcriptomic evidence across developmental stages. Statements implying direct recapitulation of the epigenetic alteration were replaced with wording describing enrichment of FCER1G-high macrophage populations identified by single-cell RNA sequencing.

Line 65 -: Integrated epigenomic and transcriptomic profiling of 48 placentae identified $PM_{2.5}$-associated hypomethylation driving FCER1G overexpression, predominantly in fetal macrophages. This epigenetic signature was recapitulated in peripheral blood from an independent pediatric AD cohort. Single-cell RNA sequencing analyses of 404,169 cells from independent placental, fetal skin, and adult skin datasets revealed consistent enrichment of FCER1G-high macrophage populations across developmental stages, supporting reactivation of a shared macrophage program.

In addition, we carefully reviewed the entire manuscript to eliminate any overstatement implying direct persistence of the same epigenetic mark across tissues. Specifically, we revised statements in the Introduction, Results, and Discussion to replace language suggesting sustained or re-emergent epigenetic alteration with terminology reflecting transcriptional reactivation, network-level conservation, or association with later FCER1G dysregulation. These revisions ensure a clear distinction between experimentally demonstrated methylation findings and transcriptomic or network-based inferences across developmental stages.

Line 110 -: These limitations underscore the need for more extensive research to elucidate how such epigenetic changes influence immune development and disease susceptibility in offspring.
Our study aims to investigate the mechanisms linking prenatal $PM_{2.5}$ exposure to AD by examining whether prenatal epigenetic alterations are associated with transcriptional programs that reappear across developmental stages.

Line 181 -: **Integrative analysis identifying disrupted epigenetic regulation of FCER1G in the placenta and its transcriptional reactivation in AD**

Line 597 -: These results establish a spatially coordinated interaction between FCER1G+ M2 macrophages and VE cells via the CCL13–ACKR1 axis, suggesting a mechanistic role in ROS-driven Th2 inflammation during AD progression.
Collectively, integrative single-cell and spatial transcriptomic analyses together with histopathological evidence support a model in which prenatal PM2.5 exposure induces epigenetic alteration in placental macrophages that is associated with reactivation of $FCER1G^{+}$ M2 macrophage programs in AD skin, driving ROS-mediated signaling and Th2 inflammation during AD progression, as summarized in the proposed causal pathway (Fig. 7f).

Line 635 -: To further elucidate the cellular context, we identified M2 macrophages enriched with FCER1G overexpression within the placentae of offspring with AD.
Integrative analysis across multiple cohorts of AD development, including our own, revealed reactivation of FCER1G-associated transcriptional and network programs in $FCER1G^{+}$ M2 macrophages.

Line 744 -: Future studies with larger sample sizes are warranted and should consider utilizing continuous exposure metrics or exploring alternative classification methods to more accurately capture the complexity of exposure–response relationships.
In conclusion, this study provides the most comprehensive evidence to date linking first-trimester $PM_{2.5}$ exposure to an increased risk of AD in young children, identifying a prenatal epigenetic alteration in FCER1G that is associated with persistent dysregulation of FCER1G expression during development.

Furthermore, in the Discussion, we explicitly acknowledge this limitation by stating that we did not directly assess the epigenetic profiles of dermal

macrophages in adult AD skin, which limits our ability to confirm maintenance of placental epigenetic alterations in affected skin.

Line 725 -: Collection of large quantities of placental tissue is challenging, and comprehensive profiling methylome and transcriptome profiling requires highly specialized tissue processing. Additionally, we acknowledge that we did not directly assess the epigenetic profiles of dermal macrophages in adult AD skin tissue, which limits our ability to fully validate the maintenance of placental epigenetic alterations in affected skin. To address these limitations, we implemented complementary strategies including one-sample Mendelian randomization, leveraging placental genotypes to assess the causal relationship between FCER1G methylation and AD.

We appreciate the reviewer's careful reading and believe the revised manuscript now more accurately reflects the scope and strength of our manuscript.

Reviewer #4 (Remarks to the Author):

The revised manuscript has been improved as expected. I still have one concern
The authors aim to investigate mechanisms linking prenatal PM2.5 exposure to AD by examining epigenetic alterations that are sustained and re-emerge across developmental stages. Their hypothesis is that prenatal PM2.5 exposure induces specific epigenetic modifications, primarily hypomethylation of immune-related genes in certain placental immune cell types. However, the analytical approach does not directly test this hypothesis. Rather than first identifying epigenetic modifications that are significantly associated with PM2.5 exposure, the authors excluded differentially methylated CpGs (DMCs) and differentially expressed genes (DEGs) overlapping with the low-exposure group, thereby focusing on epigenetic signatures associated with AD only among children with high PM2.5 exposure. While this strategy identifies epigenetic markers associated with AD in the context of high exposure, it does not provide direct evidence that these markers are induced by PM2.5 exposure itself. Consequently, the current analyses do not fully support the interpretation that PM2.5 exposure induces the observed epigenetic modifications; rather, they suggest that the associations between epigenetic modifications and AD development may be modified by PM2.5 exposure, with such associations observed only among children with high exposure. I would suggest that the author add a table or figure indicating the associations of the identified primary signature (DMC and DEG) with PM2.5 (which can be treated as continuous or binary variables) in figure 1 using all samples (n=986), to support that these reported signatures are “induced” by high PM2.5 exposure.

We sincerely thank the reviewer for this important comment. We agree that our original analysis primarily identified epigenetic signatures associated with AD under high prenatal PM2.5 exposure and did not explicitly demonstrate that these signatures are induced by PM2.5 itself.

First, methylome and transcriptome profiling were performed in 48 placental samples, whereas the full cohort of 986 children was used for epidemiological analyses. To address the concern directly, we reanalyzed all 48 placental samples without stratification.

A simple regression model (methylation ~ PM2.5) did not uniformly capture a negative correlation across all samples. We interpret this as reflecting substantial inter-individual variability in baseline placental methylation states. Increasing evidence suggests that prenatal environmental exposures do not necessarily induce uniform epigenetic shifts; rather, they reshape high-variance baseline states toward directional patterns that become transcriptionally apparent in specific disease contexts [PMID: 33341587, PMID: 36249271].

Consistent with this framework, we modeled methylation levels of the identified regulatory CpGs and FCER1G expression as a function of PM2.5

exposure (continuous and binary) and its interaction with AD status (methylation or expression ~ PM2.5 × AD status). This approach tests whether PM-associated methylation shifts differ according to disease outcome. Using this interaction model, both regulatory CpGs (cg20806175 and cg05656486) showed negative standardized beta coefficients under high PM exposure in the context of AD across multiple exposure definitions, including the entire pregnancy, first trimester, second trimester, and high versus low exposure categories. In parallel, FCER1G expression showed a concordant increase with higher PM2.5 exposure. These results indicate that hypomethylation and overexpression of FCER1G are preferentially derived under high PM conditions among individuals who develop AD, rather than representing a uniform exposure effect across all samples.

Importantly, our in vitro PM2.5 exposure experiments (Fig. 6h–j) independently demonstrate that PM2.5 directly induces hypomethylation at the distal regulatory CpG and increases FCER1G expression in M2 macrophages. These data provide mechanistic support that the identified epigenetic signature is PM-inducible.

We have clarified the statistical framework, included the interaction-based results (Extended Data Fig. 1d), and moderated causal language accordingly. Together, the revised analyses support a model in which prenatal PM2.5 exposure contributes to context-dependent epigenetic reprogramming of FCER1G regulatory loci that becomes functionally coupled to AD development. We appreciate the reviewer's suggestion, which has improved the clarity and rigor of our conclusions.

Line 195-: In contrast, no methylation changes were observed in the low $PM_{2.5}$ exposure group (Fig. 2b, bottom). Across all placental samples, linear regression did not reveal a uniform inverse association between $PM_{2.5}$ exposure and CpG methylation, consistent with substantial inter-individual variability[18,19]. However, inclusion of an interaction term between $PM_{2.5}$ exposure and AD status uncovered a significant exposure-dependent hypomethylation effect restricted to individuals who developed AD, concomitant with elevated FCER1G expression (Extended Data Fig. 1d). These data support a context-specific epigenetic response to PM2.5 rather than a generalized exposure effect.

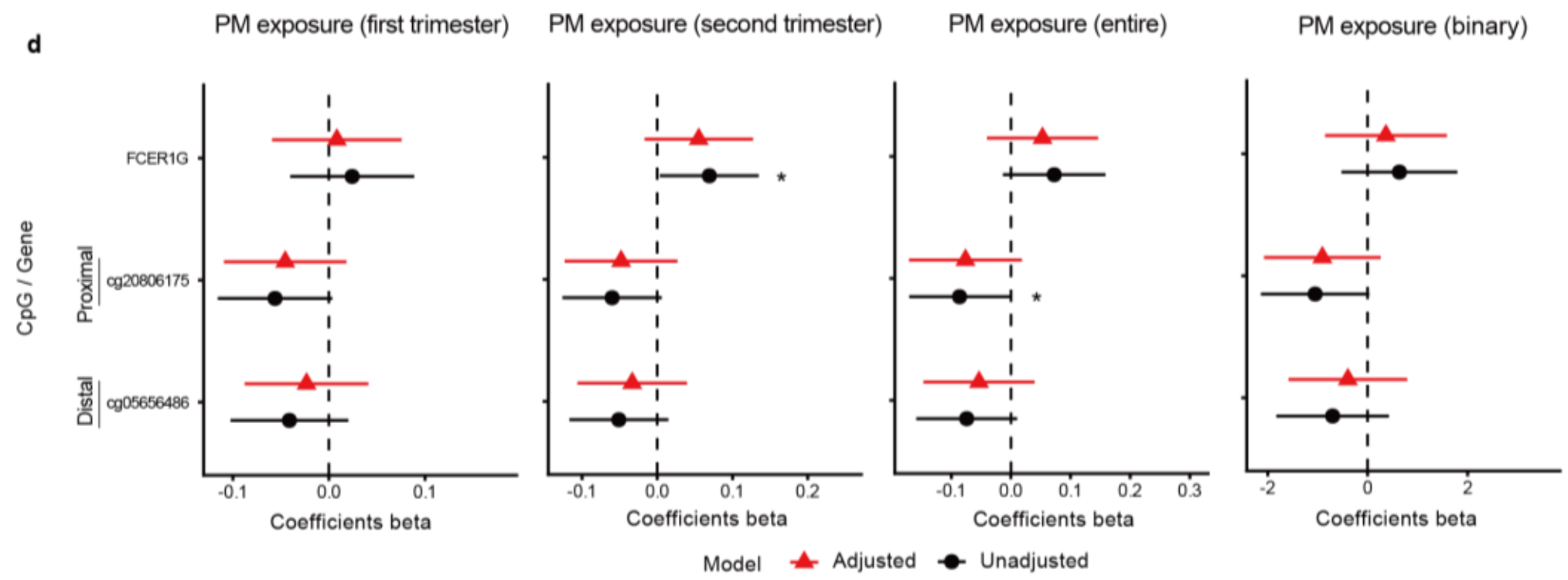

Extended Data Fig. 1d| Forest plots represent the interaction coefficients derived

from linear regression models (Target ~ PM exposure × AD status) for FCER1G expression and DNA methylation at the proximal and distal CpG sites in 48 placental samples. PM exposure was evaluated sequentially for the first trimester, second trimester, entire pregnancy, and as a binary variable (high vs. low). Error bars indicate standardized β coefficients with 95% confidence intervals. Adjusted models included child sex, maternal age, and maternal atopy. Statistical analyses include linear regression with interaction terms. *$P \leq 0.05$

Reviewer #1 (Remarks to the Author):

The authors have adequately revised the manuscript to address previously misleading statements. This reviewer has no further concerns and supports its publication in this journal.

We sincerely thank the reviewer for the positive evaluation and thoughtful feedback throughout the review process; your insights have significantly contributed to improving the clarity and overall quality of our manuscript.

Reviewer #4 (Remarks to the Author):

The authors have answered my questions. I have no further comments

We sincerely thank the reviewer for the constructive feedback, which has greatly contributed to improving the quality and clarity of our manuscript.
