# Supplementary material for: Prenatal PM2.5 exposure drives epigenetic reprogramming of fetal macrophages linked to atopic dermatitis
Source: Nat Commun. 2026 Aug 8;17:9531. doi: 10.1038/s41467-026-76298-6 (PMC13547331; doi:10.1038/s41467-026-76298-6)
Supplement: Supplementary file 2 [file 41467_2026_76298_MOESM1_ESM.pdf]

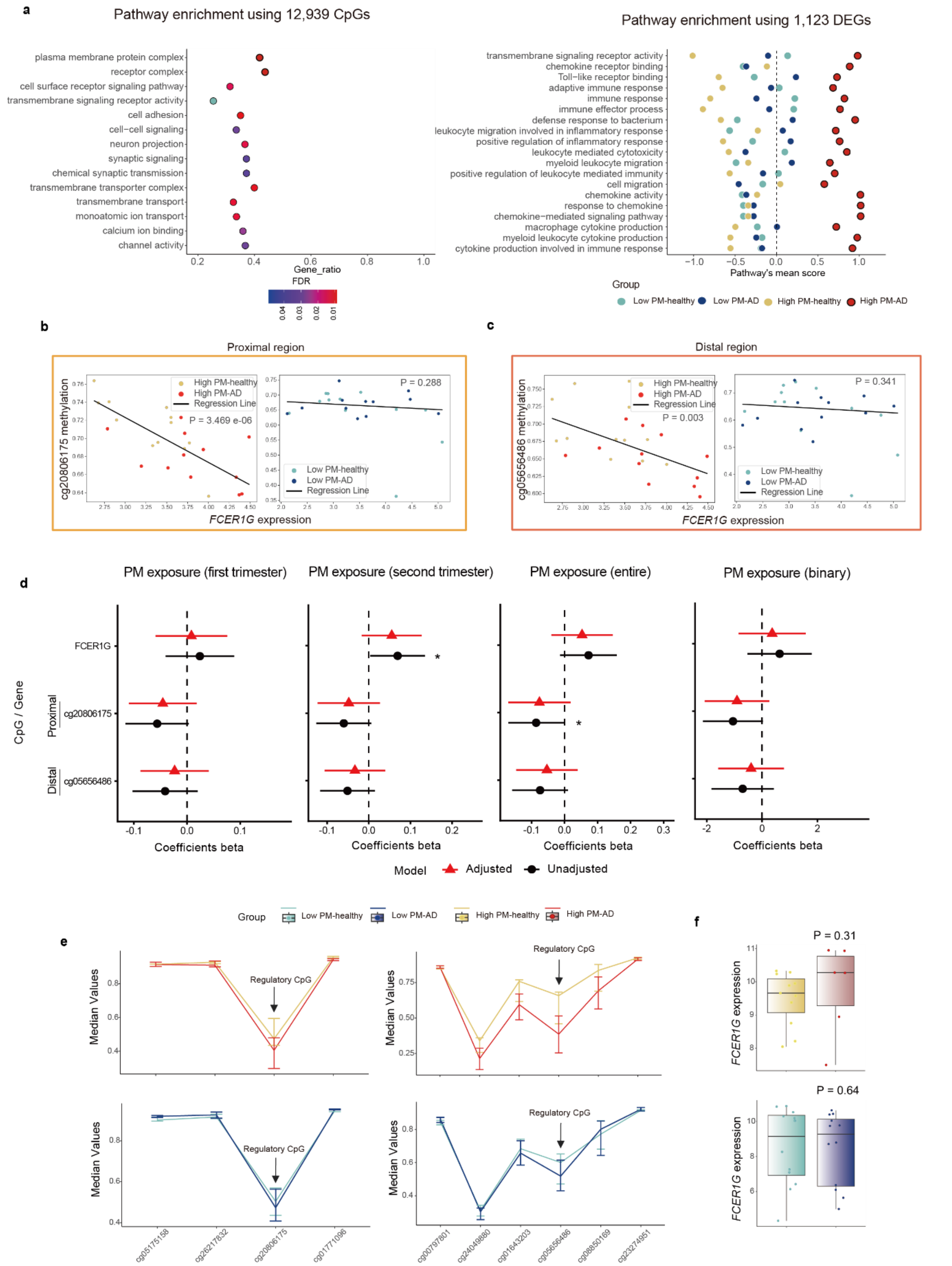

Supplementary Fig. 1. CpG methylation at *FCER1G* regulatory sites and interaction with prenatal PM exposure and AD status. a, Pathway analysis of differentially methylated CpG

sites (DMCs; left) and differentially expressed genes (DEGs; right). The x-axis shows the gene ratio, defined as the proportion of pathway-related genes relative to the total number of genes in a given pathway (left), and mean pathway scores across groups (right). Pathways with an FDR (Benjamini–Hochberg) < 0.05 were included. **b–c,** Correlation plots between proximal (cg20806175) and distal (cg05656486) CpG methylation and *FCER1G* expression in placenta. Each dot represents the High PM exposure–AD group (n = 12 placenta samples), High PM exposure–healthy group (n = 12), Low PM exposure–healthy group (n = 12), and Low PM exposure–AD group (n = 12). **d,** Forest plots represent the interaction coefficients derived from linear regression models (Target ~ PM exposure × AD status) for *FCER1G* expression and DNA methylation at the proximal and distal CpG sites in 48 placenta samples. PM exposure was evaluated sequentially for the first trimester, second trimester, entire pregnancy, and as a binary variable (high vs. low). Adjusted models included child sex, maternal age, and maternal atopy. **e,** Line plots with error bars represent the median DNA methylation levels of CpGs located within ±200 bp of the regulatory CpG (indicated by the arrow) in the proximal (left) and distal (middle) regions from cord blood samples. The CpG IDs (x-axis) are ordered based on chromosomal position. **f,** Box plots illustrate *FCER1G* expression levels across the four groups in cord blood. All cord blood samples were collected from individuals with matched placental samples. Error bars indicate standardized β coefficients with 95% confidence intervals (**d**) and median ± IQR (**e**). Box plots depict the median (central line), interquartile range (box edges), and whiskers extending to 1.5 × IQR (**f**). Statistical analyses include over-representation analysis (**a**, left), Huber regression (**b** and **c**), and linear regression with interaction terms (**d**), and Wilcoxon rank-sum test (**a**, right; High PM–AD group vs. other groups, **e** and **f**). All statistical tests were two-sided. P values in panel **a** are Benjamini–Hochberg–adjusted; all others are nominal (unadjusted). $^*P \leq 0.05$. Exact P-values and source data are provided in the Source Data file.

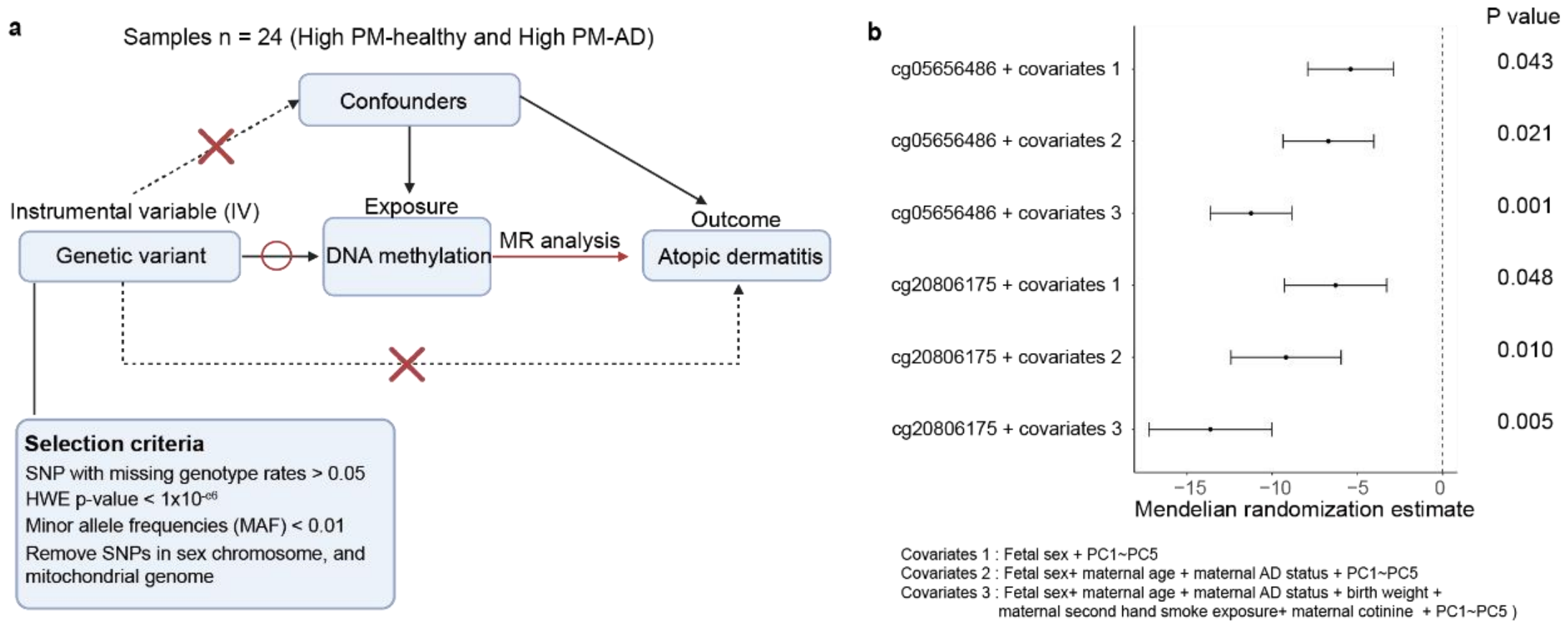

Supplementary Fig. 2. Mendelian Randomization analysis of *FCER1G* regulatory CpG sites. a, Schematic of the Mendelian Randomization (MR) analytic pipeline. The analysis was performed using placental samples from the high prenatal PM exposure group (n = 24 placenta samples) in our cohort. **b,** Error bar plot showing the MR estimates for the CpG sites cg05656486 and cg20806175. P-values corresponding to each estimate are shown to the right of the plot. Covariate combinations included in the analysis are detailed below. Error bars indicate MR estimates ± s.e. Statistical analysis was performed using two-sided instrumental variable regression with the *ivreg* function in R.

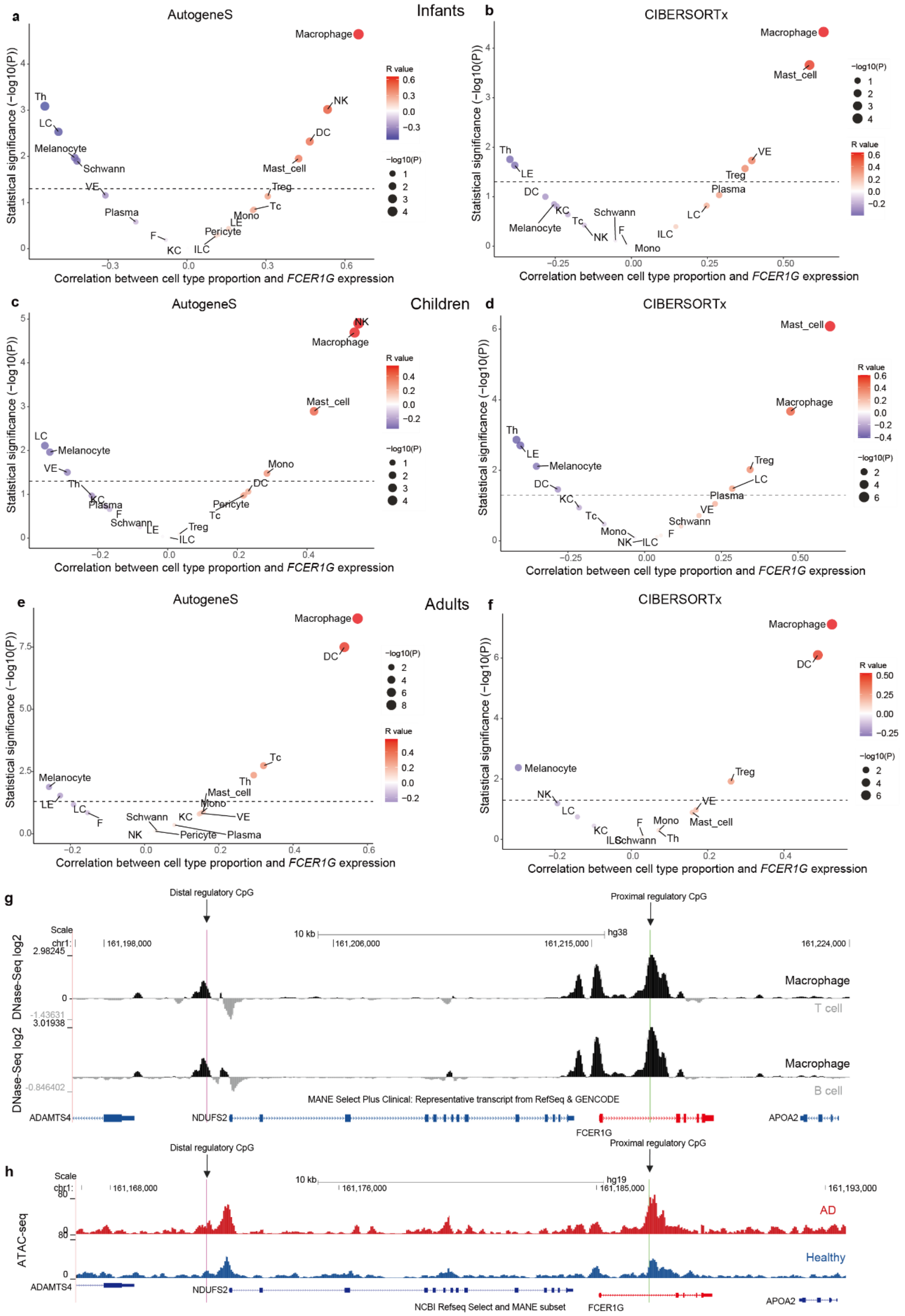

Supplementary Fig. 3. Epigenetic regulation of *FCER1G* and its positive correlation with

macrophages. a–f, Correlation plots between *FCER1G* expression and macrophage proportion across healthy and AD skin samples. Cell type proportions were inferred from skin transcriptomes using AutogeneS (**a**, **c** and **e**) and CIBERSORTx (**b**, **d** and **f**). Panels **a** and **b** represent infant samples (n = 35 samples), **c** and **d** represent children (n = 56 samples), and **e** and **f** represent adults (n = 92 samples). The dashed line along the y-axis indicates $-\log_{10}(P = 0.05)$. **g,** DNase-seq $\log_2$-transformed ratios comparing macrophages to other immune cells (T cells, B cells). Green and purple marks indicate the proximal and distal regulatory CpG sites, respectively. Data were obtained from ENCODE. **h,** Average chromatin accessibility profiles of PBMC-derived monocytes from AD patients (n = 6 samples) and healthy (n = 4 samples) based on ATAC-seq data (GSE255396). Green and purple marks indicate the proximal and distal regulatory CpG sites, respectively. Statistical analyses include two-sided Spearman correlation (**a-f**). P-values shown are nominal (unadjusted). Exact P-values and source data are provided in the Source Data file.

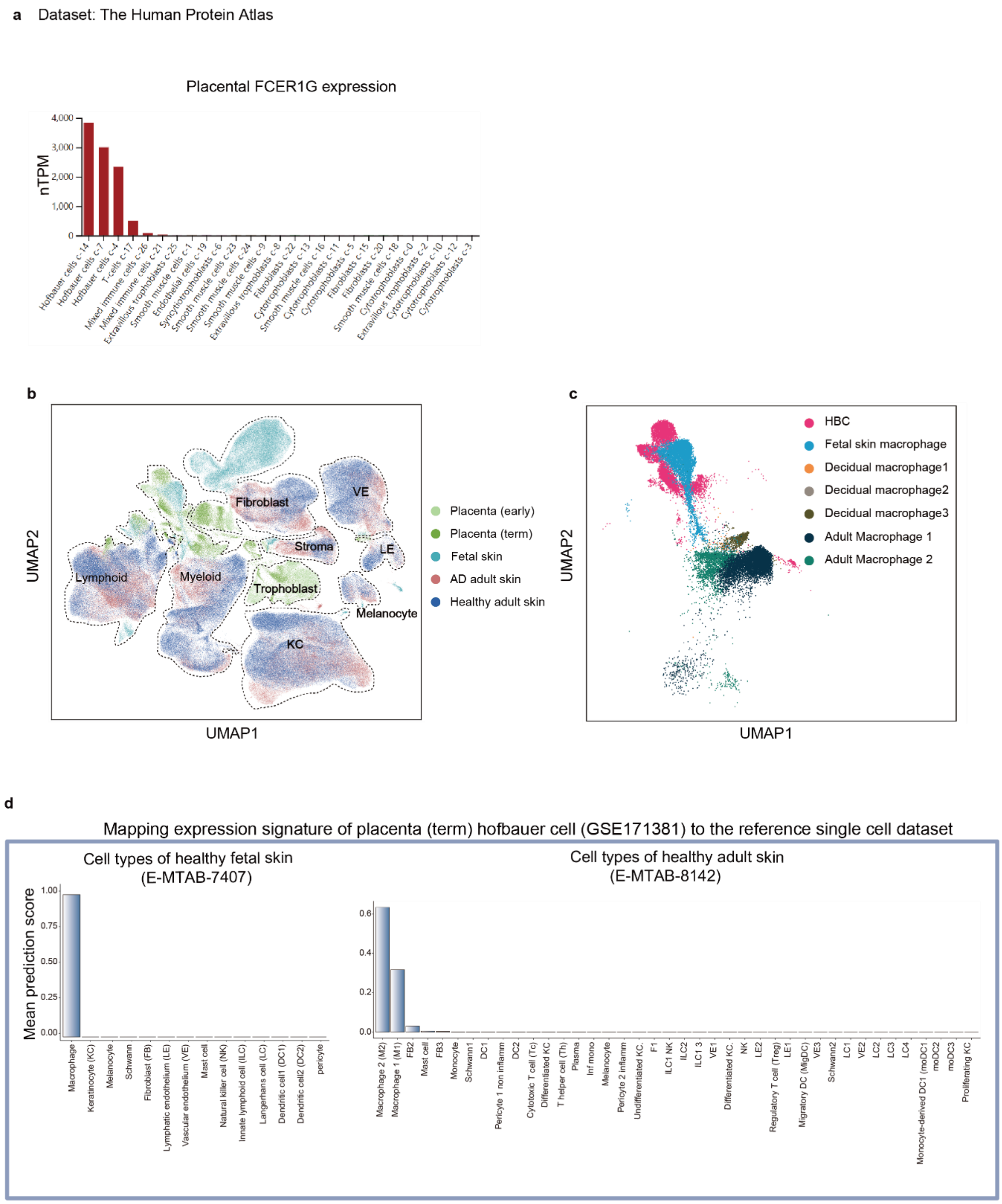

Supplementary Fig. 4. ***FCER1G*** **expression and placental Hofbauer cell state prediction across developmental stages. a,** Bar plots represent *FCER1G* expression across various placental cell types (n = 25,615 cells) from scRNA-seq data. Data accessed from Human Protein Atlas (proteinatlas.org). **b,** Uniform manifold approximation and projection (UMAP)

plots of scRNA-seq data from different developmental stages (placenta early, n = 18,547 cells; placenta term, n = 33,173 cells; fetal skin, n = 44,295 cells; healthy adult skin, n = 192,347 cells; AD adult skin, n = 115,807 cells) from the E-MTAB-6701, GSE171381, E-MTAB-7407, and E-MTAB-8142 datasets. VE, vascular endothelial cell; LE, lymphatic endothelium; KC, keratinocyte. **c,** UMAP plot of Hofbauer cells, fetal skin macrophages, decidual macrophages (maternal side), and adult macrophages. **d,** Bar plots displaying the mean prediction score of term placental Hofbauer cell states, using healthy fetal skin (left) and healthy adult skin (right) as references, derived from the TransferAnchors function in Seurat.

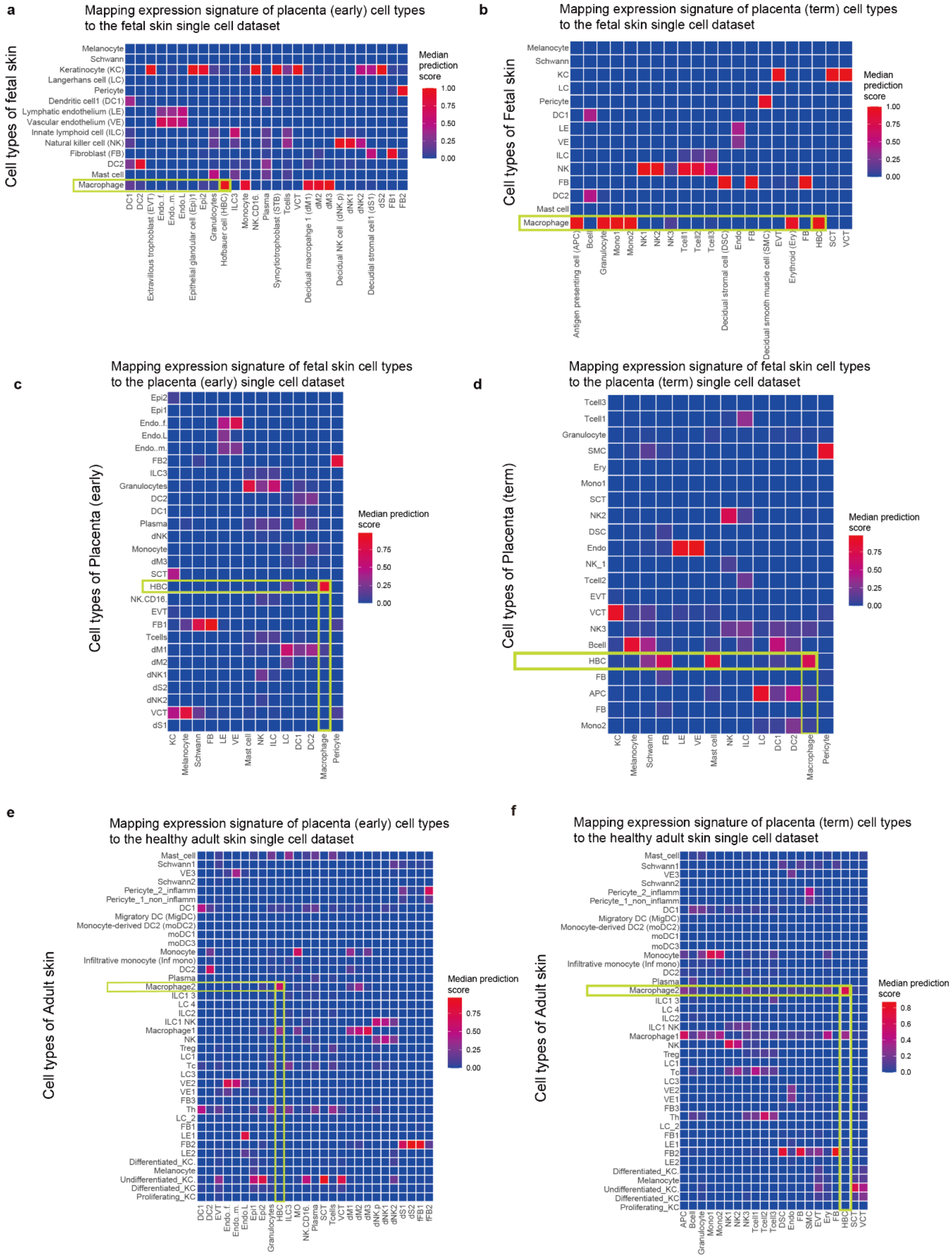

Supplementary Fig. 5. Phenotypic similarity across cells from different developmental

stages. a–b, Heatmaps showing the median prediction scores of early- (**a**) and term-stage (**b**) placental cell states using healthy fetal skin as a reference, derived from the TransferAnchors function in Seurat. **c–d,** Heatmaps showing the median prediction scores of healthy fetal skin cell types using early- (**c**) and term-stage (**d**) placenta as a reference, derived from the TransferAnchors function in Seurat. Hofbauer cells showed high prediction scores across multiple cell types, suggesting their close transcriptional relationship with fetal skin cell types. **e–f,** Heatmaps showing the median prediction scores of early- (**e**) and term-stage (**f**) placental cell states using healthy adult skin as a reference, derived from the TransferAnchors function in Seurat.

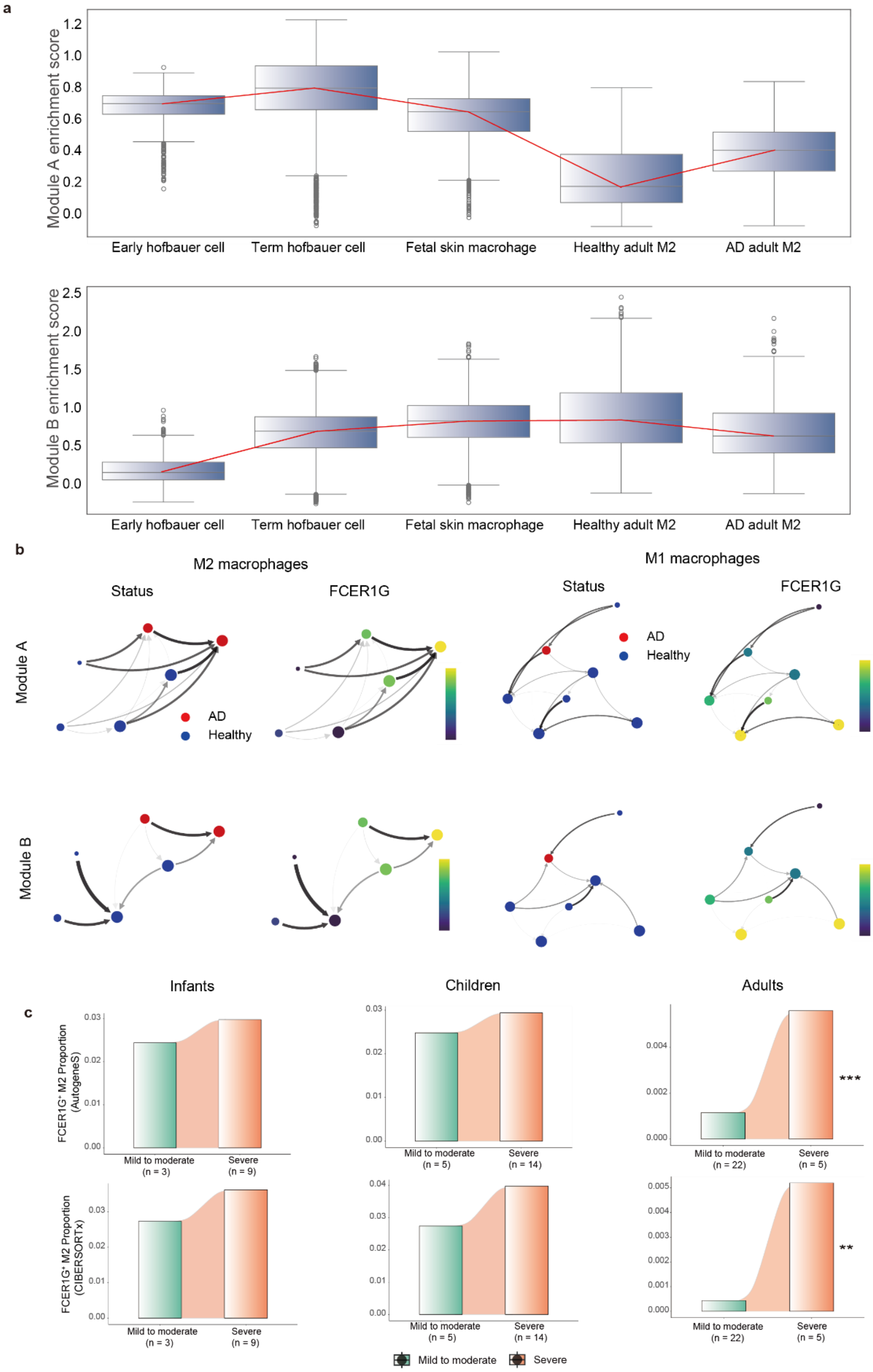

Supplementary Fig. 6. Module scores and macrophage state transitions and *FCER1G*$^{+}$

M2 proportions stratified by SCORAD. a, Box plots showing module scores calculated using scanpy.tl.score_genes across developmental stages, including Hofbauer cells, fetal skin macrophages, and adult M2 macrophages. Each box plot depicts the median (central line), interquartile range (box edges), and whiskers extending to 1.5 × IQR. **b,** The cell state transition graph, based on modules A (top) and B (bottom), displays the AD and healthy status (left) and *FCER1G* gene expression (right) for M1 and M2 macrophages. Node size is proportional to cluster size, edge thickness indicates connection strength, and arrows represent transition direction. Cell state transitions were analyzed using dynamo.pl.state_graph. **c,** Proportion of *FCER1G*$^{+}$ M2 cell types across developmental stages in AD lesional samples, stratified into mild-to-moderate and severe groups based on SCORAD scores (cutoff = 50 for infants and children, 40 for adults), estimated from bulk transcriptomes using AutogeneS (top) and CIBERSORTx (bottom). Sample sizes are indicated within the figure. Statistical analysis includes two-sided likelihood ratio test. (**c**). P-values shown are nominal (unadjusted). Exact P-values and source data are provided in the Source Data file.

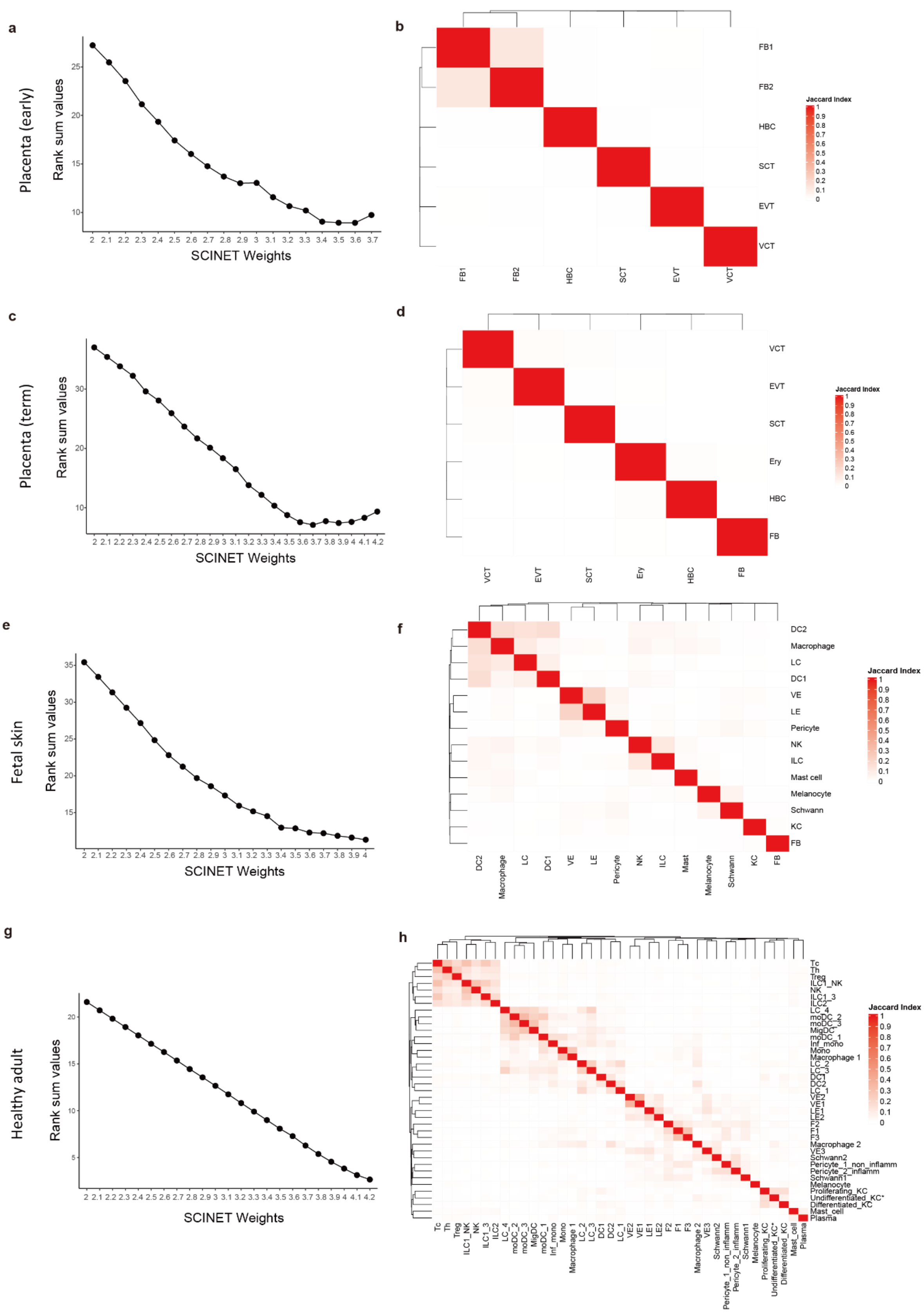

Supplementary Fig. 7. Optimal weight calculation and cell type-specific network

construction. a, c, e, g, The optimal weights for constructing cell type-specific networks in early placenta (**a**), term placenta (**c**), healthy fetal skin (**e**), and healthy adult skin (**g**) were determined by calculating the rank sum values based on SCINET weights. The rank sum values consider two factors: (1) the higher the number of marker genes included, the lower the rank, and (2) the more cell type-specific genes present without overlap, the lower the rank (see Methods for details). The optimal weights were 3.6, 3.7, 4.0, and 4.2, respectively. **b, d, f, h,** Heatmaps represent the Jaccard index, which measures the degree of overlap between the genes of each cell type-specific network corresponding to the optimal weights in **a, c, e, g.**

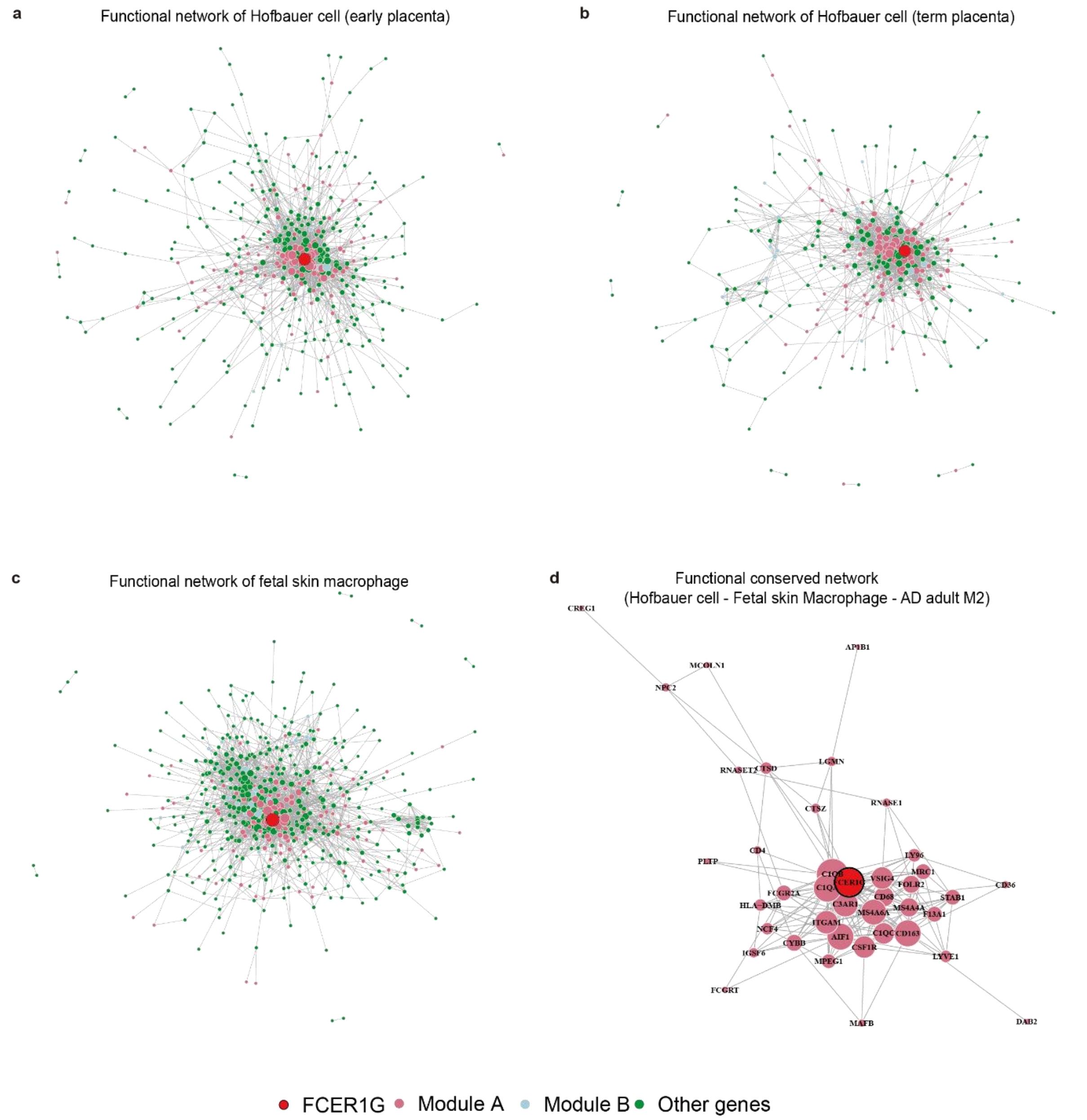

Supplementary Fig. 8. Functional network visualizations of early and term placental Hofbauer cells, fetal skin macrophages, and conserved networks. a, Network visualization of early-stage placental Hofbauer cells. **b,** Term-stage Hofbauer cells. **c,** Fetal skin macrophages. **d,** Conserved networks across Hofbauer cells, fetal skin macrophages, and AD adult M2 macrophages, showing only Module A and *FCER1G*. Node colors represent specific genes or modules. Node size corresponds to the number of interactions for each gene.

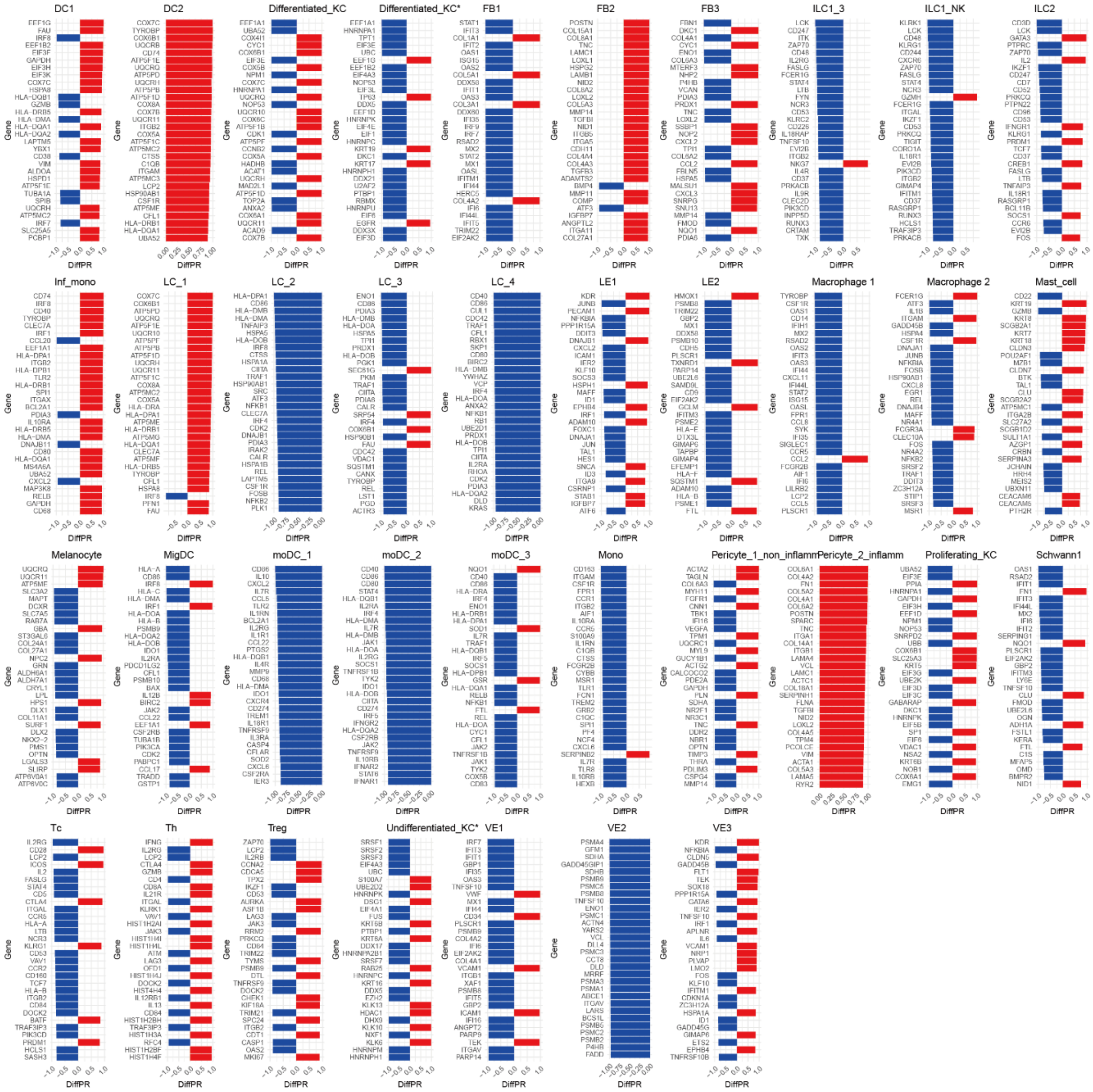

Supplementary Fig. 9. Differential percentile rank (diffPR) of genes in healthy and AD adult cell types. Top 30 genes ranked by differential percentile rank (diffPR) values, comparing healthy and AD adult cell types. Red bars indicate AD-specific central genes in these cell types. The gene list is sorted by P values. Statistics were calculated using the FindDiffHub function from scHumanNet. All statistical tests were two-sided, and P-values shown are nominal (unadjusted). Exact P-values and source data are provided in the Source Data file. DC, dendritic cell; KC, keratinocyte; FB, fibroblast; ILC, innate lymphoid cell; LC,

Langerhans cell; LE, lymphatic endothelial cell; MigDC, migratory dendritic cell; moDC, monocyte-derived dendritic cell; Mono, monocyte; Tc, cytotoxic T cell; Th, helper T cell; VE, vascular endothelial cell.

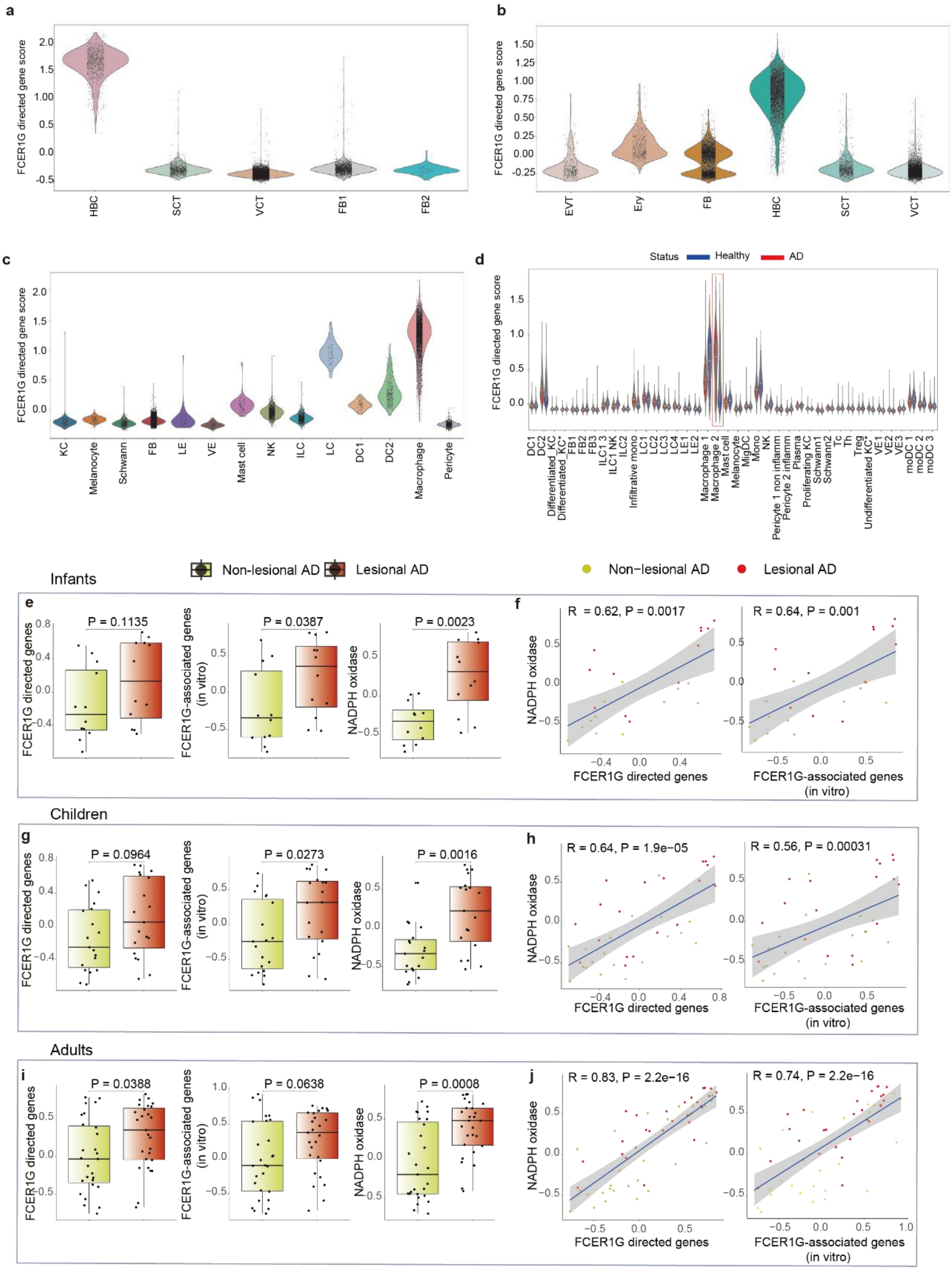

Supplementary Fig. 10. *FCER1G*-directed neighbor gene scores across developmental stages. **a–d**, Violin plots showing *FCER1G*-directed neighbor gene scores calculated using scanpy.tl.score_genes across early-stage placental cell types (**a**), term-stage placental cell types

(**b**), fetal skin cell types (**c**), and healthy and AD adult skin cell types (**d**). Each dot represents an individual cell. The gene set used for this analysis was derived from Fig. 6f. (**e, g, i).** Box plots represent score comparisons between lesional and non-lesional AD skin across developmental stages: infants (n = 12 samples per group), children (n = 19 per group), and adults (n = 27 samples per group). *FCER1G*-directed gene scores (left) were derived from the network in Fig. 6f. In vitro–identified *FCER1G*-associated gene scores (middle) from Fig. 6j, NADPH oxidase complex gene scores (right) calculated using the expression of *CYBA*, *CYBB*, *NCF1*, *NCF2*, *NCF4*, *RAC1*, and *RAC2*. **f, h, j**, Correlation plots illustrate the association between each *FCER1G*-related score and NADPH oxidase score. Red dots represent AD lesional samples and yellow dots represent non-lesional samples. Box plots depict the median (central line), interquartile range (box edges), and whiskers extending to 1.5 × IQR (**e**, **g**, **i**). Statistical analyses include Wilcoxon rank-sum test (**e, g, i**) and Spearman correlation (**f, h, j**). All statistical tests were two-sided.

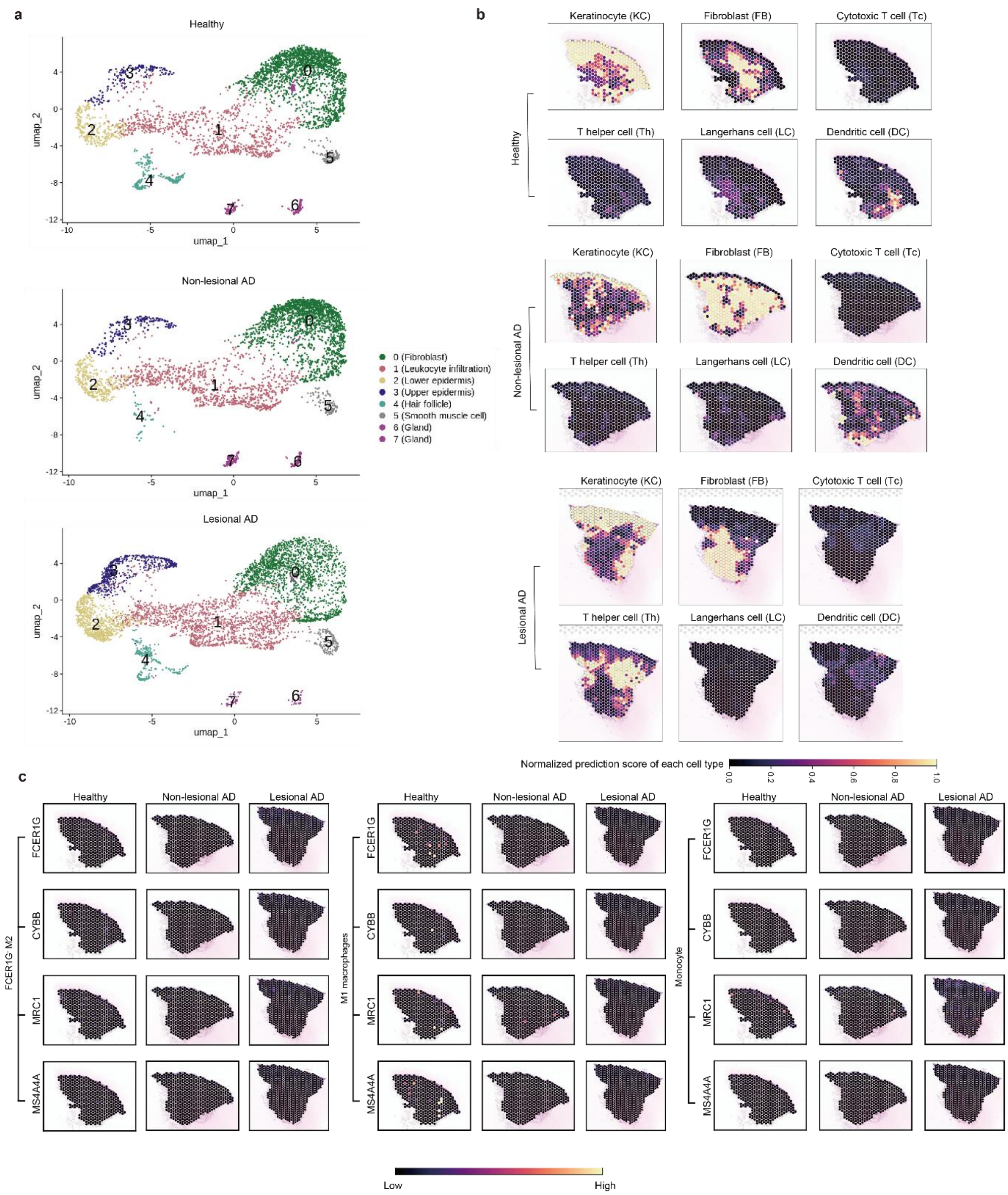

Supplementary Fig. 11. Spatial transcriptome analysis and cell type-specific prediction scores across healthy, non-lesional, and lesional AD. a, UMAP plots of total spatial transcriptome spots (healthy, n = 6; non-lesional AD, n = 5; lesional AD, n = 7) from the GSE197023 dataset. **b,** Normalized prediction scores of each cell type (Keratinocyte,

Fibroblast, Cytotoxic T cell, T helper cell, Langerhans cell, Dendritic cell), estimated using cell2location, across healthy, non-lesional and lesional AD sections. **c,** Gene expression levels of *FCER1G*, *CYBB*, *MRC1*, and *MS4A4A* in *FCER1G*$^{-}$ M2 macrophages, M1 macrophages, and monocytes across healthy, non-lesional, and lesional AD sections.

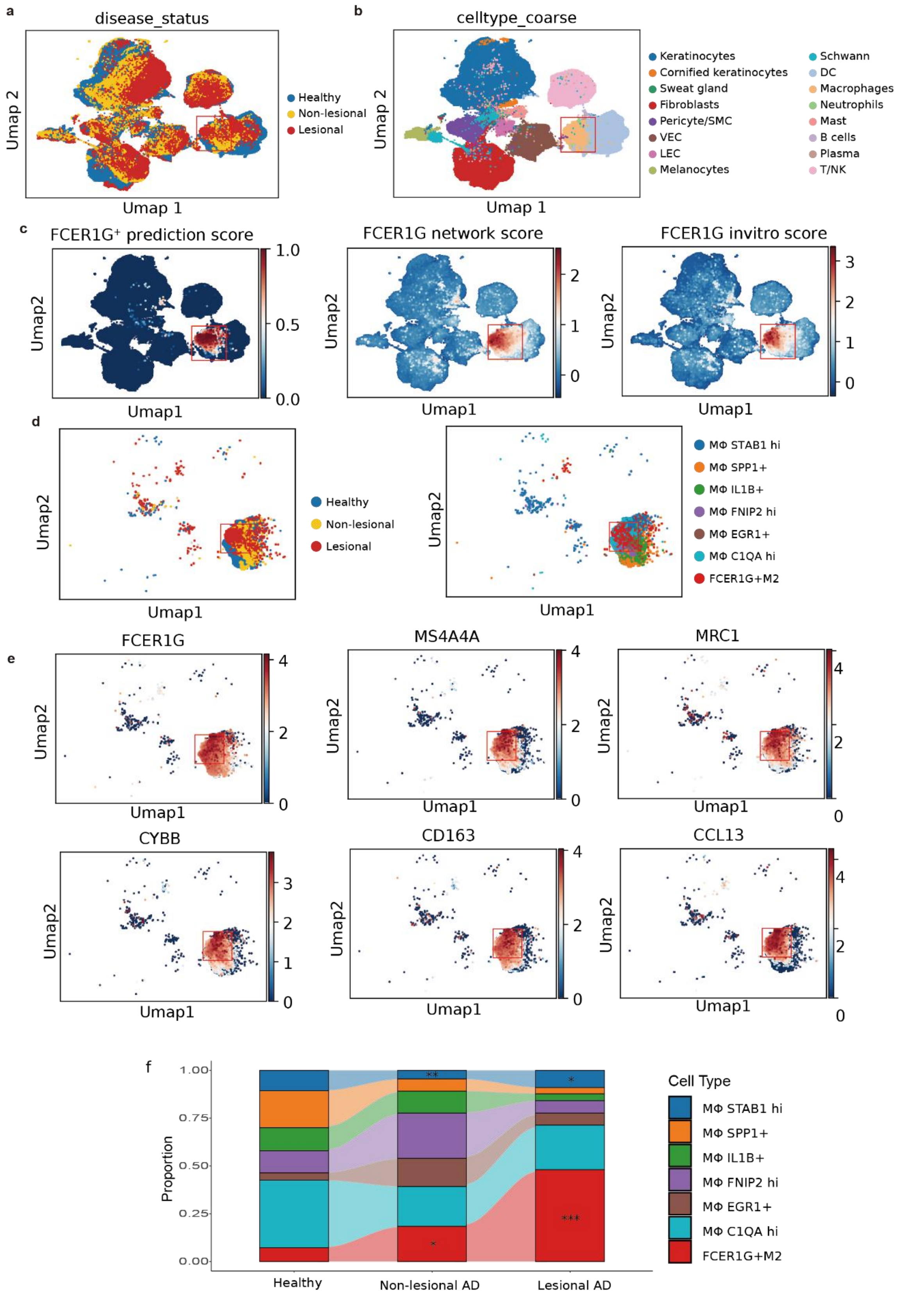

Supplementary Fig. 12. Identification of *FCER1G*⁺ M2 macrophages and their

representative gene signatures within the AD skin atlas by Fiskin et al. a, b, UMAP plots display the distribution of cells colored by atopic dermatitis (AD) status (**a**) and by each cell state as defined in the Fiskin et al. skin atlas (**b**). **c,** UMAP projections showing *FCER1G*$^{+}$ M2 macrophage–associated gene signature scores across the cellular landscape. The *FCER1G*$^{+}$ M2 prediction score was calculated using the FindTransferAnchors function in Seurat (left). The *FCER1G* network score was derived from the inferred regulatory network centered on *FCER1G* (Fig. 6f; middle). The *FCER1G* score was computed using scanpy.tl.score_genes based on the expression of *FCER1G*, *MS4A4A*, *MRC1*, *CYBB*, *CD163*, and *CCL13* (right). *MS4A4A*, *MRC1*, and *CYBB* were selected based on in vitro PM exposure validation experiments (Fig. 6**i**, **j**), *CD163* was included as a canonical M2 macrophage marker, and *CCL13* was identified as an *FCER1G*$^{+}$ M2–associated ligand through cell–cell interaction analysis. **d,** UMAP visualization restricted to macrophages, showing AD status and corresponding macrophage subclusters. **e,** Expression levels of representative *FCER1G*$^{+}$ M2 marker genes across macrophages. **f,** Proportional composition of macrophage subtypes in the Fiskin atlas, compared across AD disease states. Statistical analyses included generalized linear modeling with a quasibinomial distribution for proportion comparisons and likelihood ratio tests assessing differences of Lesional and Non-lesional AD groups relative to Healthy (**f**). All statistical tests were two-sided, and P-values shown are nominal (unadjusted). $^{*}P \leq 0.05$, $^{**}P \leq 0.001$, $^{***}P \leq 0.0001$. Exact P-values and source data are provided in the Source Data file.

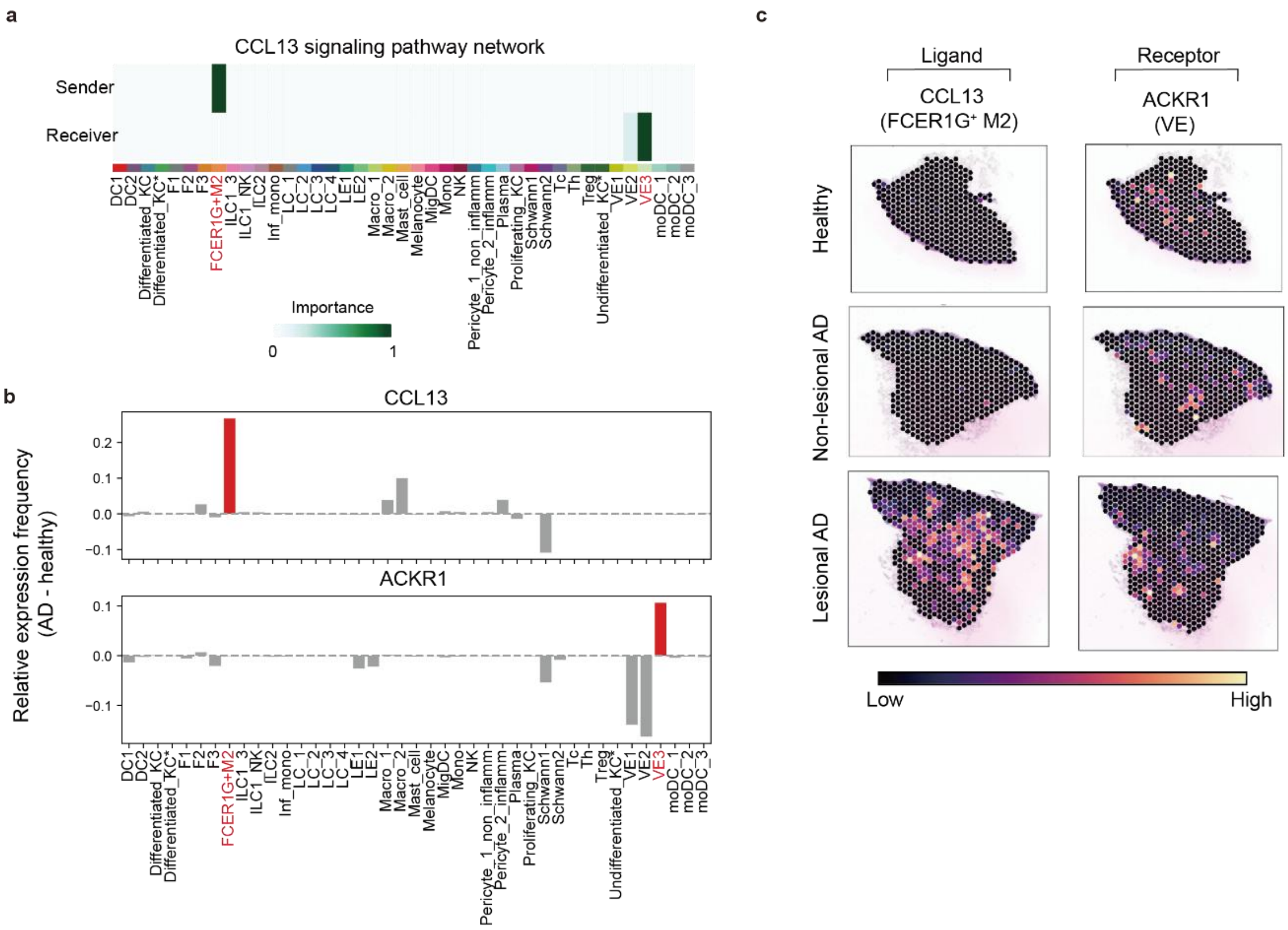

Supplementary Fig. 13. *FCER1G*⁺ M2 macrophages mediate cell–cell interactions as ligand-producing cells via CCL13 in AD skin. a, Heatmaps showing the expression levels of ligands and their corresponding receptors across each cell type. **b,** Bar plots represent the difference in the proportion of cells expressing CCL13 (top) and ACKR1 (bottom) in each cell type. **c,** Spatial expression maps of the most enriched sender and receiver cell types involved in the CCL13 interactions, across healthy, non-lesional AD, and lesional AD skin sections.
