# Supplementary material for: Prenatal PM2.5 exposure drives epigenetic reprogramming of fetal macrophages linked to atopic dermatitis
Source: Nat Commun. 2026 Aug 8;17:9531. doi: 10.1038/s41467-026-76298-6 (PMC13547331; doi:10.1038/s41467-026-76298-6)
Supplement: Supplementary file 3 [file 41467_2026_76298_MOESM2_ESM.pdf]

Description of Additional Supplementary Files

Supplementary Data 1. Study population

Supplementary Data 2. The list of differentially methylated CpG sites and differentially expressed genes in the placenta.

Supplementary Data 3. Mendelian randomization results between PM2.5-induced methylation changes and AD status.

Supplementary Data 4. Characteristics of the subjects from the four groups in the placenta based omics study

Supplementary Data 5. Characteristics of the subjects from the four groups in the cord blood based omics study.

Supplementary Data 6. The list of conserved genes across the Hofbauer cell, fetal skin macrophages, and AD M2 macrophages.
