# Supplementary material for: Prenatal PM2.5 exposure drives epigenetic reprogramming of fetal macrophages linked to atopic dermatitis
Source: Nat Commun. 2026 Aug 8;17:9531. doi: 10.1038/s41467-026-76298-6 (PMC13547331; doi:10.1038/s41467-026-76298-6)
Supplement: Supplementary file 4 [file 41467_2026_76298_MOESM9_ESM.pdf]

nature portfolio

Corresponding author(s): KWONEEL KIM

Last updated by author(s): Sep 8, 2025

# Reporting Summary

Nature Portfolio wishes to improve the reproducibility of the work that we publish. This form provides structure for consistency and transparency in reporting. For further information on Nature Portfolio policies, see our Editorial Policies and the Editorial Policy Checklist.

## Statistics

For all statistical analyses, confirm that the following items are present in the figure legend, table legend, main text, or Methods section.

| n/a | Confirmed | |
|---|---|---|
| ☐ | ☒ | The exact sample size (*n*) for each experimental group/condition, given as a discrete number and unit of measurement |
| ☐ | ☒ | A statement on whether measurements were taken from distinct samples or whether the same sample was measured repeatedly |
| ☐ | ☒ | The statistical test(s) used AND whether they are one- or two-sided<br>*Only common tests should be described solely by name; describe more complex techniques in the Methods section.* |
| ☐ | ☒ | A description of all covariates tested |
| ☐ | ☒ | A description of any assumptions or corrections, such as tests of normality and adjustment for multiple comparisons |
| ☐ | ☒ | A full description of the statistical parameters including central tendency (e.g. means) or other basic estimates (e.g. regression coefficient) AND variation (e.g. standard deviation) or associated estimates of uncertainty (e.g. confidence intervals) |
| ☐ | ☒ | For null hypothesis testing, the test statistic (e.g. *F*, *t*, *r*) with confidence intervals, effect sizes, degrees of freedom and *P* value noted<br>*Give P values as exact values whenever suitable.* |
| ☒ | ☐ | For Bayesian analysis, information on the choice of priors and Markov chain Monte Carlo settings |
| ☒ | ☐ | For hierarchical and complex designs, identification of the appropriate level for tests and full reporting of outcomes |
| ☐ | ☒ | Estimates of effect sizes (e.g. Cohen's *d*, Pearson's *r*), indicating how they were calculated |

*Our web collection on statistics for biologists contains articles on many of the points above.*

## Software and code

Policy information about availability of computer code

| | |
|---|---|
| Data collection | All data collection procedures and analysis software used in this study are described in the Methods section.<br>The code used for data analysis in this study is available at GitHub (https://github.com/sysbioinfo khu/PM_AD) or Zenodo (https://doi.org/10.5281/zenodo.14998809). |
| Data analysis | The following software and packages were used in this study: missMethyl (v1.38.0, R package), GSVA (v1.52.3, R package), gseapy (v1.1.5, Python), MethReg (v1.14.0, R package), TFBStools (v1.42.0, R package), SAMtools (v1.10), HiCExplorer (v3.7.6), hicstraw (v1.3.1), deepTools (v3.5.6), ivreg (v0.6.4, R package), Scanpy (v1.9.3, Python), Seurat (v5.3.0, R package), CIBERSORTx (Docker), AutogeneS (v1.0.4, Python), scVI (v1.1.2, Python), Velocyto (v0.17.17, Python), CellDancer (v1.1.7, Python), Dynamo (v1.3.0, Python), scHumanNet (v0.1.0, Python), cell2location (v0.1.4, Python), and CellChat (v2.2.0, R package). |

For manuscripts utilizing custom algorithms or software that are central to the research but not yet described in published literature, software must be made available to editors and reviewers. We strongly encourage code deposition in a community repository (e.g. GitHub). See the Nature Portfolio guidelines for submitting code & software for further information.

## Data

Policy information about availability of data

All manuscripts must include a data availability statement. This statement should provide the following information, where applicable:

- Accession codes, unique identifiers, or web links for publicly available datasets
- A description of any restrictions on data availability
- For clinical datasets or third party data, please ensure that the statement adheres to our policy

Placental and cord blood methylation and transcriptome data from the COCOA cohort, stratified by particulate matter 2.5 exposure and atopic dermatitis status, were made publicly available in Zenodo (https://doi.org/10.5281/zenodo.14998855). All other publicly available datasets used in this study are described in the Methods section.

## Research involving human participants, their data, or biological material

Policy information about studies with human participants or human data. See also policy information about sex, gender (identity/presentation), and sexual orientation and race, ethnicity and racism.

| | |
|---|---|
| Reporting on sex and gender | In this study, sex was included as one of the covariates, with classification based on birth records. The variable was categorized as male or female. A total of 986 participants were included, comprising 516 males and 470 females. Sex information is available in the source data, and consent for sharing individual-level data was obtained in accordance with ethical guidelines. Sex, defined by biological attributes assigned at birth, was used rather than gender, as the primary objective was to investigate biological sex differences and their effects. |
| Reporting on race, ethnicity, or other socially relevant groupings | Maternal educational level was used as a socioeconomic indicator, given its relevance as a socially meaningful categorization variable. It is a well-established proxy for socioeconomic status and is known to influence various child development outcomes. Data on maternal education were obtained from the parental questionnaire, which included specific items on the highest level of education completed by the mother. Responses were categorized into distinct groups (e.g., "High school," "College graduate," and "Postgraduate school") based on standard educational classifications. To account for potential confounding, the statistical analysis was adjusted for sex, parental allergic history, maternal age, maternal education, maternal body mass index, secondhand smoke exposure during pregnancy and infancy. This adjustment minimized the potential influence of these confounders on the observed associations. |
| Population characteristics | The study population consisted of children with a mean age of 6 years. Children with available PM2.5 exposure data from the prenatal period through age 3 who were followed for the first three years of life were included in the analysis, and the primary outcome of interest, atopic dermatitis, was observed in 37.5% of participants. |
| Recruitment | Participants were recruited from five medical centers in Seoul (Asan Medical Center, Samsung Medical Center, Severance Hospital, CHA Gangnam Medical Center, and Seoul National University Hospital) among pregnant women presenting before 26 weeks of gestation. Inclusion criteria were absence of high-risk conditions, intention to deliver at one of the affiliated hospitals, and residence in Seoul. After birth, infants were enrolled if they met eligibility criteria, excluding those born preterm or with congenital anomalies. Self-selection bias may have occurred, as health-conscious women could have been more likely to participate. Selection bias is also a concern due to the exclusion of high-risk pregnancies and preterm births, which may limit the generalizability of the findings. Additionally, location bias may affect representativeness, as all participants were recruited from a single metropolitan area. Nonetheless, the primary objective of the COCOA study was to investigate general child health, which guided the recruitment strategy to avoid a high-risk cohort. |
| Ethics oversight | The study protocol was approved by the Institutional Review Boards of the following institutions: Asan Medical Center (IRB No. 2008–0616), Samsung Medical Center (IRB No. 2009-02-021), Severance Hospital (IRB No. 4-2008-0588), CHA Gangnam Medical Center (IRB No. 2010–010), and Seoul National University Hospital (IRB No. H-1401-086-550).Written informed consent was obtained from the parents of each child. No participant compensation was provided. |

Note that full information on the approval of the study protocol must also be provided in the manuscript.

# Field-specific reporting

Please select the one below that is the best fit for your research. If you are not sure, read the appropriate sections before making your selection.

[x] Life sciences   [ ] Behavioural & social sciences   [ ] Ecological, evolutionary & environmental sciences

For a reference copy of the document with all sections, see nature.com/documents/nr-reporting-summary-flat.pdf

# Life sciences study design

All studies must disclose on these points even when the disclosure is negative.

| | |
|---|---|
| Sample size | The sample size for this epidemiologic and epigenomic study was determined based on the availability of data on PM2.5 exposure and atopic dermatitis. No formal statistical sample size calculation was conducted prior to the study. Instead, the sample size was defined by the amount of available data for both the exposure (PM2.5) and the outcome (atopic dermatitis). Given the size of the dataset, we consider the sample sufficient to detect meaningful associations between PM2.5 exposure and the occurrence of atopic dermatitis. For in vitro experiments, three |

independent biological replicates were performed for each condition to assess variability and statistical significance. Sample sizes were determined based on standard field practices and prior experience with these experimental systems. Although no formal power calculation was conducted, these group sizes are considered sufficient to detect biologically meaningful differences.

| | |
|---|---|
| Data exclusions | No data were excluded from the analyses. |
| Replication | This study is an observational epidemiologic study rather than an experimental one. To date, the findings have not been replicated in other studies, and their reproducibility remains to be evaluated in future research. |
| Randomization | This study is an observational epidemiologic study and did not involve random allocation or assignment to experimental groups. Participants were included based on their exposure to PM2.5 and the presence of atopic dermatitis. Covariates were controlled for through statistical adjustments during the analysis. |
| Blinding | This study is an observational epidemiologic study, not an experimental one. Consequently, blinding was not applicable to the study design. As there was no group allocation or random assignment of participants, blinding during data collection and analysis was not required. |

# Reporting for specific materials, systems and methods

We require information from authors about some types of materials, experimental systems and methods used in many studies. Here, indicate whether each material, system or method listed is relevant to your study. If you are not sure if a list item applies to your research, read the appropriate section before selecting a response.

## Materials & experimental systems

| n/a | Involved in the study |
|---|---|
| ☒ | ☐ Antibodies |
| ☐ | ☒ Eukaryotic cell lines |
| ☒ | ☐ Palaeontology and archaeology |
| ☒ | ☐ Animals and other organisms |
| ☒ | ☐ Clinical data |
| ☒ | ☐ Dual use research of concern |
| ☒ | ☐ Plants |

## Methods

| n/a | Involved in the study |
|---|---|
| ☒ | ☐ ChIP-seq |
| ☒ | ☐ Flow cytometry |
| ☒ | ☐ MRI-based neuroimaging |

## Eukaryotic cell lines

Policy information about cell lines and Sex and Gender in Research

| | |
|---|---|
| Cell line source(s) | THP-1 (TIB-202TM) was purchased from the American Type Culture Collection (ATCC, Manassas, VA, USA) |
| Authentication | ATCC comprehensively performs authentication and quality-control tests on all distribution lots of cell lines. |
| Mycoplasma contamination | THP-1 cells were not tested for mycoplasma contamination. |
| Commonly misidentified lines (See ICLAC register) | No commonly misidentified cell lines were used in the study. |

## Plants

| | |
|---|---|
| Seed stocks | N/A |
| Novel plant genotypes | N/A |
| Authentication | N/A |
